# Mixture Effects of Metals, PCBs, Dioxins, and Furans on Liver Function

**DOI:** 10.3390/toxics14050418

**Published:** 2026-05-11

**Authors:** Bolanle Akinyemi, Emmanuel Obeng-Gyasi

**Affiliations:** 1 Department of Built Environment, North Carolina A&T State University, Greensboro, NC 27411, USA; 2 Environmental Health and Disease Laboratory, North Carolina A&T State University, Greensboro, NC 27411, USA

**Keywords:** liver biomarkers, liver dysfunction, environmental exposure, hepatotoxicity, chemical mixtures, heavy metals, persistent organic pollutant, toxic equivalency, mixtures modeling, quantile G-computation

## Abstract

Quantifying the mixture effects on humans exposed remains challenging because mixture components are correlated and may act bidirectionally by exhibiting nonlinear dose-response relationships, which may contribute to subclinical organ dysfunction. The liver is a vital organ in the body with broad functions, making it vulnerable to injury as it is the first organ exposed to circulating toxicants, which can precipitate hepatic damage. Our study’s objective was to evaluate the combined and component-specific associations of a multi-chemical exposure mixture of heavy metals, polychlorinated biphenyls (PCBs), polychlorinated dibenzo-p-dioxins (dioxins), and polychlorinated dibenzofurans (furans), with liver biomarkers, and to compare concentration-based results with the toxic equivalent (TEQ) potency of the weighted results for dioxin-like compounds. In an unweighted analytic sample of U.S. adults from NHANES 2003–2004 with 947 complete cases, we examined heavy metals (cadmium, lead, and mercury), PCBs (12 congeners), dioxins (7 congeners), and furans (10 congeners) in relation to eight liver biomarkers (albumin, ALP, ALT, AST, GGT, LDH, total bilirubin, and total protein). We applied multi-exposure linear regression, weighted quantile sum (WQS) regression, quantile g-computation (qgcomp), and Bayesian kernel machine regression (BKMR), with parallel TEQ-based models using WHO 2005 TEFs for dioxin-like PCBs, dioxins, and furans. Across mixture methods, the mixture structure was chemically sparse, with a limited set of recurring contributors. Total bilirubin showed the most consistent positive mixture association across qgcomp and BKMR and persisted under TEQ weighting, with prominent PCB- and dioxin-like contributions (notably PCB81/PCB TEQs and dioxin-related components). Albumin demonstrated inverse mixture patterns in BKMR and TEQ-BKMR, with dioxin-like components (notably Dioxin3 and Dioxin3_TEQ) repeatedly emerging as key drivers. For ALT, ALP, AST, GGT, LDH, and total protein, overall mixture effects were frequently attenuated or null in qgcomp despite structured component weights, indicating bidirectional sub-mixtures and internal counterbalancing. BKMR PIPs similarly concentrated on a small number of dominant predictors (e.g., lead for ALP, mercury for ALT, PCB28 for AST, and cadmium and PCB189 for LDH), while interaction summaries provided limited evidence of stable non-additivity. Using multiple complementary mixture methods, we identified outcome-specific mixture patterns suggesting hepatobiliary vulnerability. TEQ concordance supports toxicological relevance of the dioxin-like axis, while metals and non–dioxin-like mechanisms likely contribute additional pathways.

## 1. Introduction

The liver is a vital organ that performs a multitude of physiological functions essential for maintaining homeostasis [1]. It is a central hub for macronutrient metabolism, detoxification of xenobiotics, protein synthesis, bile production, and endocrine signaling [1,2]. This broad function, ranging from processing dietary nutrients to clearing toxins, underscores the liver’s importance in overall health [1]. However, the same attributes that make the liver indispensable also render it vulnerable to injury. For instance, its role in metabolizing drugs and other foreign compounds means that the liver is often the first organ exposed to circulating toxicants, which can precipitate hepatic damage [3].

Liver disease is a major public health concern worldwide, with high morbidity and mortality. Globally, approximately 2 million deaths per year are attributed to liver disease, largely due to complications of cirrhosis, liver cancer, and viral hepatitis [2,4]. Liver diseases encompass a broad spectrum of etiologies, including chronic viral hepatitis (hepatitis A, B, C, D, and E), alcohol associated liver disease (ALD), and metabolic dysfunction-associated fatty liver disease (MAFLD), a term recently introduced to replace non-alcoholic fatty liver disease (NAFLD) [5]. Other causes include autoimmune hepatitis, cholestatic conditions, drug-induced liver injury, and hereditary disorders, such as Wilson’s disease, and hepatic malignancies [6,7]. These conditions often have insidious, progressive courses that can culminate in end-stage liver failure. Advanced liver disease leads to severe consequences, such as acute liver failure, cirrhosis (compensated or decompensated), portal hypertension with variceal hemorrhage, ascites, hepatic encephalopathy, and hepatocellular carcinoma, frequently necessitating liver transplantation [4,8]. Liver thus imposes a substantial global health burden and economic cost [8]. Liver dysfunction can also occur secondary to systemic illnesses. For example, congestive heart failure may cause chronic passive congestion of the liver, and various malignancies or connective tissue diseases can infiltrate or inflame hepatic tissue [7]. Moreover, medications used to treat such conditions may induce liver injury, compounding the hepatic risks.

Environmental exposures have emerged as significant, modifiable risk factors for liver dysfunction. The liver’s central role in detoxification makes it especially susceptible to damage from a range of environmental toxicants, including heavy metals and persistent organic pollutants (POPs) [9]. Humans are exposed to these contaminants through various pathways, including industrial emissions, polluted air and water, dietary intake of contaminated food, and contact with hazardous waste. Chronic exposure to toxic metals like cadmium (Cd), lead (Pb), and mercury (Hg), as well as organic pollutants such as polychlorinated biphenyls (PCBs), polychlorinated dibenzo-p-dioxins (dioxins) and polychlorinated dibenzofurans (furans), has been linked to adverse liver outcomes. These exposures can provoke oxidative stress, inflammation, fibrosis, and hepatocellular carcinoma [10,11].

Heavy metals are well-documented hepatotoxins with established mechanisms of liver injury. Cadmium, for example, accumulates in the liver and generates reactive oxygen species, leading to lipid peroxidation of cell membranes, DNA damage, and hepatocyte apoptosis [11,12,13,14].

Lead primarily disrupts the liver’s antioxidant defenses, notably by depleting glutathione and other thiols, which impairs the detoxification of reactive intermediates and promotes necroinflammation in hepatic tissue [15,16,17]. Mercury (particularly in its organic form, methylmercury) has a high affinity for sulfhydryl groups on proteins, enabling it to interfere with enzymatic and mitochondrial function in hepatocytes and exacerbate liver injury [18,19]. These molecular insults translate into measurable clinical effects. For instance, acute high-level metal exposure can cause dramatic elevations in liver enzymes, while chronic lower-level exposure contributes to subtle liver dysfunction over time. Epidemiological evidence corroborates these toxicological findings: a recent meta-analysis reported that higher Cd exposure is associated with increased risk of early liver disease [20], and population studies have observed positive correlations between blood lead or mercury levels and elevated liver enzymes [21].

In addition to heavy metals, POPs, such as PCBs, dioxins, and furans, pose significant risks to liver health due to their persistence in the environment and propensity to bioaccumulate. PCBs are a class of chlorinated compounds once widely used in industrial applications, and although banned in many countries, they remain prevalent as environmental contaminants [22]. PCBs accumulate in adipose tissue and the liver, where they can activate the aryl hydrocarbon receptor (AhR), a ligand-activated transcription factor. AhR activation by PCBs leads to upregulation of phase I metabolizing enzymes (cytochrome P450s), resulting in enhanced generation of reactive metabolites and oxidative stress, as well as dysregulation of lipid metabolism and hepatic steatosis [22,23,24,25]. For example, exposure to certain PCB congeners has been shown to induce fatty changes in the liver and promote inflammation via pathways involving oxidative damage and altered gene expression in hepatocytes [24,25].

Dioxins (e.g., 2,3,7,8-tetrachlorodibenzo-p-dioxin. TCDD) and structurally related furans are byproducts of various combustion processes and industrial chemical manufacturing. These compounds are considered among the most toxic environmental pollutants and, similar to PCBs, exert many of their effects through binding to the AhR. Dioxin-AhR binding triggers a cascade of events, including the activation of pro-inflammatory transcription factors, such as Nuclear factor-kappa B (NF-kB), and increased production of cytokines like tumor necrosis factor-alpha (TNF-α), which together contribute to hepatocyte injury, apoptosis, and fibrosis [26,27]. The liver’s response to dioxin and furan exposure often manifests as a cholestatic pattern of injury, with damage to bile canaliculi and accumulation of toxic bile components, alongside general hepatocellular toxicity. Epidemiological studies have linked chronic exposure to dioxin-like compounds with a higher prevalence of non-alcoholic fatty liver disease and perturbation in liver enzyme levels [28,29,30].

The data, combined with experimental evidence, underscore the hepatotoxic potential of dioxins and furans even at low environmental concentrations. Notably, some persistent pollutants can induce specific liver enzyme patterns. For instance, certain halogenated hydrocarbons (including some PCB congeners) chronically induce gamma-glutamyl transferase (GGT) activity, and dioxin-like toxicants have been associated with elevated alkaline phosphatase and bilirubin due to cholestatic effects [31,32]. The standard panel of liver function tests, such as alanine aminotransferase (ALT), aspartate aminotransferase (AST), alkaline phosphatase (ALP), GGT, bilirubin, albumin, and total protein, thus serve as an important set of biomarkers for detecting and monitoring toxicant-associated liver injury [33,34,35]. Each of these analytes provides insight into different aspects of hepatic function. For example, elevations in ALT and AST indicate hepatocellular damage, whereas ALP and GGT elevations suggest cholestatic or enzyme induction effects, and declines in albumin reflect impaired synthetic capacity [31,32,33,34]. In clinical practice, patients with known or suspected toxic exposures are often evaluated with this battery of tests, and patterns of abnormalities (such as disproportionately high GGT or bilirubin) can raise suspicion for toxicant-associated steatohepatitis (TASH) or other exposure-related liver injuries [36,37]. These laboratory biomarkers are, therefore, critical endpoints for epidemiologic studies examining environmental determinants of liver health.

Real-world exposure does not occur to single chemicals in isolation, and rather, populations are concurrently exposed to complex mixtures of multiple pollutants. There is growing concern that combined exposures to toxicants may have additive or synergistic effects on liver disease risk beyond the effects of individual agents. Recent research in environmental health has started to address these mixture effects. For example, one study demonstrated that combined exposure to a suite of metals, PCBs, dioxins, and furans was associated with cardiovascular dysfunction, suggesting that co-exposures can jointly influence disease processes [38]. In the context of liver health, chronic low-level exposure to a mixture of hepatotoxic agents could plausibly amplify oxidative stress or inflammatory pathways, thereby exacerbating liver injury [39]. Traditional risk assessments that evaluate one chemical at a time may therefore underestimate the true health risks present in communities facing multiple simultaneous exposures. Indeed, epidemiologic analyses have found that even when individual metals like Pb, Cd, or Hg show modest associations with liver injury, their combined presence might produce a stronger effect or affect a broader range of liver function indicators [21]. This rationale underpins the need for studies examining cumulative exposures by understanding how mixed chemical burdens contribute to liver dysfunction, which is crucial for informing public health interventions and regulatory policies aimed at reducing toxic exposures.

Considering these concerns, this study was designed to investigate the effects of combined environmental chemical exposures on biomarkers of liver function in humans. We utilized data from a nationally representative U.S. survey (NHANES 2003–2004) to assess associations between multiple concurrent exposures, including heavy metals (Cd, Pb, and Hg), POPs (PCBs, dioxins, and furans), and a panel of liver function test outcomes. We hypothesized that higher levels of these chemicals, individually and in mixtures, would be associated with evidence of liver injury or dysfunction (e.g., elevated liver enzymes and altered protein levels). By leveraging advanced mixture modeling approaches alongside traditional regression, our goal was to elucidate the cumulative impact of co-exposures on hepatic health in the general population.

## 2. Materials and Methods

### 2.1. Study Design and Population

We analyzed existing data from the 2003–2004 cycle of the National Health and Nutrition Examination Survey (NHANES). NHANES is a continuous, cross-sectional survey administered by the U.S. Centers for Disease Control and Prevention (CDC) that uses a multistage, stratified probability sampling design to generate a representative sample of the civilian, non-institutionalized U.S. population [40]. The 2003–2004 NHANES cycle included extensive health interviews, physical examinations, and laboratory testing for a variety of biomarkers [40]. All participants provided written informed consent, and the survey protocol was approved by the CDC/National Center for Health Statistics Institutional Review Board. Detailed descriptions of the NHANES study design and laboratory methods are publicly available [40].

For our analysis, we defined an analytical cohort of adult participants from NHANES 2003–2004 with complete data on relevant exposures, outcomes, and covariates. We included individuals aged 18 years and older who had laboratory measurements for all targeted environmental chemicals (see “Exposure Assessment” below) as well as results for all selected liver function tests (see “Outcome Assessment” below). Among the 10,122 persons examined in NHANES 2003–2004 (which includes all ages), 947 adults met these criteria and were retained as the final study sample. We excluded participants with any missing biomarker data or key demographic information. The resulting sample of 947 represents those with complete cases across all measures and encompasses the range of demographics of the broader NHANES adult population.

### 2.2. Exposure Assessment

The exposures of interest in this study were blood’s heavy metals and serum POPs. Specifically, NHANES measured whole blood concentrations of three toxic metals: cadmium (Cd), lead (Pb), and total mercury (Hg), and serum concentrations of multiple organochlorine compounds, including a suite of PCB congeners, dioxins, and furans. In the 2003–2004 cycle, 12 PCB congeners, 7 dioxin congeners, and 10 furan congeners were quantified in serum (see Appendix A for the list of specific analytes). All laboratory analyses were performed by the Division of Environmental Health Laboratory Sciences at CDC using state-of-the-art detection methods. Blood metal levels were determined by inductively coupled plasma mass spectrometry (ICP-MS), a highly sensitive technique for trace metal analysis [40]. Serum POP concentrations were measured using high-resolution gas chromatography coupled with high-resolution mass spectrometry (HRGC/HRMS), which is the gold-standard method for detecting dioxins, furans, and PCBs at ultra-trace levels. Concentrations of lipophilic contaminants (PCBs, dioxins, and furans) were reported on a lipid-adjusted basis (e.g., per gram of serum total lipid) to account for their tendency to partition into fat [41]. Quality control procedures and calibration in NHANES ensured the accuracy and comparability of these measurements [40]. Environmental exposure biomarkers were selected for this study based on toxicological relevance (i.e., the known or suspected hepatotoxic potential) and a high frequency of detection in the NHANES sample, to reduce the influence of non-detectable values on analyses.

### 2.3. Outcome Assessment

The outcome variables in this study were a panel of serum liver function test results, chosen to capture multiple domains of hepatic health. These included alanine aminotransferase (ALT), aspartate aminotransferase (AST), gamma-glutamyl transferase (GGT), alkaline phosphatase (ALP), lactate dehydrogenase (LDH), total bilirubin (TB), albumin, and total protein (TP). All eight biomarkers were measured in serum by standardized clinical chemistry assays as part of NHANES laboratory protocols [40]. The selection of these particular markers was guided by their widespread use in diagnosing or monitoring liver conditions and their relevance to chemical hepatotoxicity [33,34]. ALT and AST primarily reflect hepatocellular injury (elevations indicate leakage of these enzymes from damaged hepatocytes). GGT and ALP are indicative of cholestatic injury or enzyme induction (with GGT also serving as a marker of exposure to enzyme-inducing xenobiotics like certain POPs). Albumin and total protein assess the liver’s synthetic function and nutritional status (low albumin suggests impaired protein synthesis in chronic liver disease) [31,32,34]. All outcome variables were treated as continuous measures (in their original units: U/L for enzymes, mg/dL for bilirubin, g/dL for proteins) in our statistical analyses. To satisfy model assumptions, we evaluated distributions of each biomarker and applied logarithmic transformations where necessary (for example, right-skewed variables such as ALT and GGT were log-transformed in regression models to normalize their residuals). Clinical reference ranges and thresholds were not explicitly used to define outcomes in this analysis; instead, we analyzed the biomarkers across their continuous ranges to maximize statistical power and to detect associations even within the clinically “normal” range of liver function tests.

This study assessed liver biomarker variation rather than liver disease incidence. Because NHANES is cross-sectional, temporal sequence and incident liver disease cannot be established. The findings, therefore, support hypothesis generation regarding mixture-related hepatic or systemic biological burden, but they do not establish current disease incidence or causal risk.

### 2.4. Toxic Equivalency (TEQ) Calculation

Due to dioxins, furans, and certain PCB congeners sharing a common mechanism of AhR-mediated toxicity, we calculated toxic equivalency quantities TEQ to summarize the combined potency of these dioxin-like chemicals. We applied the World Health Organization (WHO) Toxic Equivalency Factor (TEF) scheme (2005 revision) to each congener to compute a total toxic equivalent concentration [41]. In brief, each dioxin, furan, or dioxin-like PCB congener was assigned a TEF value relative to the toxicity of the reference compound 2,3,7,8-TCDD (which has a TEF of one). See Appendix B for the assigned TEF for each dioxin, furan, and PCB congener. The TEQ for each participant was then calculated as follows:

$$Total\;TEQ\;\left(pg/g\;lipid\right)=\;\sum_{i=1}^{n}\left(C_{i}\;\times \;{TEF}_{i}\right)$$

where $C_{i}$ is the lipid-adjusted concentration of congener i in the participant’s serum, and ${TEF}_{i}$ is the TEF for that congener. This summation yields a single composite exposure metric (in picograms (pg) of TCDD equivalents per gram g of lipid (pg/g), which reflects the overall dioxin-like toxic burden for each individual. TEQ values were used in analyses to represent the joint exposure to dioxins, furans, and dioxin-like PCBs, while non-dioxin-like PCB exposures and heavy metals were analyzed separately (since they do not have TEF values).

### 2.5. Covariates

We adjusted for a set of covariates to control for potential confounding factors based on prior knowledge of liver disease epidemiology and exposure patterns. Demographic and lifestyle covariates included age (years, continuous), sex (male or female), race/ethnicity (categorized as Non-Hispanic White, Non-Hispanic Black, Mexican American, or Other/Multi-racial) [40,42], body mass index (BMI, treated as continuous), smoking status (current smoker, former smoker, or never smoker), alcohol consumption (categorized by self-reported alcohol use, e.g., heavy drinker, moderate, or nondrinker, based on NHANES dietary recall and questionnaire data), and household income (annual family income, adjusted for family size, as a proxy for socioeconomic status) [40]. These covariates were included in multivariable models because they are known to be associated with liver function or disease risk and might also be associated with exposure levels, thereby confounding the exposure-outcome relationships if not accounted for [21]. For example, age and sex influence baseline liver enzyme levels and the pharmacokinetics of toxicants; BMI is strongly related to liver fat accumulation (which can affect liver enzymes) and may also correlate with pollutant burdens stored in adipose tissue; and smoking and alcohol use are independent causes of liver enzyme elevation and could modify the effects of chemical exposures. By including these variables as covariates, we aimed to isolate the associations of environmental chemicals with liver function biomarkers as much as possible from these other influences. In secondary analyses, we also considered additional adjustments or stratifications (such as accounting for hepatitis viral infection status or diabetes) if relevant data were available, to ensure that our findings were robust to other liver disease risk factors. All covariates were obtained from the NHANES interview and examination files corresponding to the 2003–2004 cycle [40,42].

### 2.6. Statistical Analysis

We implemented a comprehensive statistical analysis plan to evaluate both individual and mixture effects of the exposures on liver function outcomes. Statistical significance was assessed at a two-tailed alpha level of 0.05. Analyses were conducted using R version 4.5.1 (R Foundation for Statistical Computing, Vienna, Austria) and Stata/SE version 19.5 (StataCorp LLC, College Station, TX, USA), with survey procedures and specialized packages for mixture analysis. The analytical approach consisted of the following steps and methods.

### 2.7. Descriptive Statistics

We first examined the distribution of each exposure and outcome variable. Continuous variables were summarized using means, medians, standard deviations, and interquartile ranges, while categorical variables were summarized using frequencies and percentages. Because liver biomarkers were analyzed as continuous outcomes, we described their distributions overall and across exposure strata rather than classifying participants according to clinical thresholds. We also compared characteristics of the study population across exposure levels using stratified descriptive tables. Pairwise correlations among the chemical exposures were evaluated using Spearman correlation coefficients to detect collinearity or clustering of exposures (for instance, the correlation between lead and cadmium). These descriptive analyses provided context on typical exposure levels and helped inform the modeling strategy (for example, highly correlated pollutants might require special handling in multipollutant models).

### 2.8. Multi-Pollutant Regression Models

Estimating the associations between environmental chemicals and liver function biomarkers, we fitted unweighted multivariable linear regression models for each liver outcome, with all exposure variables entered simultaneously and adjustment for age, sex, race/ethnicity, BMI, smoking, alcohol use, and income. Right-skewed liver biomarkers were log-transformed before analysis, and coefficients from these models were back-transformed and interpreted as a percent change in the outcome per interquartile range increase in exposure. Outcomes that were not log-transformed were interpreted as beta coefficients on the original scale. To facilitate comparison across chemicals with different measurement scales, exposures were modeled per interquartile range increase. We report regression estimates, 95% confidence intervals, and *p*-values from these models [38].

### 2.9. Weighted Quantile Sum (WQS) Regression

We applied WQS regression as a mixture modeling approach to assess the overall effect of the pollutant mixture on liver outcomes and to identify the constituents contributing most to any observed effect [43]. WQS is a technique that creates an index of combined exposure by assigning weights to each component based on its contribution to the outcome. In our WQS analysis, all exposures (e.g., the three metals and PCBs, dioxins, furans, and their TEQ) were included simultaneously. Each exposure was categorized into quartiles, and a weighted index (ranging from zero to one) was constructed as a linear combination of these quartile indicators. We focused on a positive direction for the association (under the assumption that higher exposure levels would not be beneficial for liver health). The WQS model was fit using bootstrap sampling to ensure stability of the weight estimates. The resulting WQS index coefficient represents the change in the liver outcome per one quantile increase in the overall mixture [43,44]. A significant positive WQS index coefficient would suggest that the mixture as a whole is associated with higher (worse) levels of the liver biomarker [45]. We also examined the weights attributed to each chemical in the final WQS solution to determine which pollutants were the dominant drivers of the mixture effect. For instance, WQS might reveal that cadmium and PCB-153 carry the largest weights in predicting ALT, highlighting them as particularly influential within the mixture [42,46]. All WQS models were adjusted for the same covariates as the single-pollutant models. WQS performs well for estimating the overall directional effect of correlated exposure mixtures and identifying influential contributors within the mixture. Unlike WQS, however, BKMR can accommodate nonlinear exposure-response relationships and potential interactions among mixture components [47].

### 2.10. Quantile G-Computation

Moreso, we implemented quantile g-computation as an additional mixture analysis method. Quantile g-computation (often abbreviated as “qgcomp”) is an extension of g-computation that estimates the joint effect of increasing all exposures in the mixture by one quantile on the outcome, under an additive dose-response assumption [48]. Unlike WQS, which focuses on a weighted index, qg-comp fits a multivariable model, including all exposures, and then calculates an overall mixture effect parameter. We specified a qg-comp model, where each exposure was divided into quantiles (quartiles), and the increase from one quantile to the next for all exposures together was the exposure contrast of interest. The method produces an estimate (and confidence interval) for the mixture effect and essentially the expected change in the outcome if all exposures were simultaneously increased from a lower quantile to a higher quantile. It also provides component-wise effect estimates analogous to regression coefficients, which can be interpreted similarly to weights indicating the relative contribution of each exposure to the overall mixture effect [49]. We ran the g-computation procedure using a regression model that included all exposures and the same covariates, and we derived the mixture effect estimate using Monte Carlo simulation as implemented in available software [50]. This approach has the advantage of being able to handle negatively correlated exposures and effect directions (by allowing positive or negative partial effects for each component), in contrast to the non-negativity constraint in WQS.

### 2.11. Bayesian Kernel Machine Regression (BKMR)

Finally, to explore potential nonlinear and interactive effects among the mixture components, we utilized BKMR. A flexible semi-parametric modeling approach that treats the exposure-response function as unknown and estimates it using kernel methods, while simultaneously allowing for high-dimensional exposure inputs. In practice, we fit BKMR models with all measured chemicals entered as predictors and each liver outcome as the response, adjusting for covariates. The BKMR model does not assume a linear relationship; instead, it learns the joint effect of the exposure vector. We ran the BKMR model in a Bayesian Markov Chain Monte Carlo (MCMC) framework, drawing a large number of posterior samples to characterize the uncertainty in the estimated exposure-response function [47]. From the BKMR results, we derived several quantities of interest: (a) the overall effect of the mixture (estimated by comparing the predicted outcome when all exposures are at their 75th percentile vs. all at their 25th percentile, with credible intervals), (b) single-exposure response functions (while other exposures are held at median levels), which allowed us to visualize how each chemical’s association with the outcome might be non-linear, and (c) two-exposure interaction functions to see if any pair of chemicals showed evidence of interaction (i.e., a departure from additivity) [47]. For example, BKMR can reveal if the effect of mercury on ALT is amplified at high levels of co-exposure to PCBs. We assessed convergence diagnostics for the BKMR MCMC chains and ensured adequate mixing. BKMR results complement the WQS findings by providing insight into complex dose-response shapes and interactions that WQS (which assumes linearity and no interactions) might miss.

## 3. Results

### 3.1. Descriptive Analysis of Covariate Variables

Table 1 indicates that the male and female groups were broadly comparable in age, BMI, income distribution, and ethnicity, suggesting limited demographic imbalance by gender. The clearest gender differences were observed in behavioral covariates, where males had a higher prevalence of smoking and alcohol use, whereas females were more likely to report not smoking and not drinking. These patterns suggest that lifestyle-related factors, rather than basic demographic characteristics, may contribute more strongly to gender-related differences in subsequent liver biomarker analyses.

### 3.2. Summary Statistics of Outcome and Exposure Variables

As shown in Table 2, the exposure profile showed clear gender-specific differences across several contaminants. Among metals, lead concentrations were significantly higher in males than in females, whereas cadmium and mercury did not differ meaningfully by gender. For PCBs, females had significantly higher mean levels of PCB66, PCB74, PCB105, PCB118, and PCB126, while males had higher mean values in the other PCB chemicals (PCB156, PCB157, and PCB169; PCB28, PCB167, PCB189, and PCB81), which were similar across gender groups. A similar pattern was observed for dioxins, where females had higher levels of 1,2,3,4,6,7,8-Heptachlorinated dioxin (1,2,3,4,6,7,8-HpCDD), 1,2,3,4,6,7,8,9-Octachlorinated dioxin (1,2,3,4,6,7,8,9-OCDD), and 2,3,7,8-Tetrachlorinated dioxin (2,3,7,8-TCDD), while the remaining dioxins showed no significant gender difference. In contrast, several furans were higher in males, including 2,3,7,8-Tetrachloro dibenzofuran (2,3,7,8-TCDF), 1,2,3,7,8-Pentachlorinated dibenzofuran (1,2,3,7,8-PeCDF), 1,2,3,7,8,9-Hexachlorinated dibenzofuran (1,2,3,7,8,9-HxCDF), 2,3,4,6,7,8-Hexachlorinated dibenzofuran (2,3,4,6,7,8-HxCDF), and 1,2,3,4,6,7,8-Heptachlorinated dibenzofuran (1,2,3,4,6,7,8-HpCDF), whereas (2,3,4,7,8-Pentachlorinated dibenzofuran (2,3,4,7,8-PeCDF), 1,2,3,4,7,8-Hexachlorinated dibenzofuran (1,2,3,4,7,8-HxCDF), 1,2,3,6,7,8-Hexachlorinated dibenzofuran (1,2,3,6,7,8-HxCDF), 1,2,3,4,7,8,9-Heptachlorinated dibenzofuran (1,2,3,4,7,8,9-HpCDF), and 1,2,3,4,6,7,8,9-Octachlorinated dibenzofuran (1,2,3,4,6,7,8,9-OCDF)) other furan chemicals were comparable between groups.

Overall, these findings suggest that gender-related differences in exposure were more pronounced for specific PCB, dioxin, and furan congeners than for metals, indicating potentially distinct exposure sources, toxicokinetic patterns, or body-burden distributions between males and females.

### 3.3. Spearman’s Correlation Analysis

**Figure 1:**
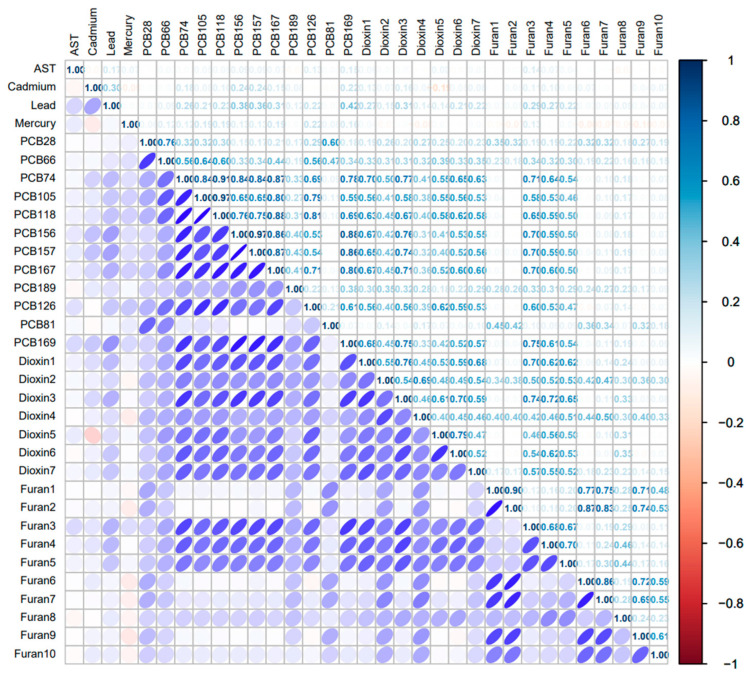
Spearman Correlation Analysis of all exposures with AST.

The Spearman correlation analysis in Figure 1 shows clear clustering among the exposure variables, with the strongest correlations observed within the POP classes. Metals were only weakly correlated overall, where cadmium and lead showed a modest positive correlation, while mercury was essentially uncorrelated with lead and slightly inversely correlated with cadmium. In contrast, several PCB congeners were strongly intercorrelated, particularly PCB156 with PCB157 and PCB105 with PCB118, indicating substantial collinearity within the PCB mixture. Dioxins also showed moderate-to-strong positive correlations, especially between 1,2,3,4,6,7,8-HpCDD and 1,2,3,4,6,7,8,9-OCDD, while furans displayed similar within-class clustering, with especially strong correlations between 2,3,7,8-TCDF and 1,2,3,7,8-PeCDF and between 1,2,3,7,8,9-HxCDF and 2,3,4,6,7,8-HxCDF.

Cross-class correlations were also evident, particularly between PCBs and dioxins and between dioxins and furans, suggesting shared sources, common exposure pathways, or similar toxicokinetic behavior. For example, 1,2,3,6,7,8-HxCDD showed strong positive correlations with several PCBs and furans. Overall, the correlation pattern indicates that POP exposures tend to occur as correlated mixtures, whereas the metals were comparatively independent. These findings support the use of mixture-based methods, such as WQS or BKMR, and suggest that collinearity should be considered when interpreting single-pollutant estimates.

### 3.4. Linear Regression Analysis

As shown in Table 3, in the unweighted multi-exposure linear regression models, after simultaneous adjustment for co-exposures and covariates, most chemicals were not independently associated with liver biomarkers. This was indicated by confidence intervals that included the null value. This pattern suggests attenuation after mutual adjustment, likely due in part to collinearity within the exposure mixture. Nonetheless, several associations remained. For albumin, mercury, PCB118, and 2,3,7,8-TCDD were positively associated, whereas PCB105 and 1,2,3,6,7,8-HxCDD were inversely associated. For ALP, only lead showed a clear positive association, while total protein was largely null.

More distinct signals were observed for hepatocellular and cholestatic markers. ALT, mercury, PCB118, and 2,3,4,7,8-PeCDF were positively associated, whereas PCB167 and 1,2,3,6,7,8-HxCDD were inversely associated. For AST, 2,3,4,7,8-PeCDF was positively associated, while PCB167, 1,2,3,6,7,8-HxCDD, and 2,3,4,6,7,8-HxCDF were inversely associated. For GGT, cadmium and 2,3,4,7,8-PeCDF were positively associated, whereas PCB74 was inversely associated. For LDH, lead and PCB189 were positively associated, while cadmium was inversely associated. For total bilirubin, lead and OCDD were positively associated, whereas 1,2,3,6,7,8-HxCDD and HpCDF were inversely associated. Overall, only a limited subset of metals and POP congeners showed consistent associations across outcomes, with lead, mercury, cadmium, PCB118, PCB167, HxCDD, and 2,3,4,7,8-PeCDF showing the most consistent signals. These estimates reflect effects per interquartile range increase in exposure, and for log-transformed outcomes, they represent percent change in the biomarker.

### 3.5. Weighted Quantile Sum (WQS) Regression

**Figure 2:**
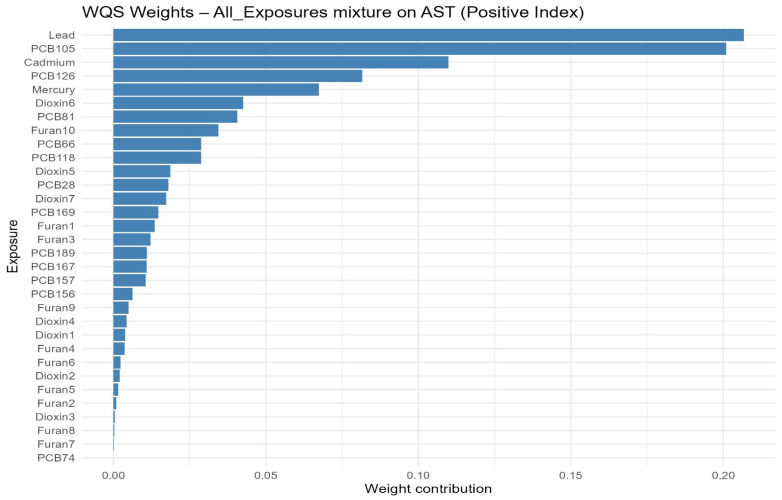
Weighted Quantile Sum (WQS) component weights for the positive association index of the all-exposure mixture with AST. This figure estimates an overall mixture index and assigns weights to individual chemicals according to their relative contribution to the mixture association. Higher weights indicate greater contribution within the fitted WQS model but should not be interpreted as definitive evidence of causality. Overall, the figure highlights the chemicals that contributed most strongly to the estimated mixture-liver biomarker association, helping identify pollutants that may be important drivers of the observed pattern.

**Figure 3:**
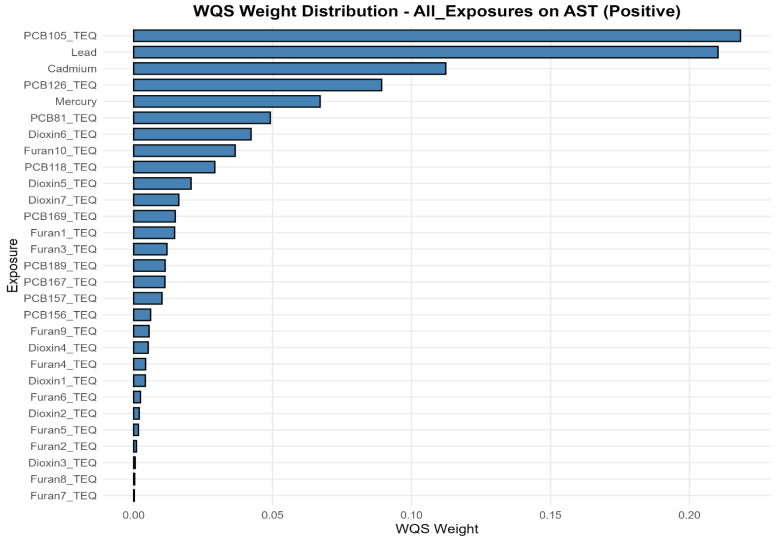
Weighted Quantile Sum (WQS) component weights for the positive association index of the TEQ-based all-exposure mixture with AST. This figure estimates an overall mixture index and assigns weights to individual chemicals according to their relative contribution to the mixture association. Higher weights indicate greater contribution within the fitted WQS model but should not be interpreted as definitive evidence of causality. Overall, the figure highlights the chemicals that contributed most strongly to the estimated mixture-liver biomarker association, helping identify pollutants that may be important drivers of the observed pattern.

In the positive-direction WQS models, the weight distributions were concentrated in a relatively small subset of chemicals, indicating that the observed mixture signals for liver biomarkers were driven by a limited number of dominant contributors rather than by uniform contributions across the full exposure panel. This is consistent with the intended role of WQS regression, which estimates a joint mixture effect and identifies the components that most strongly characterize the weighted index under correlation, rather than isolating independent causal effects for each analyte. In the primary concentration-based models, mercury and lead were recurrent major contributors, together with selected dioxin-like compounds and PCBs. Albumin was driven mainly by mercury, 1,2,3,4,6,7,8-HpCDD, 1,2,3,4,6,7,8-HpCDF, PCB126, and lead. ALP was overwhelmingly dominated by lead, with smaller secondary contributions from 1,2,3,4,7,8,9-HpCDF and PCB81. ALT and AST were characterized principally by lead, PCB105, PCB126, mercury, and cadmium. GGT showed a broader profile led by mercury with additional contributions from 1,2,3,4,7,8-Hexachlorinated dioxin (1,2,3,4,7,8-HxCDD), PCB126, PCB105, cadmium, and several furans. LDH was dominated by lead and mercury. Total bilirubin was driven chiefly by 1,2,3,4,6,7,8-HpCDD and PCB81, with mercury also contributing. Total protein was led by PCB126, mercury, and lead. The results are found in Figure 2; Figures S66–S73. The TEQ-based models showed strong concordance with the original analyses, with the same core drivers generally retained after potency-weighting of dioxin-like PCBs, dioxins, and furans (Figure 3; Figures S74–S81). Notably, PCB105_TEQ and PCB126_TEQ remained prominent for aminotransferases, while 1,2,3,4,6,7,8-HpCDD_TEQ and PCB81_TEQ continued to dominate total bilirubin. Because TEQ transformation is designed to summarize the relative toxic potency of dioxin-like congeners on a common scale, this concordance suggests that the mixture structure was not merely a concentration-ranking artifact but was also consistent with toxicologic potency.

### 3.6. Quantile G-Computation

**Figure 4:**
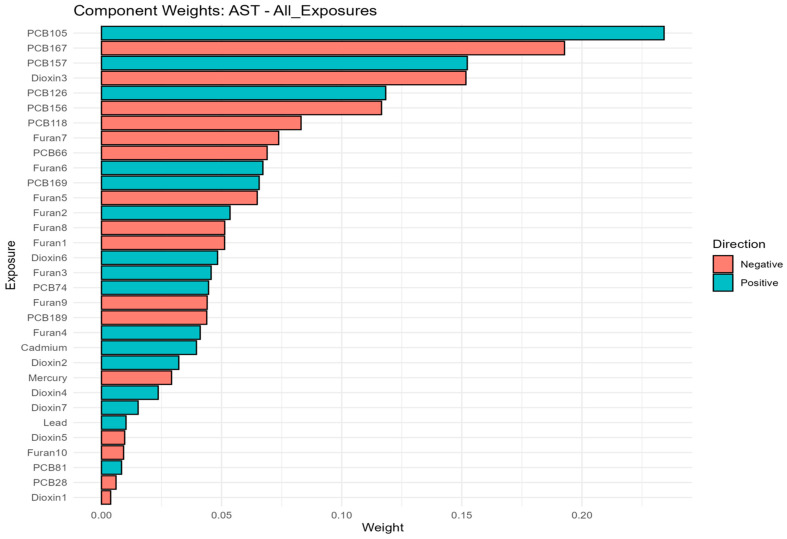
Quantile g-computation component weights for all exposures, concentration-based mixture in relation to AST. This figure evaluates the joint association of the chemical mixture with liver biomarker AST. qgcomp estimates the expected change in the AST associated with a simultaneous one-quantile increase in all mixture components, while allowing chemicals to contribute to positive or negative directions. The displayed weights represent the relative contribution of each pollutant to the overall mixture estimate. Higher values of weight indicate greater relative contribution within the fitted model, but should not be interpreted as definitive evidence of causality. Overall, Figure 4 illustrates whether the mixture association is driven by pollutants acting in similar or opposing directions.

In linear qgcomp models, total bilirubin was the only biomarker with a consistent positive overall mixture effect across all exposure parameterizations. A one-quartile simultaneous increase in the full mixture was associated with higher total bilirubin (ψ = 1.08, 95% CI: 0.26, 1.90; *p* = 0.010), and comparable associations were observed in the TEQ-congener (ψ = 0.93, 95% CI: 0.23, 1.64; *p* = 0.0098) and TEQ-plus-metals (ψ = 1.07, 95% CI: 0.27, 1.86; *p* = 0.0085) models. All other biomarkers showed null overall mixture effects, with generally positive but imprecise estimates for albumin, ALT, AST, GGT, LDH, and total protein, while ALP and LDH in the TEQ-congener models were directionally negative but nonsignificant. Nonlinear qgcomp models showed little evidence of nonlinearity, *psi*1 estimates were similar to the linear results, and *psi*2 was not significant for any outcome, although AST in the all-exposure model showed borderline curvature (*p* = 0.063). Overall, these findings indicate that the qgcomp mixture was robustly associated only with total bilirubin, and that the observed mixture effects were largely linear in form (Figure 4; Figures S82–S89).

#### Component-Weight Patterns

**Figure 5:**
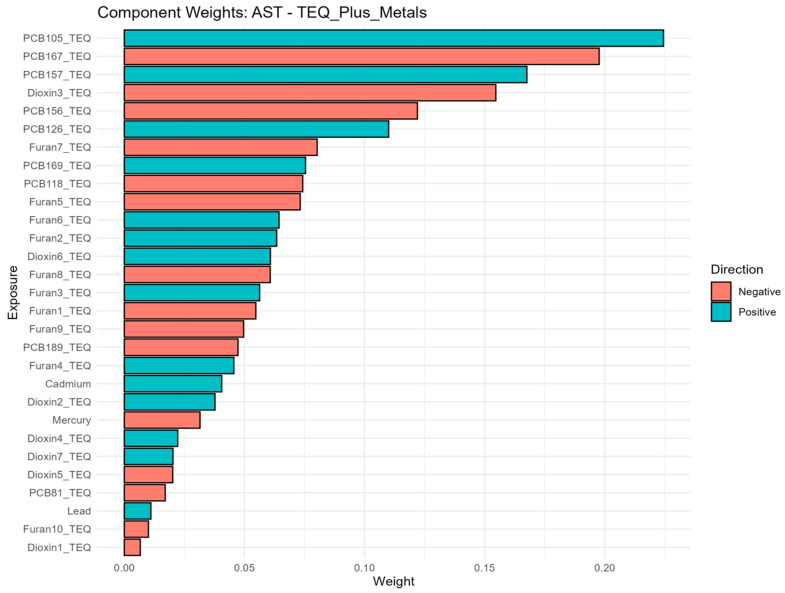
Quantile g-computation component weights for the TEQ-plus-metals mixture in relation to AST. This figure evaluates the joint association of the chemical mixture with liver biomarker AST. qgcomp estimates the expected change in the AST associated with a simultaneous one-quantile increase in all mixture components, while allowing chemicals to contribute to positive or negative directions. The displayed weights represent the relative contribution of each pollutant to the overall mixture estimate. Higher values of weight indicate greater relative contribution within the fitted model, but should not be interpreted as definitive evidence of causality. Overall, Figure 5 illustrates whether the mixture association is driven by pollutants acting in similar or opposing directions.

The component-weight decomposition showed pronounced bidirectionality across most biomarkers, indicating that null overall ψ estimates often reflected offsetting positive and negative sub mixtures rather than the absence of mixture structure. For albumin, positive weights were concentrated in PCB126, Furan3, 1,2,3,4,6,7,8-HpCDD, and 2,3,7,8-TCDD, whereas negative weights were driven by PCB169, PCB66, PCB157, and 1,2,3,7,8-PeCDD. ALP showed a positive cluster centered on PCB157 and lead, opposed by negative contributions from PCB156, PCB105, Furan3, and Furan1. For ALT and AST, positive weights were dominated by PCB105, PCB126, and PCB157, with smaller contributions from lead/cadmium and 1,2,3,7,8,9-HxCDF, while negative weights were consistently led by PCB167, 1,2,3,6,7,8-HxCDD, and PCB156. GGT showed positive contributions from PCB126, PCB156, and 1,2,3,7,8,9-HxCDF, with negative contributions from 1,2,3,6,7,8-HxCDD, Furan1, 1,2,3,4,6,7,8-HpCDF, and PCB81. LDH was characterized by positive weights for PCB157, PCB118, 1,2,3,4,7,8-HxCDD, and lead, opposed by negative weights for PCB156, 1,2,3,4,7,8,9-HpCDF, PCB167, and PCB105. For Total bilirubin, positive contributions from PCB157, PCB81, 1,2,3,4,6,7,8,9-OCDF, and PCB105 outweighed a negative cluster dominated by PCB156, 1,2,3,4,6,7,8-HpCDF, and 1,2,3,7,8-PeCDD, consistent with its significant positive net mixture effect. Total protein was driven positively by PCB126 and several dioxins/furans, while 1,2,3,4,7,8-HxCDD, 1,2,3,6,7,8-HxCDD, PCB66, and PCB169 contributed negatively. These patterns were largely preserved in the TEQ-congener and TEQ-plus-metals models (Figure 5; Figures S82–S89).

### 3.7. BKMR Analysis

BKMR was used to evaluate the joint and component-specific associations between a multi-chemical mixture (metals, PCB congeners, dioxins, and furans) and eight liver biomarkers. Across outcomes, patterns were generally characterized by (i) predominantly flat univariate exposure response functions for many mixture components, (ii) outcome-specific trends in the overall mixture effect, (iii) a limited number of exposures showing notable single-variable main effects, and (iv) minimal evidence of strong interaction structure, with wide credible intervals for a small subset of compounds.

#### 3.7.1. Posterior Inclusion Probability (PIP)

Across outcomes, the BKMR PIPs show that liver biomarkers are explained by a few dominant chemicals, not broad mixture contributions. Albumin is almost entirely driven by 1,2,3,6,7,8-HxCDD (conditional PIP = 0.92). ALP is dominated by metals, especially lead (conditional PIP = 0.92), with 2,3,4,7,8-PeCDF contributing secondarily (0.51). ALT is again metal-driven, led by mercury (conditional PIP = 0.94), with modest additional contributions from PCB126 (0.46) and 1,2,3,4,6,7,8-HpCDF (0.41). AST is overwhelmingly PCB-driven, essentially PCB28 alone (conditional PIP = 0.99). GGT shows no single driver; metals have moderate inclusion (0.30–0.36 each), but effects are diffuse across many low-PIP contributors. LDH is dominated by cadmium (condPIP = 0.98) and PCB189 (0.74), with smaller roles for 1,2,3,4,7,8-HxCDD and 1,2,3,6,7,8-HxCDD. Total bilirubin is dominated by PCBs, especially PCB81 (conditional PIP = 0.87), with 1,2,3,6,7,8-HxCDD also notable (=0.68). Total protein shows no meaningful chemical contributors (all PIPs = 0).

#### 3.7.2. Univariate Dose Response Relationship

**Figure 6:**
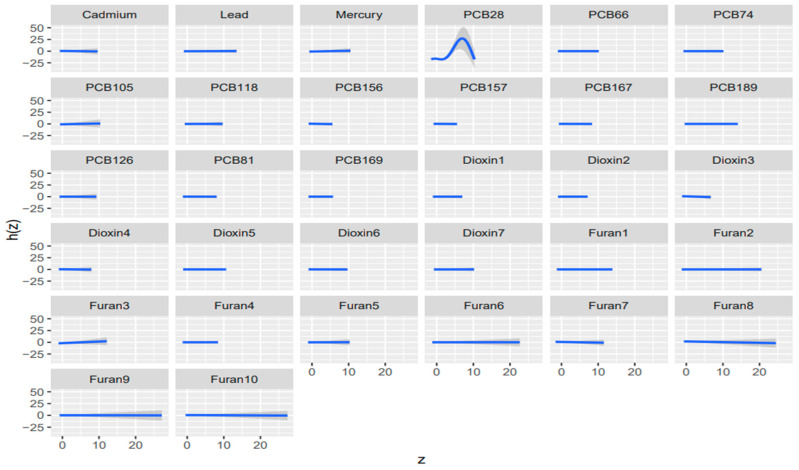
Univariate dose-response relationship for concentration-based exposures relating to AST, 95% credible intervals of exposures on liver biomarker AST. Adjusted for alcohol consumption, smoking status, age, ethnicity, income level, gender, and BMI. (Appendix A and Supplementary Materials for albumin, ALP, ALT, GGT, LDH, total bilirubin and total protein).

**Figure 7:**
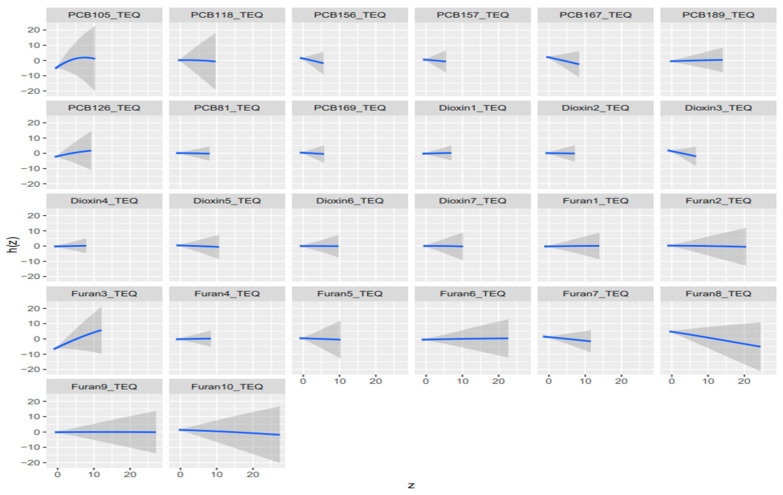
Univariate dose-response relationship for dioxin-like (TEQ-based) exposures and AST; 95% credible intervals of TEQ values for dioxin-like exposures on liver biomarker AST. Adjusted for alcohol consumption, smoking status, age, ethnicity, income level, gender, and BMI. (Appendix A and Supplementary Materials for albumin, ALP, ALT, GGT, LDH, total bilirubin, and total protein).

Univariate BKMR exposure response functions were largely flat in both the main-mixture and TEQ-weighted analyses, indicating limited evidence of strong independent effects for most components when co-exposures were fixed at the median. In the main-mixture models (Figure 6; Figures S2–S9), departures from the null were outcome-specific and driven by a few chemicals, and 1,2,3,6,7,8-HxCDD showed a clear inverse pattern with albumin and total bilirubin, lead exhibited a non-monotonic (inverted-U) relationship with ALP, mercury showed the strongest monotonic increase with ALT, PCB28 displayed a pronounced nonlinear peak for AST, cadmium was inversely related to LDH, and PCB189 showed a strong positive trend with LDH; GGT and total protein were largely null. In the TEQ models (Figure 7; Figures S34–S41), signals were more concentrated in dioxin-like potency metrics, and 1,2,3,6,7,8-HxCDD_TEQ consistently showed monotonic inverse associations with albumin and total bilirubin (and inversely trended with ALT and LDH), PCB189_TEQ increased LDH, 1,2,3,4,7,8-HxCDD_TEQ trended positively with LDH, and PCB81_TEQ/PCB126_TEQ exhibited inverted-U patterns for bilirubin; among furans, 1,2,3,4,6,7,8,9-OCDF_TEQ was inversely related to ALP, while AST showed opposing furan TEQ trends (2,3,4,7,8-PeCDF_TEQ positive; 1,2,3,4,6,7,8-HpCDF_TEQ negative). Overall, both approaches suggest mostly weak conditional relationships, but TEQ weighting sharpens and prioritizes dioxin-like drivers (especially 1,2,3,6,7,8-HxCDD_TEQ and select PCB TEQs), whereas the main-mixture analysis highlights additional non-TEQ drivers such as lead (ALP) and mercury (ALT).

#### 3.7.3. Overall Exposure Effect

**Figure 8:**
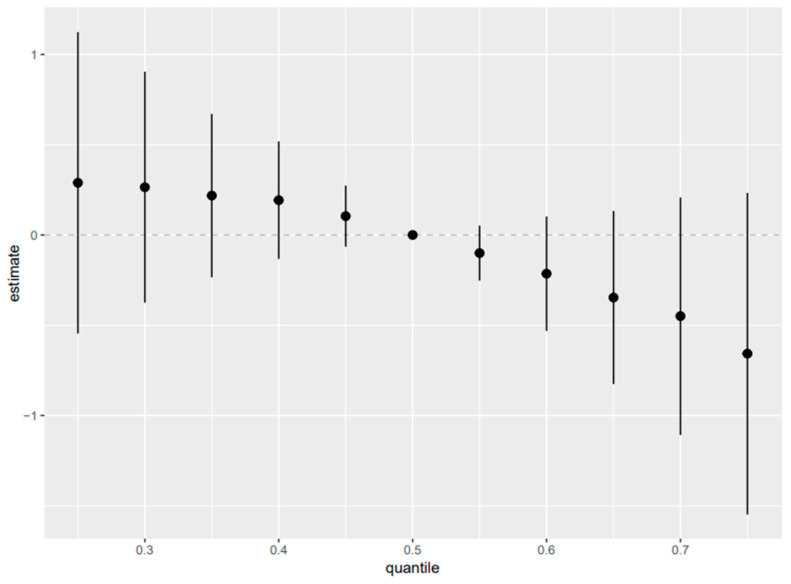
Overall exposure effect for concentration-based exposures relating to AST, 95% credible intervals of exposures on the liver biomarker AST. This figure shows the increasing quantile of combined exposures from the 25th to 75th quantile as compared to the 50th quantile. Adjusted for alcohol consumption, smoking status, age, ethnicity, income level, gender, and BMI (Appendix A and Supplementary Materials for albumin, ALP, ALT, GGT, LDH, total bilirubin, and total protein).

**Figure 9:**
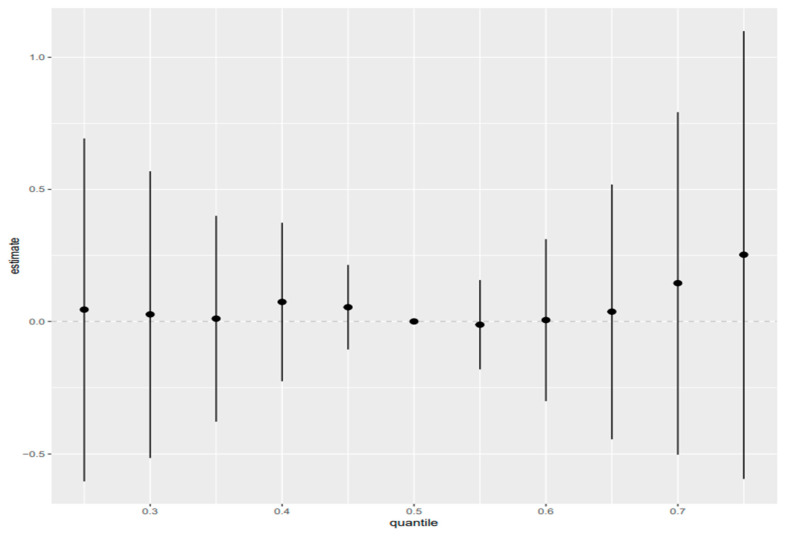
Overall exposure effect for dioxin-like (TEQ-based) exposures and AST; 95% credible intervals of TEQ values for dioxin-like exposures on liver biomarker AST. This figure shows the increasing quantile of combined exposures from the 25th to 75th quantile as compared to the 50th quantile. Adjusted for alcohol consumption, smoking status, age, ethnicity, income level, gender, and BMI. (Appendix A and SI for albumin, ALP, ALT, GGT, LDH, total bilirubin, and total protein).

Overall mixture effect is defined as contrasts in the estimated BKMR mixture function when all exposures were jointly set to specified quantiles relative to the median (50th percentile), demonstrated clear outcome-specific patterns and revealed important differences between the main-mixture and TEQ-weighted models. In the main-mixture analysis (Figure 8; Figures S10–S17), the overall exposure effect exhibited a monotonic inverse gradient for albumin, with estimates shifting from positive at lower joint-exposure quantiles to progressively negative at higher quantiles, consistent with lower albumin at higher cumulative exposure. ALP and total bilirubin showed monotonic positive gradients, indicating higher values at higher joint exposure levels. In contrast, ALT and AST demonstrated overall decreasing trends across increasing joint-exposure quantiles. GGT remained near-null across most quantiles with only minimal upward displacement at the upper tail, whereas LDH displayed a non-monotonic profile, characterized by modest positive effects at lower quantiles, a negative region at mid-to-upper quantiles, and a rebound at the highest quantile. Total protein showed no meaningful overall association. In the TEQ weighted analysis (Figure 9; Figures S42–S49), the overall mixture effects preserved the core patterns for albumin (monotonic decrease) and total bilirubin (monotonic increase) and similarly captured a non-monotonic LDH response. However, TEQ weighting shifted enzyme-related patterns. ALP and ALT showed inverse gradients (progressively decreasing with higher joint TEQ burden), and GGT demonstrated a clearer positive gradient across TEQ quantiles, while AST remained largely near-null with only a modest upward tendency at higher TEQ levels.

#### 3.7.4. Single-Variable Effects

**Figure 10:**
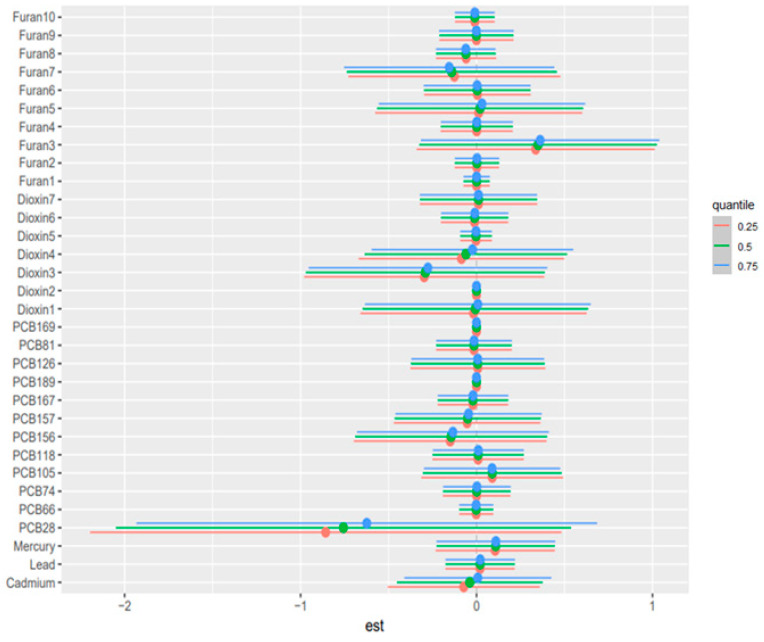
Single-variable effects for concentration-based exposures relating to AST, 95% credible intervals of exposures on liver biomarker AST. This figure defines the change in response associated with a change in a single exposure from its 25th to 75th quantile while all other exposures are fixed at a specific quantile (25th, 50th, or 75th). Adjusted for alcohol consumption, smoking status, age, ethnicity, income level, gender, and BMI. (Appendix A and Supplementary Materials for albumin, ALP, ALT, GGT, LDH, total bilirubin, and total protein).

**Figure 11:**
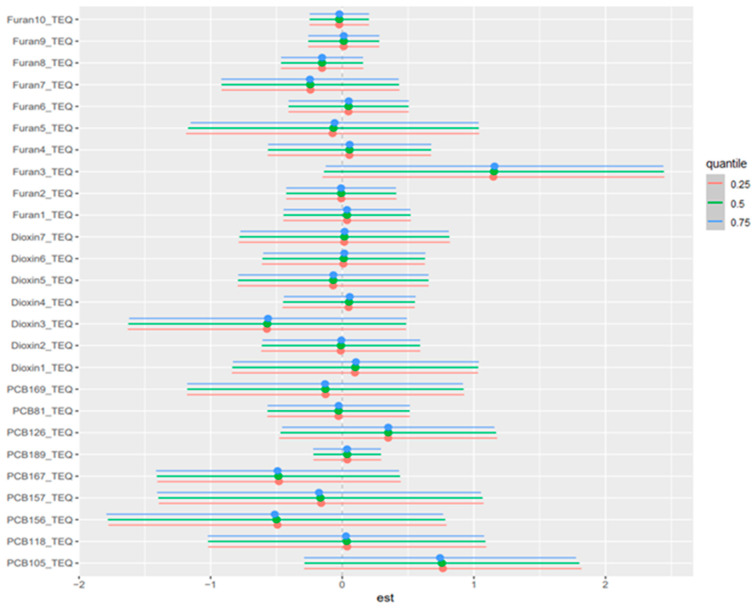
Single-variable effects for dioxin-like (TEQ-based) exposures and AST; 95% credible intervals of TEQ values for dioxin-like exposures on liver biomarker AST. This figure defines the change in response associated with a change in a single potency-weighted dioxin-like exposure from its 25th to 75th quantile while all other exposures are fixed at a specific quantile (25th, 50th, or 75th). Adjusted for alcohol consumption, smoking status, age, ethnicity, income level, gender, and BMI. (Appendix A and Supplementary Materials for albumin, ALP, ALT, GGT, LDH, total bilirubin, and total protein).

Single-variable risk summaries indicated that, in both the main-mixture and TEQ-weighted BKMR models, only a limited subset of mixture components exhibited discernible main effects. Across the different fixed mixture-background levels, the effect estimates were very similar, and their uncertainty intervals largely overlapped, indicating little evidence that the exposure to outcome association changes meaningfully with co-exposure levels. In the main-mixture analysis (Figure 10; Figures S18–S25), the clearest signals were outcome-specific and sparse. 1,2,3,6,7,8-HxCDD showed the largest inverse displacement for albumin; lead was the dominant positive contributor for ALP; PCB28 stood out for AST; PCB189 was the strongest positive contributor for LDH (with weaker, imprecise dioxin signals); and PCB81 showed the most pronounced positive association with total bilirubin, while GGT and total protein were largely null. The TEQ analysis (Figure 11; Figures S50–S57) preserved key drivers for albumin and total bilirubin with 1,2,3,6,7,8-HxCDD_TEQ as the primary inverse contributor for albumin (and also the strongest inverse for ALT) and PCB81_TEQ as the most stable positive predictor of total bilirubin, but shifted emphasis toward dioxin-like potency metrics and select TEQ components. ALP showed positive shifts for 1,2,3,4,7,8-HxCDD_TEQ and 1,2,3,6,7,8-HxCDD_TEQ alongside inverse tendencies for 2,3,4,7,8-PeCDF_TEQ and PCB169_TEQ. AST was most strongly characterized by a positive 2,3,4,7,8-PeCDF_TEQ signal; GGT showed large but imprecise positive estimates for PCB105_TEQ and PCB157_TEQ; and LDH reflected opposing TEQ contributions (notably 1,2,3,6,7,8-HxCDD_TEQ inverse vs. 1,2,3,4,7,8-HxCDD_TEQ and PCB189_TEQ positive). Overall, TEQ weighting largely retained the outcome-specific sparsity of single-variable effects but reallocated prominence toward dioxin-like potency and furan-related signals, whereas the main-mixture model highlighted metals and specific PCB congeners for several outcomes.

#### 3.7.5. Single-Variable Interaction Summaries

**Figure 12:**
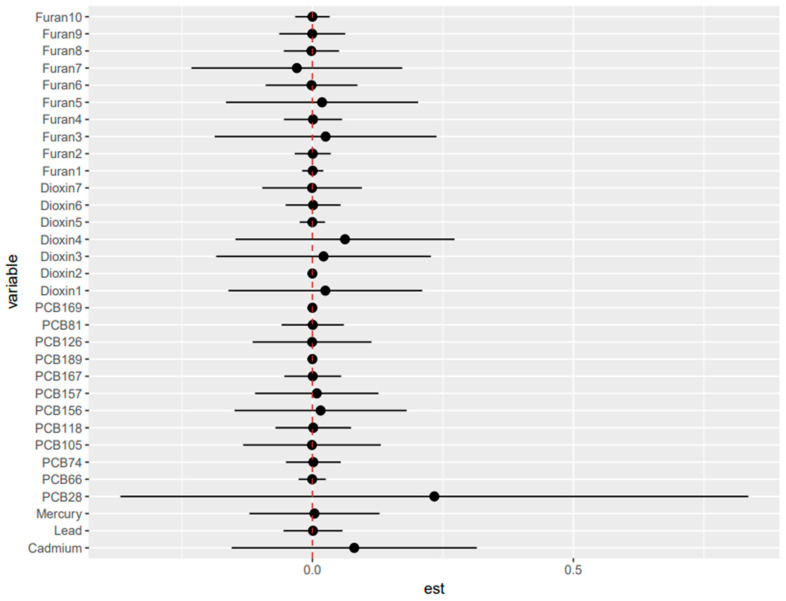
Single-variable interactions for concentration-based exposures relating to AST, 95% credible intervals of exposures on the liver biomarker AST. This figure compares the effect of each exposure when all other exposures are fixed at the 75th quantile compared to the 25th quantile. Adjusted for alcohol consumption, smoking status, age, ethnicity, income level, gender, and BMI. (Appendix A and Supplementary Materials for albumin, ALP, ALT, GGT, LDH, total bilirubin and total protein).

**Figure 13:**
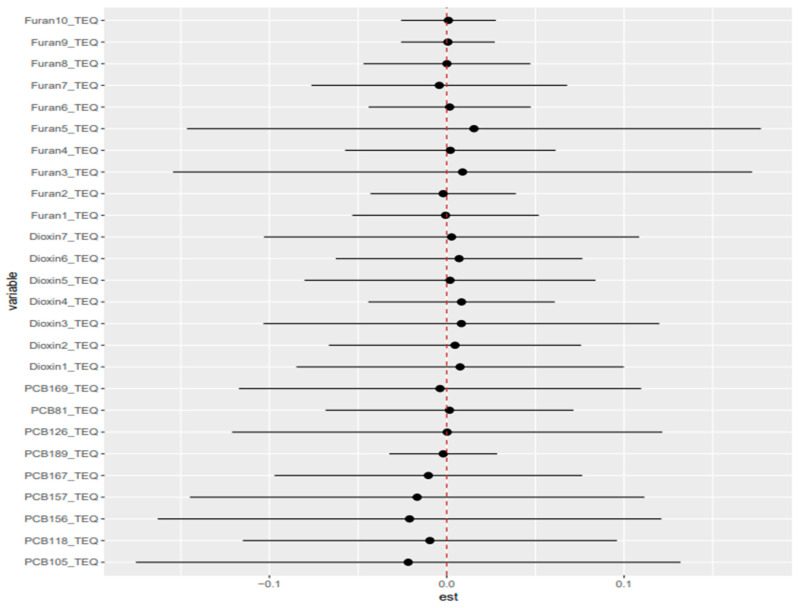
Single-variable interactions for dioxin-like (TEQ-based) exposures and AST; 95% credible intervals of TEQ values for dioxin-like exposures on liver biomarker AST. This figure compares the effect of each exposure when all other exposures are fixed at the 75th quantile compared to the 25th quantile. Adjusted for alcohol consumption, smoking status, age, ethnicity, income level, gender, and BMI. (Appendix A and Supplementary Materials for albumin, ALP, ALT, GGT, LDH, total bilirubin, and total protein).

Single-variable interaction summaries suggested minimal non-additivity in both the main-mixture and TEQ-weighted BKMR models. Across outcomes, the posterior mean interaction estimates were generally close to zero, indicating little evidence of strong interaction effects. In the main-mixture analysis (Figure 12; Figures S26–S33), the primary feature was localized imprecision (wider credible intervals) for a small subset of chemicals. Most notable were 1,2,3,7,8-Pentachlorodibenzo-p-dioxin (1,2,3,7,8-PeCDD) and PCB169 for Albumin, lead with PCB169 and 2,3,4,7,8-PeCDF (ALP), mercury, 1,2,3,6,7,8-HxCDD and PCB126 (ALT), PCB28 (AST), lead, PCB157 and 2,3,4,7,8-PeCDF (GGT), cadmium and select dioxins and PCB189 (LDH), lastly, PCB81 and 1,2,3,6,7,8-HxCDD (total bilirubin). Rather than consistent non-zero interaction effects, total protein remained uniformly null. The TEQ interaction summaries (Figure 13; Figures S58–S65) mirrored these conclusions, with interactions largely near null and uncertainty concentrated in a limited set of TEQ predictors, particularly 1,2,3,7,8-PeCDD_TEQ, 1,2,3,4,7,8-HxCDD_TEQ and 1,2,3,6,7,8-HxCDD_TEQ (several outcomes), 2,3,4,7,8-PeCDF_TEQ and PCB169_TEQ (ALP and AST), 1,2,3,4,6,7,8-HpCDD_TEQ and PCB105_TEQ (GGT), and PCB81_TEQ and 1,2,3,6,7,8-HxCDD_TEQ (total bilirubin). Overall, both frameworks indicate that mixture associations are driven primarily by main effects (and marginal nonlinearity), with no strong, stable interaction structure detected.

### 3.8. Integrated TEQ Findings Across Biomarkers

Across TEQ-based BKMR summaries, the joint mixture effect indicated inverse associations for albumin, ALP, and ALT, positive associations for total bilirubin (and weakly GGT), non-monotonic behavior for LDH, and largely null overall effects for AST and total protein. Component-specific results consistently highlighted a small set of influential TEQ predictors: 1,2,3,6,7,8-HxCDD_TEQ (dominant inverse contributor across albumin and several enzyme/cell-injury markers), PCB81_TEQ (dominant positive contributor for bilirubin), PCB189_TEQ and 1,2,3,4,7,8-HxCDD_TEQ (positive contributors for LDH), and 2,3,4,7,8-PeCDF_TEQ (strongest positive contributor for AST and the most recurrent interaction candidate for ALP and AST). Interaction summaries further suggested that TEQ associations were driven primarily by main effects and marginal nonlinearity, with limited evidence of strong non-additivity and uncertainty concentrated in a few dioxin- and PCB-related TEQ components.

## 4. Discussion

In this study, linear regression, WQS regression, qgcomp, BKMR, and TEQ potency weighted reparameterizations make different assumptions about correlation, functional form, and effect structure. The results were interpreted by considering the consistency and direction of findings across analytical methods rather than relying on a single association estimate. WQS estimates a single-index mixture effect under a directional constraint and identifies “index-defining” contributors in correlated mixtures [46]. qgcomp estimates the net effect of jointly increasing all exposures by one quantile without assuming directional homogeneity, and BKMR flexibly models nonlinear, potentially non-additive exposure–response surfaces while enabling hierarchical selection via group and conditional posterior inclusion probabilities (PIPs). The persistence of key signals under TEQ reparameterization further indicates that a portion of the mixture pattern is consistent with dioxin-like potency on the WHO TEF scale rather than being solely a concentration-ranking artifact [41].

### 4.1. Hepatic Allostatic Load Expressed by Pathway-Specific Biomarkers

Conceptually, the liver can be framed as carrying hepatic allostatic load of cumulative “wear-and-tear” from chronic demands for xenobiotic metabolism, oxidative defense, bile acid transport, and inflammatory regulation [48]. Importantly, hepatic allostatic load need not manifest as a uniform elevation of all exposures. Instead, it can produce divergent biomarker signatures depending on whether the dominant perturbation is hepatocellular injury or leakage (ALT/AST), cholestatic/biliary dysfunction (ALP, total bilirubin), glutathione-linked detoxification demand (GGT), generalized cellular stress (LDH), or hepatic synthetic/inflammatory status (albumin).

### 4.2. Total Bilirubin as the Most Reproducible Mixture Endpoint Across Methods

Across methods, total bilirubin emerged as the most robust mixture-associated biomarker. In qgcomp, bilirubin showed a statistically significant positive net mixture effect across all three mixture definitions (all exposures, TEQ congeners, TEQ and metals), indicating that a one-quantile joint increase in the mixture corresponded to higher bilirubin on the original scale (all *p*-values = 0.008–0.010 in the qgcomp outputs) [49]. This finding is reinforced by BKMR, where bilirubin demonstrated the clearest monotonic positive overall mixture gradient in both the main-mixture and TEQ-weighted models, and by PIP patterns that concentrated support within the PCB and dioxin-like subsets (notably PCB81 and 1,2,3,6,7,8-HxCDD). qgcomp further clarifies the mechanism by exposing a bidirectional mixture architecture, and bilirubin was driven by a positive-weight sub mixture centered on PCB157, PCB81, 1,2,3,4,6,7,8,9-OCDF, and PCB105, opposed by a negative sub mixture led by PCB156, 1,2,3,4,6,7,8-HpCDF, and 1,2,3,7,8-PeCDD.

The persistence of a positive net effect despite strong negative weights implies that the bilirubin-elevating sub-mixture is sufficiently dominant to outweigh countervailing components. This pattern is biologically plausible. Dioxin-like compounds and select PCBs can disrupt bile acid homeostasis and hepatobiliary transport pathways through AhR-mediated transcriptional programs [41,50,51]. Experimental evidence shows that potent AhR agonists such as TCDD can increase serum bilirubin and ALP in animal models [50], and mechanistic work demonstrates that AhR modulation affects cholestatic injury phenotypes and bile acid metabolism, including bilirubin-related outcomes [52,53]. Thus, the cross-method reproducibility for bilirubin supports an interpretation of subclinical hepatobiliary stress as a key manifestation of mixture-related hepatic allostatic load in this dataset.

### 4.3. Albumin and Synthetic/Inflammatory Signaling

Albumin showed consistent evidence of vulnerability in BKMR, with a monotonic inverse overall mixture gradient and a prominent inverse univariate trend for 1,2,3,6,7,8-HxCDD and 1,2,3,6,7,8-HxCDD_TEQ, while PIPs identified 1,2,3,6,7,8-HxCDD as the dominant contributor. This pattern is consistent with the notion that higher cumulative exposure, especially within dioxin-like components, may be associated with lower albumin, potentially reflecting subtle shifts in hepatic synthetic capacity and/or inflammation-linked downregulation rather than overt failure. qgcomp yielded a null-to-imprecise overall effect for albumin, but the structured weights indicate counterbalancing positive and negative sub-mixtures, again reinforcing that “null overall” can reflect internal cancellation rather than absence of mixture architecture [49]. This is a recurring theme across outcomes and underscores why triangulation across methods is informative.

### 4.4. Enzyme Biomarkers Bidirectionality, Nonlinearity, and Method-Dependent Estimands

For hepatocellular and cholestatic enzymes (ALT, AST, ALP, GGT), the combined evidence indicates a strong mixture structure but less consistent net directionality, an expected outcome when multiple mechanistic pathways operate in opposition.

#### 4.4.1. ALT and AST

WQS weights repeatedly emphasized metals (notably mercury and lead) and dioxin-like PCBs (PCB105 and PCB126) as major index contributors, whereas qgcomp estimated a largely net-null effect for aminotransferases, consistent with counterbalancing positive and negative sub mixtures. BKMR adds important details; it identified strong univariate nonlinearity for select drivers (e.g., mercury increasing ALT; PCB28 peak for AST) and concentrated PIPs on those representative contributors. This pattern aligns with prior NHANES evidence linking PCBs, lead, and mercury to ALT elevation as a marker of liver injury/NAFLD-related processes [54], but also underscores that mixture behavior in population data can be non-monotonic and bidirectional, which qgcomp and BKMR capture more directly than one-direction WQS.

#### 4.4.2. ALP

Main-mixture BKMR suggested increasing ALP with higher joint exposure quantiles, while TEQ-weighted BKMR suggested an inverse ALP gradient. This divergence is informative rather than contradictory. ALP in the full mixture may be driven by non-TEQ pathways, particularly metals (consistent with WQS and PIP patterns that strongly prioritized lead for ALP), whereas TEQ emphasizes dioxin-like potency and can shift the mixture axis toward AhR-related processes. The qgcomp results for ALP were net-null with broad uncertainty, consistent with counterbalancing sub-mixtures and/or limited precision.

#### 4.4.3. GGT

GGT showed diffuse contributor profiles (WQS “broader” weights; BKMR PIPs not dominated by a single driver) and net-null qgcomp effect. TEQ-weighted BKMR showed a clearer positive overall gradient, consistent with the concept that GGT reflects glutathione-linked detoxification demand and oxidative stress burden. GGT has been proposed as a biomarker of exposure to multiple pollutants, including lead, cadmium, and dioxin-like compounds, because it participates in glutathione conjugate metabolism [55]. Thus, the GGT pattern is consistent with a “stress-axis” biomarker that integrates across exposures without yielding one dominant driver.

### 4.5. LDH and Generalized Cellular Stress

LDH consistently behaved as a nonlinear, stress-axis endpoint rather than a single-pathway marker. BKMR overall effects were non-monotonic in both main-mixture and TEQ-weighted models, and PIPs concentrated on cadmium and PCB189 with smaller dioxin contributions. qgcomp showed net-null ψ for LDH with structured weights again consistent with internal counterbalancing. The prominence of cadmium is mechanistically coherent, and cadmium can induce hepatotoxicity through oxidative stress and inflammatory signaling, including disruption of Nrf2 pathways and activation of NF-κB/NLRP3-related responses [56].

### 4.6. TEQ Versus Concentration-Based Mixtures

A key contribution of the study is the explicit comparison between concentration-based mixtures and TEQ-weighted dioxin-like potency. The strong concordance of the core drivers in WQS and qgcomp after TEQ transformation (particularly the persistence of total bilirubin drivers and the recurring involvement of dioxin-like PCB TEQs) suggests the inference that mixture structure is not purely a concentration-ranking artifact. Rather, these patterns suggest that the mixture structure is broadly consistent with toxicological potency ordering [41,51]. At the same time, divergences (e.g., ALP and ALT directionality shifts in BKMR overall effects) likely reflect that TEQ captures only the AhR-relevant axis of the mixture and does not represent metal toxicity or non–dioxin-like PCB mechanisms.

### 4.7. Interaction Evidence Across Methods

BKMR single-variable interaction summaries were generally centered near zero in both the main-mixture and TEQ-weighted models, with uncertainty localized to a small subset of key contributors. This pattern suggests that observed mixture signals are driven primarily by main effects and marginal nonlinearity, with limited stable evidence for strong non-additive interaction structure consistent with BKMR’s documented ability to detect interactions when present but also its tendency to allocate signal to representative exposures under correlation [47].

Future studies should consider incorporating additional clinical and pharmacologic indicators that may influence liver biomarker variation, including underlying metabolic, infectious, renal, inflammatory, and liver-related conditions, as well as exposure to prescription medications or other drugs with potential hepatotoxic effects. These factors may confound, mediate, or modify the association between environmental chemical mixtures and liver biomarkers. However, their inclusion requires careful causal specification because some conditions may be consequences of environmental exposure rather than independent confounders. Adjusting for such variables without considering their causal role could result in overadjustment and attenuation of the total mixture effect.

### 4.8. Strengths and Limitations

#### 4.8.1. Strengths

A key strength of this work is the methodological triangulation applied to the same exposure panel and liver outcomes. The use of several mixture estimators also helps prevent misinterpretation of mixture-analysis results. By integrating multi-exposure linear regression with complementary mixture methods (WQS, qgcomp, and BKMR, including TEQ-weighted specifications), the study leverages approaches that target different mixture estimands and assumptions, allowing the study to distinguish robust signals from method-specific artifacts. The convergence of findings, particularly the repeated identification of a chemically sparse mixture architecture with a limited set of dominant contributors, strengthens confidence that the observed patterns reflect underlying mixture structure rather than idiosyncrasies of any single model.

Moreso, comprehensive phenotyping of liver function, which is the inclusion of eight biomarkers spanning hepatic synthetic function (albumin, total protein), hepatocellular injury (ALT, AST), cholestatic/hepatobiliary activity (ALP, total bilirubin), and generalized cellular/oxidative stress proxies (LDH, GGT) enable pathway-specific interpretation rather than reliance on a single marker. Additionally, the mixture decomposition and interpretability of WQS weight distributions, qgcomp positive and negative component weights, and BKMR group and conditional PIPs provide convergent evidence about which chemicals drive mixture signals, improving interpretability beyond a single overall mixture coefficient.

Also, the ability to capture complex exposure response features like BKMR’s flexible framework supports detection of nonlinearity and potential interactions, while qgcomp explicitly accommodates bidirectional mixture contributions, which is essential when sub mixtures exert opposing effects that can mask the net associations.

Toxicological relevance through TEQ comparison by evaluating both concentration-based mixtures and TEQ-weighted mixtures adds mechanistic specificity by testing whether associations align with dioxin-like potency structure, helping distinguish AhR-relevant mixture behavior from non–dioxin-like pathways. Lastly, the internal consistency checks across mixture definitions, such as the stability of key drivers across the main and TEQ-based formulations, supports the robustness of the identified mixture architecture, even when exposures are re-expressed on a potency-weighted scale.

#### 4.8.2. Limitations

This study has several limitations. First, the cross-sectional design of NHANES precludes causal inference, and temporal relationships between environmental exposures and liver biomarkers cannot be established. Second, exposure and outcome measurements were obtained at a single time point, which may not fully capture long-term exposure patterns or chronic liver function changes. Third, residual confounding may persist despite adjustment for key demographic and behavioral factors, including the potential influence of unmeasured variables such as underlying clinical conditions or medication use.

## 5. Conclusions

In summary, BKMR analysis of this mixture suggests subclinical hepatobiliary stress at higher exposures, with albumin suppression and bilirubin elevation as consistent signals. The TEQ approach sharpened the focus on dioxin-like toxicity, particularly affecting bilirubin and GGT, while the main-exposure model highlighted additional influences of metals. These findings are biologically plausible given known mechanisms by which POPs and metals affect the liver, but are tempered by statistical uncertainty. Major limitations include wide credible intervals and unmeasured confounding.

## Figures and Tables

**Table 1:** Descriptive characteristics of unadjusted covariates by gender presented as mean (s.d) or n (%).

n = 947		**Overall** **Mean (S.D)**	**Male** **Mean (S.D)**	**Female** **Mean (S.D)**
Age (years)		49.38 (19.12)	50.22 (19.05)	48.60 (19.17)
BMI		28.45 (6.34)	28.13 (5.17)	28.75 (7.25)
Income (*n*, %)	USD 0 to USD 14,999	160 (16.9%)	71 (15.6%)	89 (18.1%)
	USD 15,000 to USD 24,999	168 (17.7%)	68 (14.9%)	100 (20.3%)
	USD 25,000 to USD 44,999	231 (24.4%)	116 (25.5%)	115 (23.4%)
	USD 45,000 to USD 64,999	136 (14.4%)	72 (15.8%)	64 (13.0%)
	USD 65,000 to USD 74,999	54 (5.7%)	29 (6.4%)	25 (5.1%)
	USD 75,000 and over	189 (20.0%)	93 (20.4%)	96 (19.5%)
	Unavailable	9 (1.0%)	6 (1.3%)	3 (0.6%)
Ethnicity (*n*, %)	Mexican American	541 (57.1%)	265 (58.2%)	276 (56.1%)
	Other Hispanic	168 (17.7%)	76 (16.7%)	92 (18.7%)
	Non-Hispanic White	177 (18.7%)	80 (17.6%)	97 (19.7%)
	Non-Hispanic Black	31 (3.3%)	19 (4.2%)	12 (2.4%)
	Other Race	30 (3.2%)	15 (3.3%)	15 (3.0%)
Smokers (*n*, %)	No	480 (50.7%)	189 (41.5%)	291 (59.1%)
	Yes	467 (49.3%)	266 (58.5%)	201 (40.9%)
Alcohol (*n*, %)	No	294 (31.0%)	84 (18.5%)	210 (42.7%)
	Yes	653 (69.0%)	371 (81.5%)	282 (57.3%)

Values are presented as mean, standard deviation (s.d) for continuous variables, and n (%) for categorical variables. Gender, income, BMI, age, ethnicity, smoking status, and alcohol use were included to characterize the study population and were considered potential confounders in the adjusted mixture models. These variables provide context for the main chemical mixture and liver biomarker associations but are not the primary exposures of interest.

**Table 2:** Distribution of exposure variables by sex.

	Overall Mean (SD)	Male Mean (SD)	Female Mean (SD)	*p*-Value
Cadmium	0.57 (0.67)	0.56 (0.64)	0.58 (0.70)	0.6017
Lead	2.10 (2.09)	2.53 (2.32)	1.70 (1.77)	<0.0001
Mercury	1.69 (2.18)	1.81 (2.52)	1.58 (1.81)	0.1133
PCB28	5.60 (3.05)	5.54 (3.16)	5.65 (2.94)	0.5626
PCB66	1.81 (1.64)	1.66 (1.52)	1.95 (1.74)	0.0064
PCB74	9.19 (11.14)	7.91 (10.92)	10.37 (11.22)	0.0006
PCB105	2.43 (3.64)	2.04 (3.18)	2.78 (3.99)	0.0016
PCB118	12.32 (17.59)	10.53 (15.76)	13.96 (19.00)	0.0025
PCB156	6.29 (6.84)	6.95 (7.62)	5.68 (5.98)	0.0045
PCB157	1.51 (1.70)	1.64 (1.88)	1.39 (1.50)	0.0281
PCB167	1.58 (2.11)	1.54 (2.25)	1.61 (1.97)	0.6241
PCB189	0.40 (0.82)	0.44 (0.88)	0.36 (0.77)	0.1290
PCB126	28.45 (33.60)	25.52 (32.41)	31.16 (34.48)	0.0096
PCB81	5.91 (3.87)	6.10 (4.20)	5.73 (3.54)	0.1507
PCB169	18.07 (16.98)	20.63 (18.27)	15.70 (15.33)	<0.0001
1,2,3,7,8-PeCDD	5.11 (4.47)	5.01 (4.33)	5.21 (4.60)	0.4988
1,2,3,4,7,8-HxCDD	4.59 (3.73)	4.67 (3.94)	4.51 (3.52)	0.4919
1,2,3,6,7,8-HxCDD	31.57 (26.07)	32.57 (26.96)	30.65 (25.22)	0.2586
1,2,3,7,8,9-HxCDD	4.68 (3.67)	4.50 (3.36)	4.85 (3.93)	0.1400
1,2,3,4,6,7,8-HpCDD	39.54 (34.58)	36.34 (30.78)	42.49 (37.55)	0.0058
1,2,3,4,6,7,8,9-OCDD	322.70 (304.89)	278.64 (283.98)	363.45 (317.91)	<0.0001
2,3,7,8-TCDD	2.21 (2.43)	1.94 (2.13)	2.47 (2.66)	0.0007
2,3,7,8-TCDF	1.60 (0.77)	1.69 (0.90)	1.51 (0.61)	0.0006
1,2,3,7,8-PeCDF	1.69 (0.87)	1.79 (1.08)	1.60 (0.61)	0.0008
2,3,4,7,8-PeCDF	5.93 (4.90)	6.12 (5.43)	5.75 (4.35)	0.2466
1,2,3,4,7,8-HxCDF	4.62 (3.27)	4.79 (3.49)	4.46 (3.04)	0.1159
1,2,3,6,7,8-HxCDF	4.05 (3.09)	4.25 (3.32)	3.86 (2.85)	0.0527
1,2,3,7,8,9-HxCDF	1.94 (1.10)	2.02 (0.77)	1.87 (1.33)	0.0361
2,3,4,6,7,8-HxCDF	1.96 (0.83)	2.06 (0.90)	1.87 (0.76)	0.0004
1,2,3,4,6,7,8-HpCDF	9.05 (12.55)	10.17 (16.86)	8.02 (6.19)	0.0104
1,2,3,4,7,8,9-HpCDF	2.15 (1.90)	2.24 (0.95)	2.06 (2.47)	0.1343
1,2,3,4,6,7,8,9-OCDF	3.79 (6.72)	3.80 (3.24)	3.78 (8.80)	0.9573

*p*-values were computed using unadjusted two-sample *t*-tests.

**Table 3:** Unweighted multivariable linear regression adjusted for sex, age, race/ethnicity, income, smoking, alcohol use, and BMI.

	Albumin				ALP			ALT			AST	
Variable	Estimate	2.50%	97.50%	Estimate	2.50%	97.50%	Estimate	2.50%	97.50%	Estimate	2.50%	97.50%
Cadmium	0.0461	−0.1045	0.1967	0.4213	−0.8614	1.7207	−0.9975	−2.721	0.7565	−0.5954	−1.8945	0.7208
Lead	−0.0261	−0.2011	0.1489	1.7467	0.2381	3.278	0.958	−1.0812	3.0393	0.9497	−0.5815	2.5046
Mercury	0.1693	0.0120	0.3266	−0.6693	−1.9942	0.6734	3.6077	1.7246	5.5256	0.9706	−0.4071	2.3674
PCB28	−0.0687	−0.4401	0.3027	1.7972	−1.3792	5.076	−0.0601	−4.2956	4.3629	1.6921	−1.5536	5.0448
PCB66	−0.1198	−0.4860	0.2465	−2.1993	−5.2094	0.9065	0.7172	−3.4934	5.1115	−0.6063	−3.7354	2.6245
PCB74	0.2211	−0.2589	0.7011	−0.4423	−4.4387	3.7213	1.7611	−3.7777	7.6187	2.799	−1.4213	7.2001
PCB105	−0.6966	−1.2653	−0.1279	2.4862	−2.3701	7.5841	−6.0761	−12.1025	0.3635	−1.8964	−6.6504	3.0996
PCB118	0.8545	0.0818	1.6272	−0.912	−7.2368	5.844	11.8678	2.2291	22.4153	4.0539	−2.7369	11.3189
PCB156	−0.2703	−1.5360	0.9954	−7.2048	−16.7073	3.3819	−3.1291	−16.4203	12.2758	−5.457	−15.3511	5.5936
PCB157	−0.1282	−1.4647	1.2082	8.9626	−2.7845	22.1291	6.8019	−8.6091	24.8116	5.8508	−5.8106	18.956
PCB167	0.2427	−0.7098	1.1952	−4.9377	−12.3605	3.1137	−14.86	−23.8095	−4.8593	−9.9472	−17.1354	−2.1354
PCB189	−0.0147	−0.1427	0.1134	−0.0046	−1.0915	1.0942	−0.196	−1.6749	1.3052	0.4503	−0.6667	1.5799
PCB126	0.0441	−0.3124	0.4005	1.7113	−1.3367	4.8535	0.56	−3.5339	4.8276	1.9174	−1.2066	5.1402
PCB81	0.1211	−0.1362	0.3785	−0.8151	−2.97	1.3876	−0.032	−2.987	3.013	−0.3959	−2.6095	1.868
PCB169	−0.3593	−1.0067	0.2881	−0.4651	−5.8163	5.1902	−0.1898	−7.447	7.6364	−0.5713	−6.0374	5.2129
1,2,3,7,8-PeCDD	−0.2459	−0.9194	0.4277	−2.0143	−7.4891	3.7845	5.8802	−2.1175	14.5314	3.0132	−2.8723	9.2553
1,2,3,4,7,8-HxCDD	0.0141	−0.2824	0.3106	−0.0692	−2.5666	2.4921	0.0476	−3.3522	3.567	−0.4406	−2.9857	2.1713
1,2,3,6,7,8-HxCDD	−1.0380	−1.7303	−0.3457	4.2794	−1.7041	10.6272	−12.564	−19.3447	−5.2133	−9.9784	−15.2602	−4.3674
1,2,3,7,8,9-HxCDD	−0.0485	−0.3469	0.2500	2.1233	−0.4454	4.7583	0.7357	−2.7095	4.303	0.7934	−1.7999	3.4553
1,2,3,4,6,7,8-HpCDD	0.1571	−0.2577	0.5719	−2.0051	−5.414	1.5267	4.7534	−0.1925	9.9445	1.4404	−2.1689	5.1828
1,2,3,4,6,7,8,9-OCDD	0.2729	−0.0631	0.6089	−1.4812	−4.2666	1.3852	−1.2464	−5.0406	2.6993	0.0684	−2.8255	3.0485
2,3,7,8-TCDD	0.4036	0.0770	0.7301	1.2858	−1.4983	4.1486	0.8496	−2.9181	4.7635	0.9688	−1.8702	3.8898
2,3,7,8-TCDF	0.0162	−0.3124	0.3447	−1.3079	−4.0373	1.4992	1.6819	−2.14	5.6531	1.4038	−1.4649	4.3559
1,2,3,7,8-PeCDF	−0.3254	−0.6996	0.0487	1.2097	−1.9713	4.4939	−3.8905	−7.9931	0.3949	−1.6066	−4.7698	1.6616
2,3,4,7,8-PeCDF	0.1168	−0.4530	0.6867	−2.1983	−6.8417	2.6765	12.0509	4.8474	19.7492	8.7425	3.4627	14.2917
1,2,3,4,7,8-HxCDF	−0.0377	−0.5539	0.4786	−1.2264	−5.4843	3.2234	0.9827	−4.9165	7.248	1.9128	−2.5802	6.613
1,2,3,6,7,8-HxCDF	0.0883	−0.3764	0.5530	1.1367	−2.7966	5.2291	−1.7052	−6.8898	3.768	−0.7111	−4.6604	3.4017
1,2,3,7,8,9-HxCDF	−0.1874	−0.6921	0.3173	1.6289	−2.6559	6.1023	4.6504	−1.3299	10.9932	4.2628	−0.2329	8.9611
2,3,4,6,7,8-HxCDF	0.4670	−0.0490	0.9830	−1.4383	−5.6853	2.9998	−4.6746	−10.2409	1.2369	−5.0815	−9.2643	−0.7059
1,2,3,4,6,7,8-HpCDF	−0.0515	−0.1554	0.0525	0.4367	−0.4507	1.332	−0.7145	−1.9108	0.4965	−0.7702	−1.6671	0.135
1,2,3,4,7,8,9-HpCDF	0.1334	−0.1713	0.4382	−0.0202	−2.5872	2.6145	−1.9203	−5.3441	1.6273	−0.5113	−3.1242	2.1722
1,2,3,4,6,7,8,9-OCDF	0.0129	−0.1444	0.1701	−0.9146	−2.2358	0.4245	0.3403	−1.4829	2.1973	−0.7521	−2.106	0.6205
		**GGT**			**LDH**			**Total Bilirubin**			**Total Protein**	
**Variable**	**Estimate**	**2.50%**	**97.50%**	**Estimate**	**2.50%**	**97.50%**	**Estimate**	**2.50%**	**97.50%**	**Estimate**	**2.50%**	**97.50%**
Cadmium	3.3827	0.7203	6.1156	−0.9581	−1.7566	−0.1531	−1.1588	−2.5257	0.2272	0.0911	−0.1197	0.3018
Lead	1.0131	−2.0033	4.1223	1.465	0.5151	2.4239	1.9347	0.2986	3.5975	−0.0765	−0.3213	0.1684
Mercury	0.0324	−2.6567	2.7958	0.1917	−0.6518	1.0424	1.0814	−0.3781	2.5623	0.1487	−0.0714	0.3688
PCB28	0.0359	−6.1974	6.6834	0.6884	−1.3015	2.7185	0.1399	−3.2403	3.6382	0.2811	−0.2385	0.8007
PCB66	1.7466	−4.5083	8.4112	−1.6223	−3.5399	0.3334	0.7363	−2.6178	4.2059	−0.4736	−0.9861	0.0388
PCB74	−8.8116	−16.0871	−0.9054	1.7683	−0.8235	4.4279	1.6557	−2.757	6.2685	0.2373	−0.4343	0.9088
PCB105	2.2392	−7.3531	12.8247	0.3987	−2.6238	3.515	−3.7131	−8.6453	1.4854	−0.4106	−1.2063	0.3851
PCB118	−0.684	−13.1271	13.5414	0.5977	−3.4947	4.8638	7.3434	−0.0582	15.2931	0.3437	−0.7374	1.4249
PCB156	8.7648	−12.649	35.4281	1.3712	−5.2954	8.5071	−2.4987	−13.2661	9.6054	0.5826	−1.1882	2.3533
PCB157	−7.864	−26.9054	16.1377	−1.4894	−8.3172	5.8469	−2.0119	−13.4014	10.8757	−0.969	−2.8388	0.9008
PCB167	−2.3255	−17.1822	15.1963	−3.013	−7.8531	2.0813	−2.3613	−10.592	6.627	0.2754	−1.0572	1.608
PCB189	0.7556	−1.4545	3.0153	0.7198	0.029	1.4153	0.027	−1.1502	1.2181	0.0044	−0.1748	0.1835
PCB126	3.7201	−2.4907	10.3266	0.8481	−1.0656	2.7988	0.4277	−2.8282	3.7926	0.4006	−0.0981	0.8993
PCB81	−1.8215	−6.1019	2.654	−0.4868	−1.8537	0.8991	1.9108	−0.4852	4.3646	0.1727	−0.1874	0.5327
PCB169	−5.422	−15.4559	5.8027	−1.9598	−5.3126	1.5117	1.5208	−4.3778	7.7832	−0.3076	−1.2134	0.5982
1,2,3,7,8-PeCDD	7.7236	−4.1405	21.056	1.9857	−1.6404	5.7456	−0.3434	−6.3605	6.0603	−0.8479	−1.7903	0.0945
1,2,3,4,7,8-HxCDD	−0.0705	−5.0737	5.1963	1.5099	−0.095	3.1405	−0.1034	−2.8048	2.673	−0.139	−0.5539	0.2758
1,2,3,6,7,8-HxCDD	−10.2362	−20.3808	1.2009	−3.5629	−7.0853	0.093	−7.6857	−13.4094	−1.5837	−0.296	−1.2646	0.6726
1,2,3,7,8,9-HxCDD	2.5862	−2.5828	8.0295	−0.8902	−2.4674	0.7124	−0.465	−3.174	2.3198	0.0027	−0.4149	0.4203
1,2,3,4,6,7,8-HpCDD	1.4202	−5.6117	8.9761	−0.4925	−2.6864	1.7509	1.5016	−2.3174	5.4699	−0.0836	−0.6639	0.4968
1,2,3,4,6,7,8,9-OCDD	−1.9438	−7.4882	3.933	0.9679	−0.8391	2.8079	3.1763	0.0203	6.4318	0.3696	−0.1005	0.8396
2,3,7,8-TCDD	1.5448	−4.0398	7.4545	−0.1775	−1.9142	1.5899	−0.2048	−3.1727	2.8541	0.3125	−0.1444	0.7693
2,3,7,8-TCDF	1.1453	−4.451	7.0694	−0.0264	−1.7764	1.7549	0.3272	−2.6748	3.4218	0.2518	−0.2079	0.7115
1,2,3,7,8-PeCDF	−3.124	−9.2035	3.3625	−0.4512	−2.433	1.5708	1.5991	−1.8552	5.175	−0.1651	−0.6885	0.3583
2,3,4,7,8-PeCDF	11.184	0.7326	22.7199	0.6571	−2.3791	3.7878	−0.0193	−5.1507	5.3898	−0.154	−0.9513	0.6433
1,2,3,4,7,8-HxCDF	−0.8195	−9.304	8.4588	0.5578	−2.194	3.387	−2.6873	−7.2229	2.0701	0.0199	−0.7024	0.7422
1,2,3,6,7,8-HxCDF	0.8801	−6.9228	9.3371	−0.0174	−2.4838	2.5113	3.0514	−1.2826	7.5756	0.2588	−0.3914	0.909
1,2,3,7,8,9-HxCDF	4.0521	−4.658	13.558	1.7196	−1.0023	4.5164	1.7587	−2.8802	6.6192	−0.2785	−0.9846	0.4275
2,3,4,6,7,8-HxCDF	−4.3523	−12.5311	4.5912	−0.0949	−2.8276	2.7146	−1.2505	−5.8511	3.5748	0.006	−0.7159	0.728
1,2,3,4,6,7,8-HpCDF	−1.538	−3.2955	0.2514	−0.1281	−0.6847	0.4315	−0.9699	−1.9173	−0.0134	−0.1055	−0.2509	0.04
1,2,3,4,7,8,9-HpCDF	−1.4374	−6.5054	3.9054	−1.376	−2.9782	0.2526	−0.7935	−3.5496	2.0413	0.3322	−0.0942	0.7585
1,2,3,4,6,7,8,9-OCDF	−0.3605	−3.0384	2.3913	0.0231	−0.8188	0.8722	−0.1695	−1.6106	1.2927	−0.1107	−0.3307	0.1093

Adjusted for gender, age, ethnicity, income, smoking, alcohol, and BMI.

## Data Availability

The NHANES dataset is publicly available online, accessible at https://wwwn.cdc.gov/nchs/nhanes/continuousnhanes/overview.aspx?BeginYear=2003 (accessed on 1 May 2025).

## Supplementary Materials

The following supporting information can be downloaded at https://www.mdpi.com/article/10.3390/toxics14050418/s1, Figure S1: Spearman correlation matrix for albumin and the exposure variables. Figures S2–S9: Univariate dose response functions of exposures on albumin, ALP, ALT, AST, GGT, LDH, TB, and TP, respectively. Figures S10–S17: Overall exposure effect summaries for albumin, ALP, ALT, AST, GGT, LDH, TB, and TP, respectively. Figures S18–S25: Single-variable effects for albumin, ALP, ALT, AST, GGT, LDH, TB, and TP, respectively. Figures S26–S33: Single-variable interaction effects for albumin, ALP, ALT, AST, GGT, LDH, TB, and TP, respectively. Figures S34–S41: TEQ-based univariate dose–response relationships for albumin, ALP, ALT, AST, GGT, LDH, TB, and TP, respectively. Figures S42–S49: TEQ-based overall exposure effect summaries for albumin, ALP, ALT, AST, GGT, LDH, TB, and TP, respectively. Figures S50–S57: TEQ-based single-variable effects for albumin, ALP, ALT, AST, GGT, LDH, TB, and TP, respectively. Figures S58–S65: TEQ-based single-variable interaction effects for albumin, ALP, ALT, AST, GGT, LDH, TB, and TP, respectively. Figures S66–S73: Weighted Quantile Sum (WQS) regression results for the main exposure mixture for albumin, ALP, ALT, AST, GGT, LDH, TB, and TP, respectively. Figures S74–S81: WQS regression results for the combined metals and dioxin-like TEQ mixture for albumin, ALP, ALT, AST, GGT, LDH, TB, and TP, respectively. Figures S82–S89: Quantile g-computation results for albumin, ALP, ALT, AST, GGT, LDH, TB, and TP, respectively. Tables S1–S8: Posterior inclusion probability (PIP), group PIP, and conditional PIP estimates for albumin, ALP, ALT, AST, GGT, LDH, total bilirubin, and total protein, respectively.

## Appendix A. Study Key and Chemical Code Name

**Table:**

Chemical Group	Chemical Code/Name	Study Key	Description
PCBs	PCB28 Lipid Adj (ng/g)	PCB28	Polychlorinated biphenyl congener 28; persistent organic pollutant, bioaccumulative.
	PCB66 Lipid Adj (ng/g)	PCB66	PCB congener 66; moderately chlorinated PCB, bioaccumulative, persistent pollutant.
	PCB74 Lipid Adj (ng/g)	PCB74	PCB congener 74; persistent, bioaccumulative, potentially toxic pollutant.
	PCB105 Lipid Adj (ng/g)	PCB105	PCB congener 105; dioxin-like, bioaccumulative, toxic pollutant.
	PCB118 Lipid Adj (ng/g)	PCB118	PCB congener 118; dioxin-like PCB, persistent, bioaccumulative, toxic.
	PCB156 Lipid Adj (ng/g)	PCB156	PCB congener 156; highly chlorinated, dioxin-like, persistent pollutant.
	PCB157 Lipid Adj (ng/g)	PCB157	PCB congener 157; persistent organic pollutant, moderate toxicity.
	PCB167 Lipid Adj (ng/g)	PCB167	PCB congener 167; environmentally persistent, potential toxicological concern.
	PCB189 Lipid Adj (ng/g)	PCB189	PCB congener 189; highly chlorinated, very persistent, known for bioaccumulation.
	PCB126-3,3′,4,4′,5-pncb Lipid Adj (pg/g)	PCB126	Highly toxic, dioxin-like PCB congener; potent bioaccumulative chemical.
	PCB81-3,4,4′,5-tcb Lipid Adj (pg/g)	PCB81	Dioxin-like PCB congener; highly toxic, persistent pollutant.
	PCB169-3,3′,4,4′,5,5′-hxcb Lipid Adj (pg/g)	PCB169	Highly chlorinated dioxin-like PCB, significant bioaccumulation, toxicity.
Dioxins	LBXD01LA– 1,2,3,7,8-Pentachlorodibenzo-p-dioxin Lipid Adj (pg/g)	Dioxin1- 1,2,3,7,8-PeCDD	Pentachlorodibenzo-p-dioxin; highly toxic, persistent environmental contaminant.
	LBXD02LA– 1,2,3,4,7,8-Hexachlorinated dioxin Lipid Adj (pg/g)	Dioxin2- 1,2,3,4,7,8-HxCDD	Hexachlorinated dioxin; highly toxic, persistent, carcinogenic contaminant.
	LBXD03LA– 1,2,3,6,7,8-Hexachlorinated dioxin Lipid Adj (pg/g)	Dioxin3- 1,2,3,6,7,8-HxCDD	Hexachlorinated dioxin; persistent, bioaccumulative, highly toxic.
	LBXD04LA– 1,2,3,7,8,9-Hexachlorinated dioxin Lipid Adj (pg/g)	Dioxin4- 1,2,3,7,8,9-HxCDD	Hexachlorinated dioxin; persistent organic pollutant, toxicological concern.
	LBXD05LA– 1,2,3,4,6,7,8-Heptachlorinated dioxin Lipid Adj (pg/g)	Dioxin5- 1,2,3,4,6,7,8-HpCDD	Heptachlorinated dioxin; persistent, bioaccumulative, toxic contaminant.
	LBXD07LA– 1,2,3,4,6,7,8,9-Octachlorinated dioxin Lipid Adj (pg/g)	Dioxin6-1,2,3,4,6,7,8,9-OCDD	Octachlorinated dioxin (OCDD); highly persistent, bioaccumulative, toxic environmental pollutant.
	LBXTCDLA– 2,3,7,8-Tetrachlorinated dioxin Lipid Adj (pg/g)	Dioxin7- 2,3,7,8-TCDD	Tetrachlorodibenzo-p-dioxin (TCDD); extremely toxic, carcinogenic, persistent pollutant.
Furans (PCDFs)	LBXF01LA– 2,3,7,8-Tetrachloro dibenzofuran Lipid Adj (pg/g)	Furan1- 2,3,7,8-TCDF	Tetrachlorodibenzofuran (TCDF); toxic, bioaccumulative, environmentally persistent.
	LBXF02LA– 1,2,3,7,8-Pentachlorinated dibenzofuran Lipid Adj (pg/g)	Furan2- 1,2,3,7,8-PeCDF	Pentachlorinated dibenzofuran; highly toxic, persistent contaminant.
	LBXF03LA– 2,3,4,7,8-Pentachlorinated dibenzofuran Lipid Adj (pg/g)	Furan3- 2,3,4,7,8-PeCDF	Pentachlorinated dibenzofuran; toxic, persistent environmental pollutant.
	LBXF04LA– 1,2,3,4,7,8-Hexachlorinated dibenzofuran Lipid Adj (pg/g)	Furan4- 1,2,3,4,7,8-HxCDF	Hexachlorinated dibenzofuran; persistent, bioaccumulative, toxic pollutant.
	LBXF05LA– Lipid Adj (pg/g)	Furan5- 1,2,3,6,7,8-HxCDF	Hexachlorinated dibenzofuran; toxic, persistent, bioaccumulative contaminant.
	LBXF06LA– 1,2,3,7,8,9-Hexachlorinated dibenzofuran Lipid Adj (pg/g)	Furan6- 1,2,3,7,8,9-HxCDF	Hexachlorinated dibenzofuran; persistent pollutant with significant toxicity.
	LBXF07LA– 2,3,4,6,7,8-Hexachlorinated dibenzofuran Lipid Adj (pg/g)	Furan7- 2,3,4,6,7,8-HxCDF	Hexachlorinated dibenzofuran; toxic, persistent, bioaccumulative chemical.
	LBXF08LA– 1,2,3,4,6,7,8-Heptachlorinated dibenzofuran Lipid Adj (pg/g)	Furan8- 1,2,3,4,6,7,8-HpCDF	Heptachlorinated dibenzofuran; persistent, bioaccumulative, toxic contaminant.
	LBXF09LA– 1,2,3,4,7,8,9-Heptachlorinated dibenzofuran Lipid Adj (pg/g)	Furan9- 1,2,3,4,7,8,9-HpCDF	Heptachlorinated dibenzofuran; persistent, toxic environmental pollutant.
	LBXF10LA– 1,2,3,4,6,7,8,9-Octachlorinated dibenzofuran Lipid Adj (pg/g)	Furan10-1,2,3,4,6,7,8,9-OCDF	Octachlorinated dibenzofuran (OCDF); highly chlorinated, persistent pollutant.

## Appendix B

### Appendix B.1. PCB Congeners WHO TEF Values and TEQ Eligibility

**Table:**

Chemical Group	Study Key	Congener	Dioxin-Like (DL) PCB Class	WHO 2005 TEF	TEQ Eligible?
	PCB28	PCB congener 28	Non–DL PCB	—	No
	PCB66	PCB congener 66	Non–DL PCB	—	No
	PCB74	PCB congener 74	Non–DL PCB	—	No
	PCB105	2,3,3′,4,4′-pentaCB	Mono-ortho DL-PCB	0.00003	Yes
	PCB118	2,3′,4,4′,5-pentaCB	Mono-ortho DL-PCB	0.00003	Yes
PCBs	PCB156	2,3,3′,4,4′,5-hexaCB	Mono-ortho DL-PCB	0.00003	Yes
	PCB157	2,3,3′,4,4′,5′-hexaCB	Mono-ortho DL-PCB	0.00003	Yes
	PCB167	2,3′,4,4′,5,5′-hexaCB	Mono-ortho DL-PCB	0.00003	Yes
	PCB189	2,2′,3,4,4′,5,5′-heptaCB	Mono-ortho DL-PCB	0.00003	Yes
	PCB81	3,4,4′,5-tetraCB	Non-ortho DL-PCB	0.0003	Yes
	PCB126	3,3′,4,4′,5-pentaCB	Non-ortho DL-PCB	0.1	Yes
	PCB169	3,3′,4,4′,5,5′-hexaCB	Non-ortho DL-PCB	0.03	Yes

### Appendix B.2. Dioxins and Furans Congener WHO TEF Values

**Table:**

Chemical Group	Study Key	Congener	WHO 2005 TEF
	Dioxin1	1,2,3,7,8-PeCDD	1
	Dioxin2	1,2,3,4,7,8-HxCDD	0.1
	Dioxin3	1,2,3,6,7,8-HxCDD	0.1
Dioxins (PCDDs)	Dioxin4	1,2,3,7,8,9-HxCDD	0.1
	Dioxin5	1,2,3,4,6,7,8-HpCDD	0.01
	Dioxin6	OCDD (1,2,3,4,6,7,8,9-OCDD)	0.0003
	Dioxin7	2,3,7,8-TCDD	1
	Furan1	2,3,7,8-TCDF	0.1
	Furan2	1,2,3,7,8-PeCDF	0.03
	Furan3	2,3,4,7,8-PeCDF	0.3
	Furan4	1,2,3,4,7,8-HxCDF	0.1
Furans (PCDFs)	Furan5	1,2,3,6,7,8-HxCDF	0.1
	Furan6	1,2,3,7,8,9-HxCDF	0.1
	Furan7	2,3,4,6,7,8-HxCDF	0.1
	Furan8	1,2,3,4,6,7,8-HpCDF	0.01
	Furan9	1,2,3,4,7,8,9-HpCDF	0.01
	Furan10	OCDF (1,2,3,4,6,7,8,9-OCDF)	0.0003

## Appendix C. Classification of Chemical Contributors Across Mixture Analyses

**Table:**

Evidence Category	Chemicals	Basis for Classification Across Regression, WQS, qgcomp, and BKMR	Interpretation
	Lead	Strong BKMR evidence for ALP, with high metal group contribution and lead dominating the conditional PIP within the metal group. Also appeared as an important contributor in mixture-weighted analyses.	Suggests lead may be an important contributor to cholestatic or biliary-related liver biomarker variation, especially ALP.
	Mercury	Strong BKMR evidence for ALT, where mercury dominated the metal group contribution. Mercury also appeared prominently in several WQS/qgcomp mixture-weight summaries.	Suggests mercury may contribute to hepatocellular biomarker variation, especially ALT-related patterns.
	PCB28	Very strong BKMR signal for AST, with the PCB group showing high group PIP and PCB28 dominating the conditional PIP.	Indicates a strong outcome-specific association with AST, but interpretation should consider PCB correlation and model dependence.
Stronger recur-ring or out-come-specific contributors	PCB81	Strong BKMR signal for total bilirubin, with PCB81 dominating the PCB group contribution.	Suggests possible relevance to bilirubin-related variation, potentially reflecting hepatic transport, metabolism, or systemic processing pathways.
	PCB126 and PCB169	Appeared repeatedly in WQS/qgcomp and TEQ-related analyses; PCB126 was relevant in ALT mixture patterns, while PCB169 appeared in albumin/ALP-related BKMR conditional patterns.	Supports the importance of dioxin-like PCB activity, especially when toxic potency is considered.
	Dioxin3	Repeatedly showed high conditional PIP within the dioxin group for several outcomes, including albumin, ALP, ALT, and total bilirubin, although group-level strength varied by outcome.	Suggests Dioxin3 is a recurring dioxin-related contributor, but its evidence is stronger for some biomarkers than others.
	Furan3, Furan8, Furan10	Furan3 was important for ALP, Furan8 for ALT-related patterns, and Furan10 for total bilirubin-related patterns in mixture models.	Indicates that furans may contribute to an outcome-specific manner rather than as uniformly strong predictors across all biomarkers.
Moderate or model-dependent contributors	Cadmium	Showed moderate evidence in some models, including GGT- and LDH-related patterns, but did not consistently dominate across biomarkers.	May contribute to liver biomarker variation under selected model assumptions or outcomes, but evidence was less consistent than lead or mercury.
	PCB105, PCB156, PCB157, PCB167, PCB189	Appeared in selected WQS/qgcomp or BKMR patterns but generally showed lower or less consistent contribution than PCB28, PCB81, PCB126, or PCB169.	These PCBs may be relevant in specific outcome contexts, but their evidence was not sufficiently consistent to classify them as dominant contributors.
	Dioxin1, Dioxin4, Dioxin5, Dioxin6, Dioxin7	Some dioxins contributed in WQS/qgcomp or conditional BKMR summaries, especially for GGT and albumin-related patterns, but their evidence varied across biomarkers.	Suggests possible class-level importance of dioxins, with individual congeners showing variable or model-dependent contribution.
	Furan2, Furan5, Furan6, Furan7, Furan9	Showed selected contributions in some WQS/qgcomp or BKMR models but did not repeatedly dominate across outcomes.	Supports possible outcome-specific contribution, but evidence remains moderate and should be interpreted as sensitivity-level evidence.
	PCB66 and PCB74	Generally low BKMR conditional contribution and limited recurring dominance in WQS/qgcomp summaries.	Limited evidence of independent or dominant contribution in the present dataset.
	PCB118	Although biologically relevant as a dioxin-like PCB, it did not consistently dominate the mixture analyses and showed relatively low BKMR contribution in several outcomes.	Should be interpreted as low or inconsistent statistical evidence in this dataset, not as evidence of no hepatotoxic potential.
Low-association or inconsistent contributors	Dioxin2	Generally lower or inconsistent contribution across BKMR and mixture-weighted summaries.	Limited evidence of dominant association under the current model structure.
	Furan1 and Furan4	Generally weak or inconsistent contribution across models, with no stable dominance across liver biomarkers.	Low evidence of association in this analysis; future studies may clarify whether effects appear under different exposure ranges or latency windows.
	Total protein outcome across chemicals	BKMR PIP values for total protein were essentially null across the evaluated chemicals.	Suggests limited evidence that the mixture explained total protein variation in the present dataset.

Classification reflects the consistency and relative strength of evidence across multiple-exposure linear regression, WQS, qgcomp, and BKMR models. Stronger recurring contributors were chemicals repeatedly identified across methods or showing strong outcome-specific dominance. Moderate contributors were chemicals identified only in selected outcomes or methods. Low-association contributors were chemicals with weak, unstable, or inconsistent evidence across models. These categories are not toxicological rankings and should not be interpreted as proof that low-association chemicals lack hepatotoxic potential.
