# Supplementary material for: Mixture Effects of Metals, PCBs, Dioxins, and Furans on Liver Function
Source: Toxics. 2026 May 11;14(5):418. doi: 10.3390/toxics14050418 (PMC13211406; doi:10.3390/toxics14050418)
Supplement: Supplementary file 1 [file toxics-14-00418-s001.zip › toxics-4283373-supplementary.pdf]

SUPPLEMENTARY DOCUMENTS

Supplementary Materials. Figure S1: Spearman correlation matrix for albumin and the exposure variables. Figures S2–S9: Univariate dose-response functions of exposures on albumin, ALP, ALT, AST, GGT, LDH, TB, and TP, respectively. Figures S10–S17: Overall exposure effect summaries for albumin, ALP, ALT, AST, GGT, LDH, TB, and TP, respectively. Figures S18–S25: Single-variable effects for albumin, ALP, ALT, AST, GGT, LDH, TB, and TP, respectively. Figures S26–S33: Single-variable interaction effects for albumin, ALP, ALT, AST, GGT, LDH, TB, and TP, respectively. Figures S34–S41: TEQ-based univariate dose–response relationships for albumin, ALP, ALT, AST, GGT, LDH, TB, and TP, respectively. Figures S42–S49: TEQ-based overall exposure effect summaries for albumin, ALP, ALT, AST, GGT, LDH, TB, and TP, respectively. Figures S50–S57: TEQ-based single-variable effects for albumin, ALP, ALT, AST, GGT, LDH, TB, and TP, respectively. Figures S58–S65: TEQ-based single-variable interaction effects for albumin, ALP, ALT, AST, GGT, LDH, TB, and TP, respectively. Figures S66–S73: Weighted Quantile Sum (WQS) regression results for the main exposure mixture for albumin, ALP, ALT, AST, GGT, LDH, TB, and TP, respectively. Figures S74–S81: WQS regression results for the combined metals and dioxin-like TEQ mixture for albumin, ALP, ALT, AST, GGT, LDH, TB, and TP, respectively. Figures S82–S89: Quantile g-computation results for albumin, ALP, ALT, AST, GGT, LDH, TB, and TP, respectively. Tables S1–S8: Posterior inclusion probability (PIP), group PIP, and conditional PIP estimates for albumin, ALP, ALT, AST, GGT, LDH, total bilirubin, and total protein, respectively.

SUPPLEMENTARY DOCUMENTS

Spearman Correlation

S1. ALBUMIN

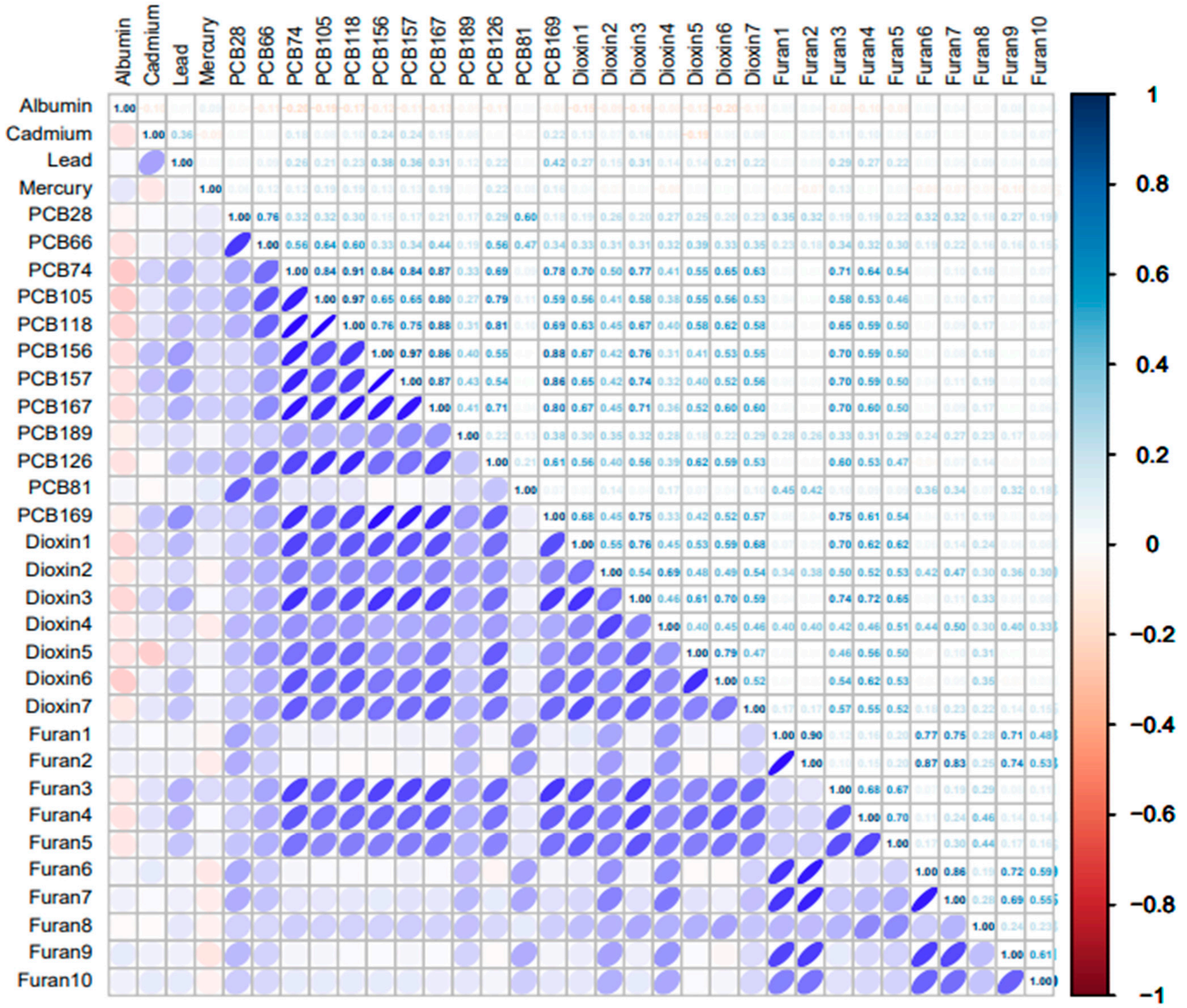

SUPPLEMENTARY DOCUMENTS

UNIVARIATE DOSE-RESPONSE

S2. ALBUMIN

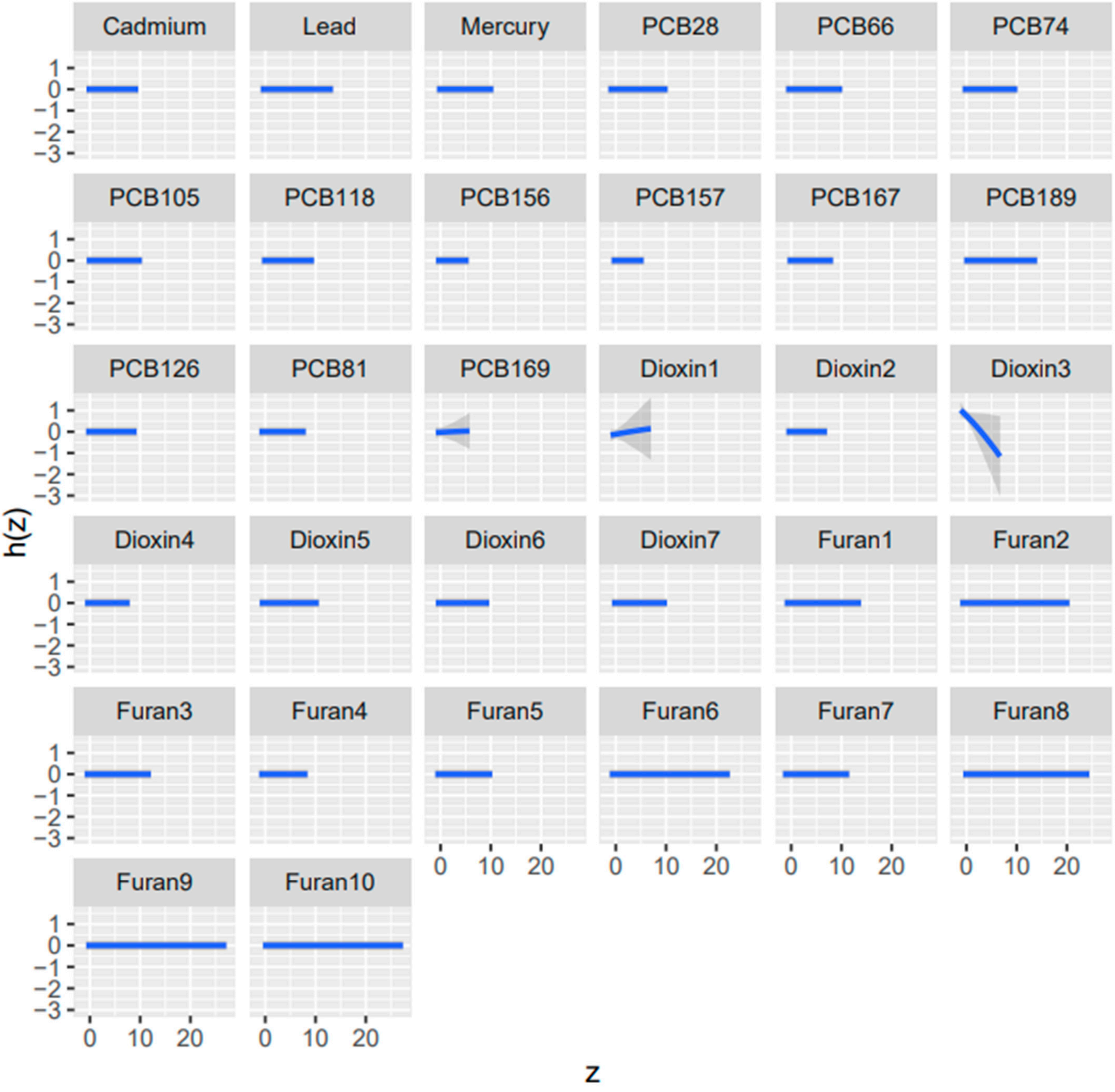

SUPPLEMENTARY DOCUMENTS

S3. ALKALINE PHOSPHATASE (ALP)

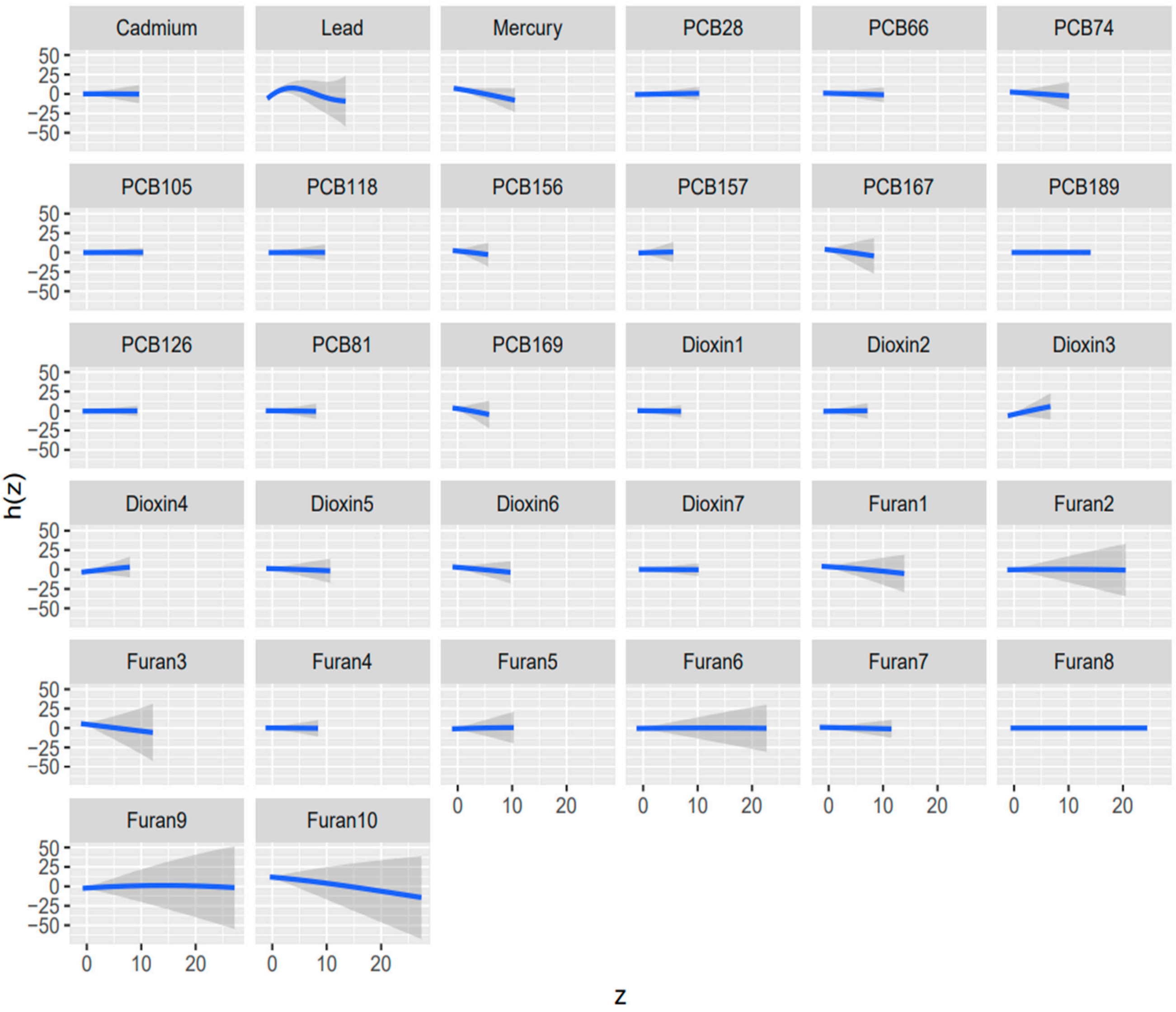

SUPPLEMENTARY DOCUMENTS

S4. ALANINE AMINOTRANSFERASE (ALT)

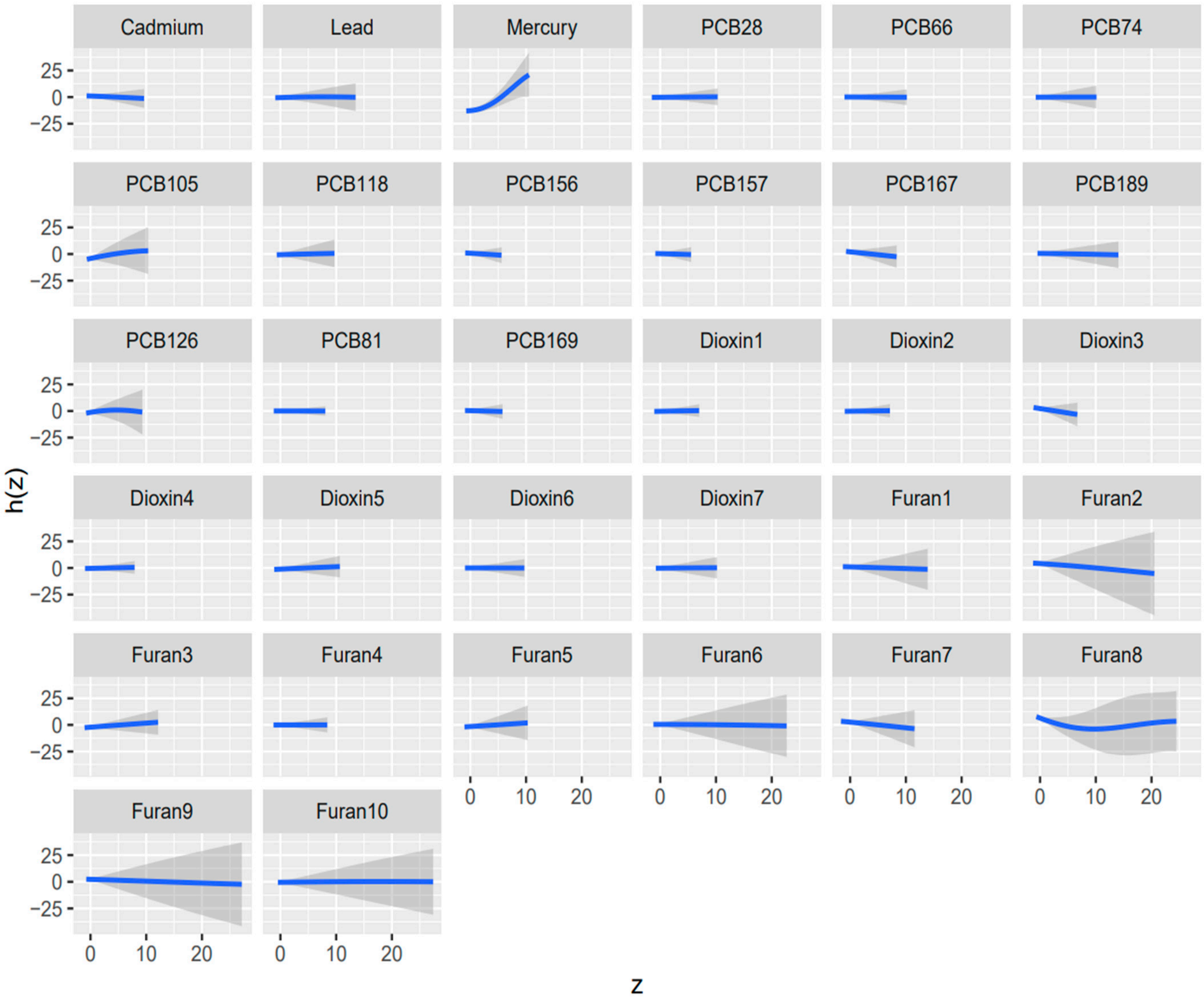

SUPPLEMENTARY DOCUMENTS

S5. ASPARTATE AMINOTRANSFERASE(AST)

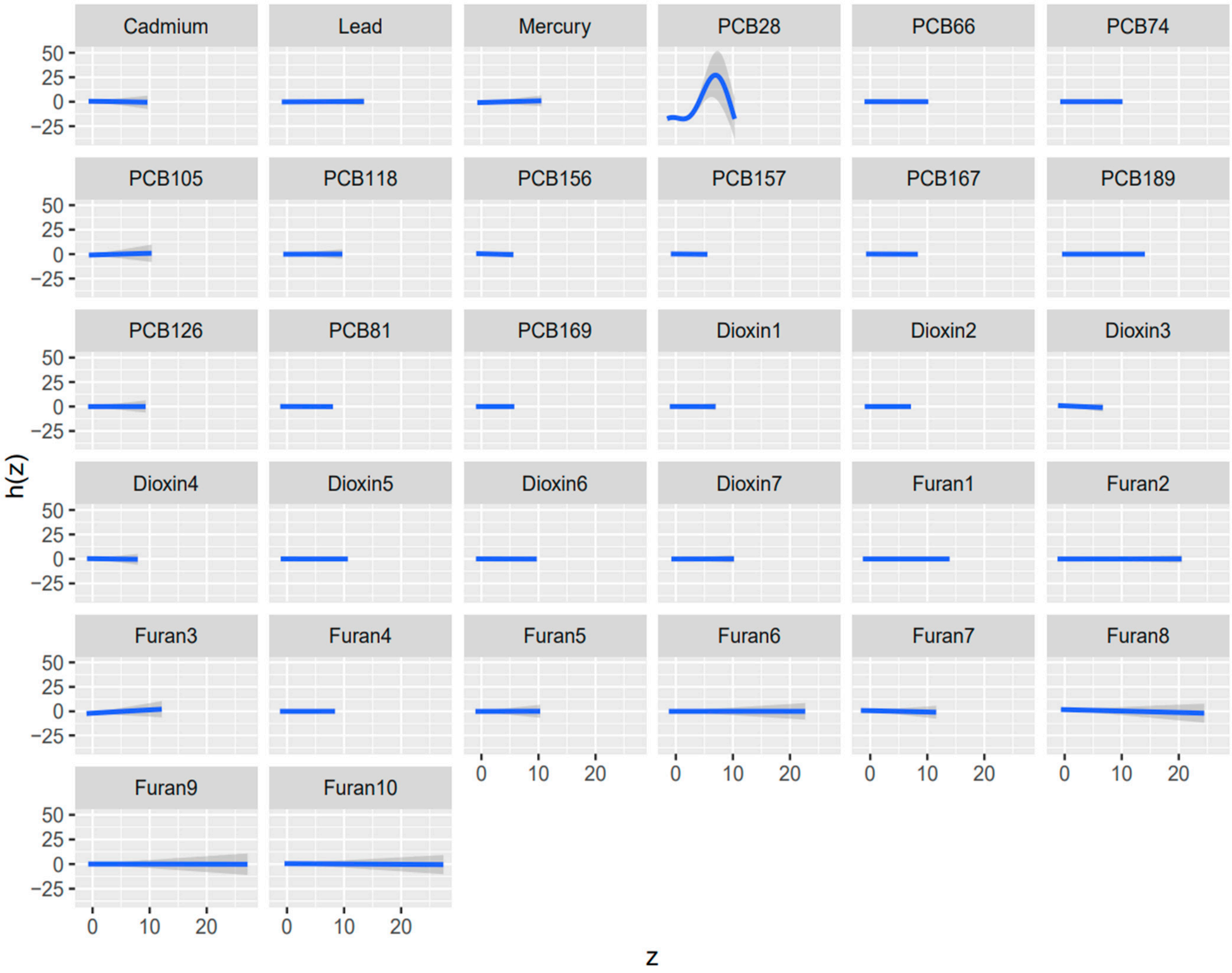

SUPPLEMENTARY DOCUMENTS

S6. GAMMA GLUTAMYL TRANSFERASE (GGT)

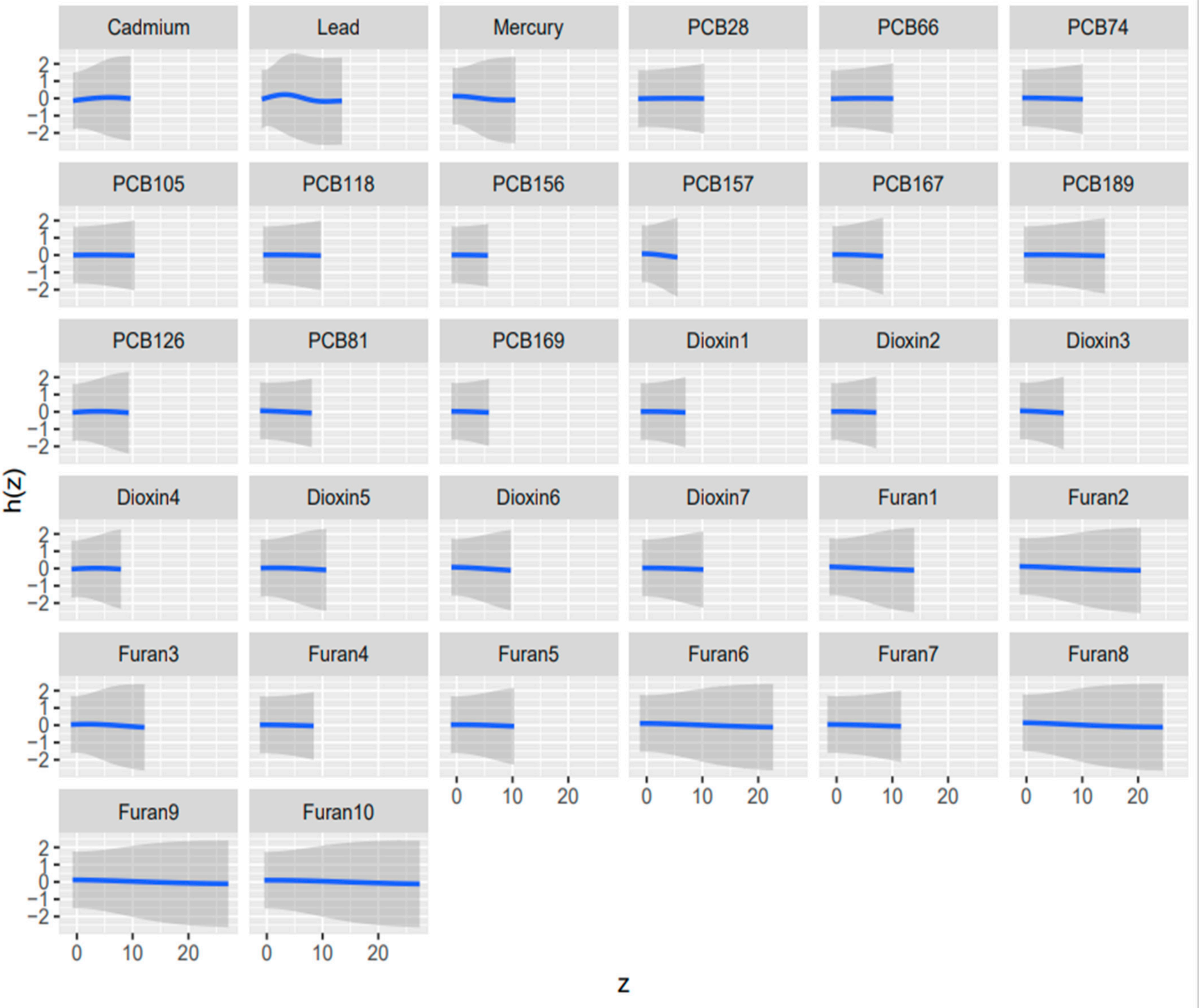

SUPPLEMENTARY DOCUMENTS

S7. LACTATE DEHYDROGENASE (LDH)

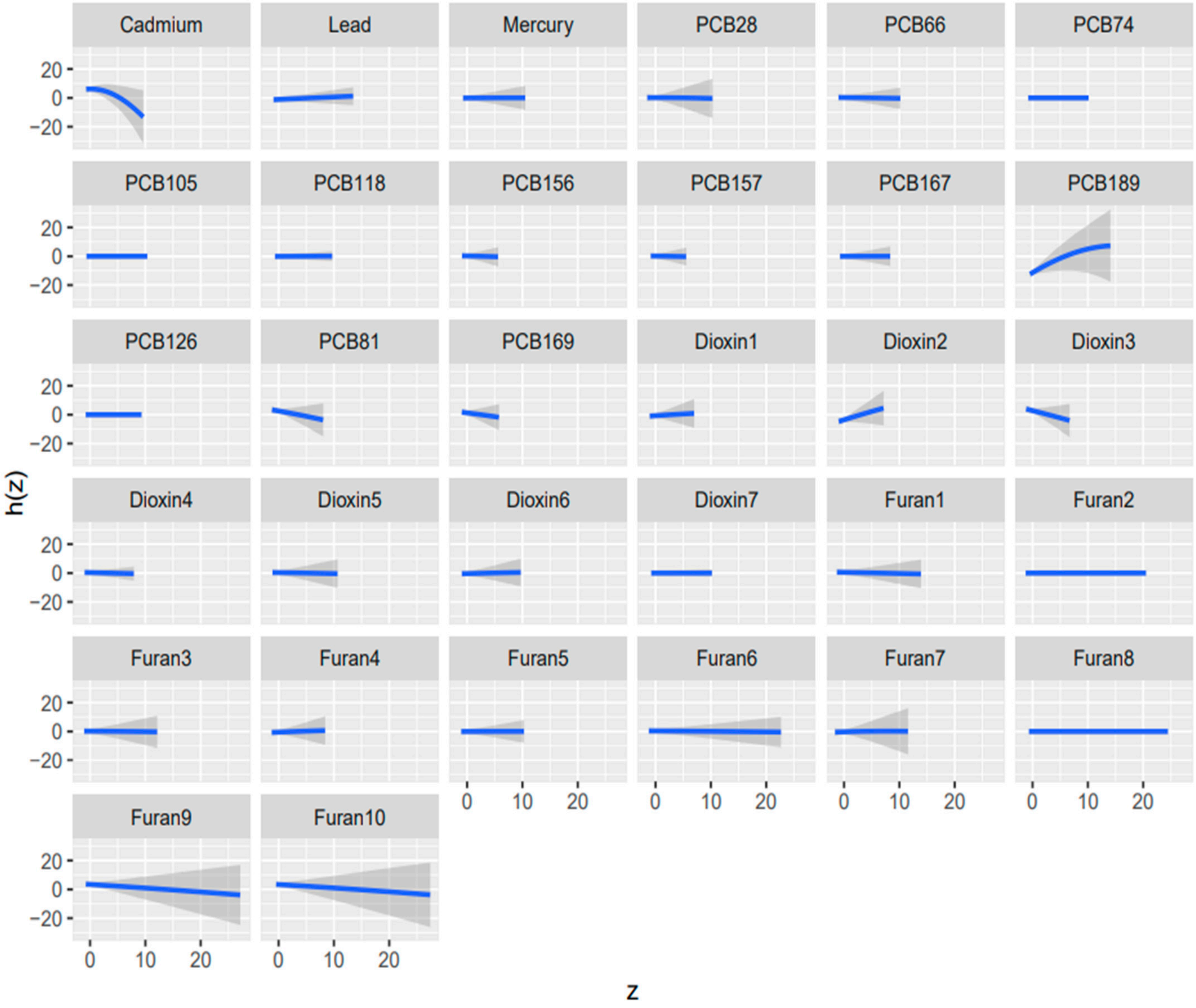

SUPPLEMENTARY DOCUMENTS

S8. TOTAL BILIRUBIN (TB)

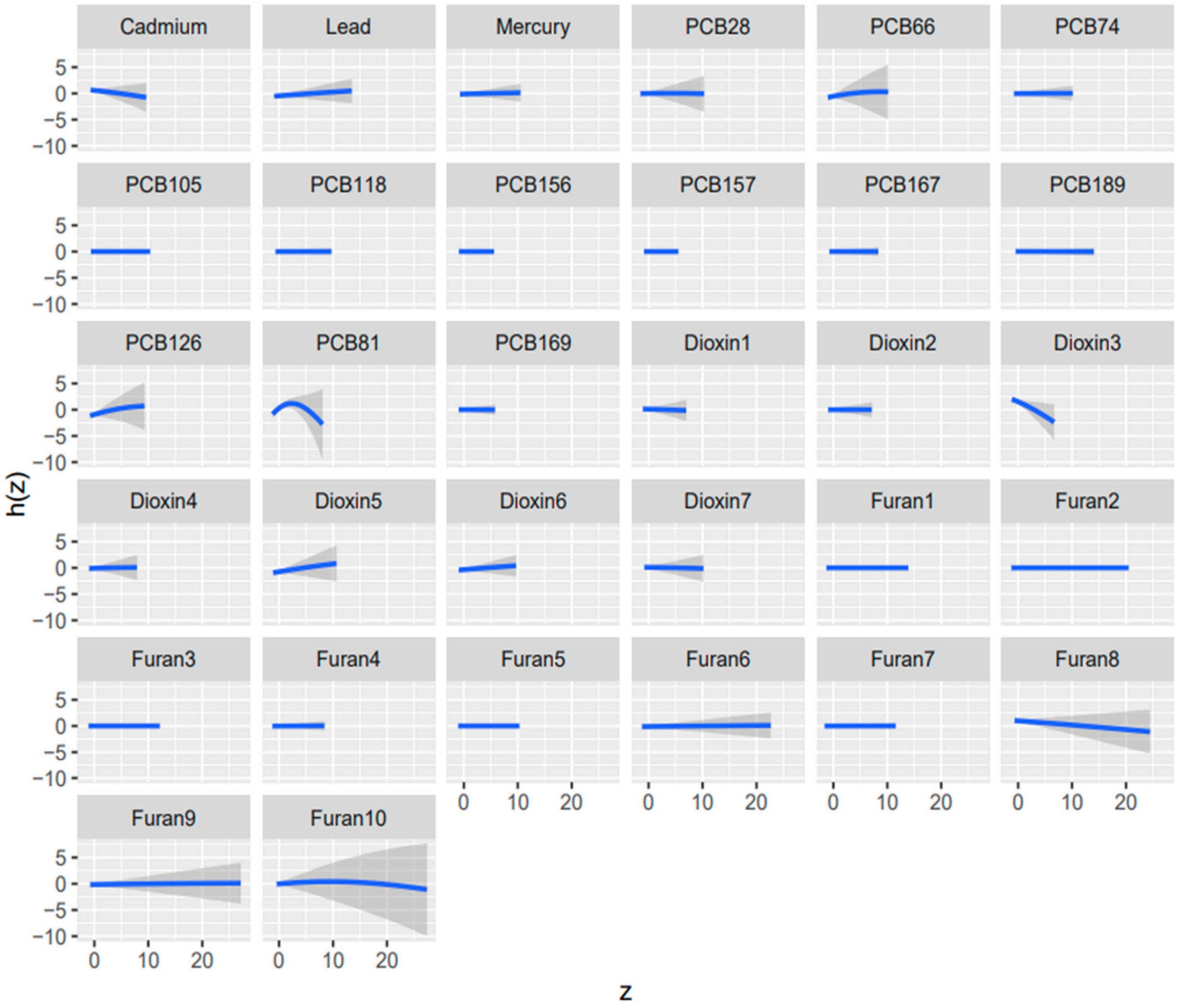

SUPPLEMENTARY DOCUMENTS

S9. TOTAL PROTEIN (TP)

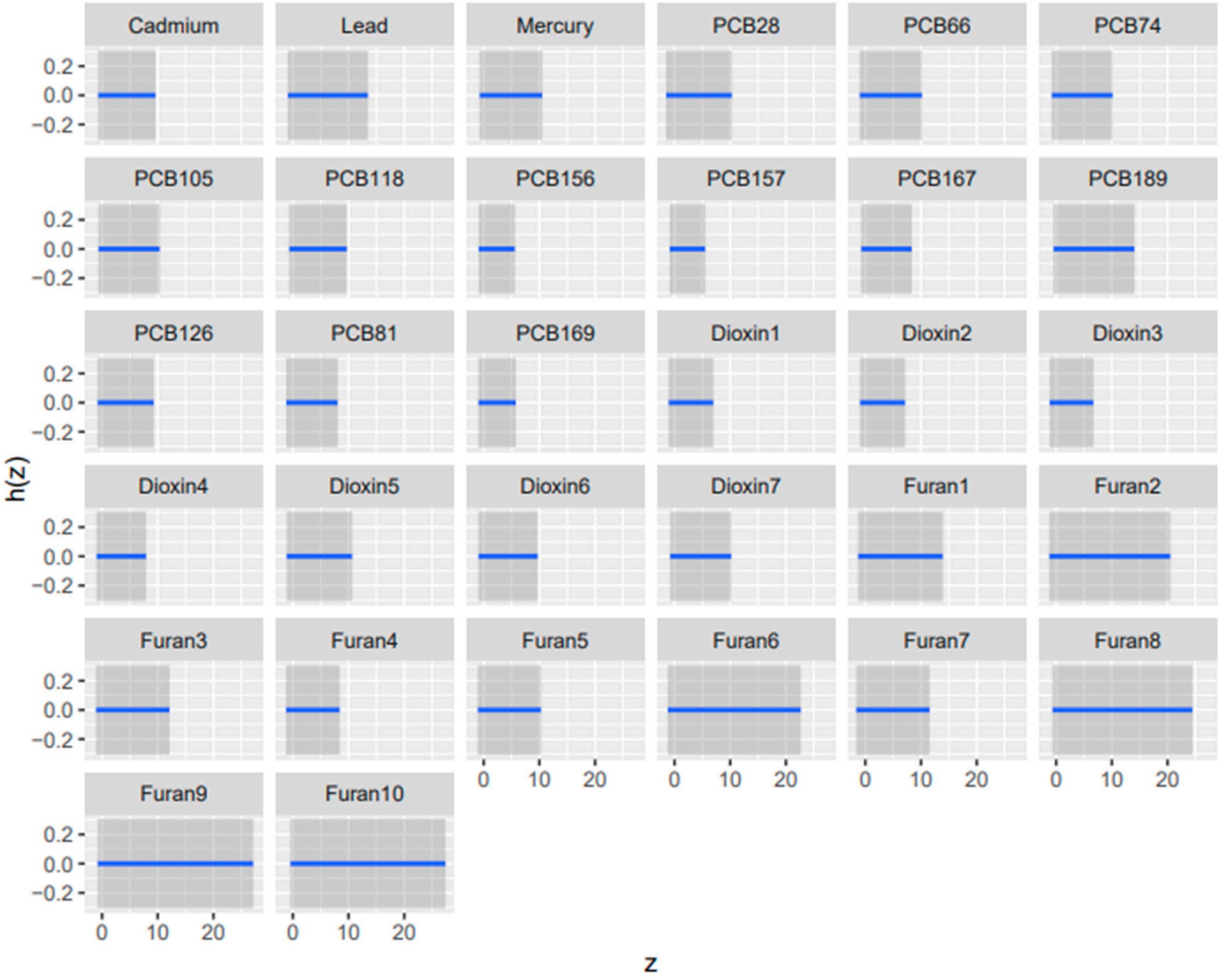

OVERALL EXPOSURE EFFECT SUMMARY

S10. ALBUMIN

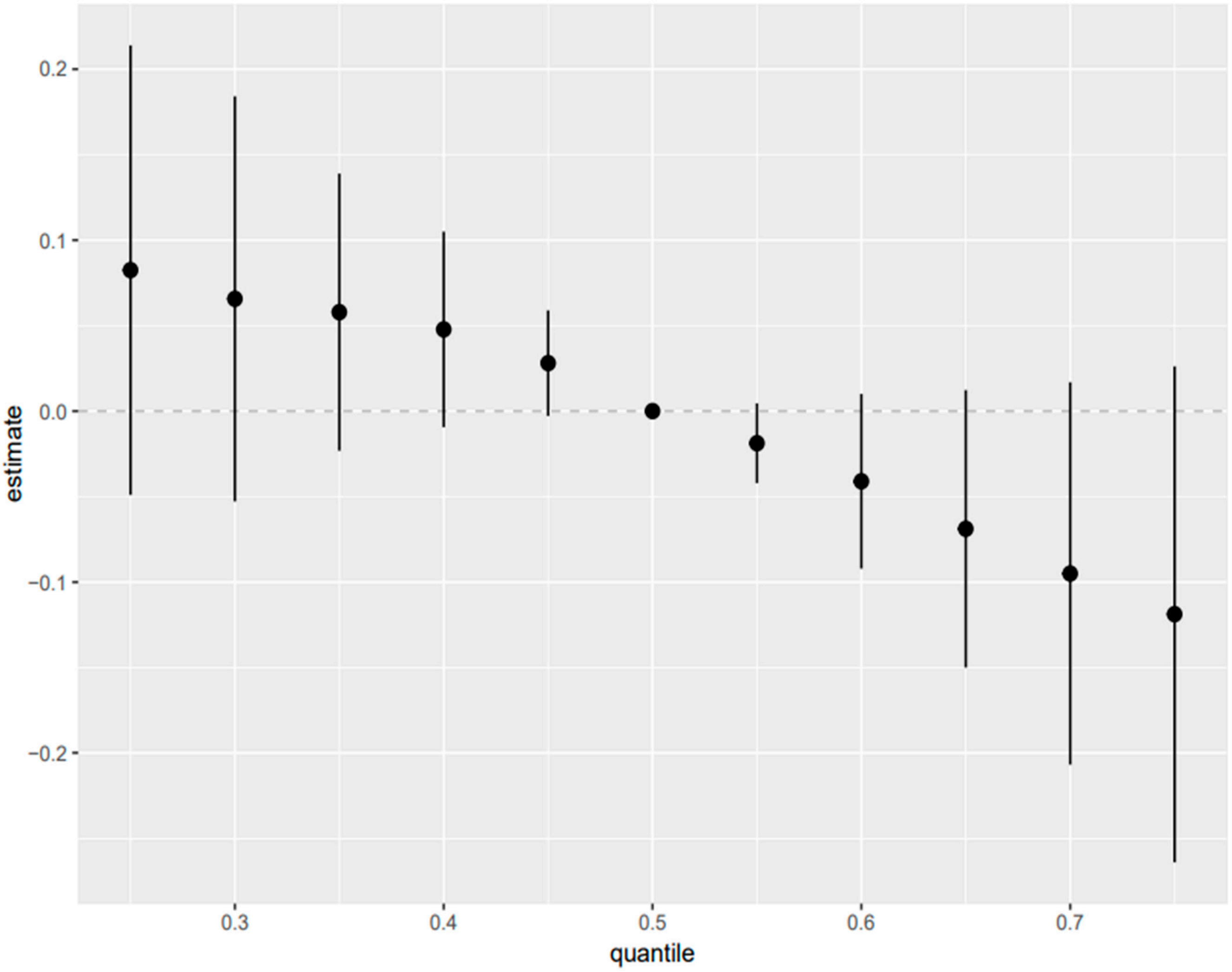

SUPPLEMENTARY DOCUMENTS

S11. ALKALINE PHOSPHATASE (ALP)

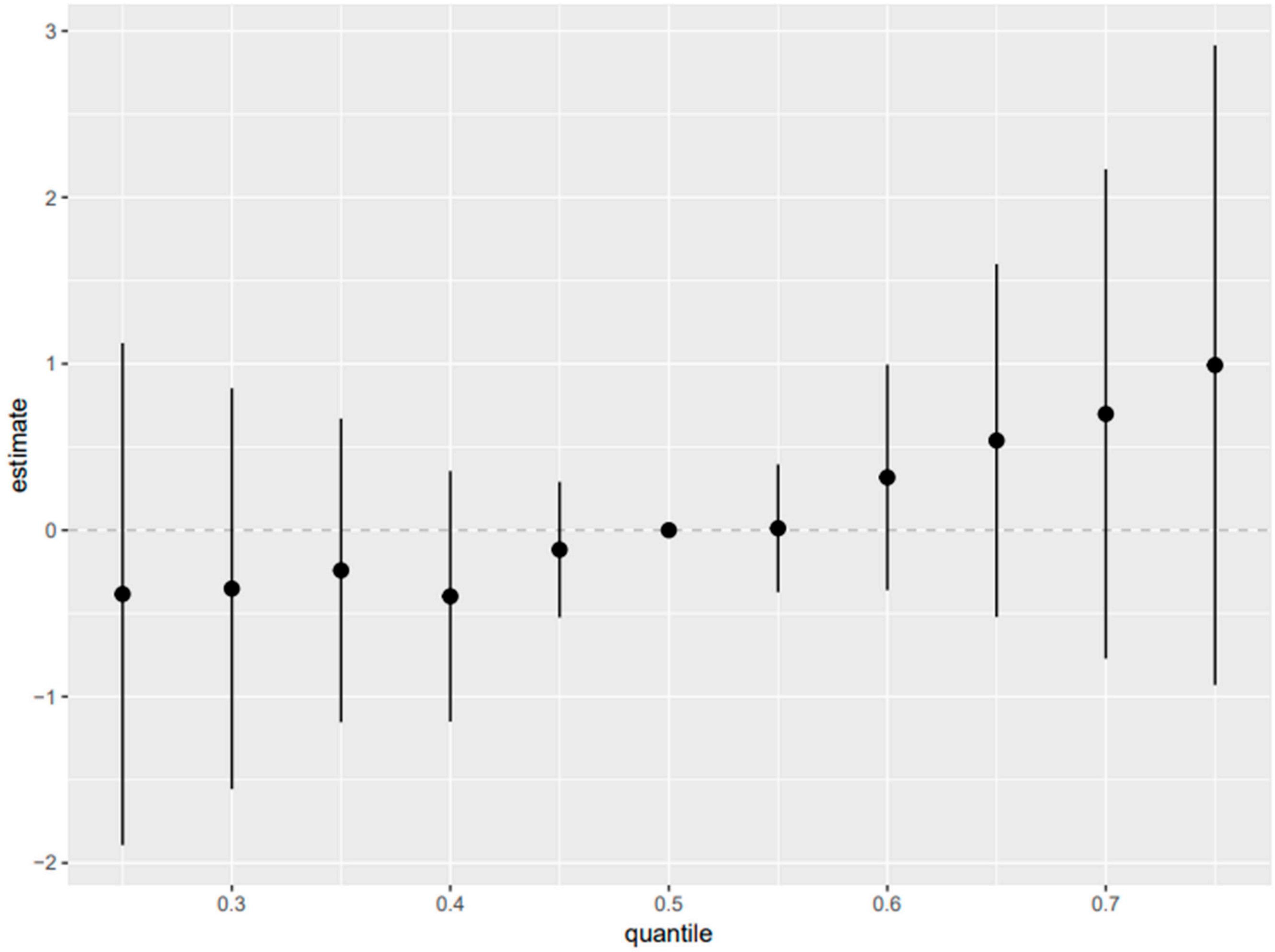

S12. ALANINE AMINOTRANSFERASE (ALT)

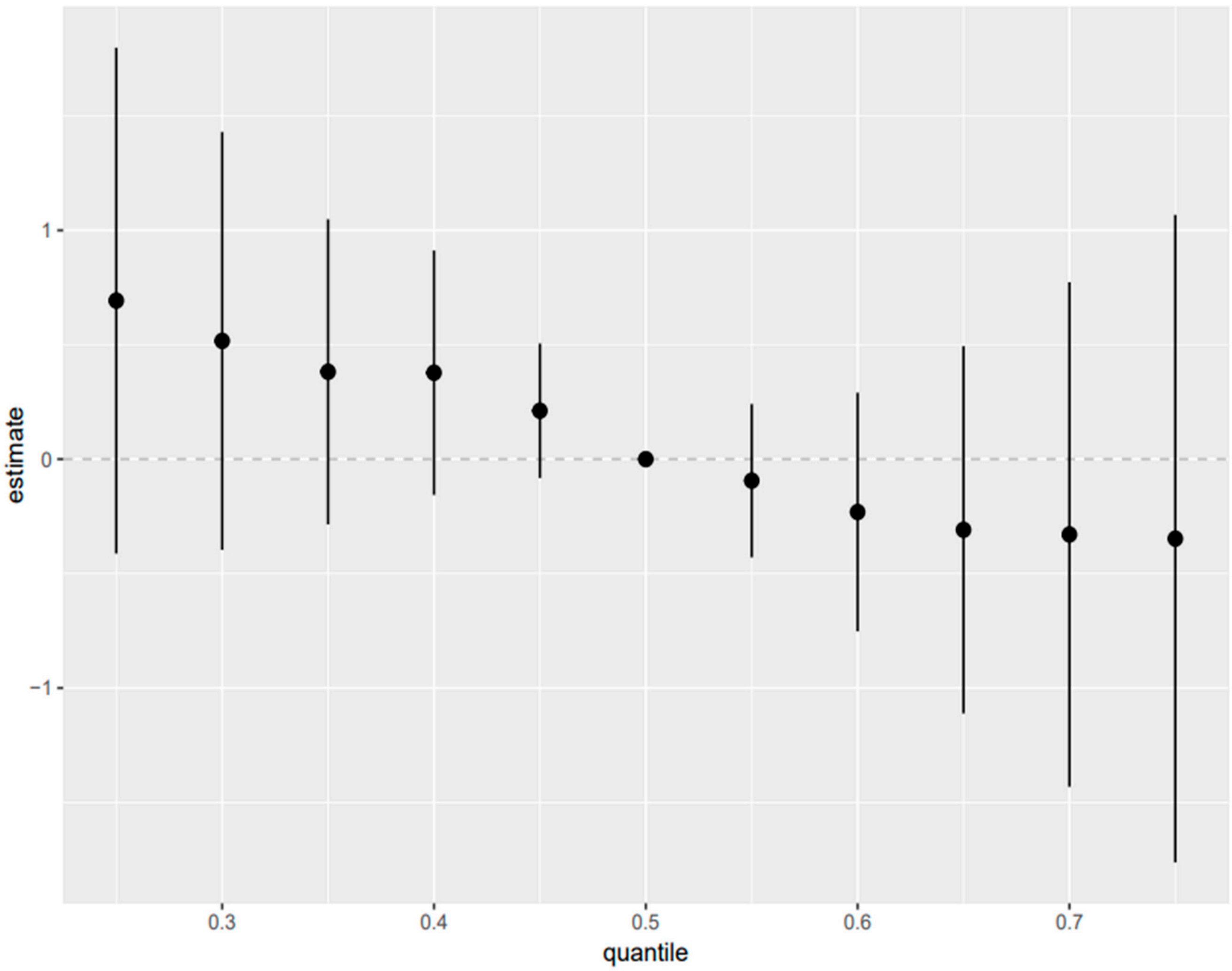

SUPPLEMENTARY DOCUMENTS

S13. ASPARTATE AMINOTRANSFERASE (AST)

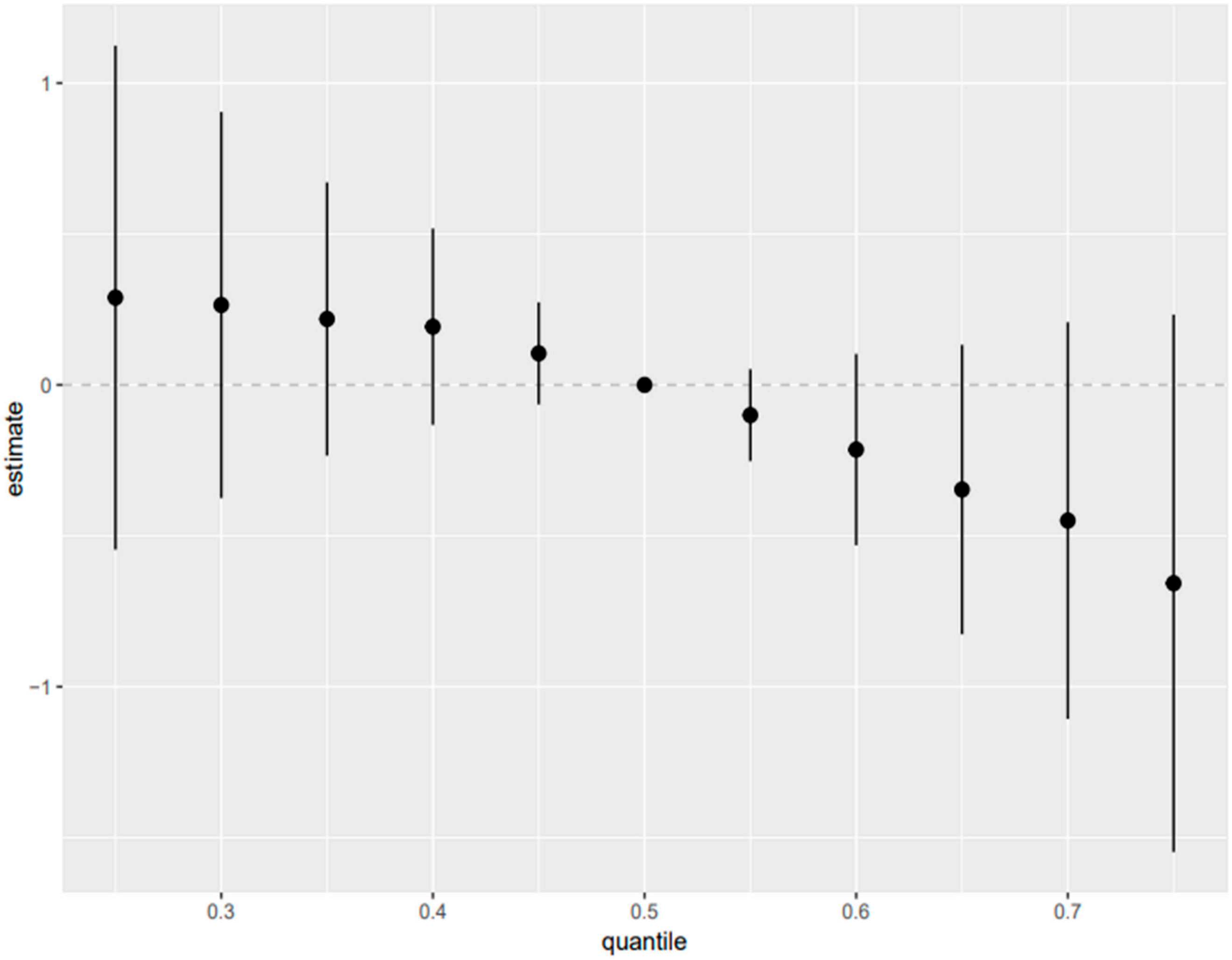

SUPPLEMENTARY DOCUMENTS

S14. GAMMA GLUTAMYL TRANSFERASE (GGT)

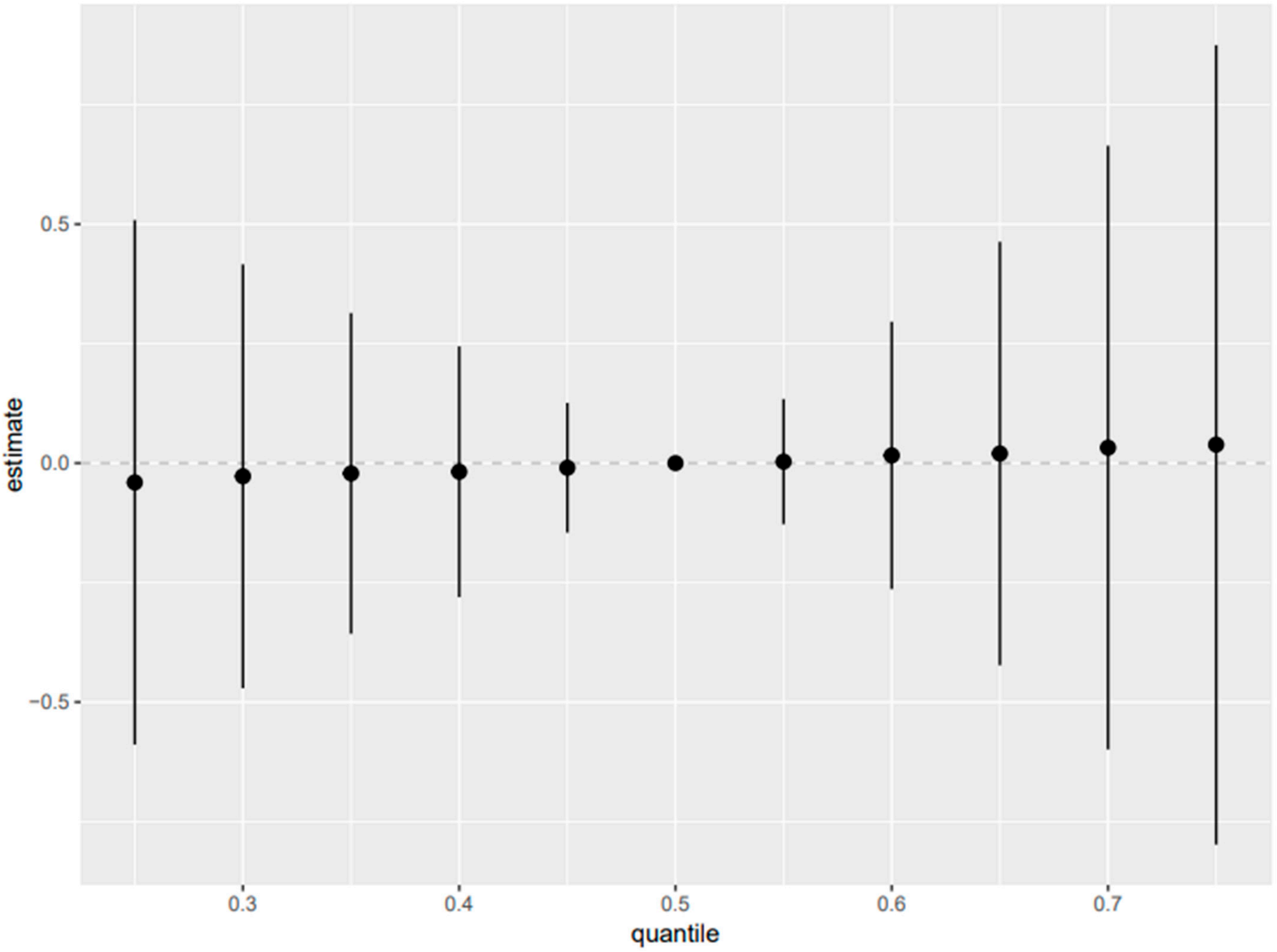

SUPPLEMENTARY DOCUMENTS

S15. LACTATE DEHYDROGENASE (LDH)

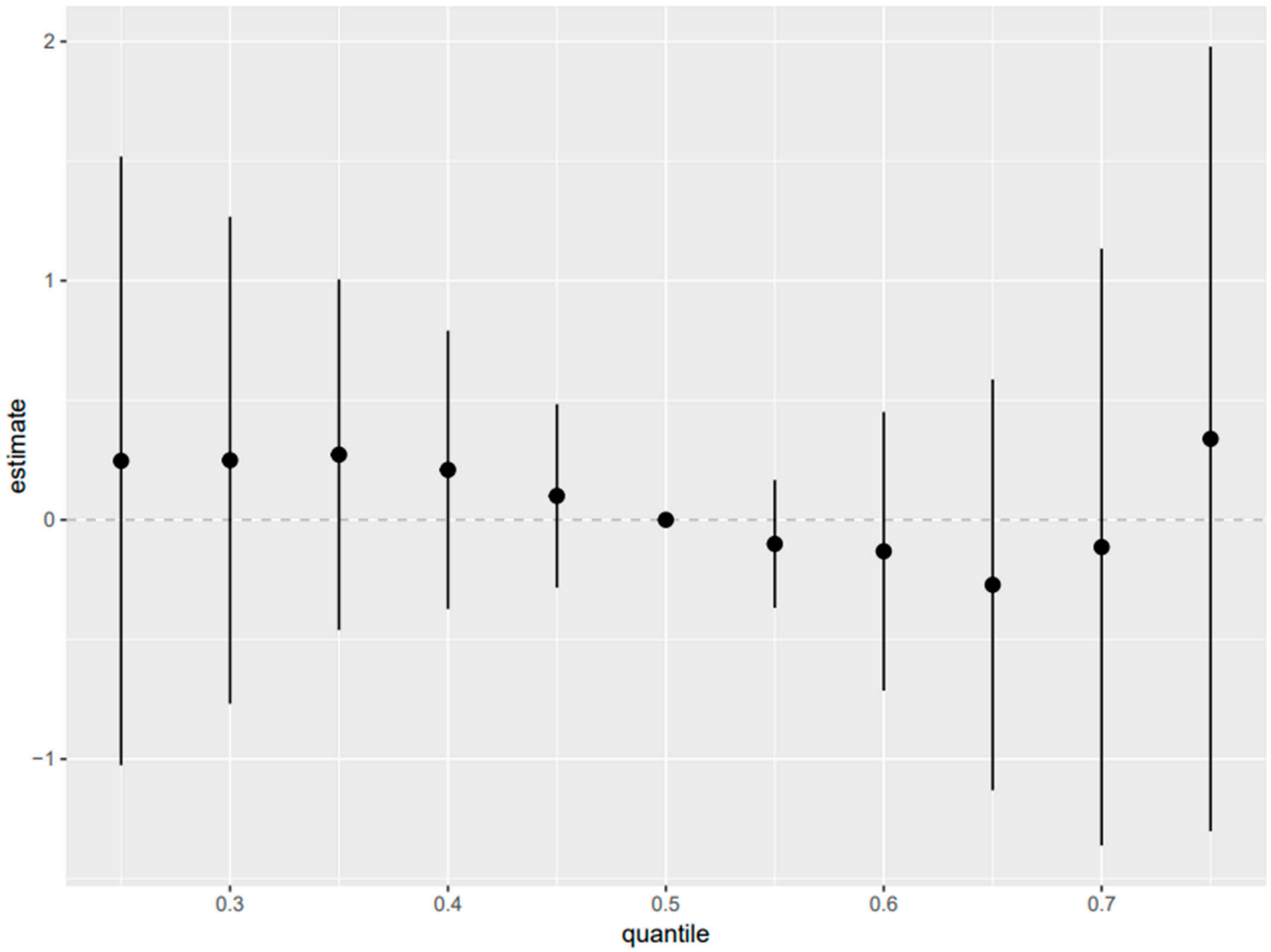

SUPPLEMENTARY DOCUMENTS

S16. TOTAL BILIRUBIN

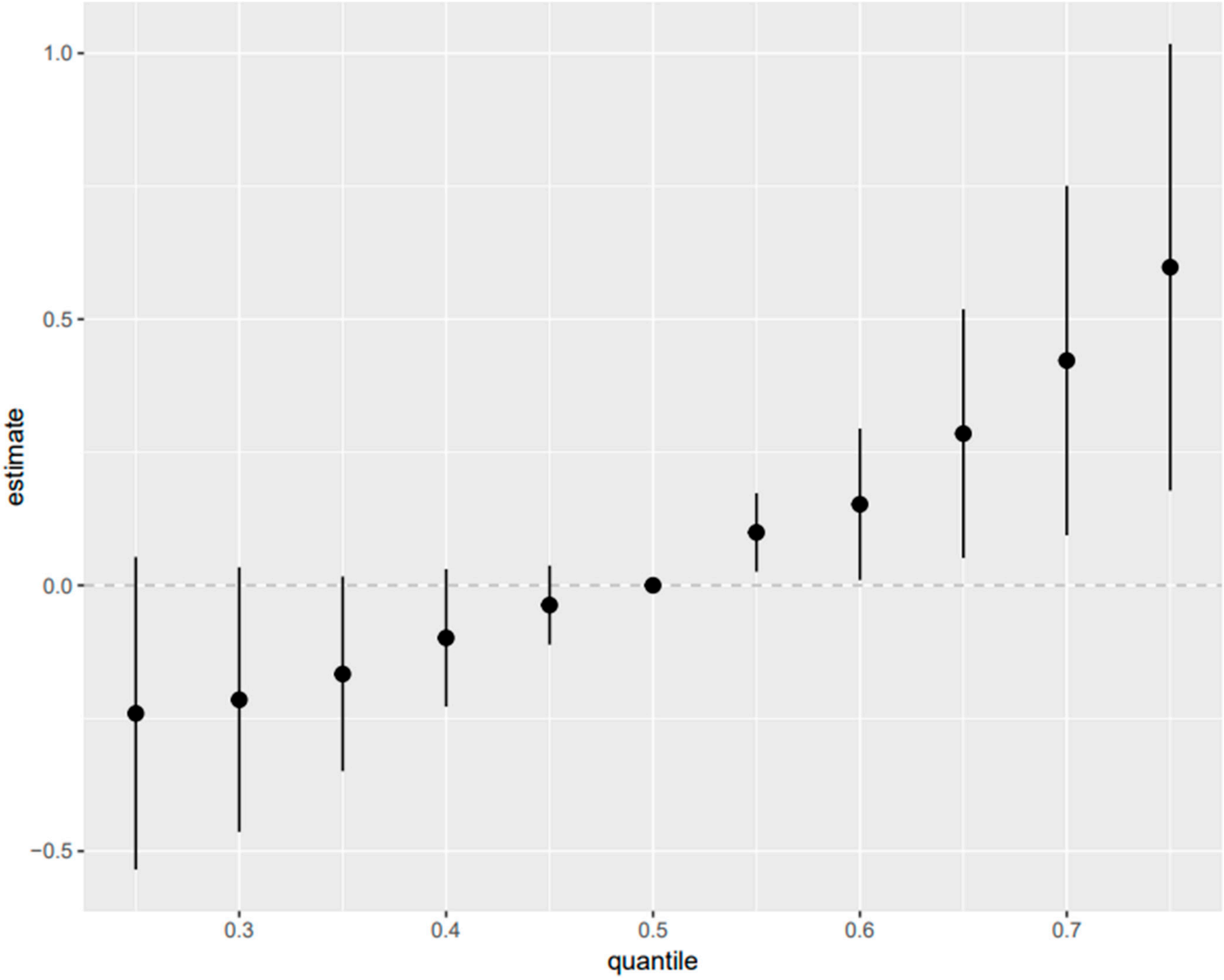

SUPPLEMENTARY DOCUMENTS

S17. TOTAL PROTEIN

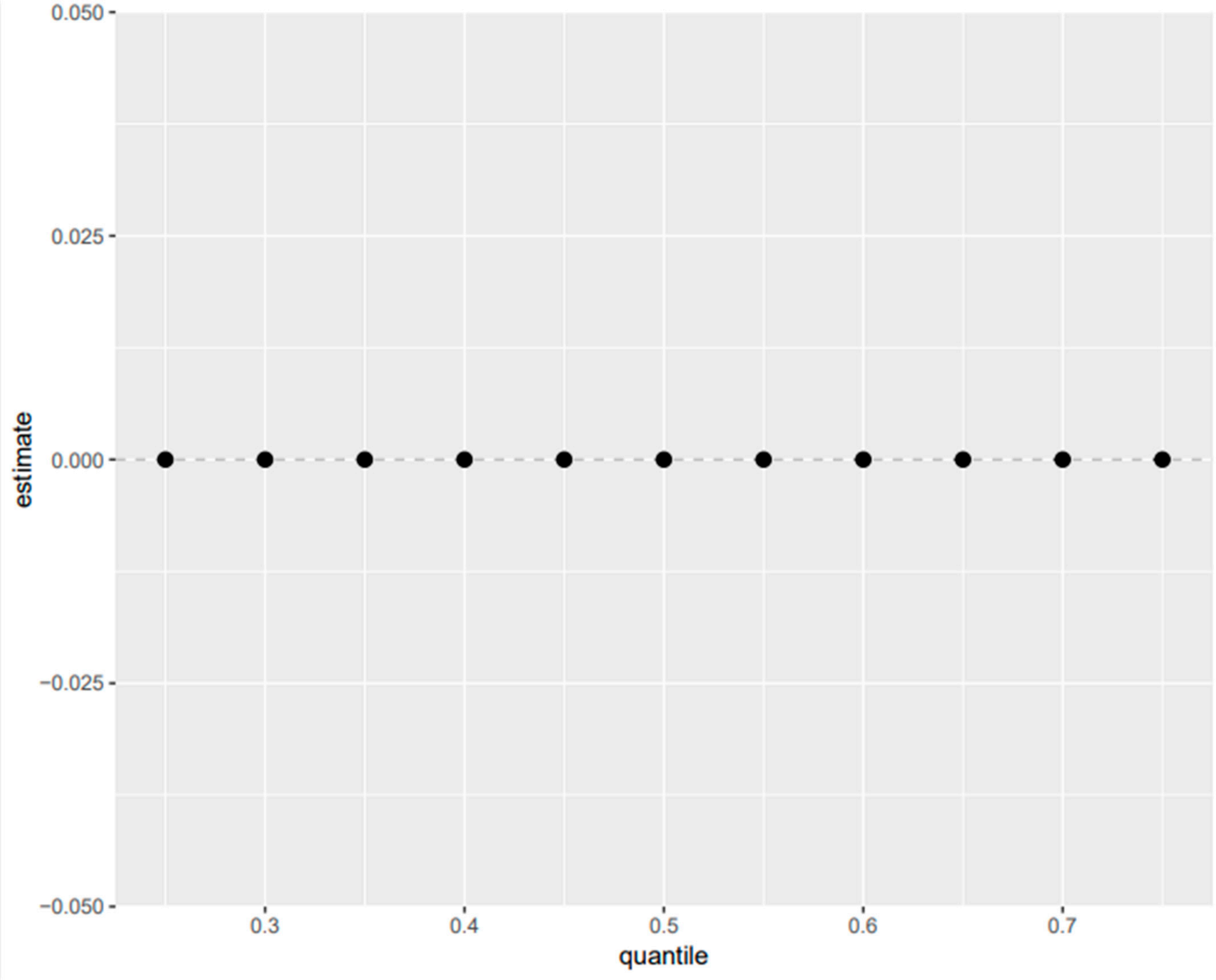

SINGLE VARIABLE EFFECTS

S18. ALBUMIN

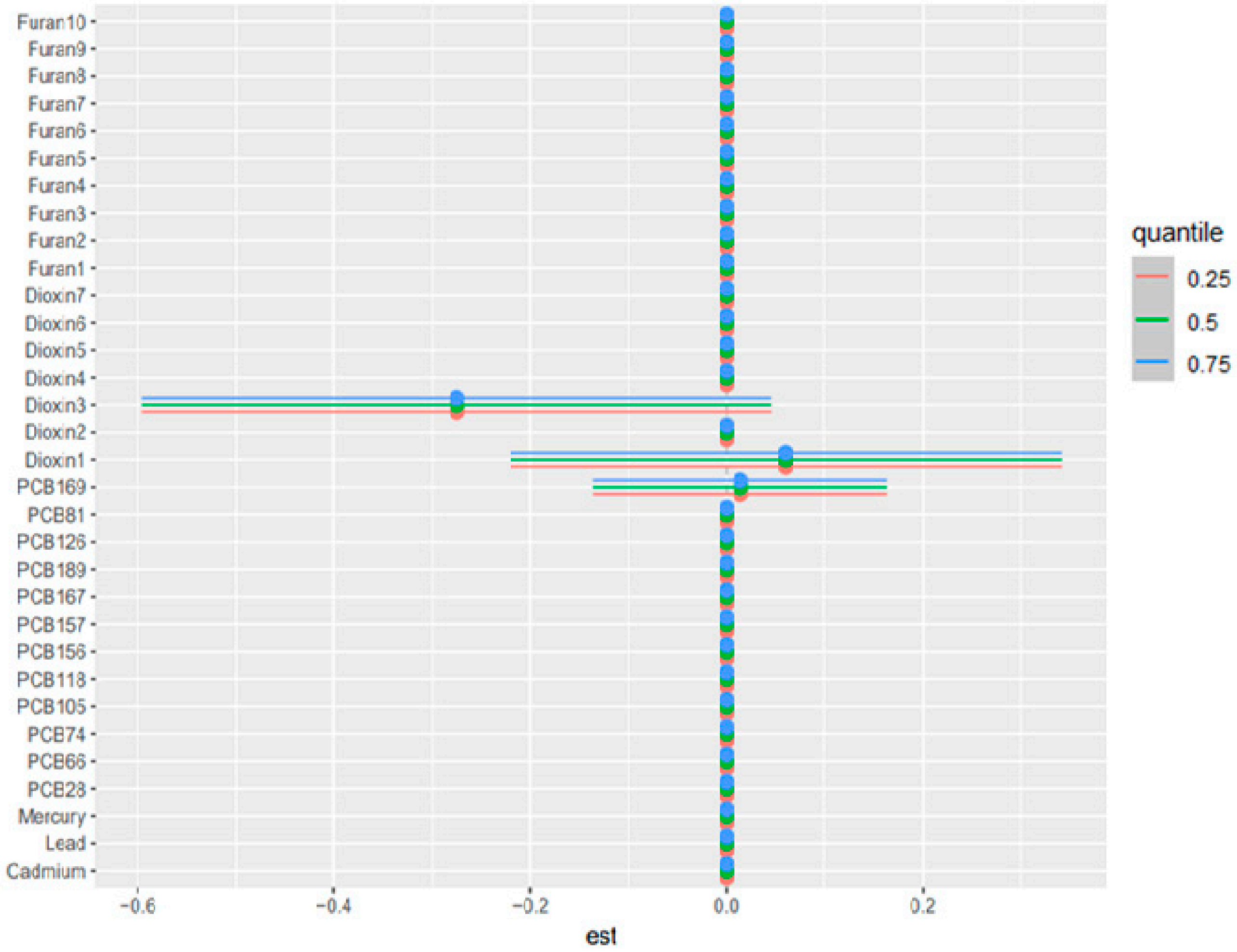

SUPPLEMENTARY DOCUMENTS

S19. ALKALINE PHOSPHATASE (ALP)

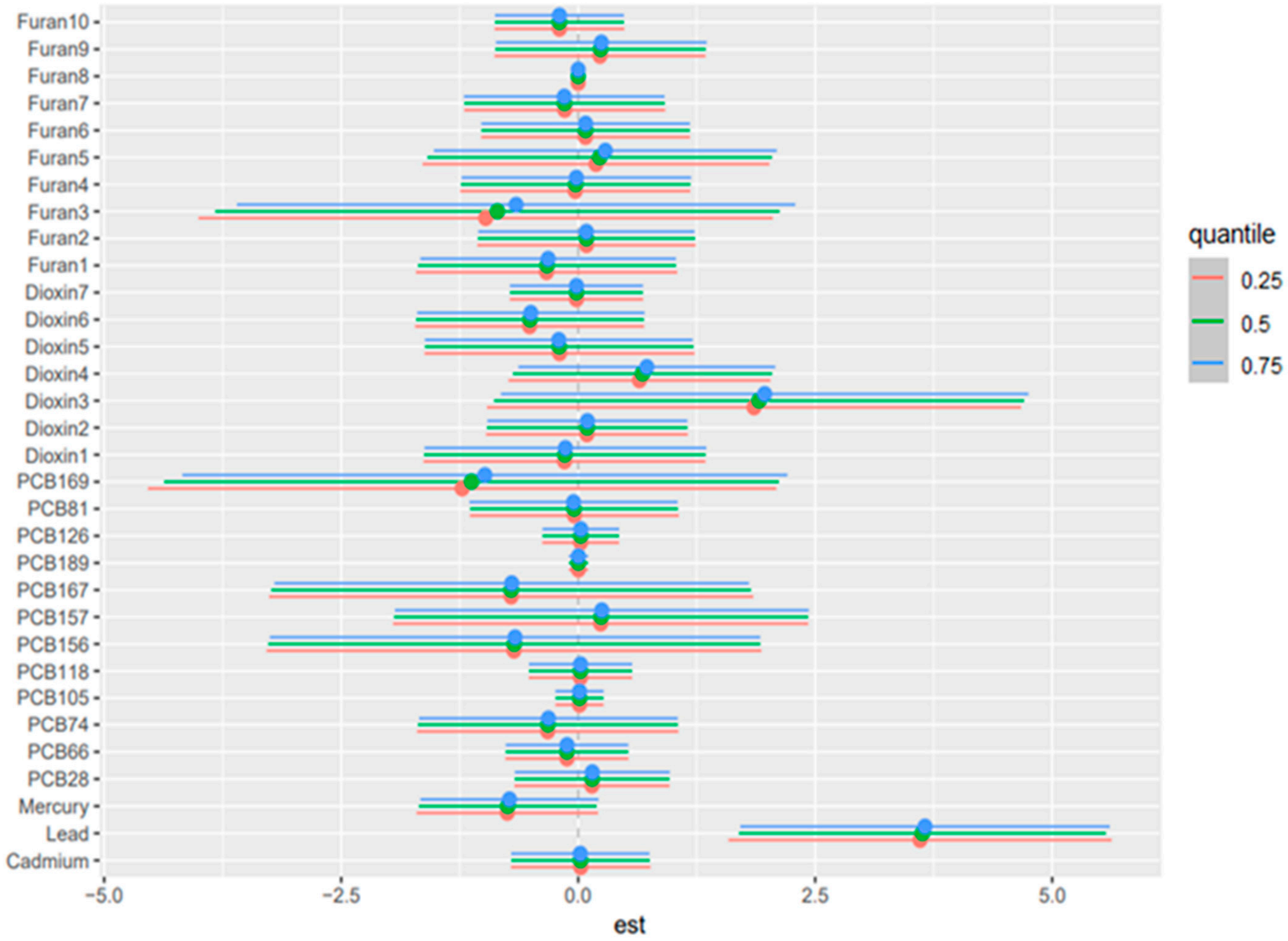

S20. ALANINE AMINOTRANSFERASE (ALT)

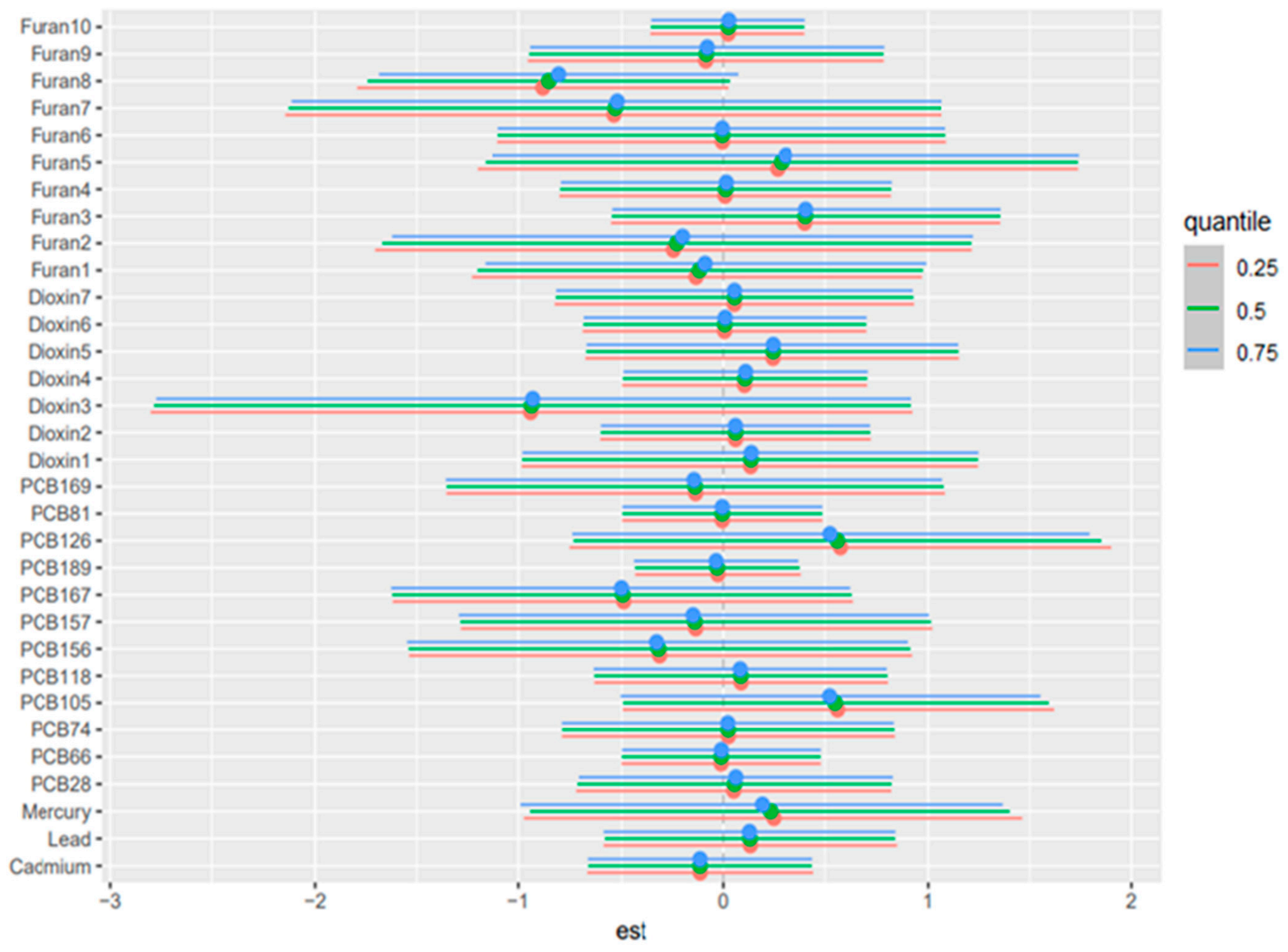

S21. ASPARTATE AMINOTRANSFERASE (AST)

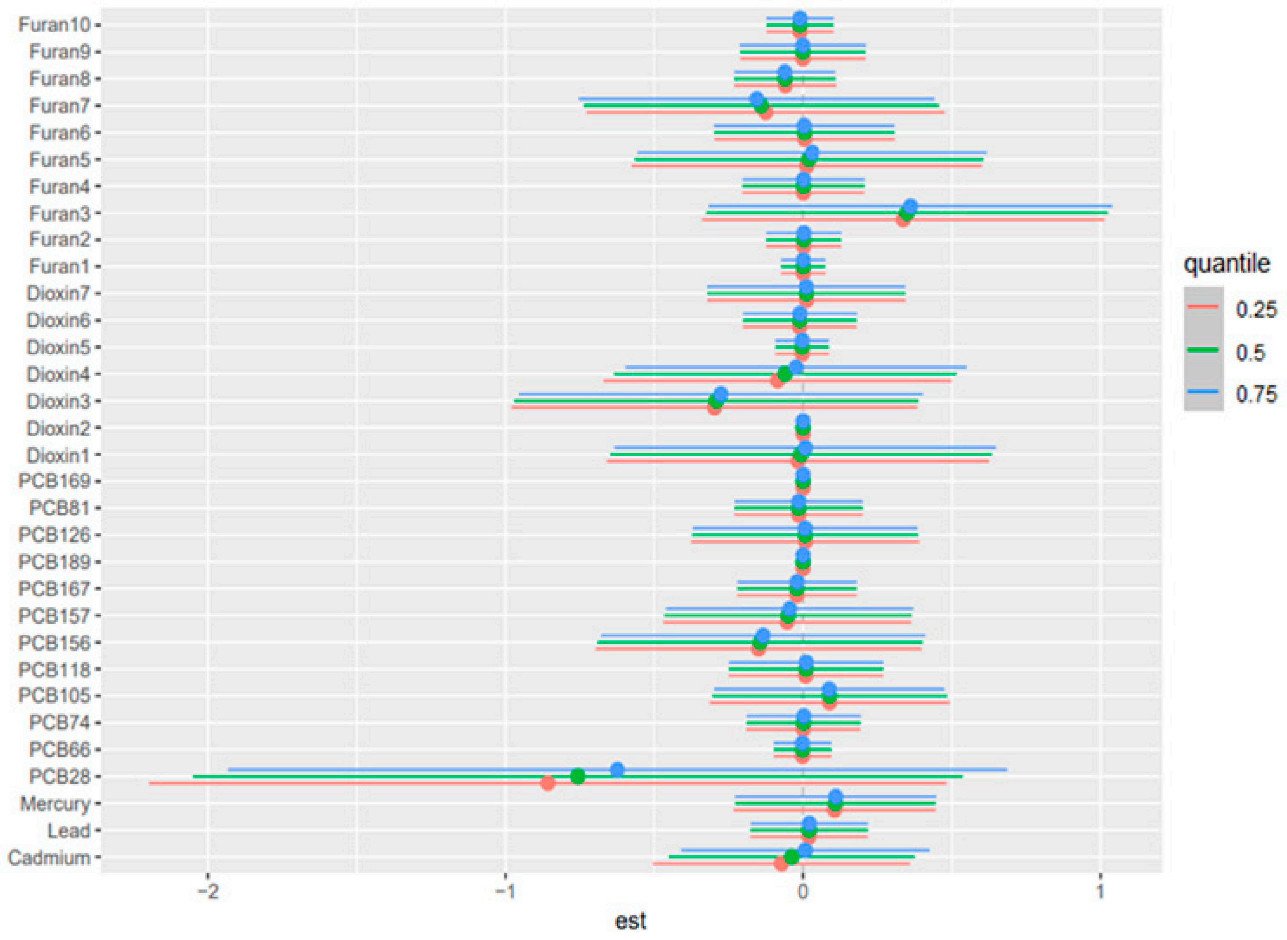

SUPPLEMENTARY DOCUMENTS

S22. GAMMA GLUTAMYL TRANSFERASE (GGT)

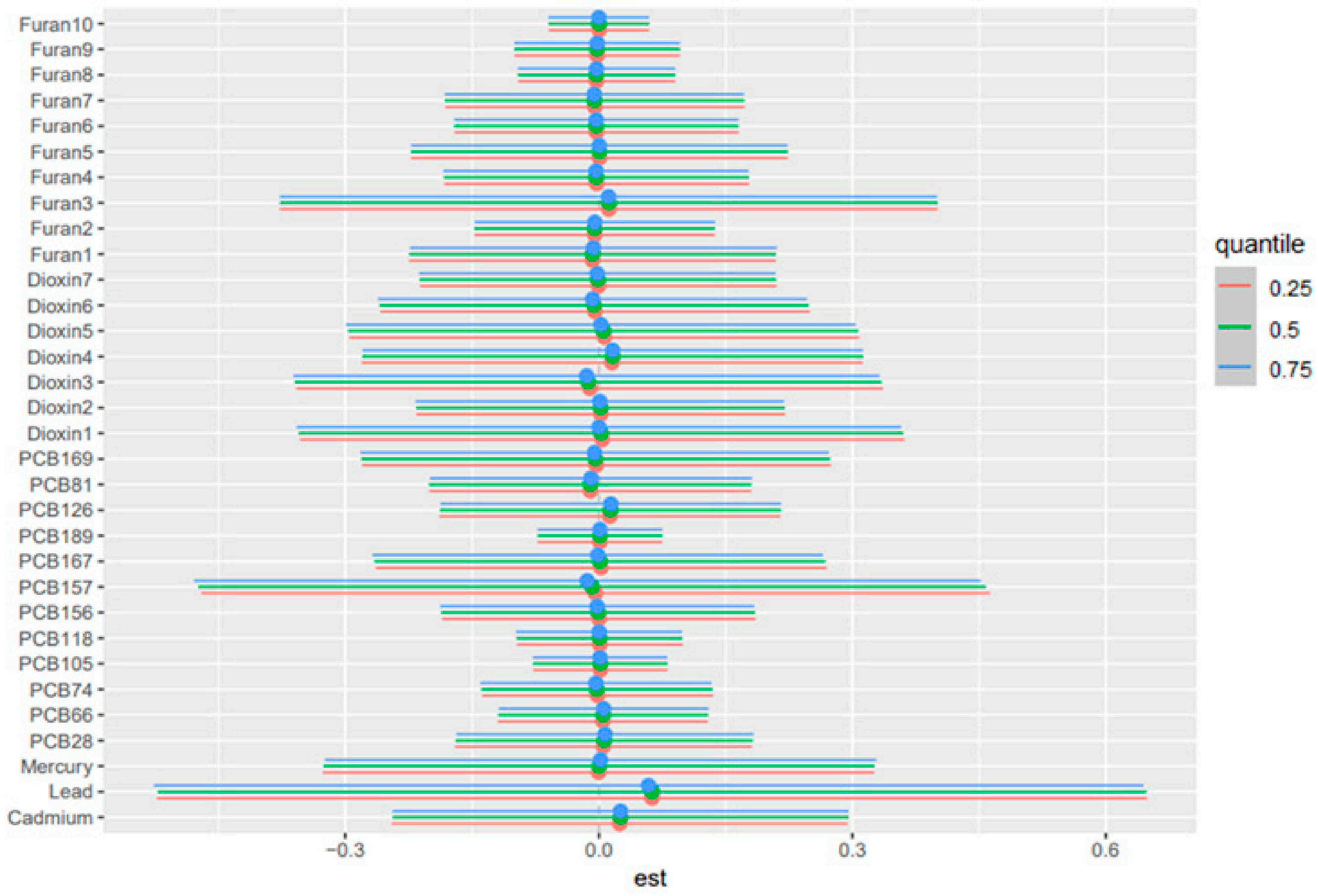

SUPPLEMENTARY DOCUMENTS

S23. LACTATE DEHYDROGENASE (LDH)

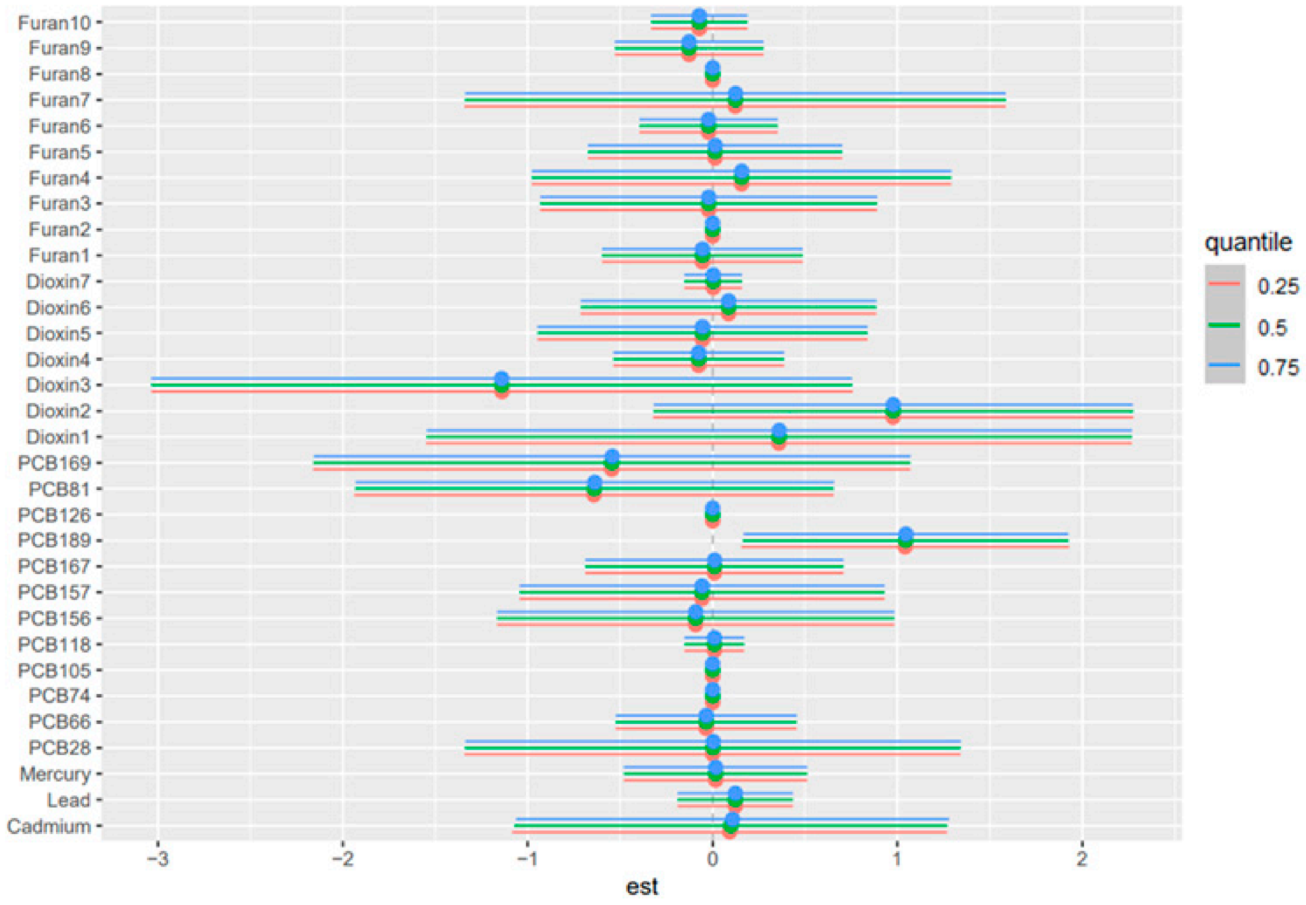

SUPPLEMENTARY DOCUMENTS

S24. TOTAL BILIRUBIN

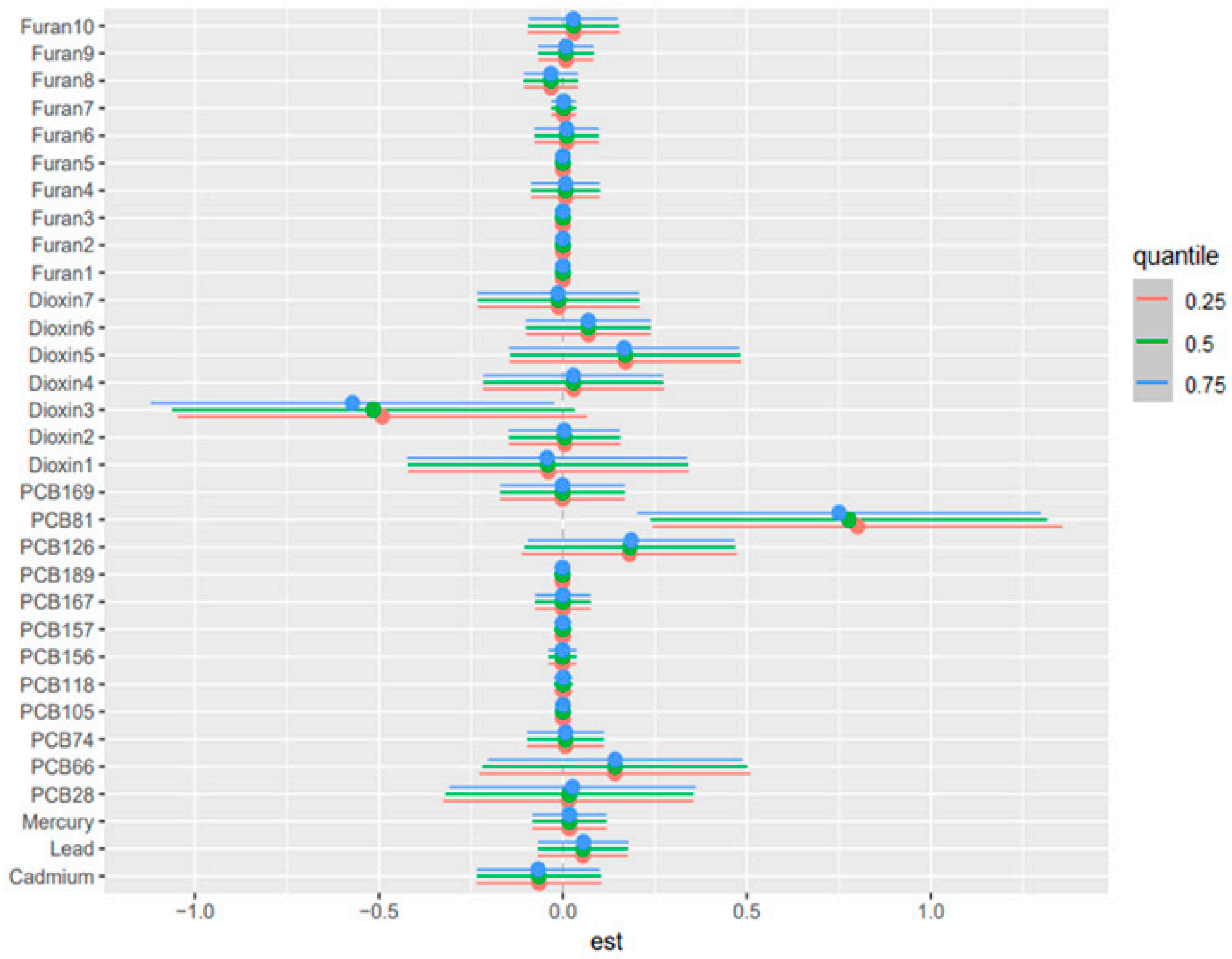

SUPPLEMENTARY DOCUMENTS

S25. TOTAL PROTEIN

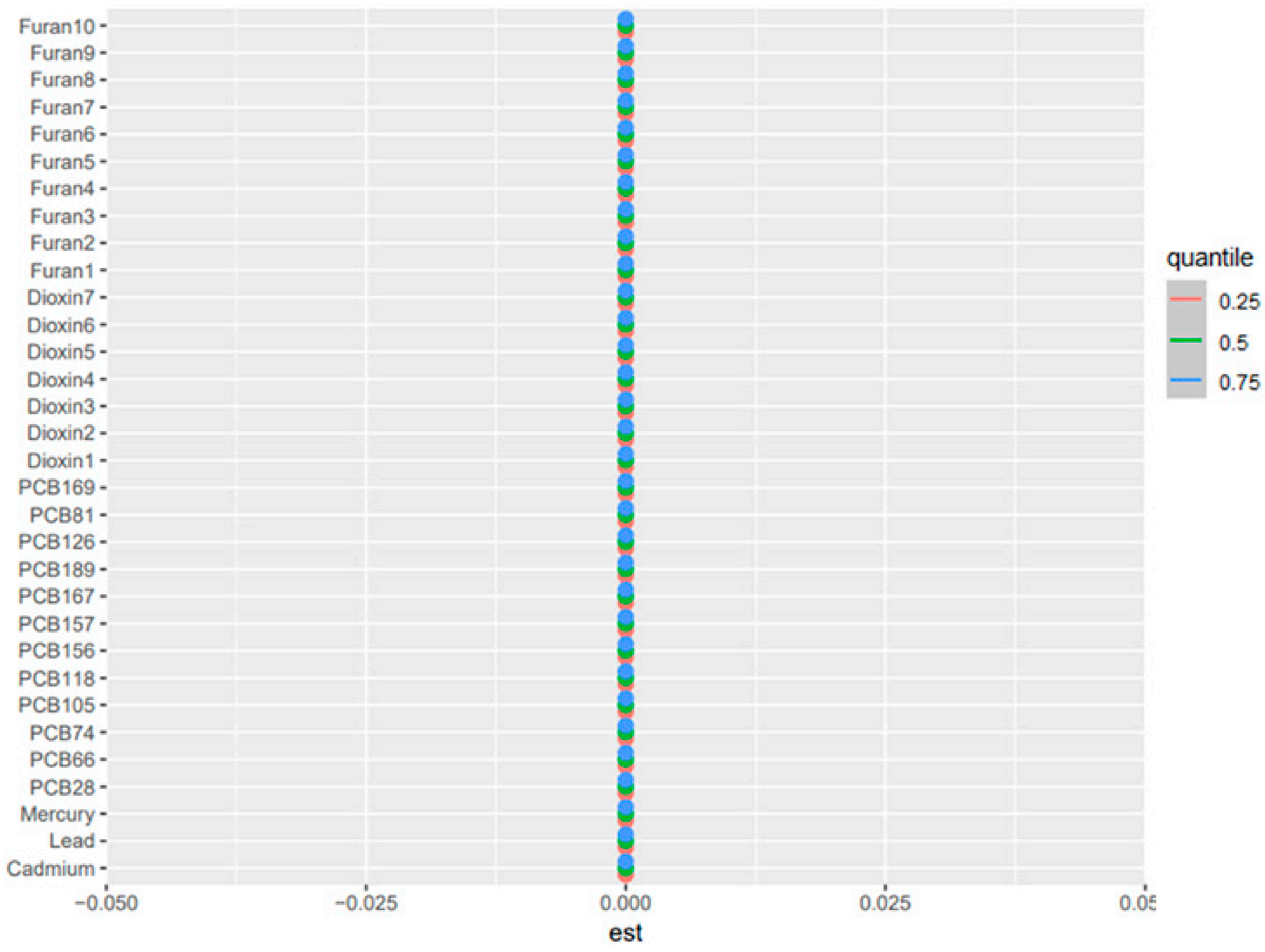

SUPPLEMENTARY DOCUMENTS

SINGLE VARIABLE INTERACTION

S26. ALBUMIN

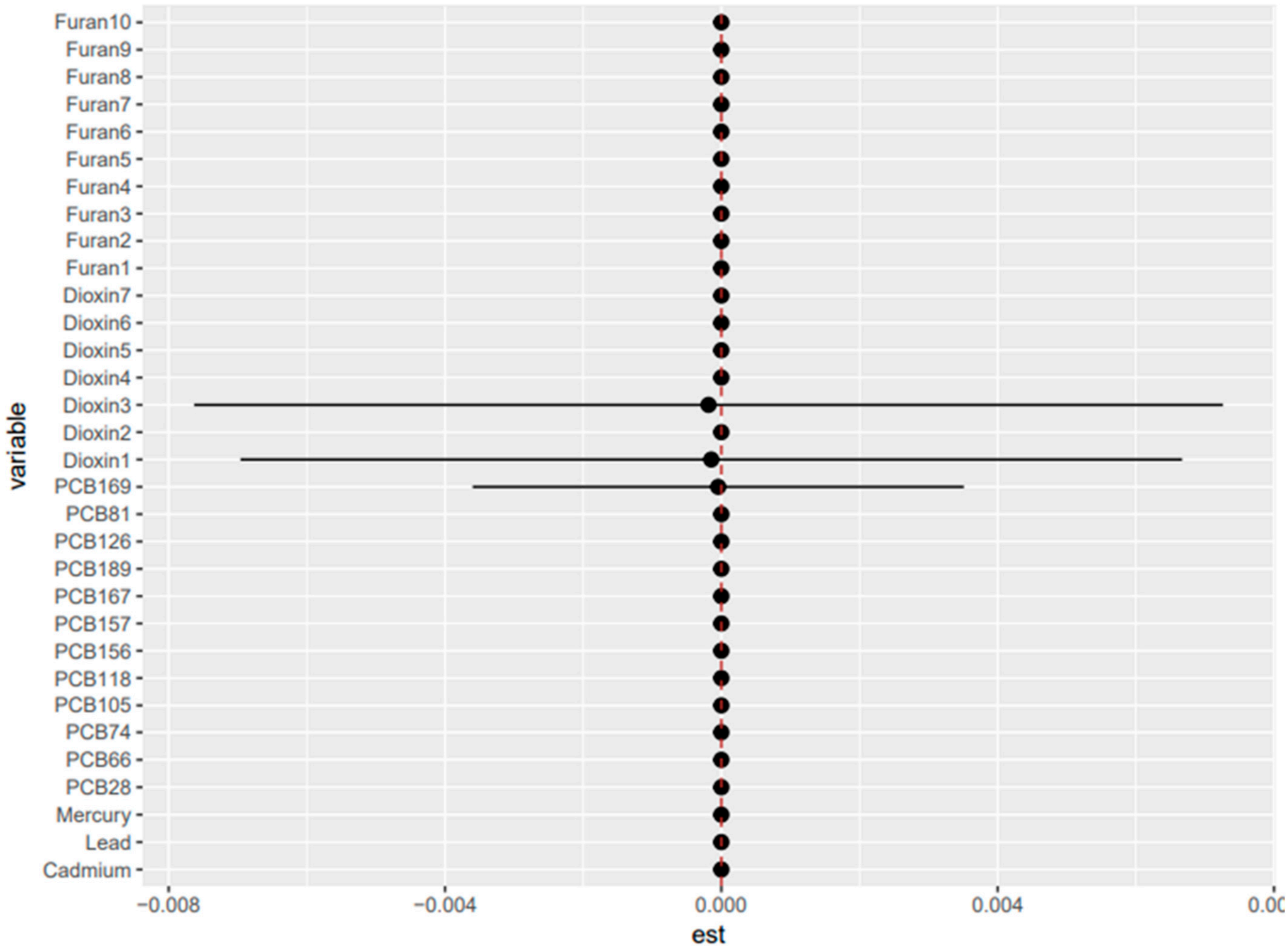

SUPPLEMENTARY DOCUMENTS

S27. ALKALINE PHOSPHATASE (ALP)

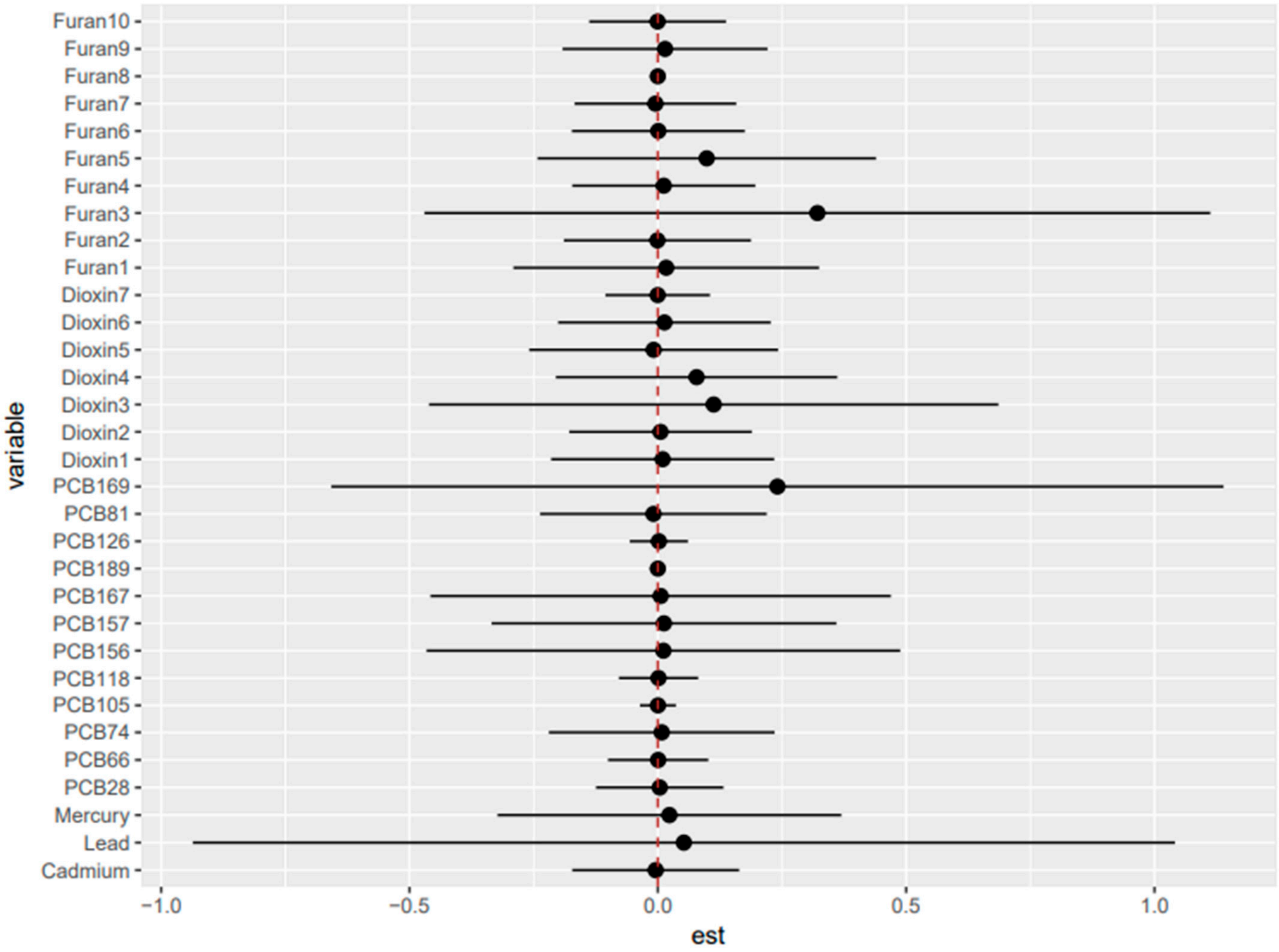

SUPPLEMENTARY DOCUMENTS

S28. ALANINE AMINOTRANSFERASE (ALT)

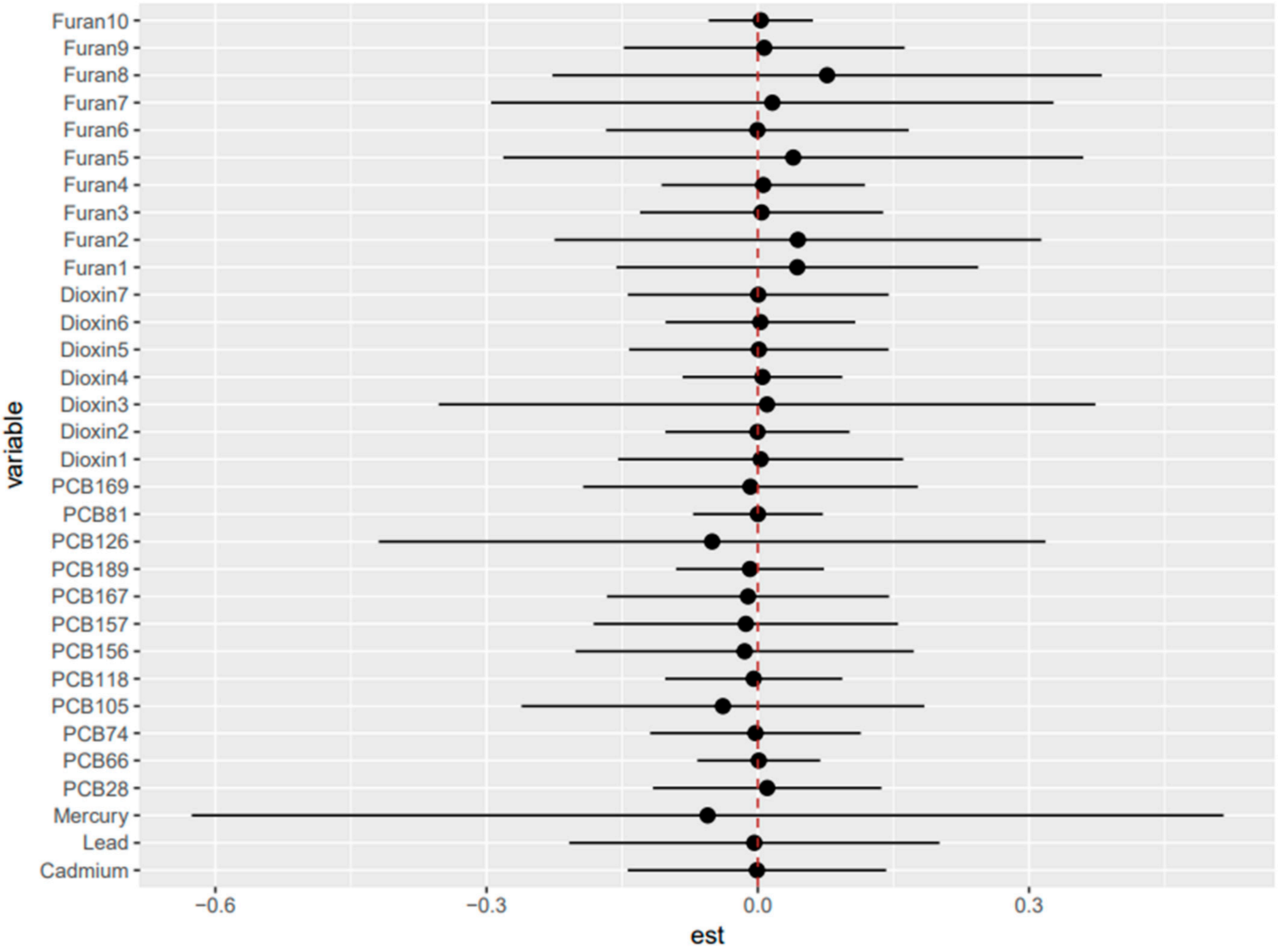

S29. ASPARTATE AMINOTRANSFERASE (AST)

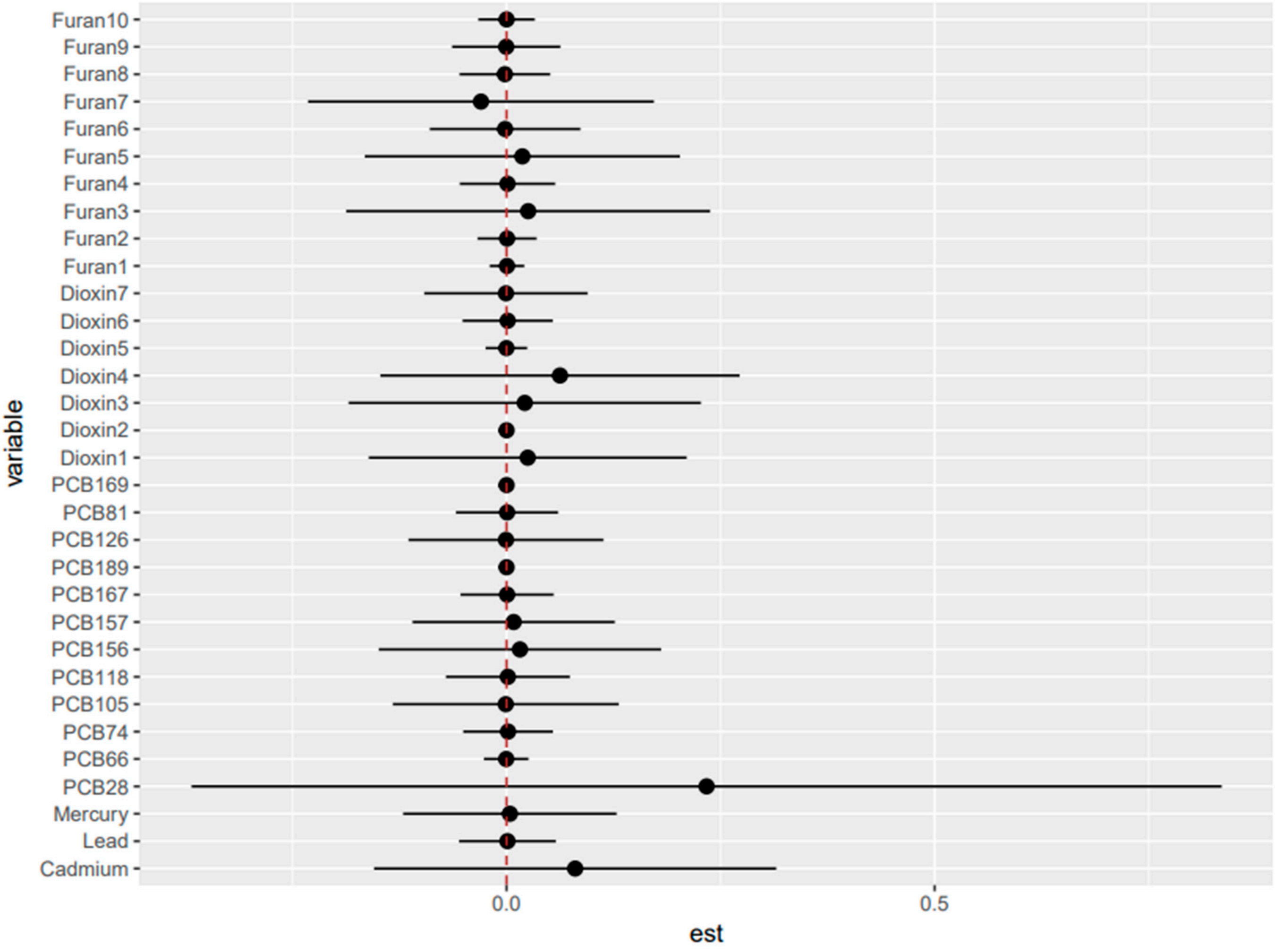

SUPPLEMENTARY DOCUMENTS

S30. GAMMA GLUTAMYL TRANSFERASE (GGT)

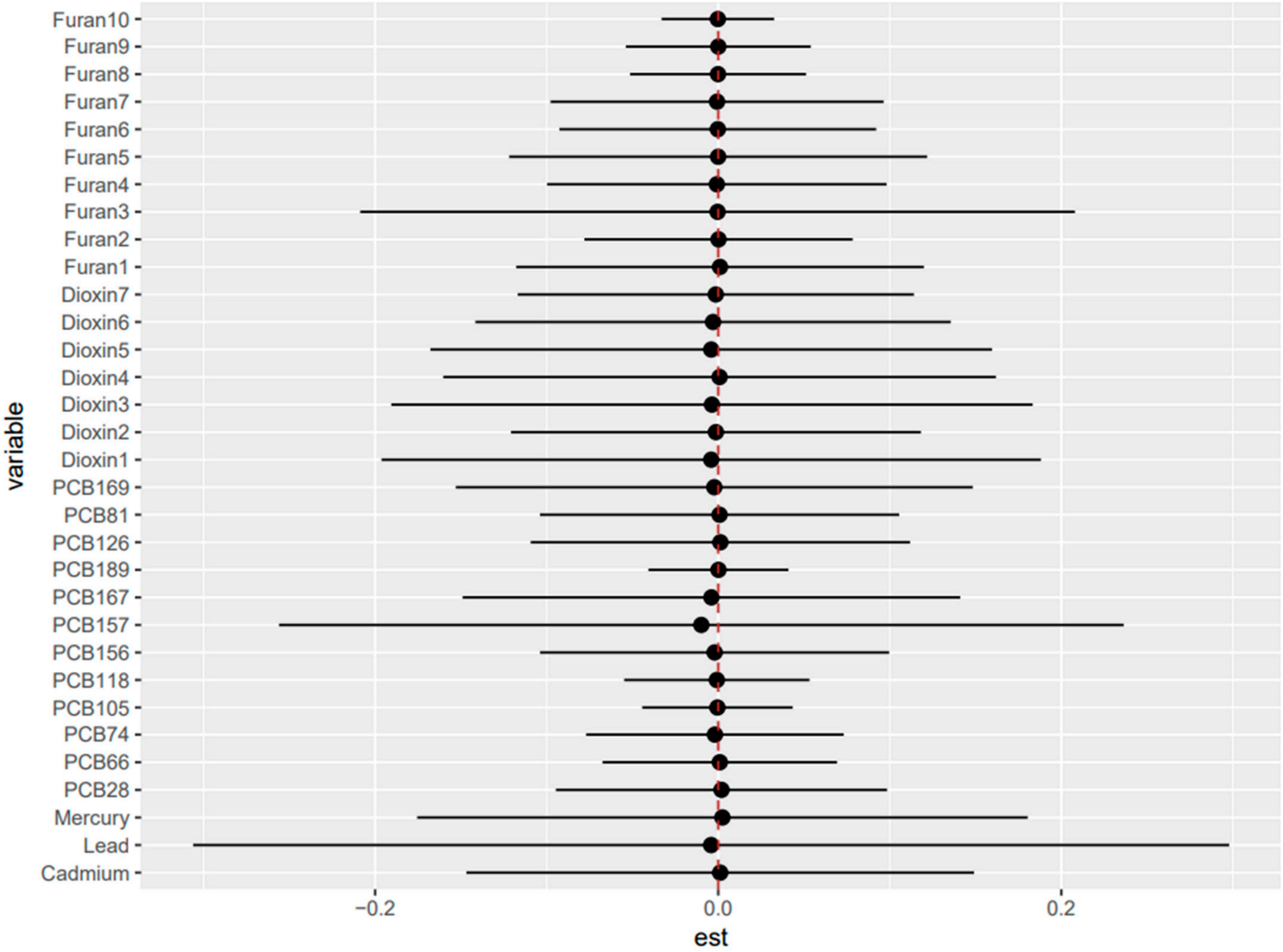

SUPPLEMENTARY DOCUMENTS

S31. LACTATE DEHYDROGENASE (LDH)

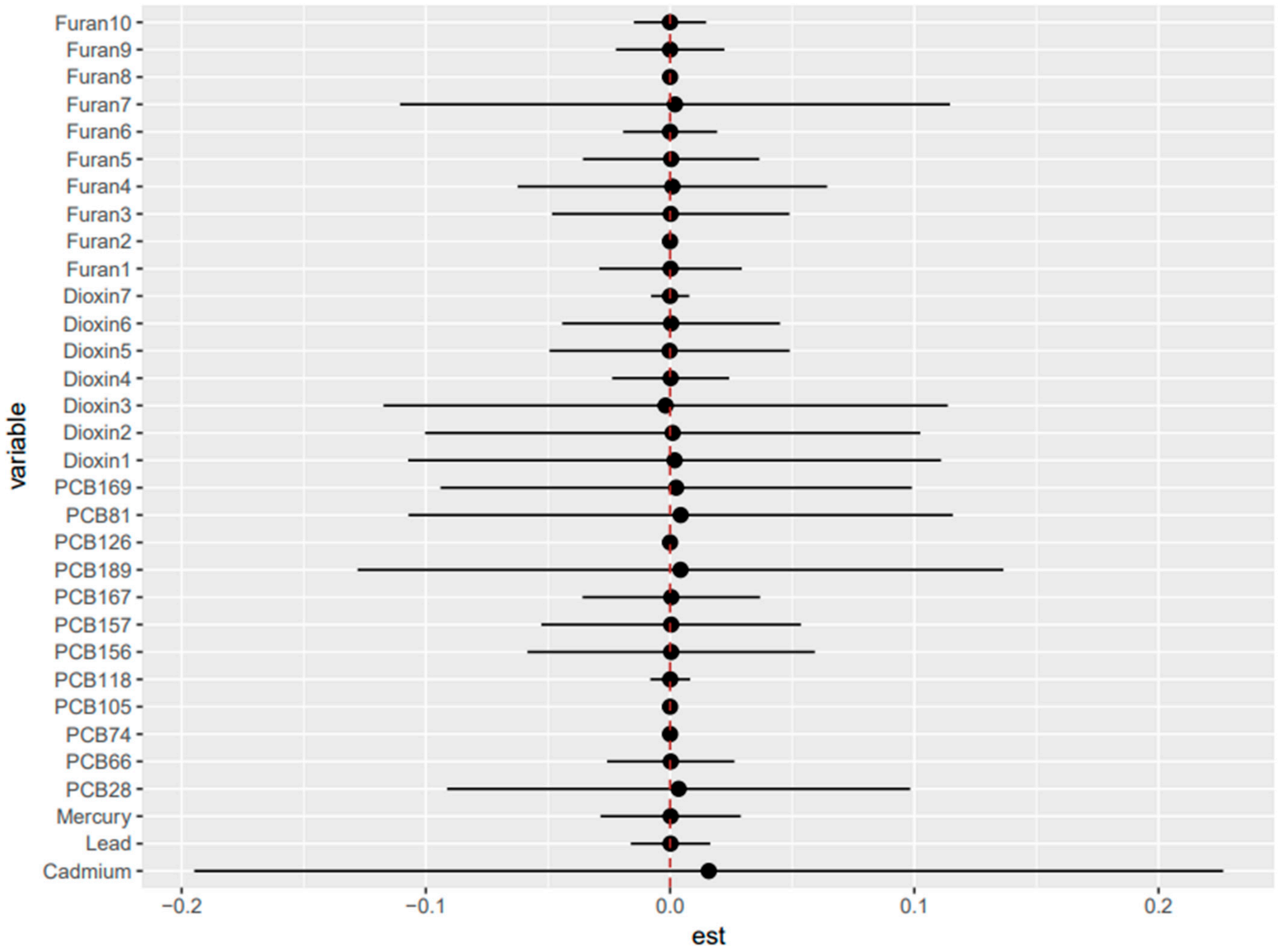

S32. TOTAL BILIRUBIN

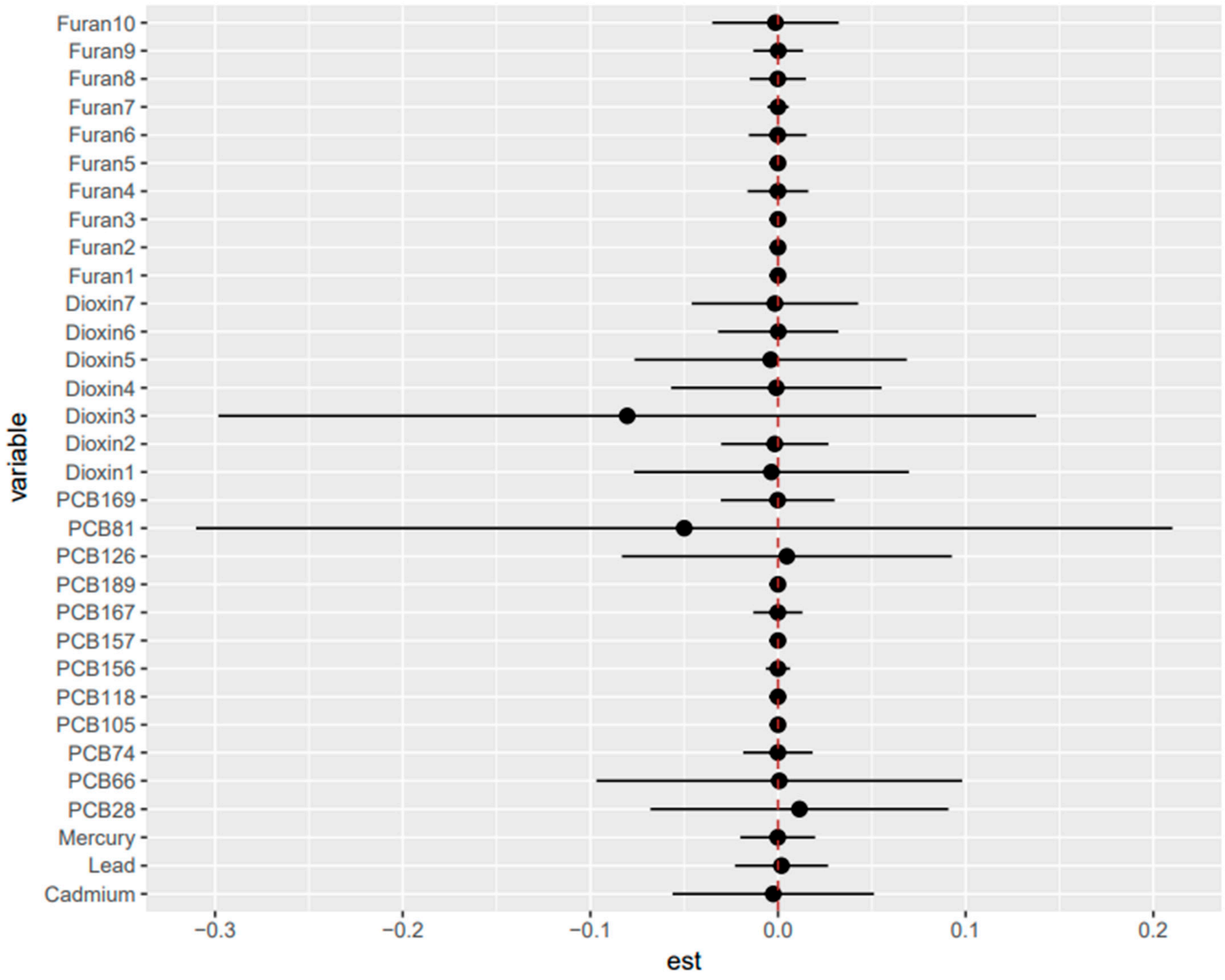

SUPPLEMENTARY DOCUMENTS

S33. TOTAL PROTEIN (TP)

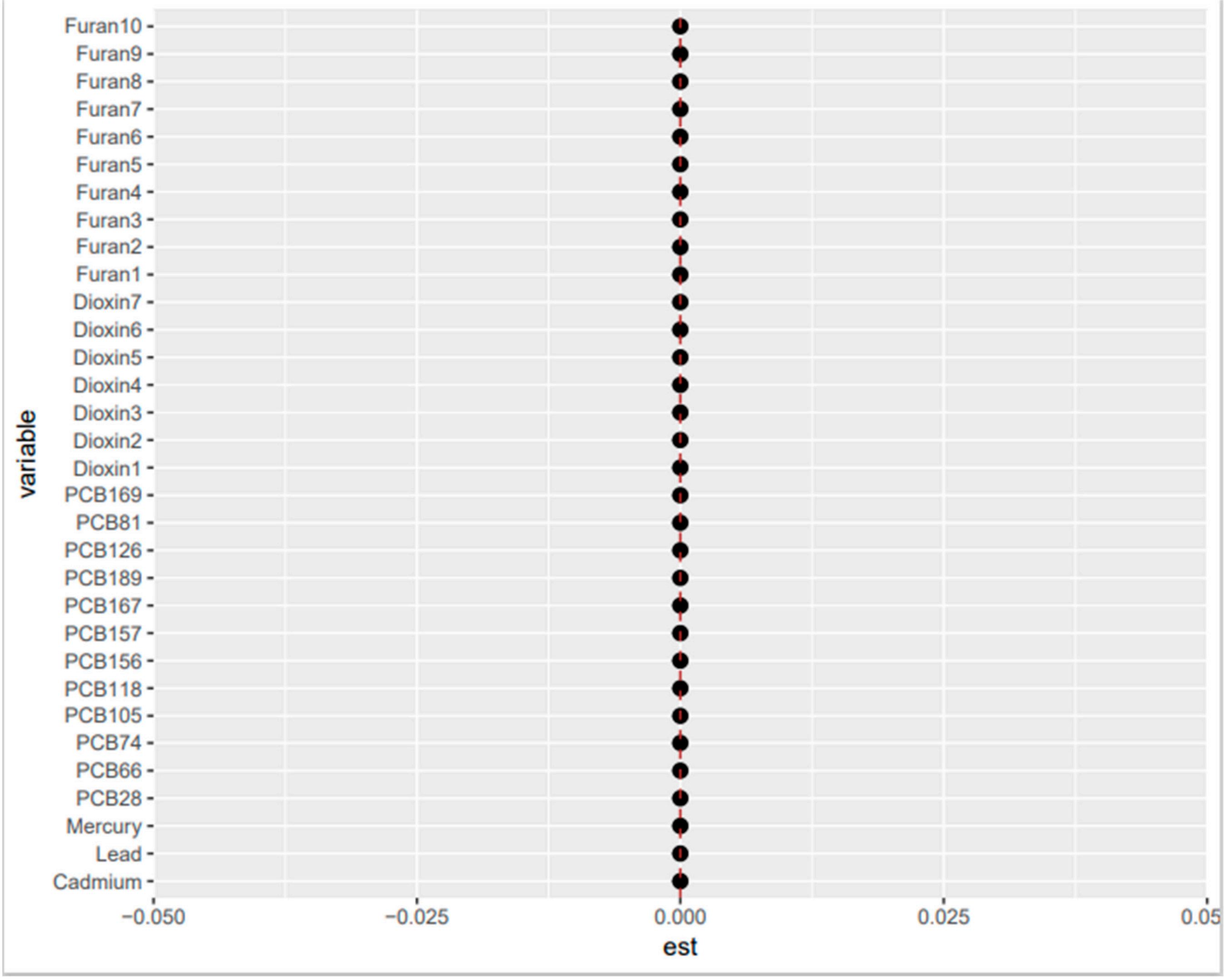

SUPPLEMENTARY DOCUMENTS

TEQ VALUE DIOXIN LIKE CHEMICAL
UNIVARIATE DOSE-RESPONSE RELATIONSHIP

S34. ALBUMIN-TEQ

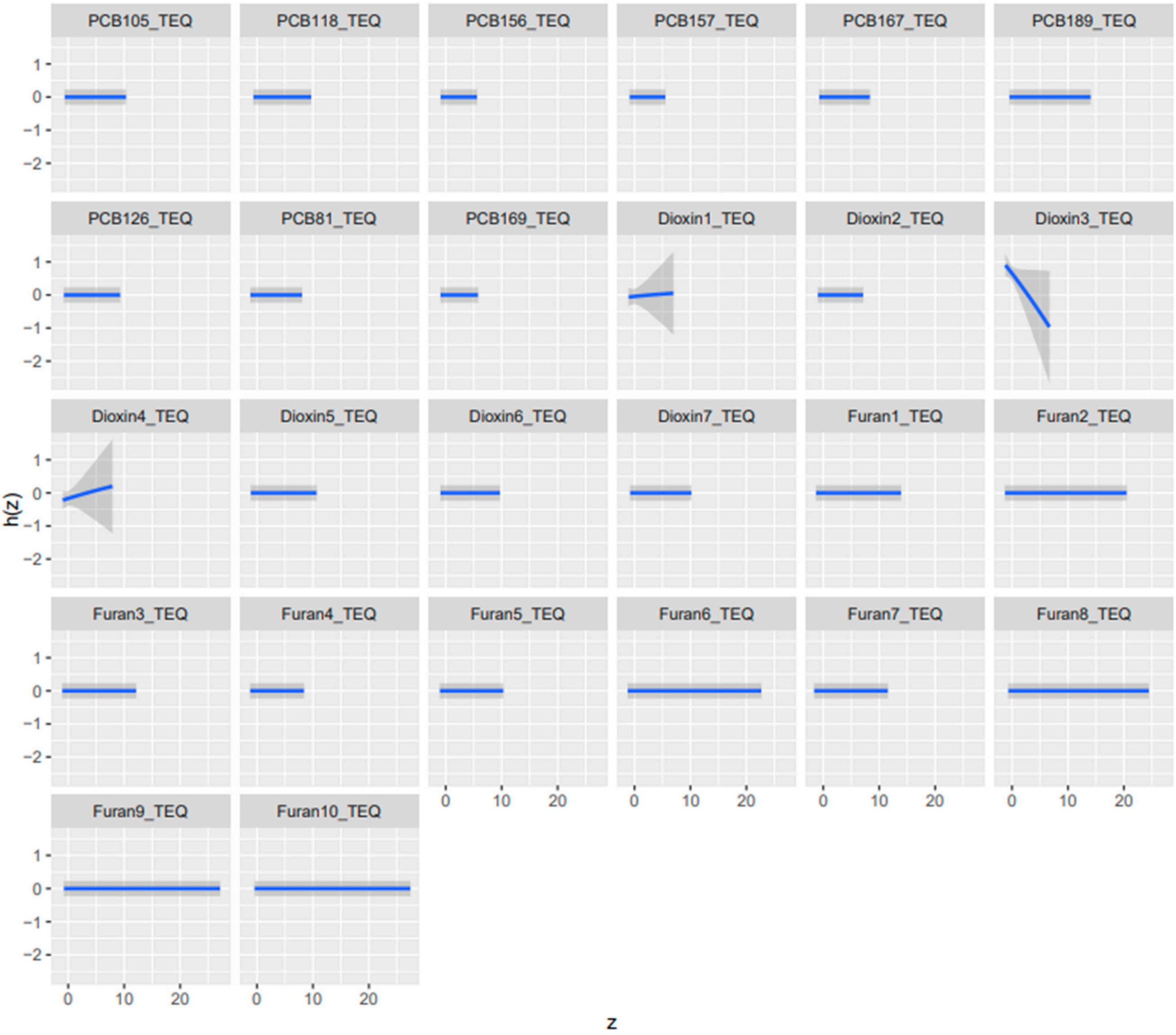

SUPPLEMENTARY DOCUMENTS

S35. ALKALINE PHOSPHATASE (ALP)-TEQ

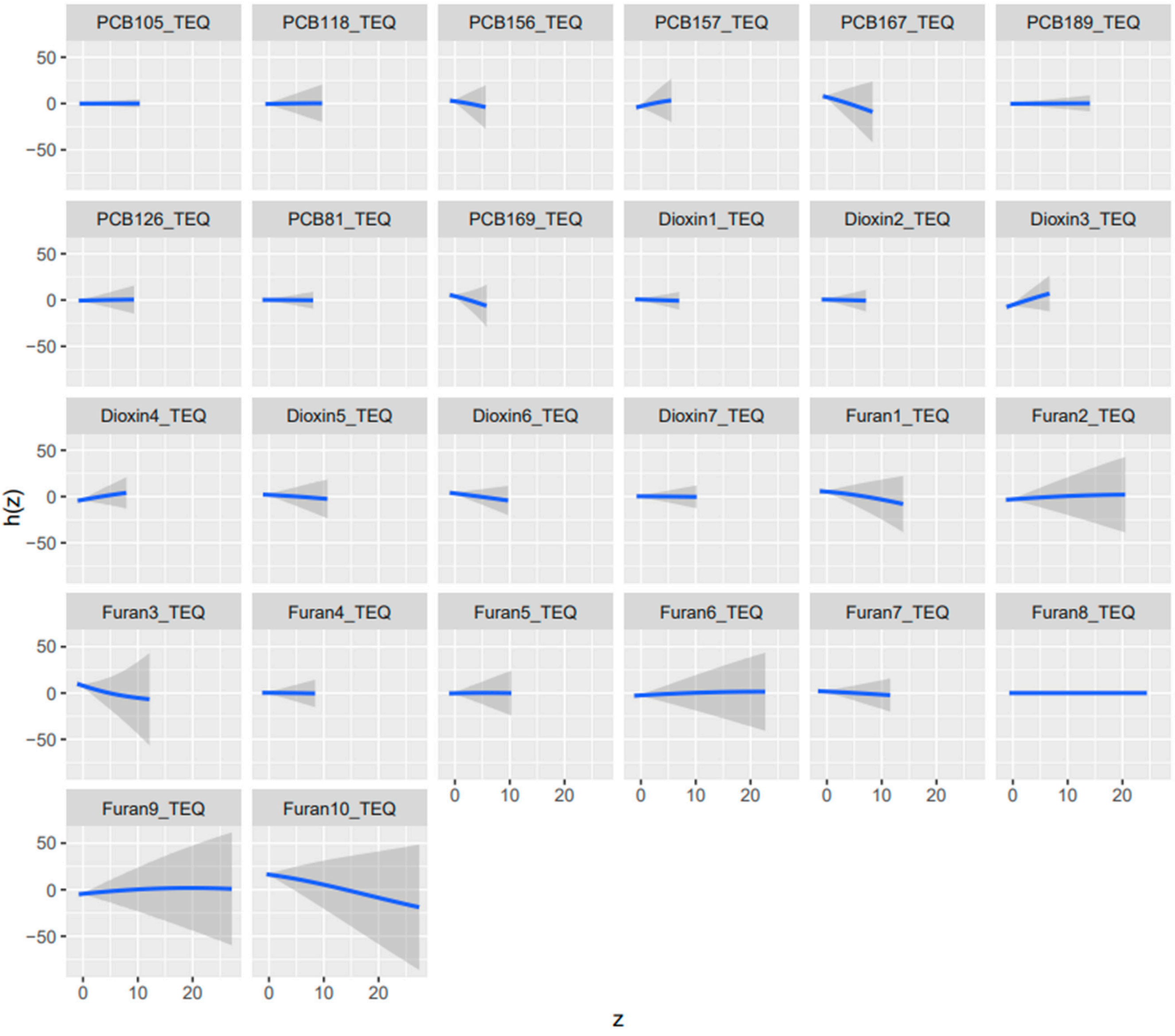

SUPPLEMENTARY DOCUMENTS

S36. ALANINE AMINOTRANSFERASE (ALT)-TEQ

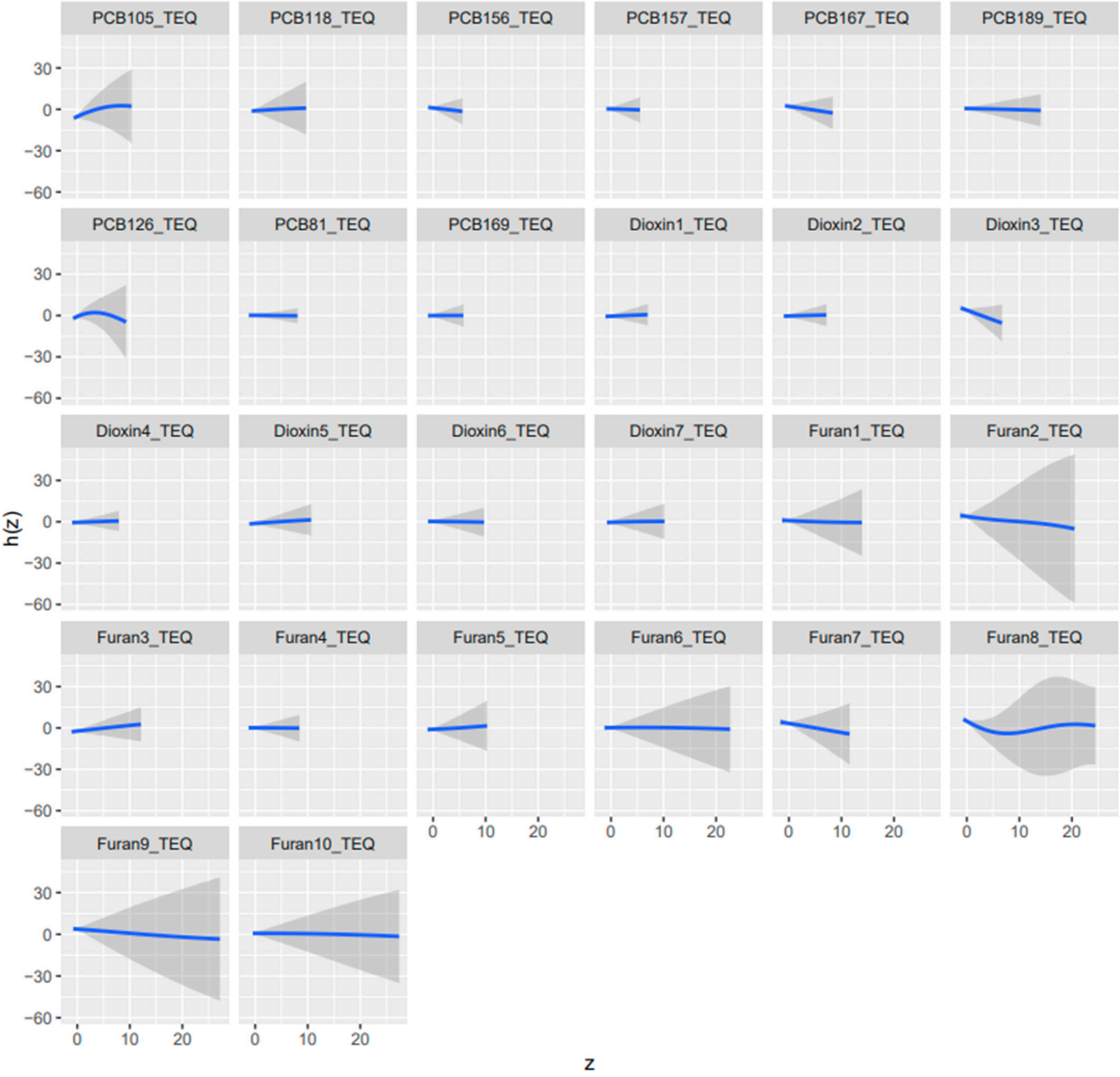

SUPPLEMENTARY DOCUMENTS

S37. ASPARTATE AMINOTRANSFERASE (AST)-TEQ

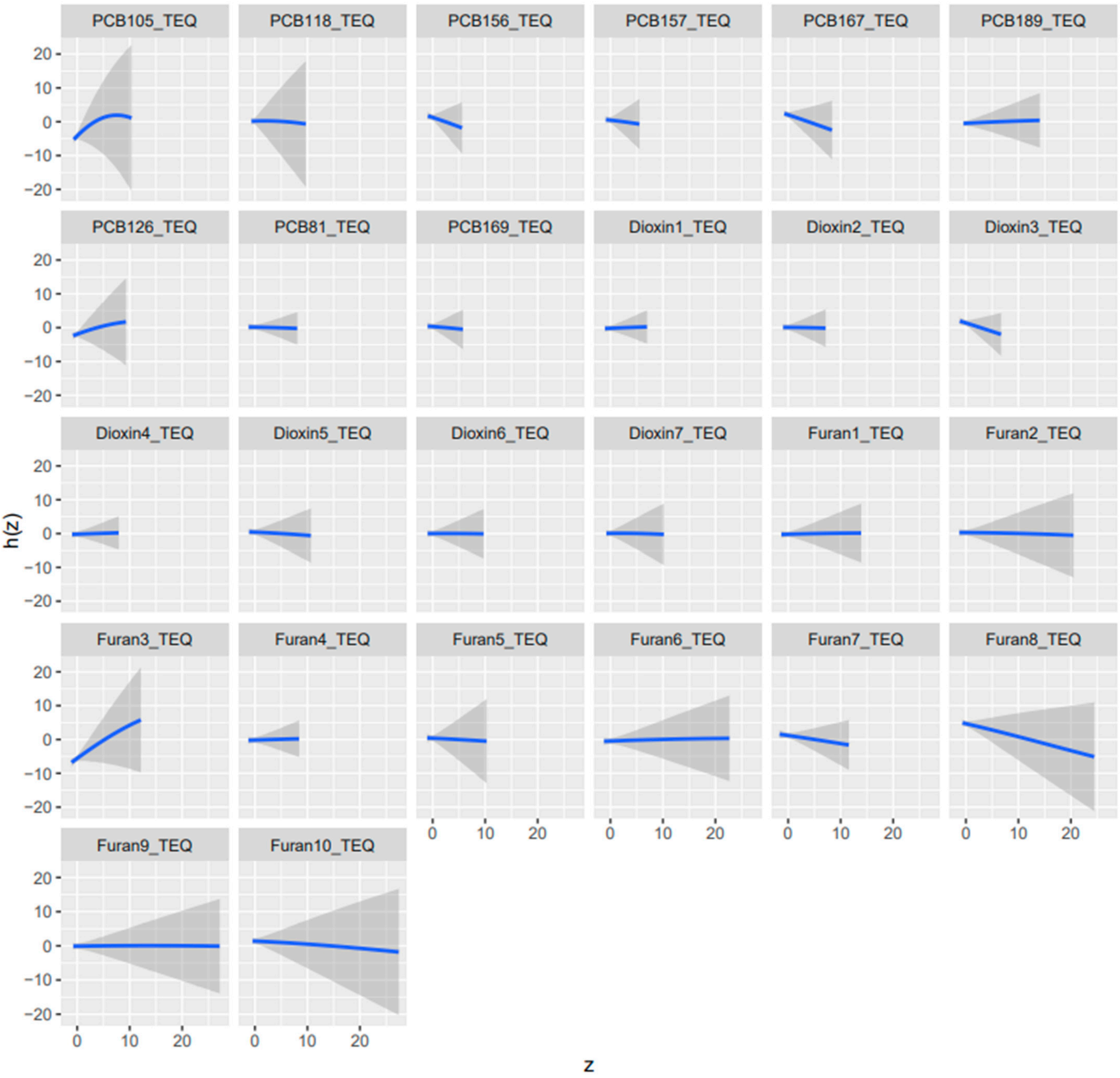

SUPPLEMENTARY DOCUMENTS

S38. GAMMA GLUTAMYL TRANSFERASE (GGT)-TEQ

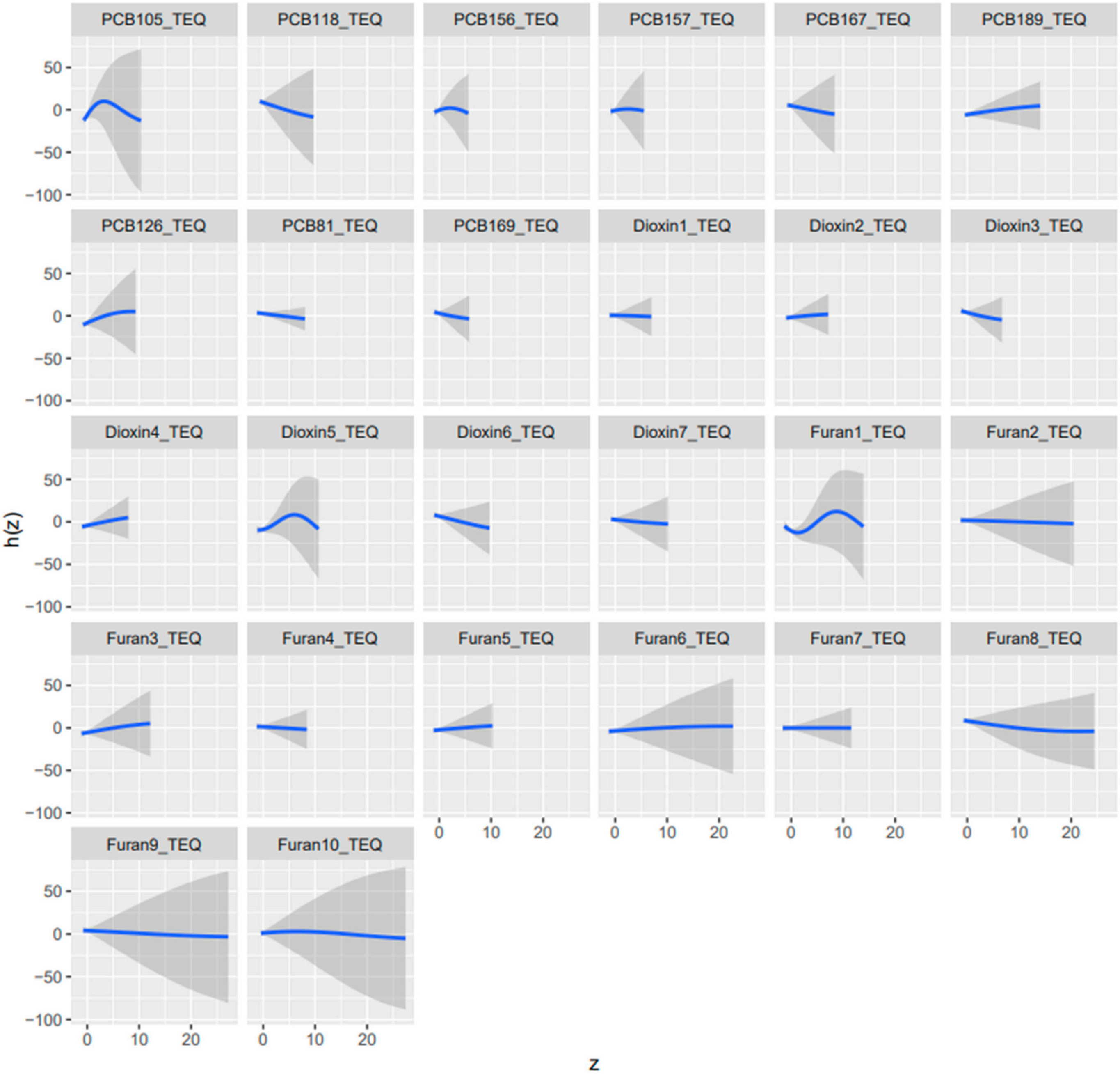

SUPPLEMENTARY DOCUMENTS

S39. LACTATE DEHYDROGENASE (LDH)-TEQ

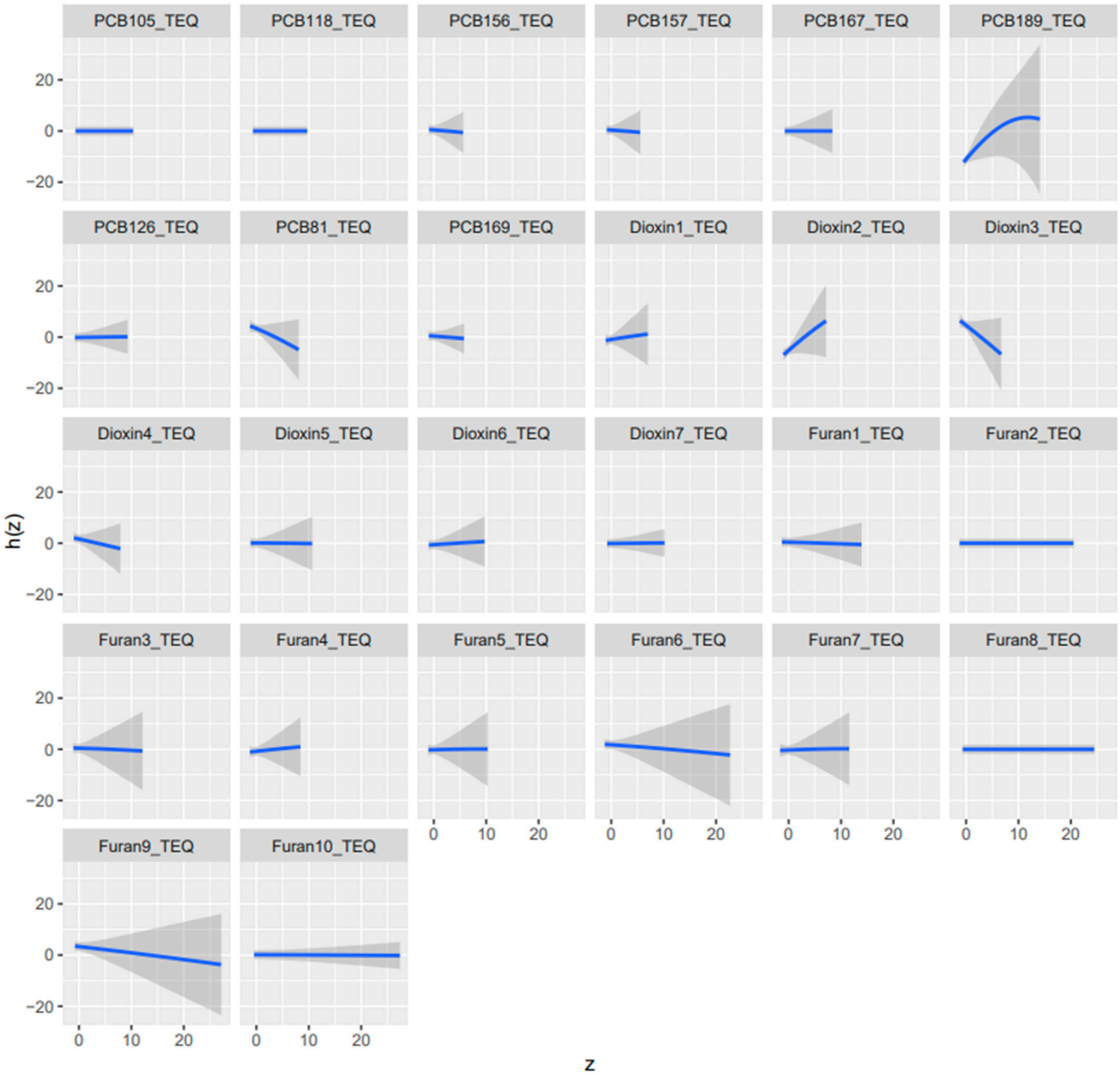

SUPPLEMENTARY DOCUMENTS

S40. TOTAL BILIRUBIN (TB)-TEQ

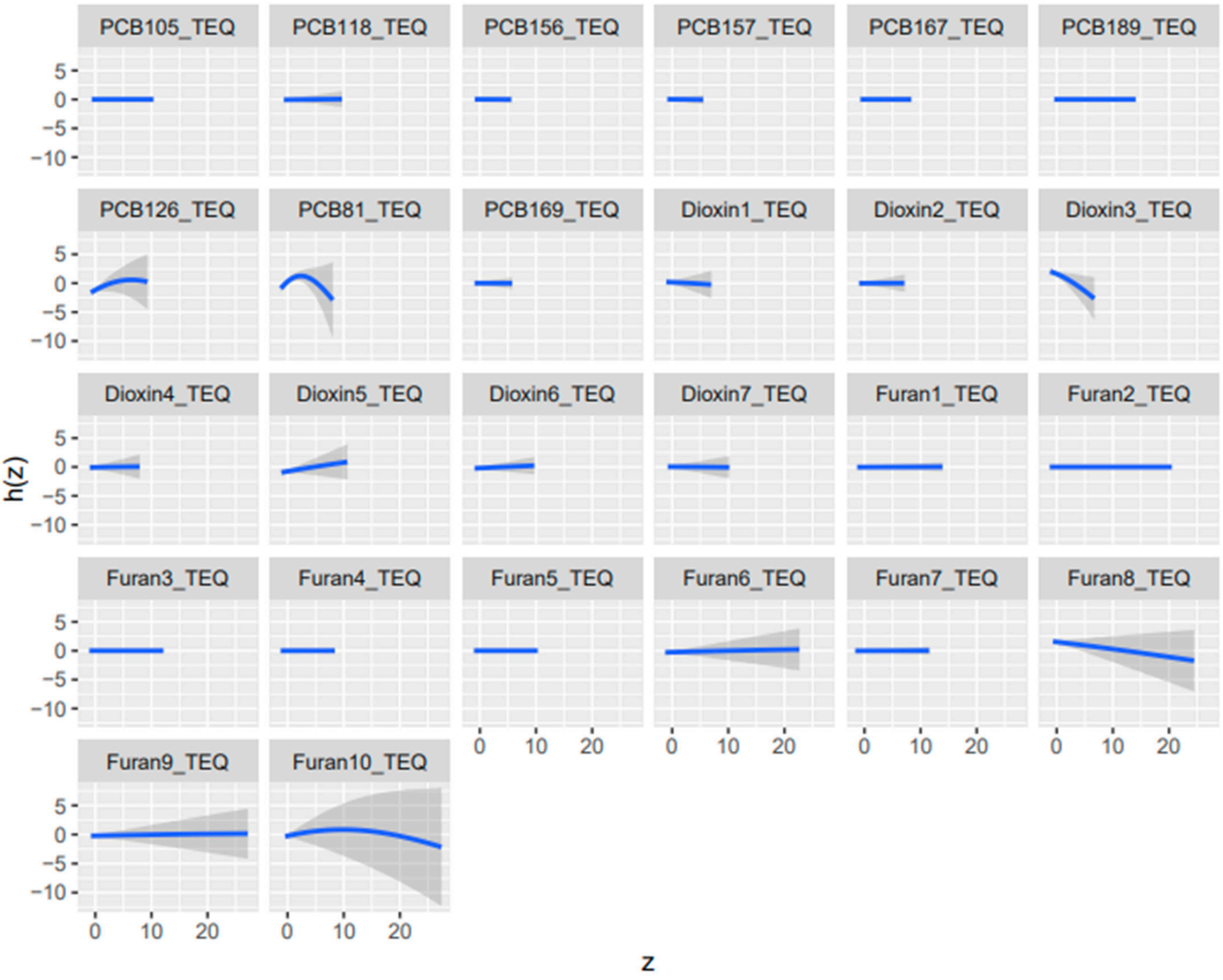

SUPPLEMENTARY DOCUMENTS

S41. TOTAL PROTEIN (TP-TEQ)

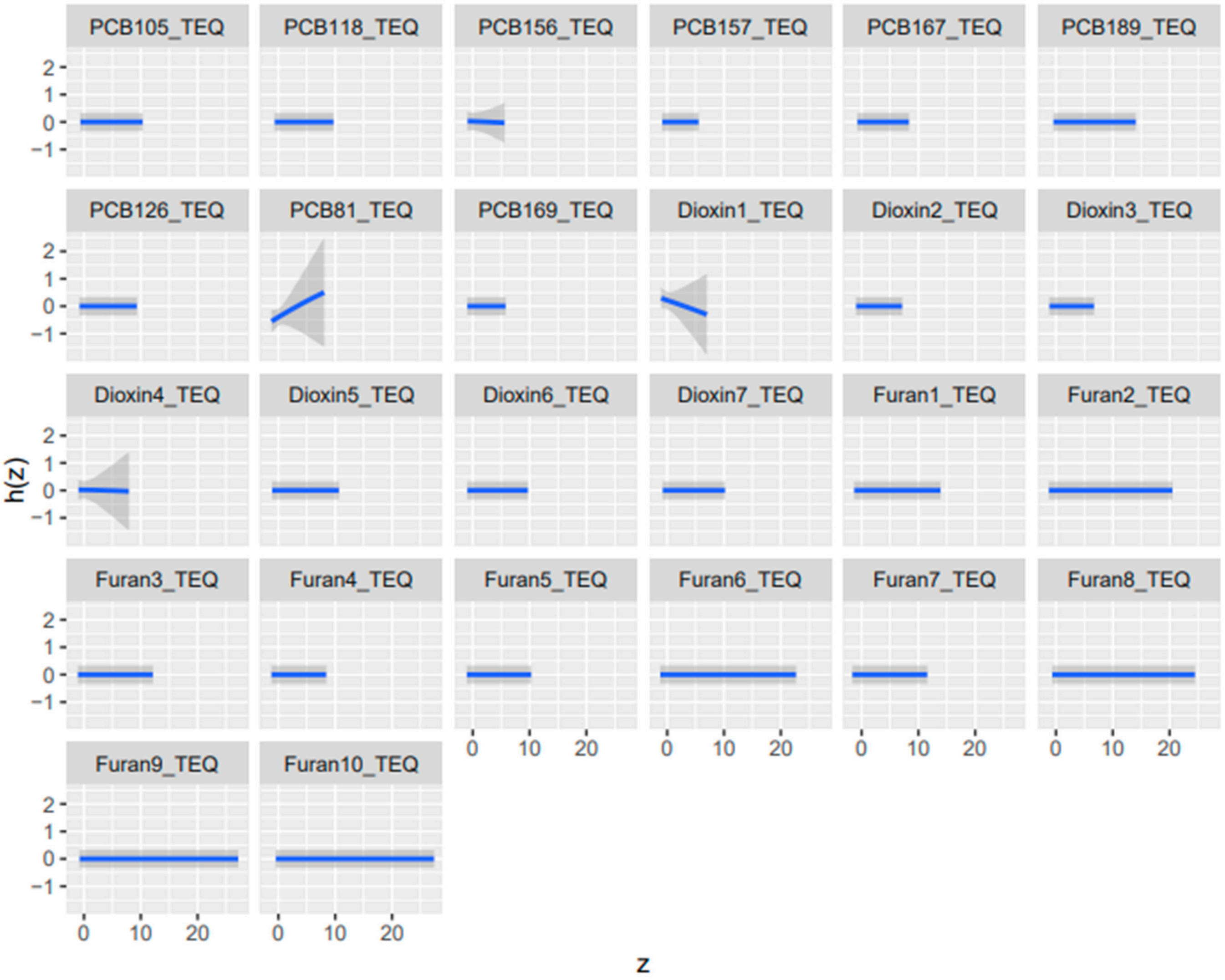

SUPPLEMENTARY DOCUMENTS

OVERALL EXPOSURE EFFECT SUMMARY

S42. ALBUMIN-TEQ

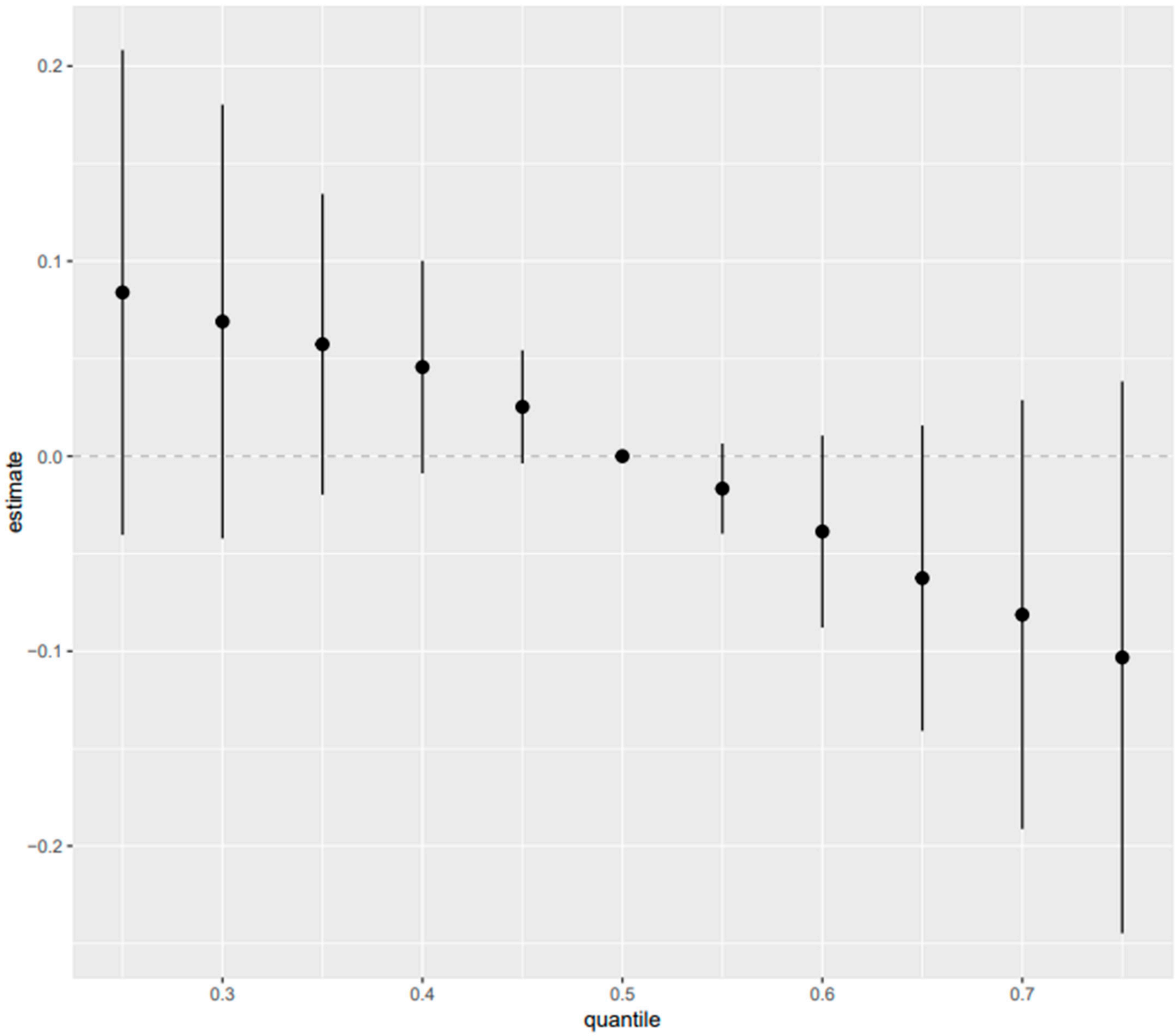

SUPPLEMENTARY DOCUMENTS

S43. ALP-TEQ

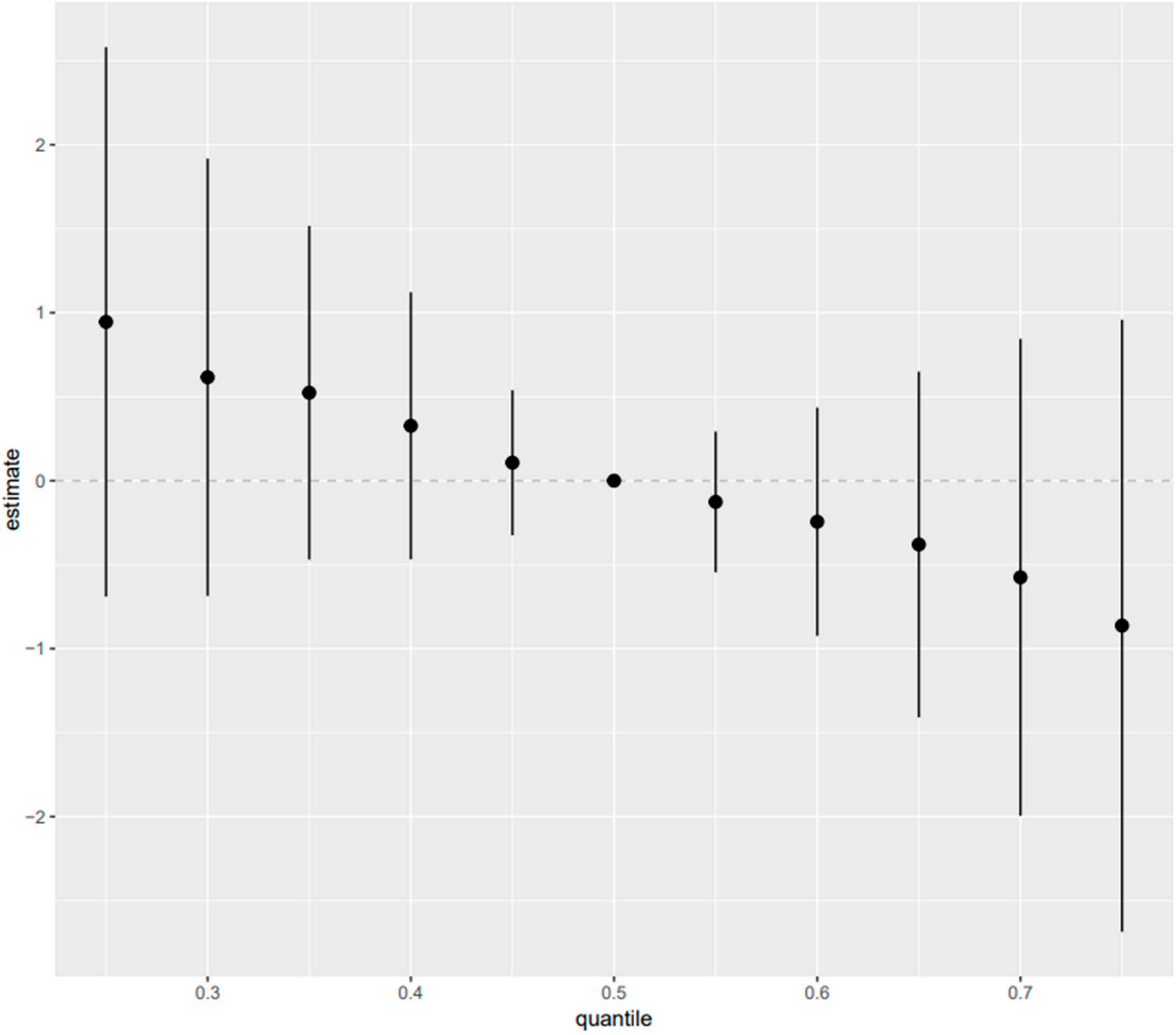

S44. ALT-TEQ

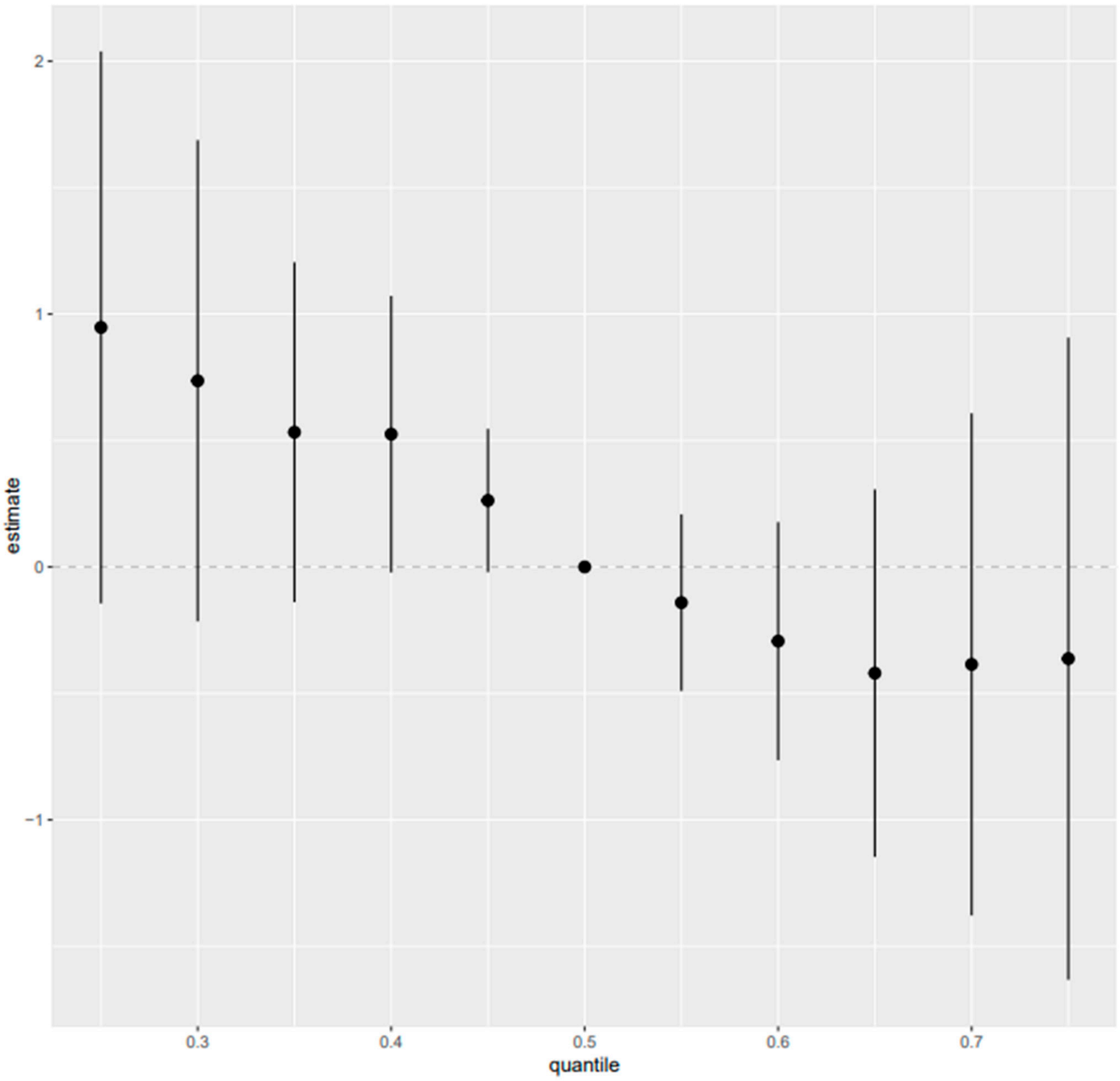

S45. AST-TEQ

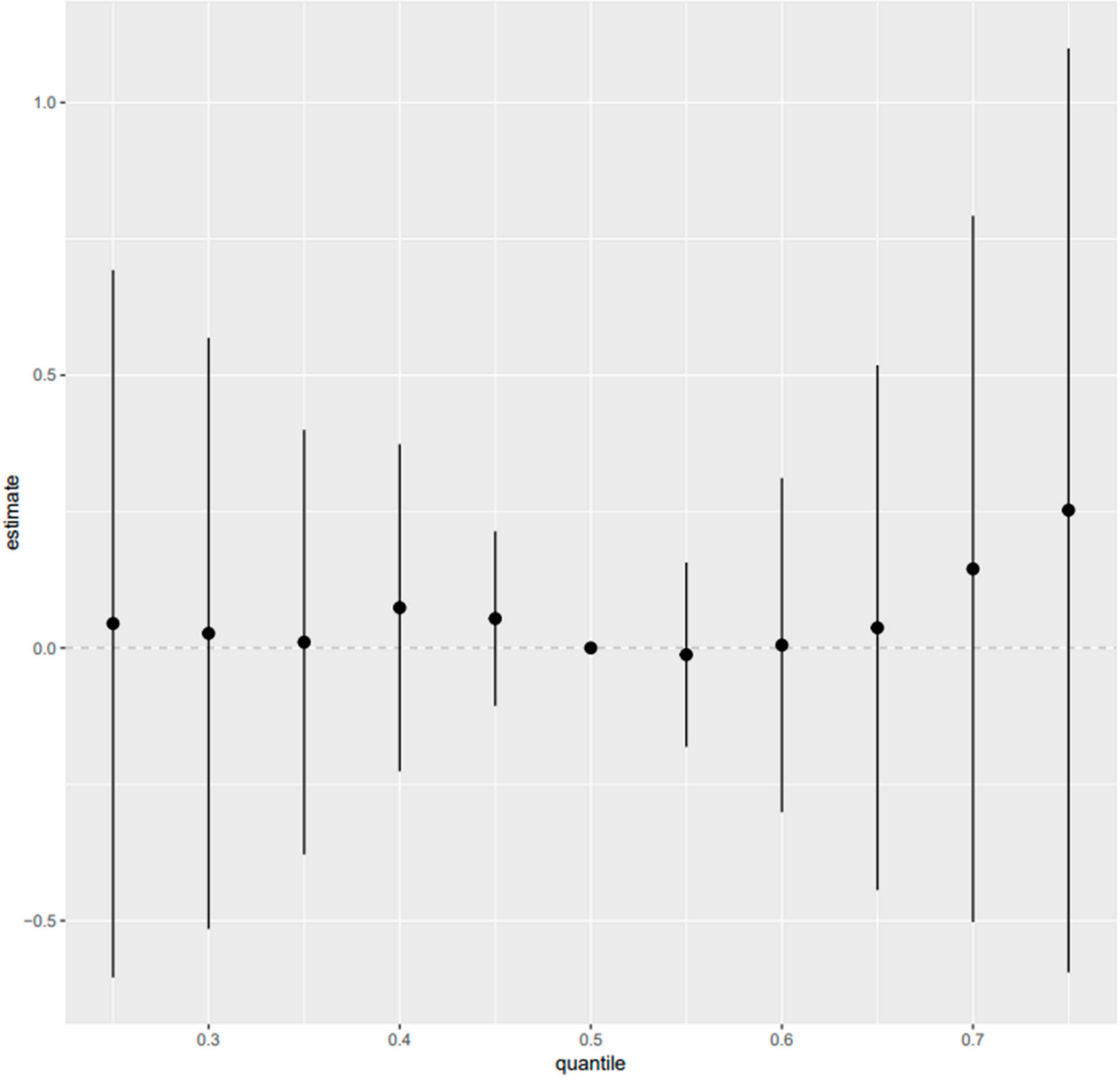

SUPPLEMENTARY DOCUMENTS

S46. GGT-TEQ

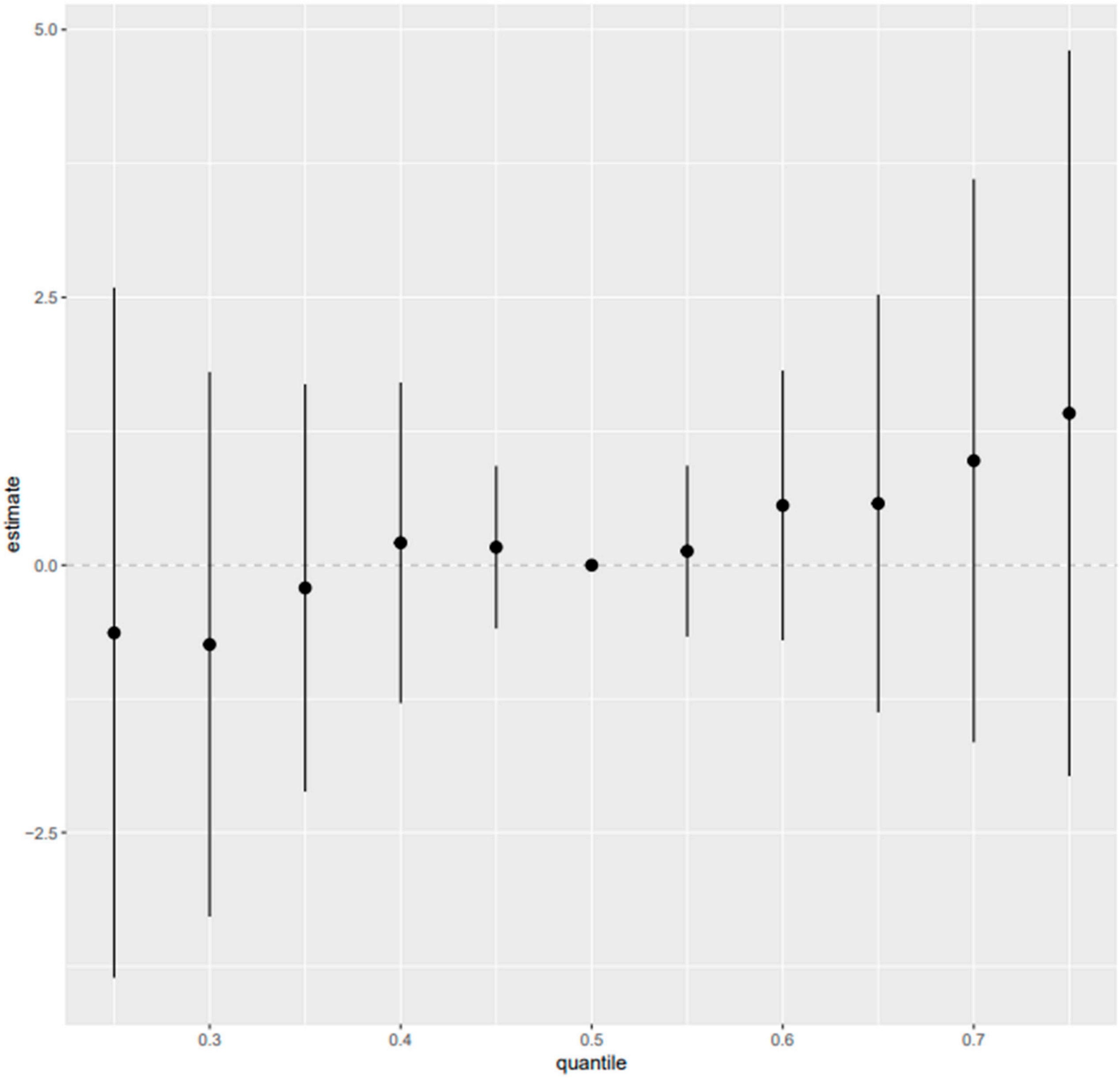

SUPPLEMENTARY DOCUMENTS

S47. LDH-TEQ

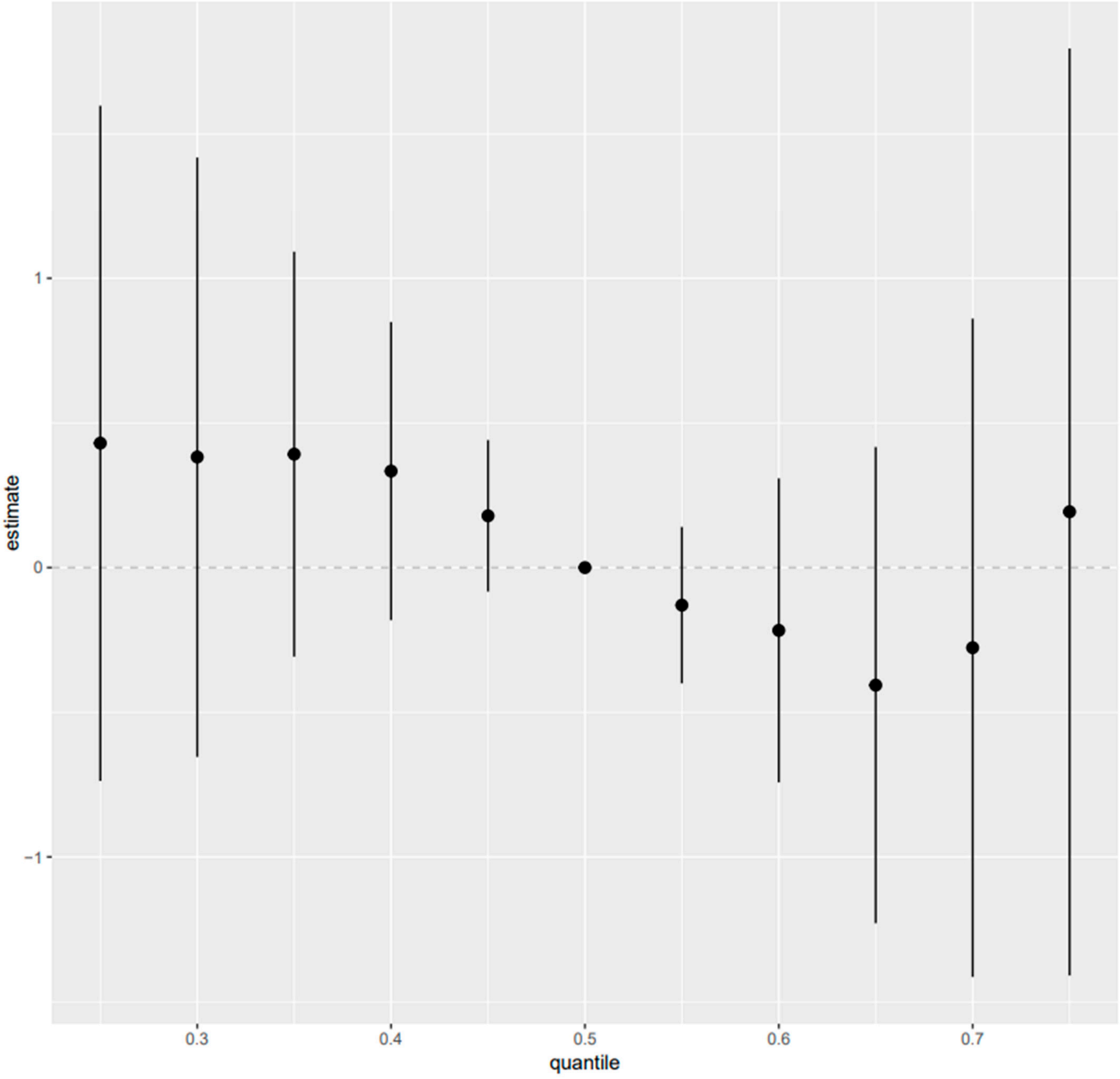

SUPPLEMENTARY DOCUMENTS

S48. TB-TEQ

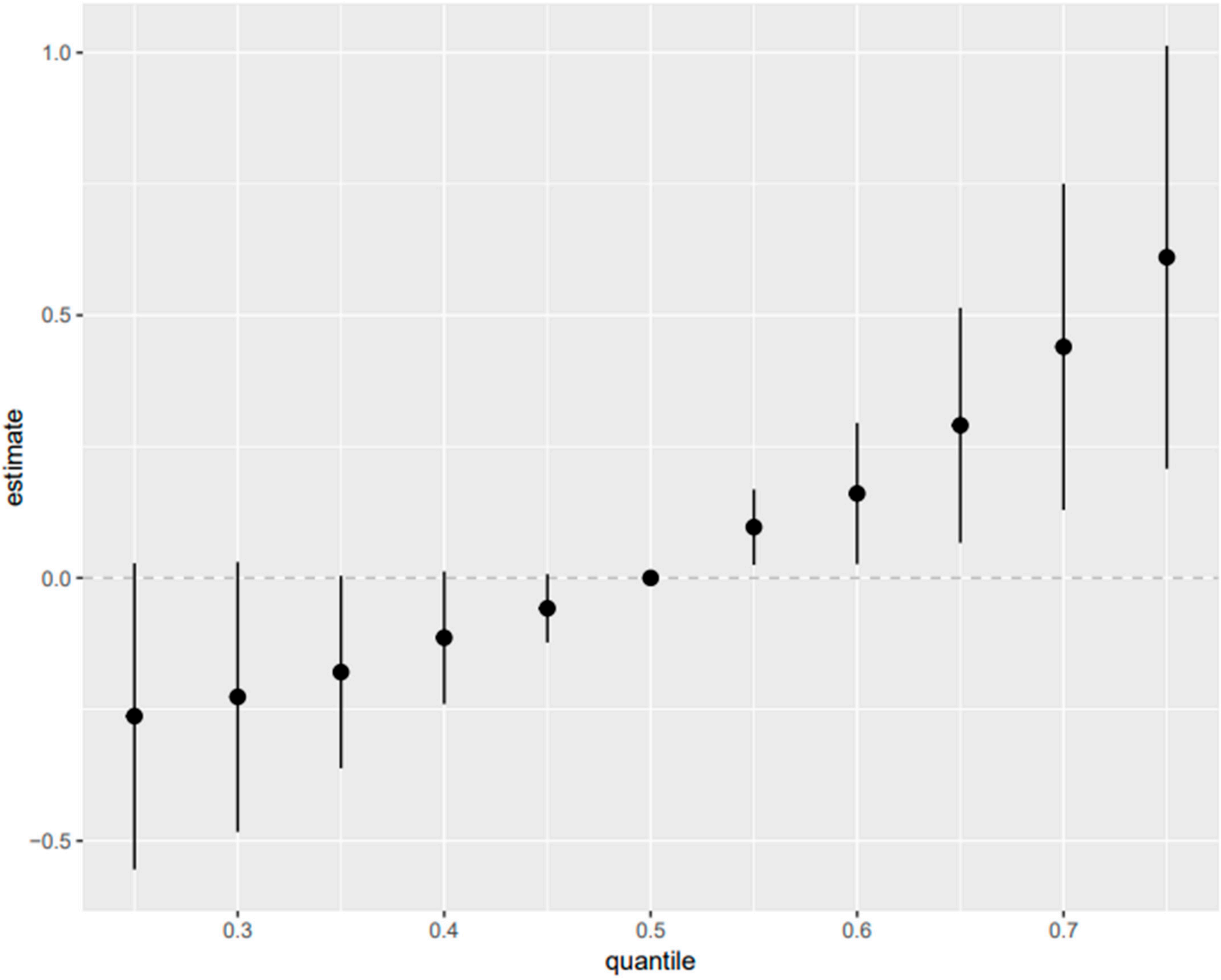

S49. TP-TEQ

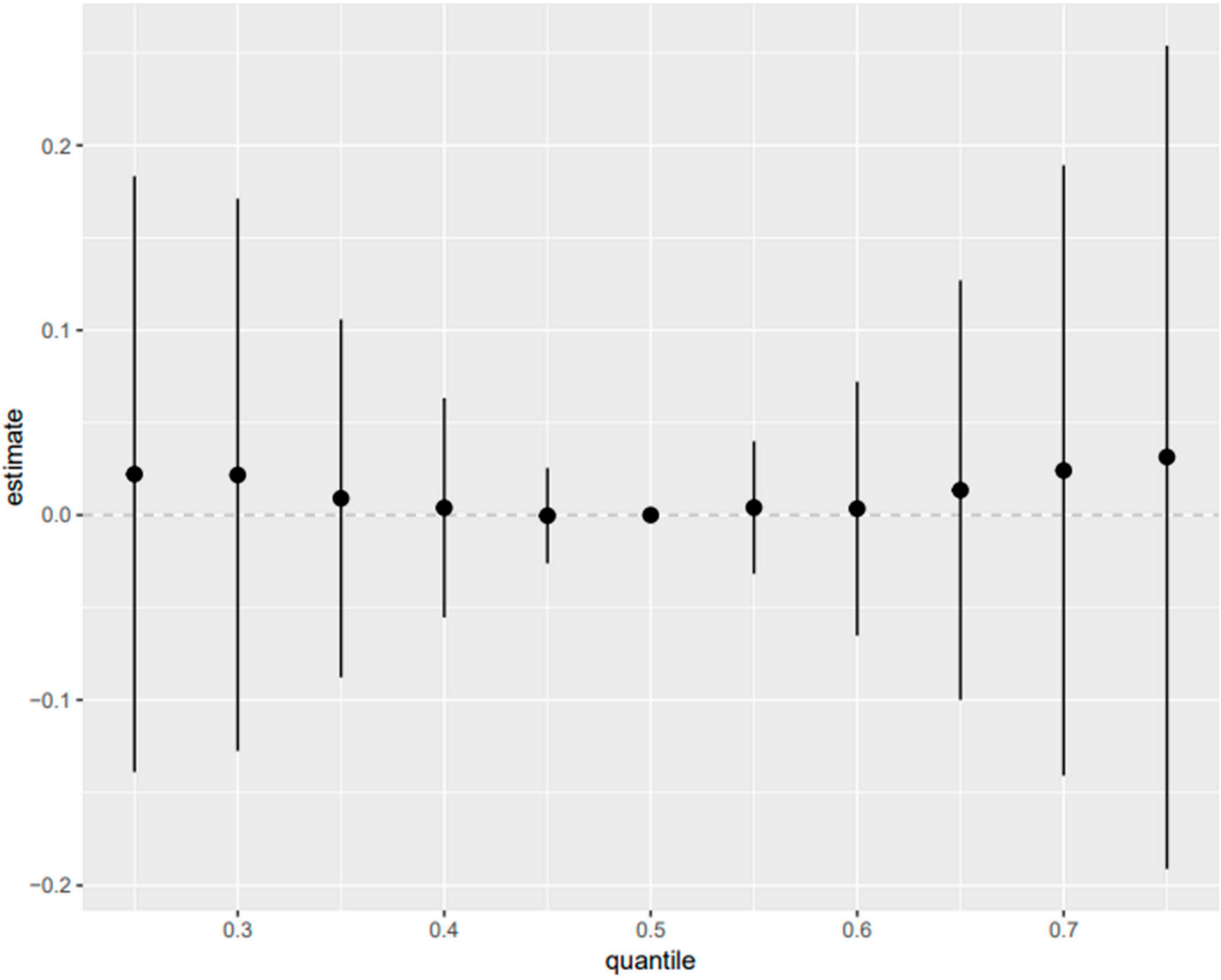

SUPPLEMENTARY DOCUMENTS

SINGLE VARIABLE EFFECTS

S50. Albumin-TEQ

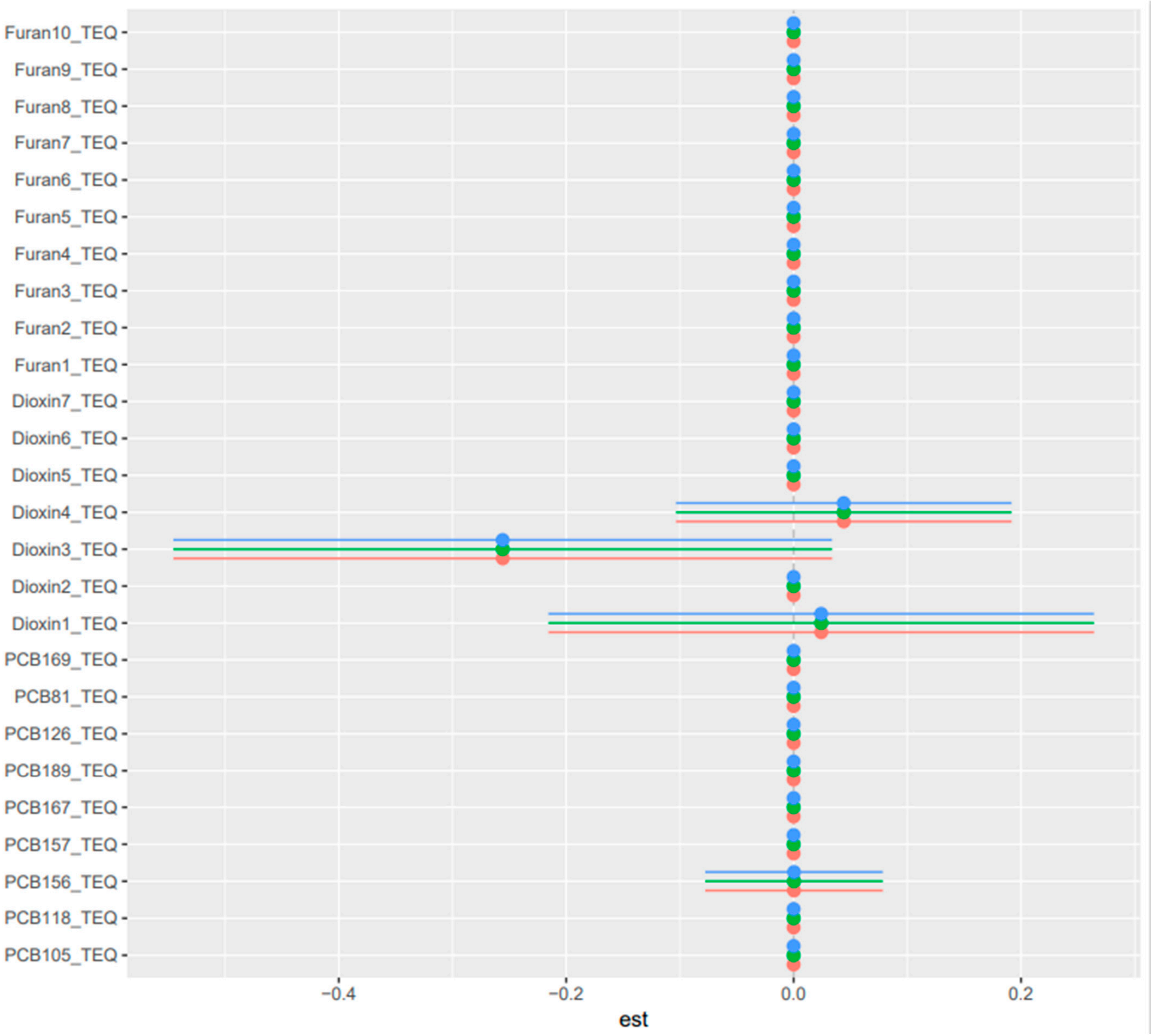

SUPPLEMENTARY DOCUMENTS

S51. ALP-TEQ

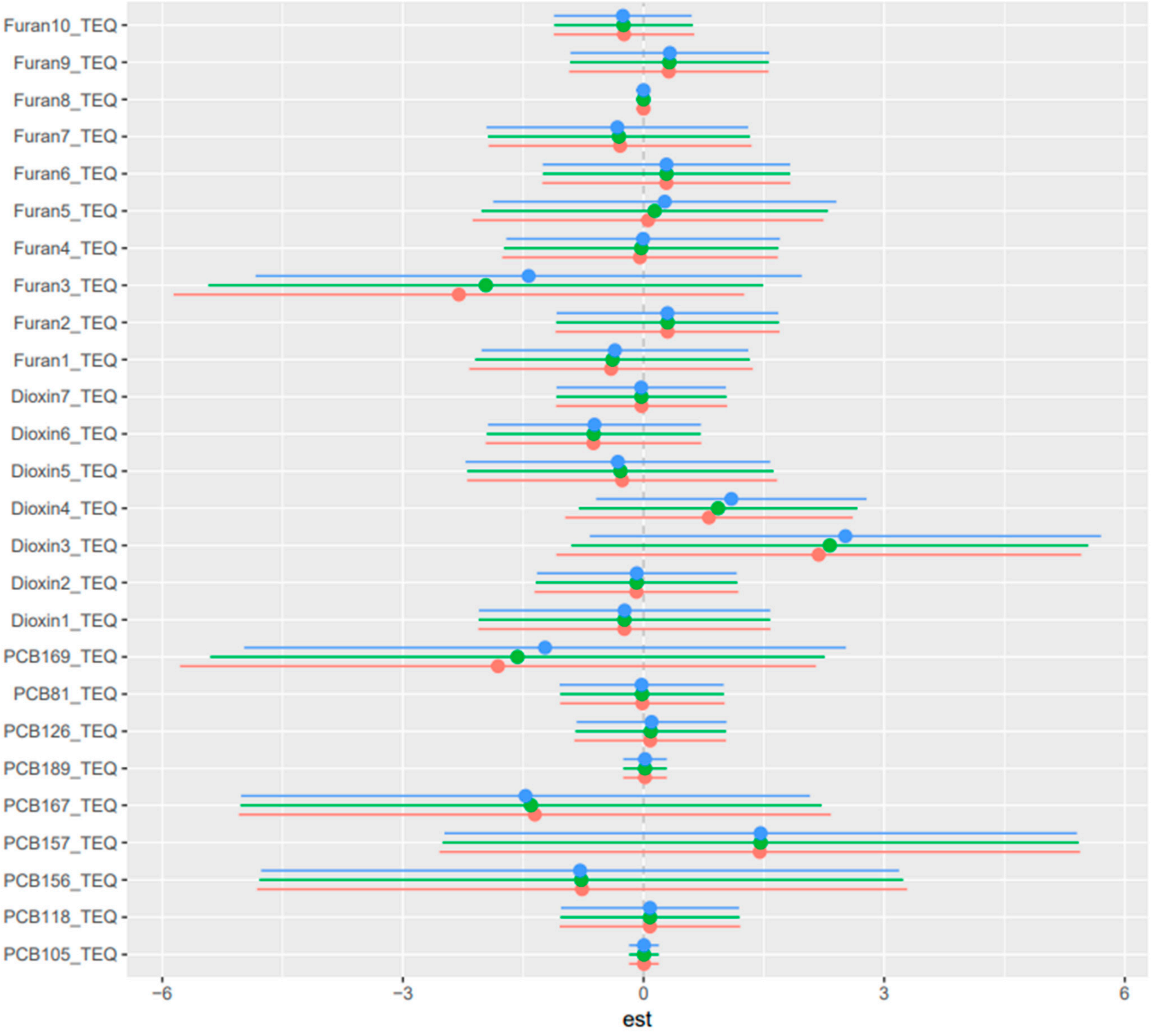

SUPPLEMENTARY DOCUMENTS

S52. ALT-TEQ

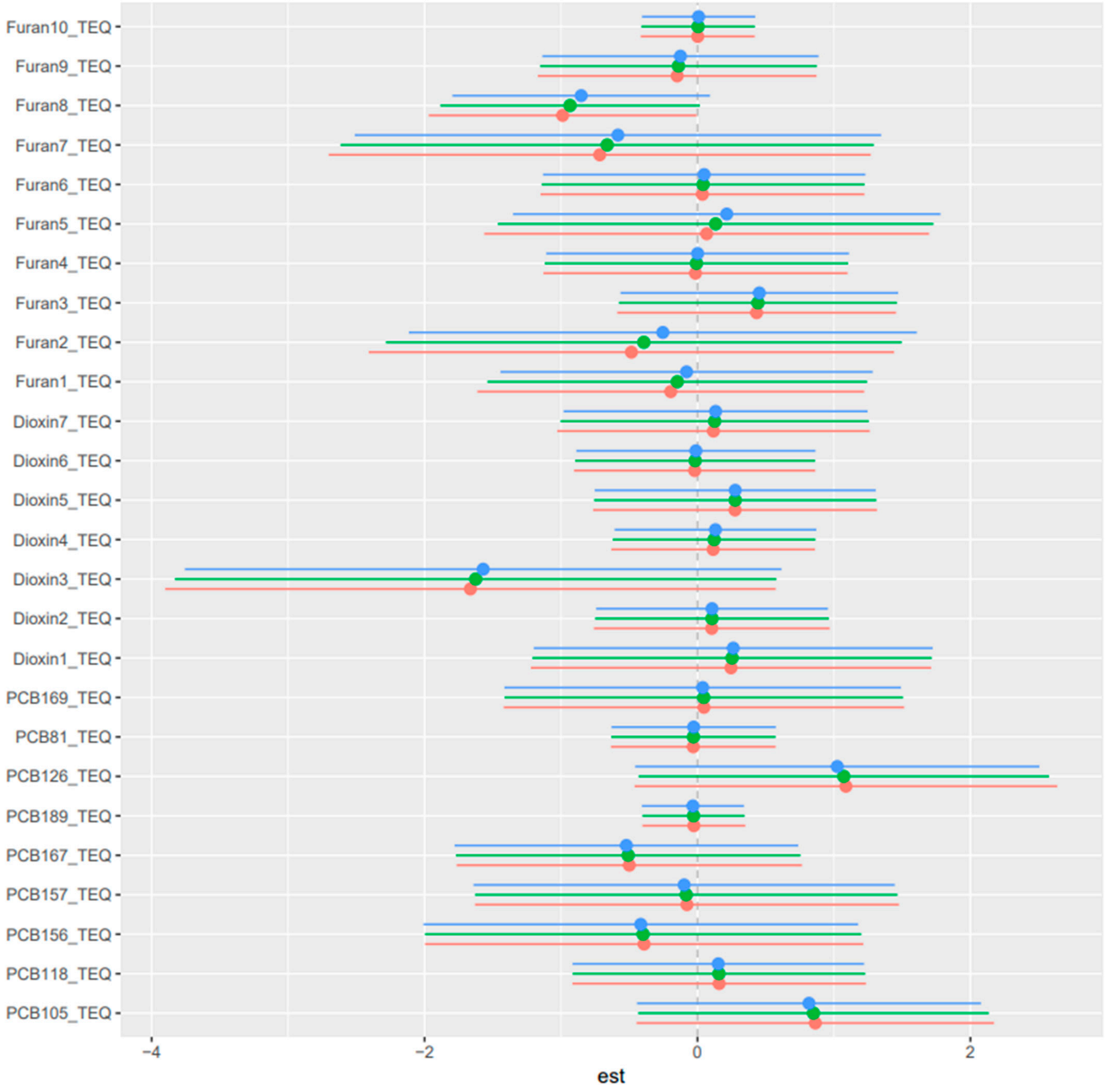

SUPPLEMENTARY DOCUMENTS

S53. AST-TEQ

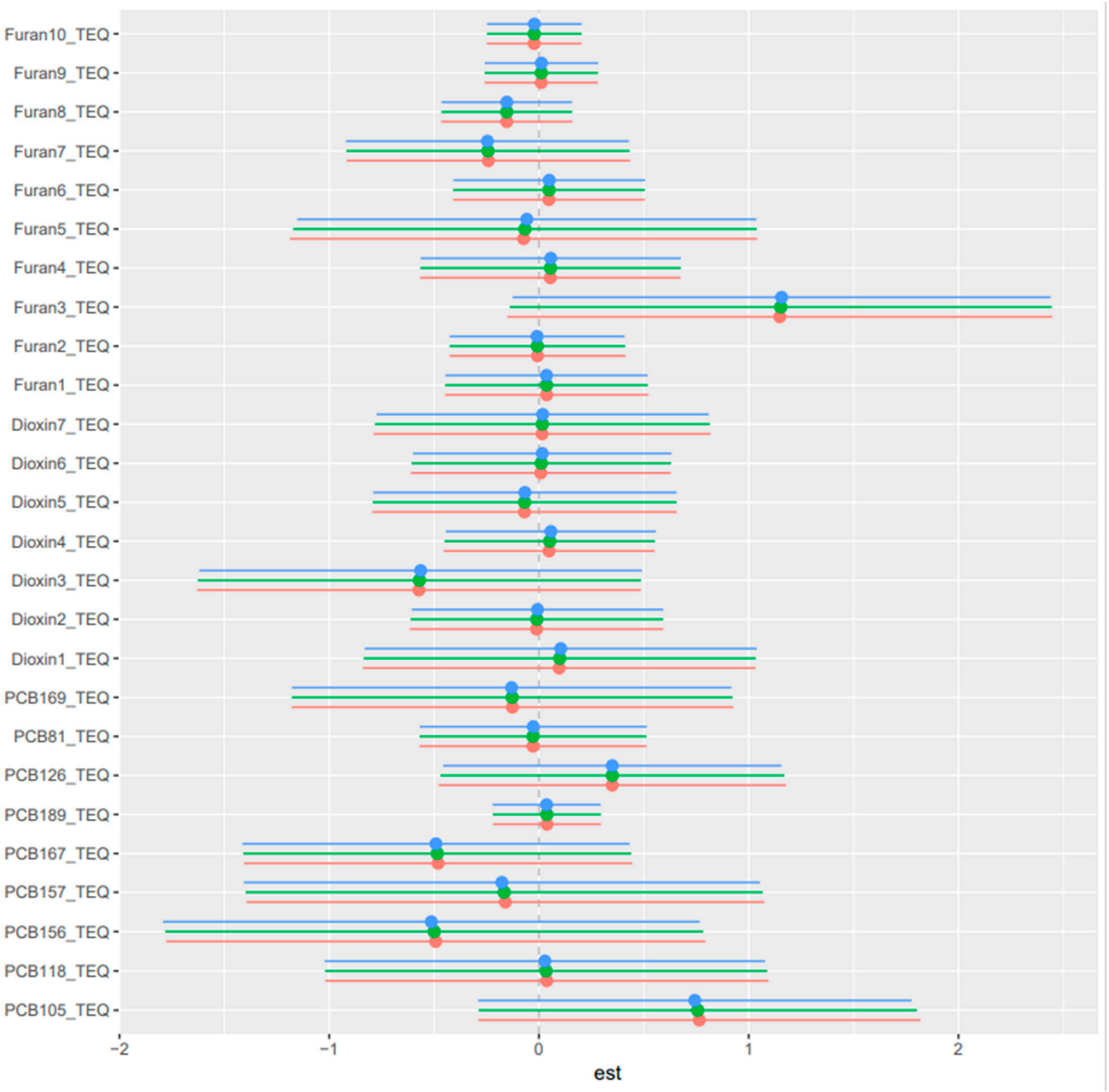

SUPPLEMENTARY DOCUMENTS

S54. GGT-TEQ

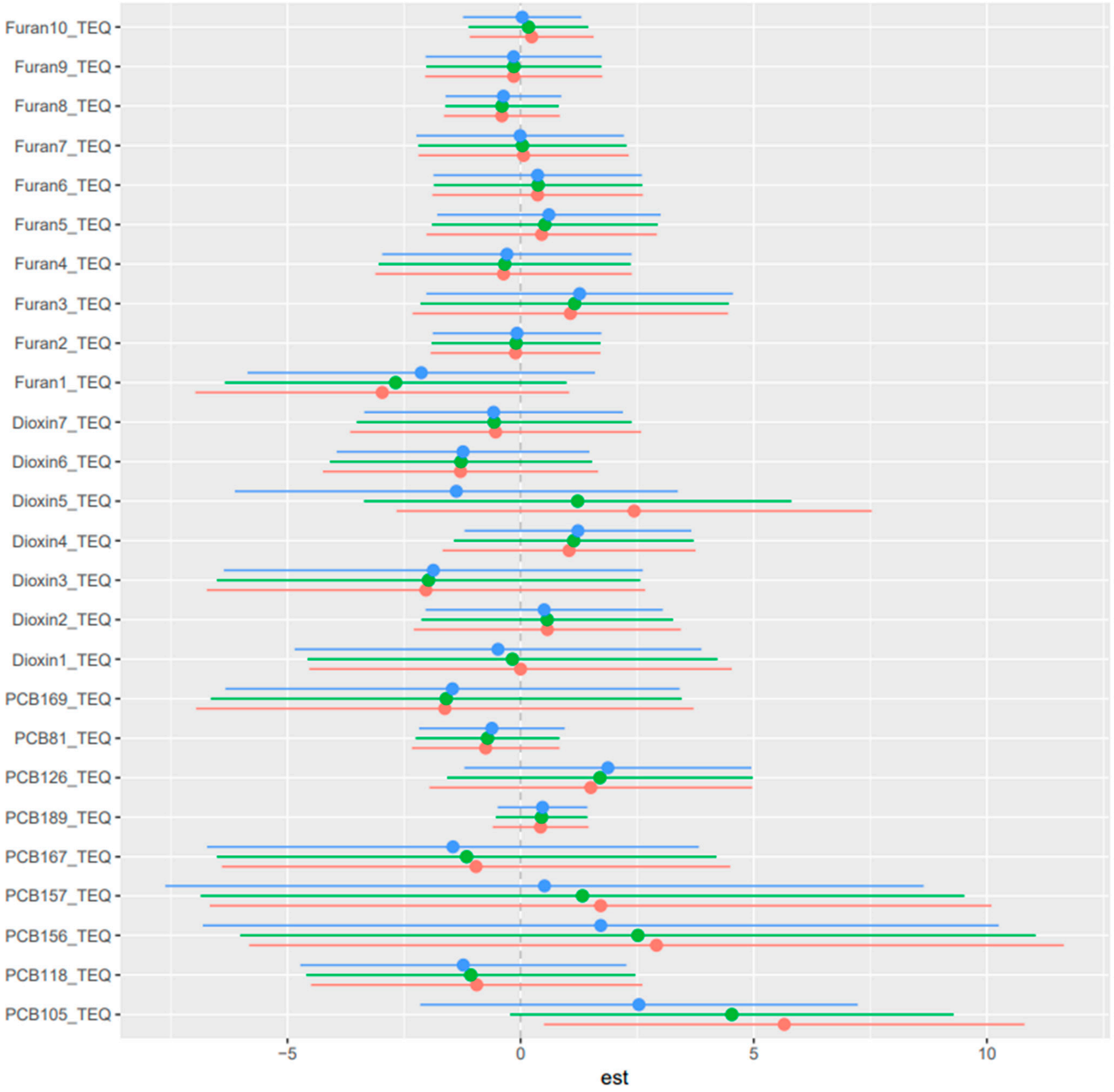

SUPPLEMENTARY DOCUMENTS

S55. LDH-TEQ

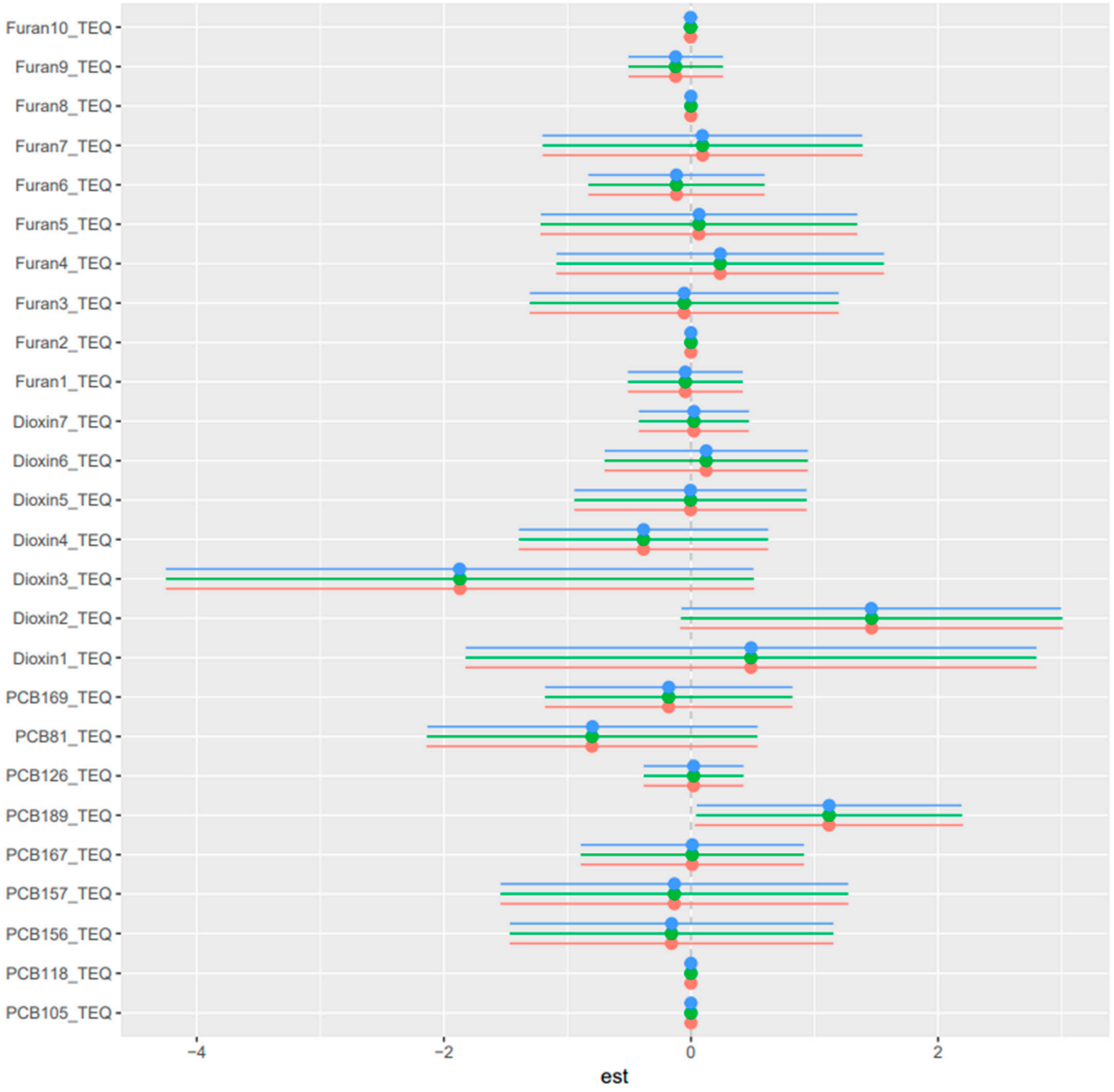

SUPPLEMENTARY DOCUMENTS

S56. TB-TEQ

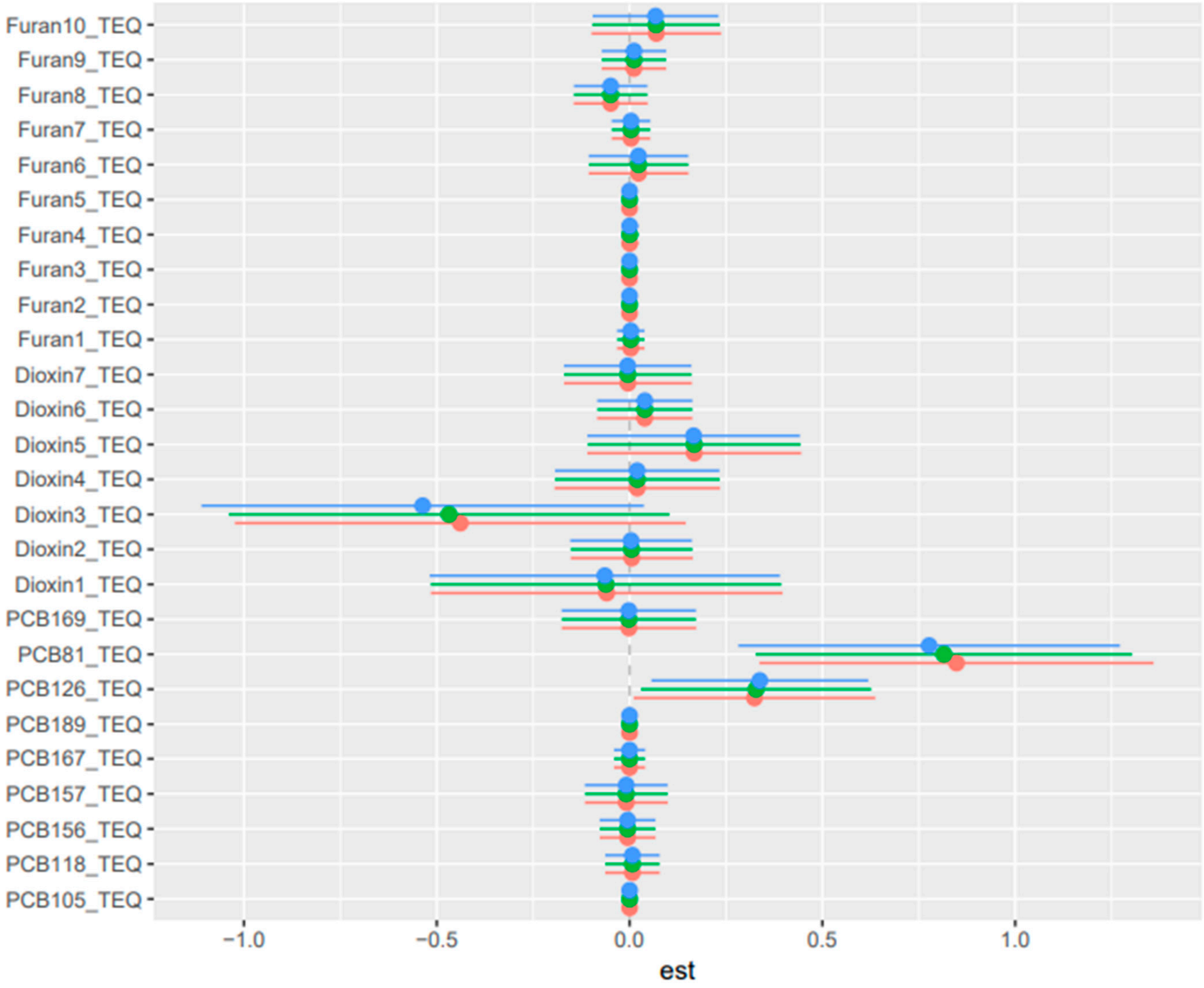

SUPPLEMENTARY DOCUMENTS

S57. TP-TEQ

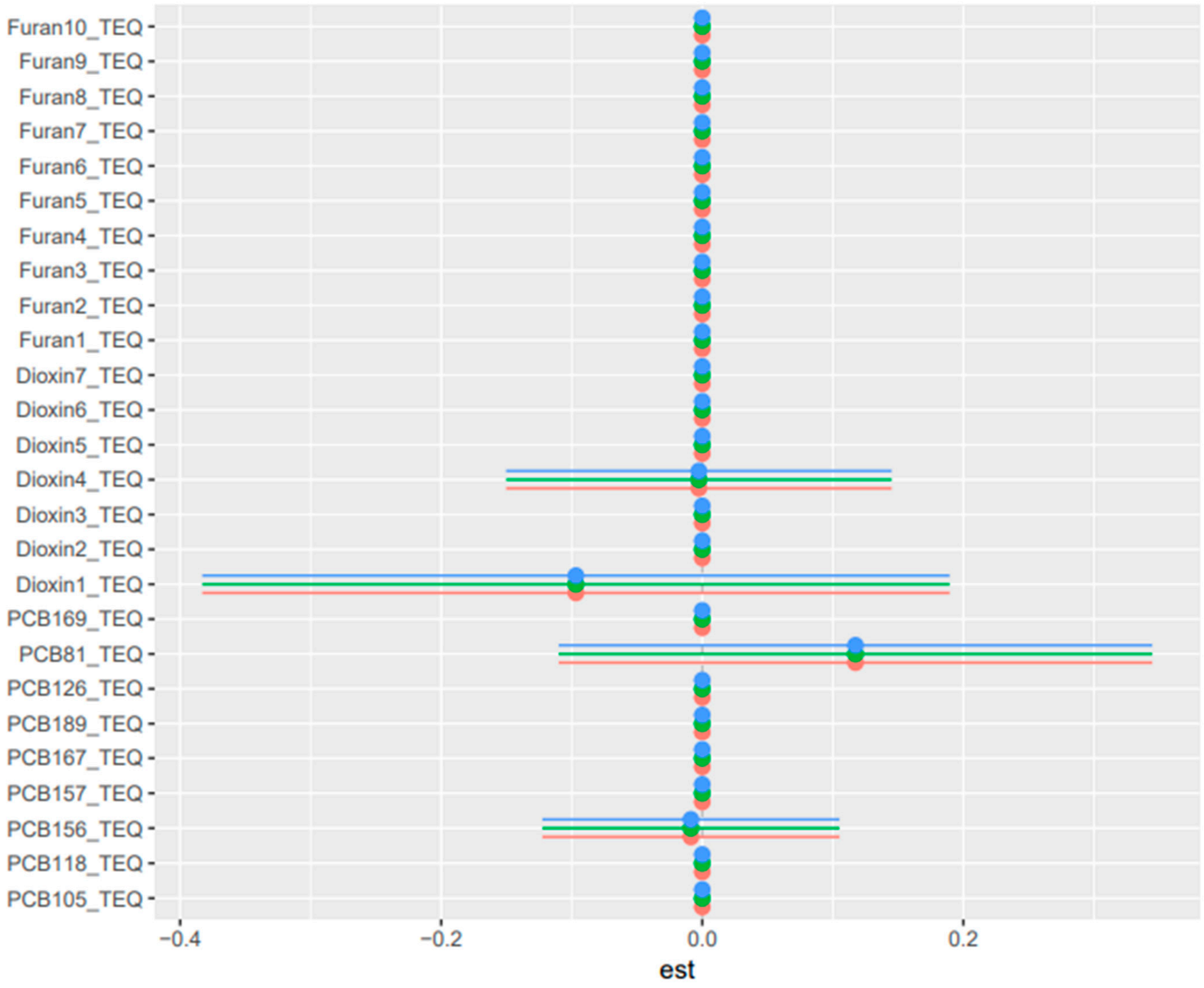

SUPPLEMENTARY DOCUMENTS

Single Variable Interaction

S58. Albumin-TEQ

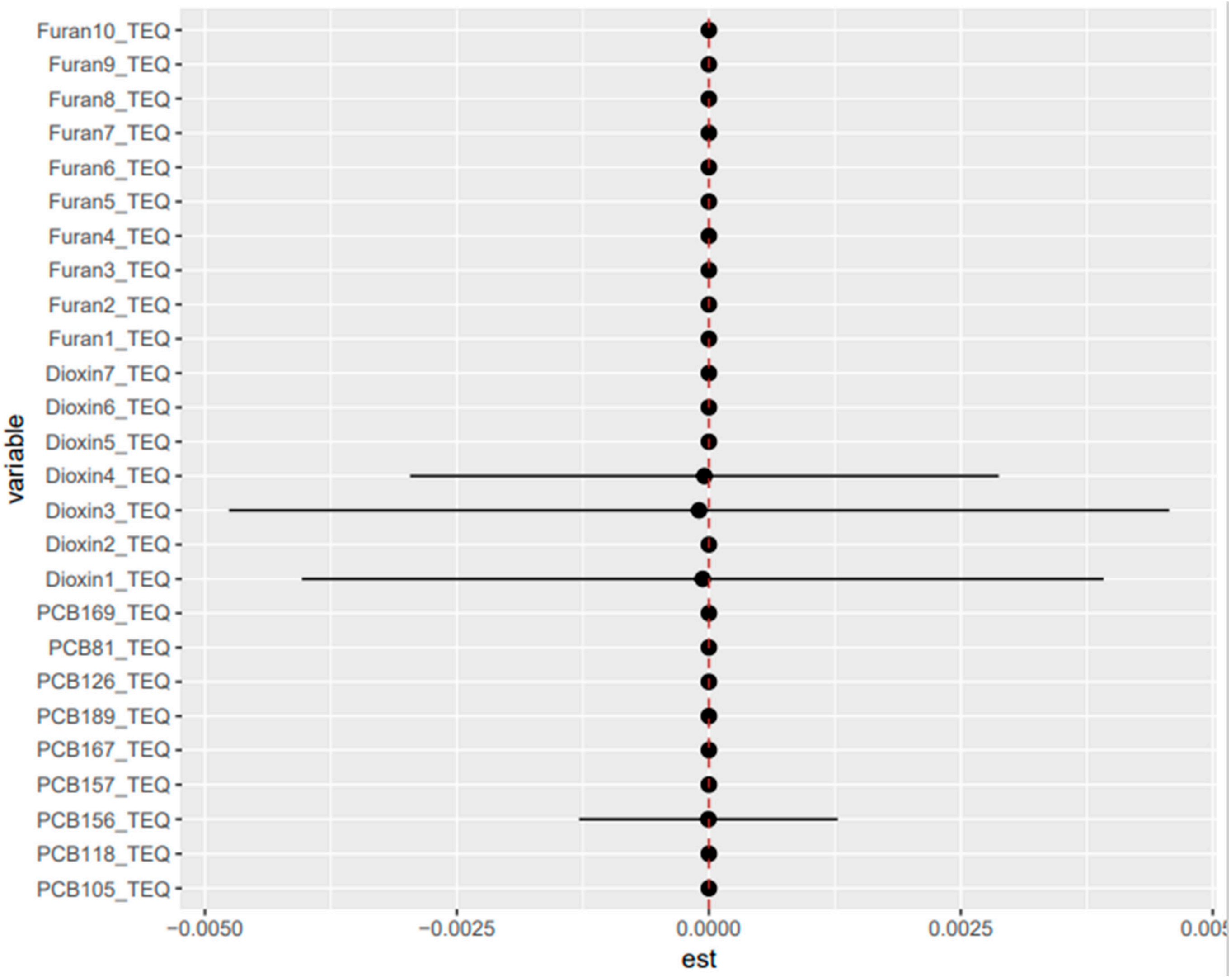

SUPPLEMENTARY DOCUMENTS

S59. ALP-TEQ

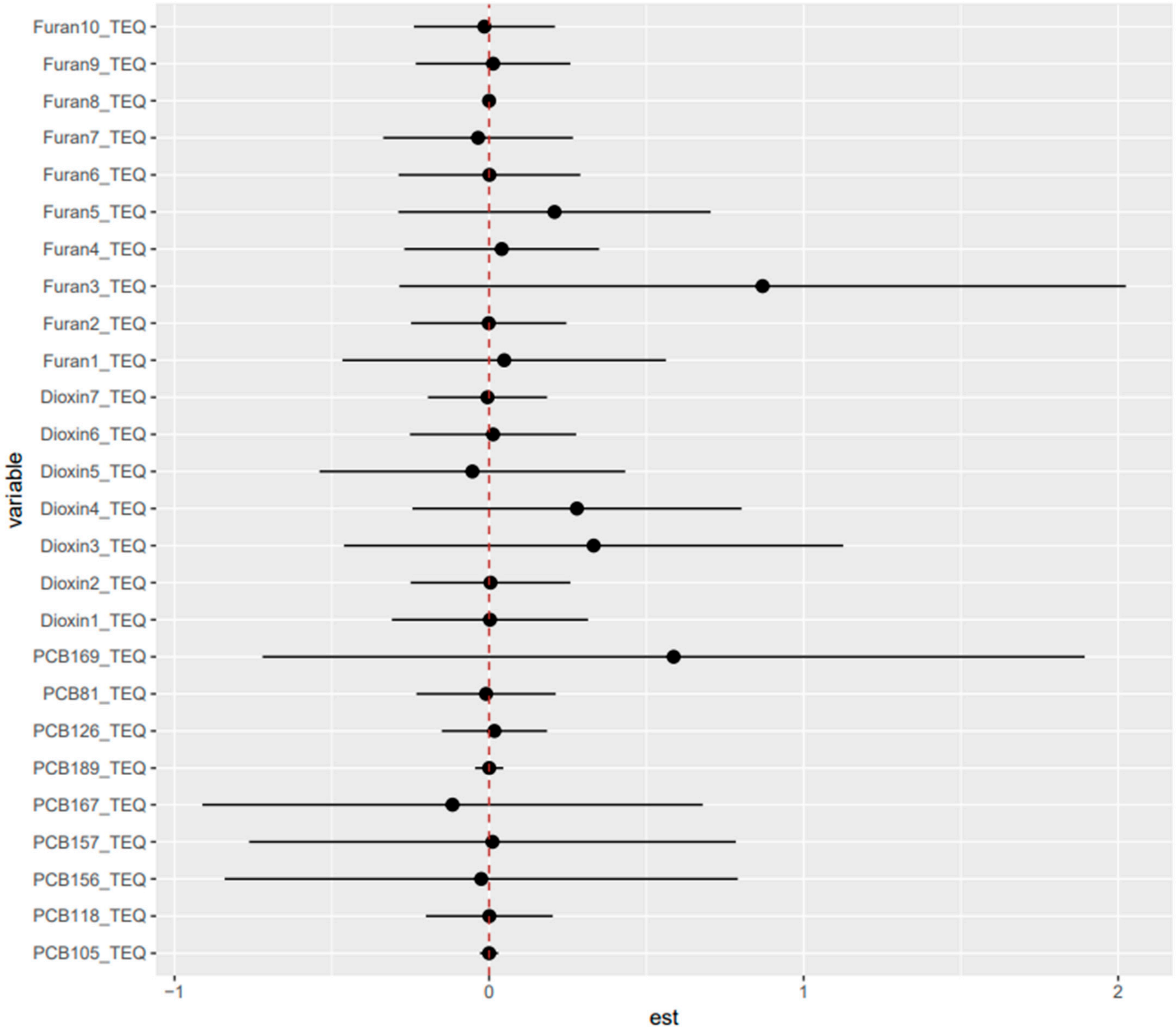

SUPPLEMENTARY DOCUMENTS

S60. ALT-TEQ

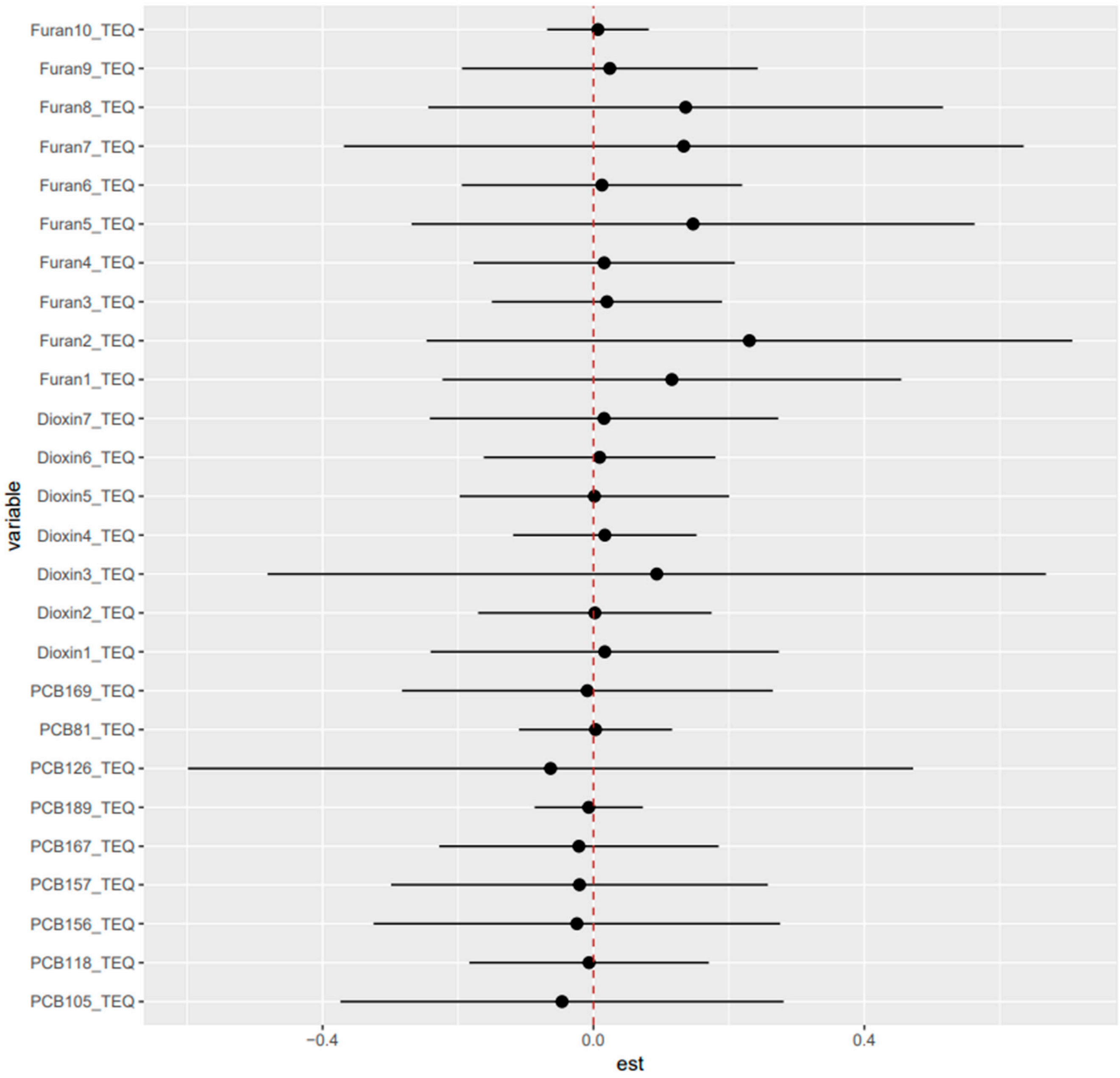

SUPPLEMENTARY DOCUMENTS

S61. AST-TEQ

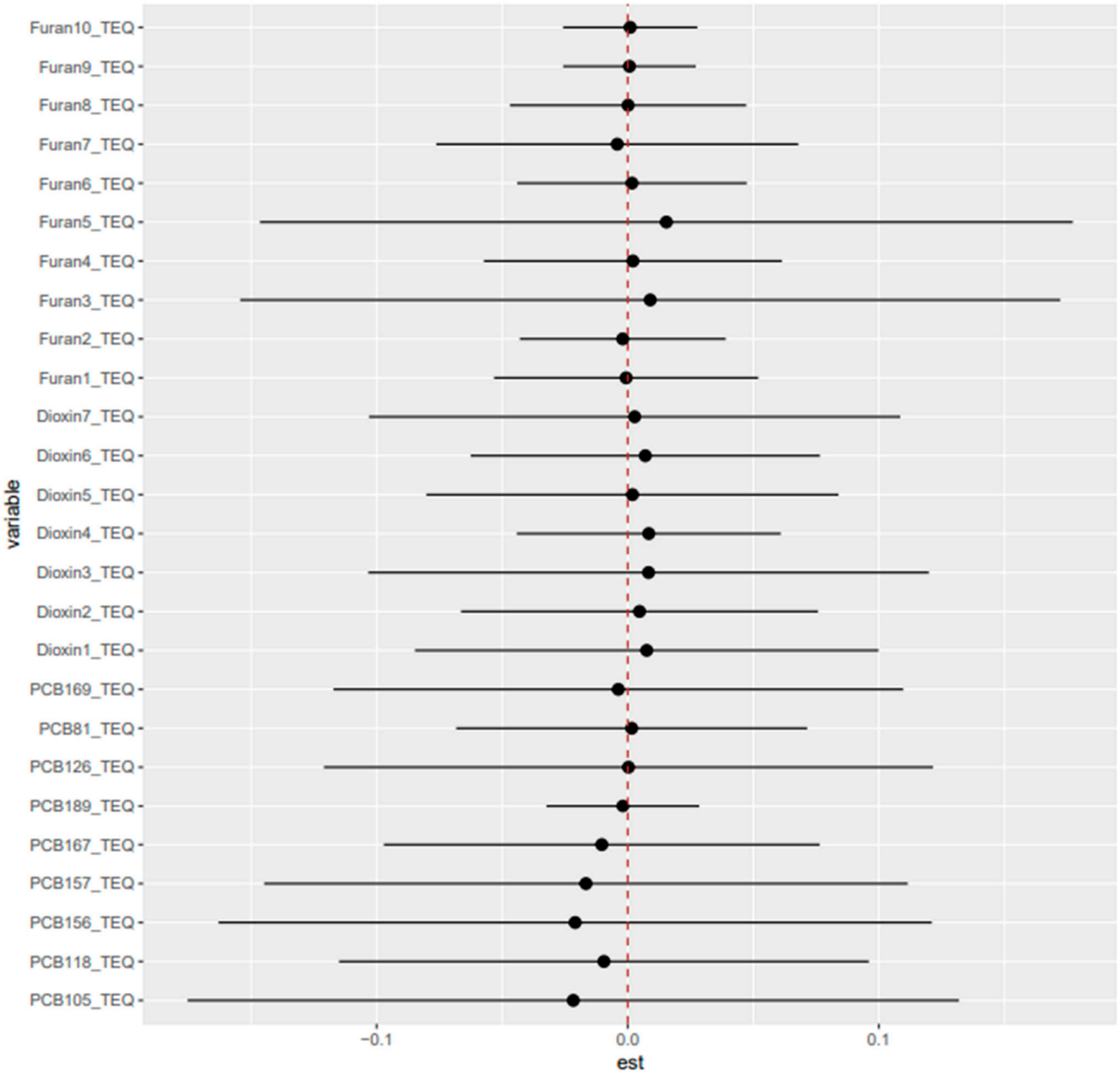

SUPPLEMENTARY DOCUMENTS

S62. GGT-TEQ

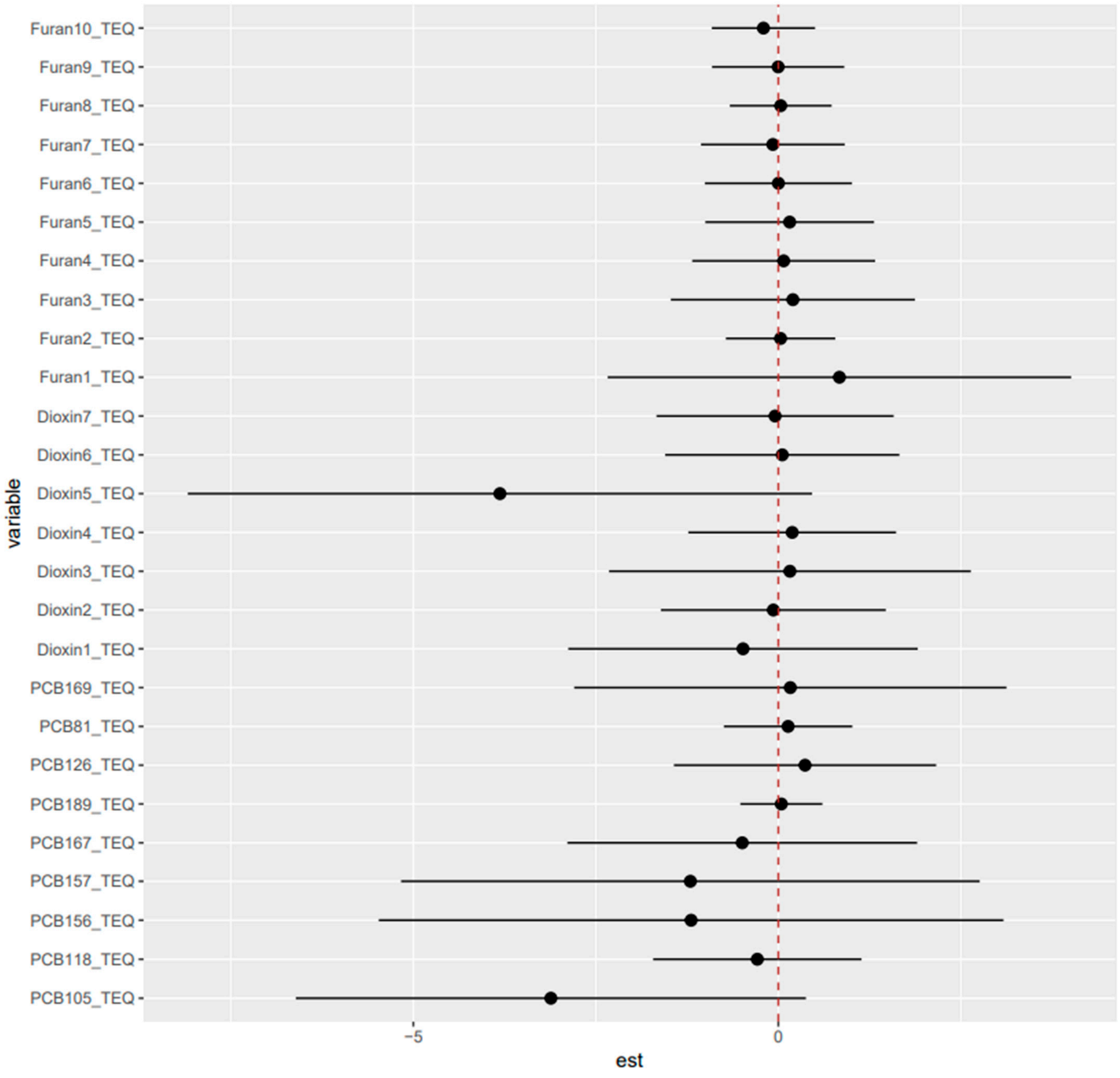

SUPPLEMENTARY DOCUMENTS

S63. LDH-TEQ

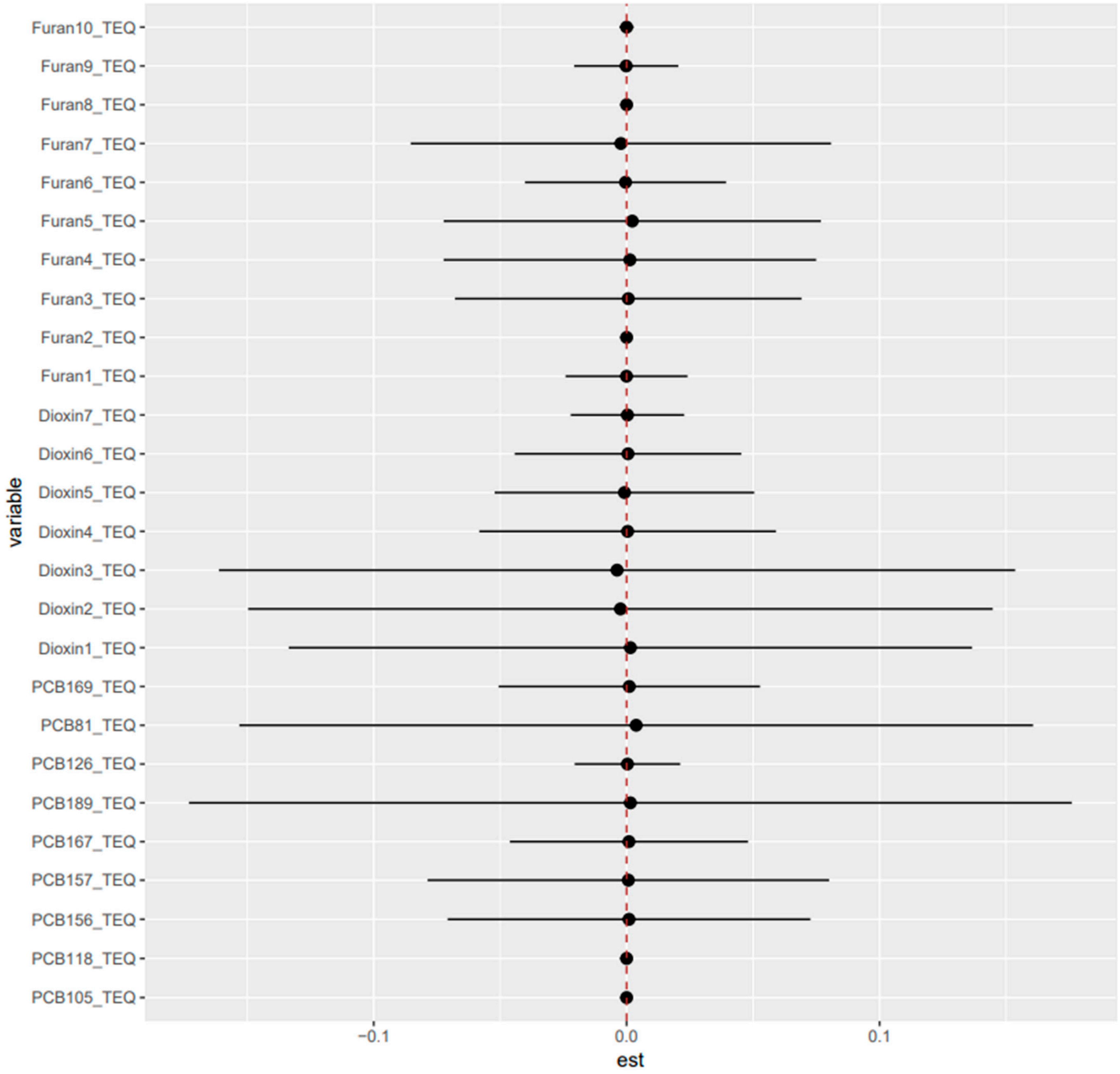

SUPPLEMENTARY DOCUMENTS

S64. TB-TEQ

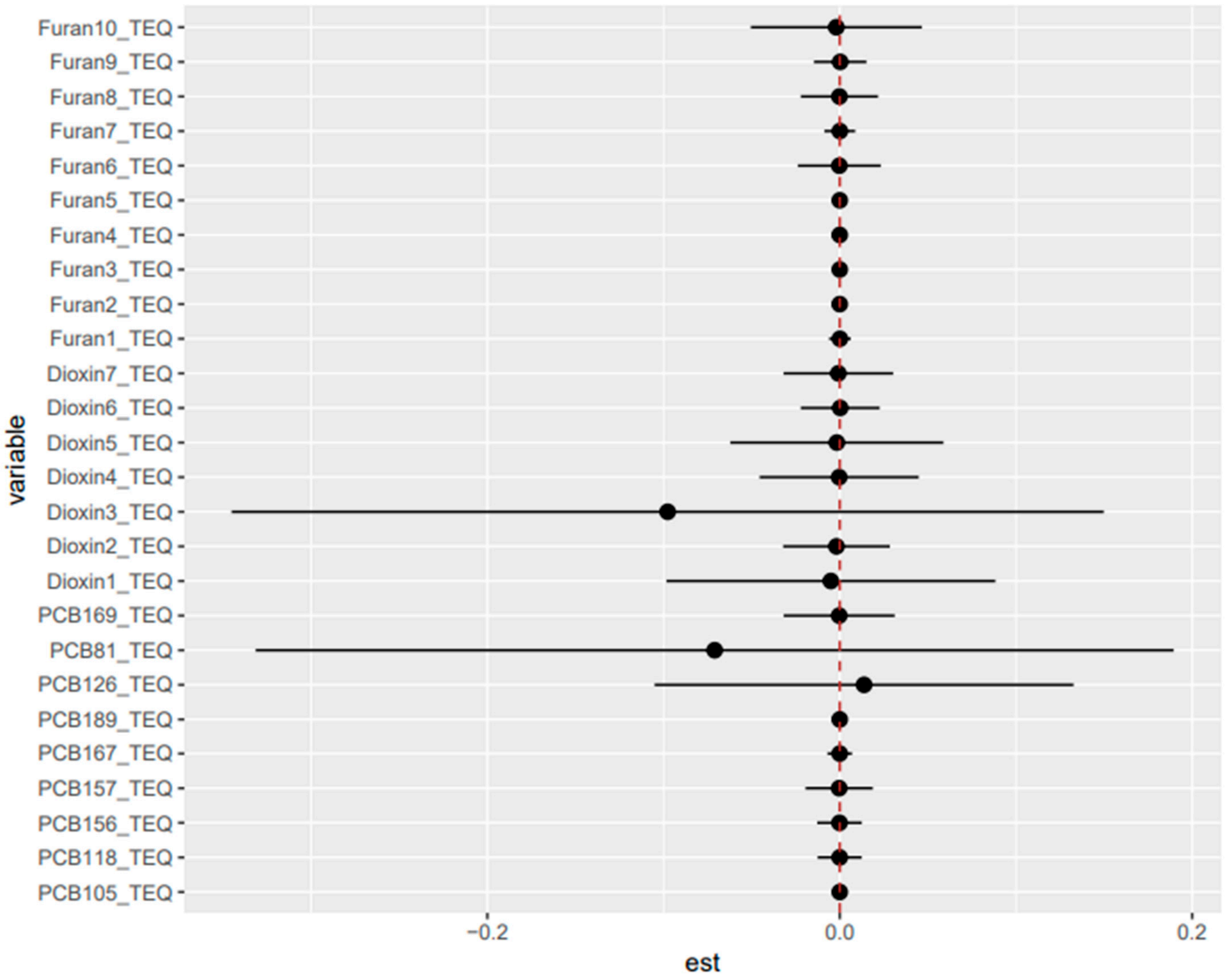

SUPPLEMENTARY DOCUMENTS

S65. TP-TEQ

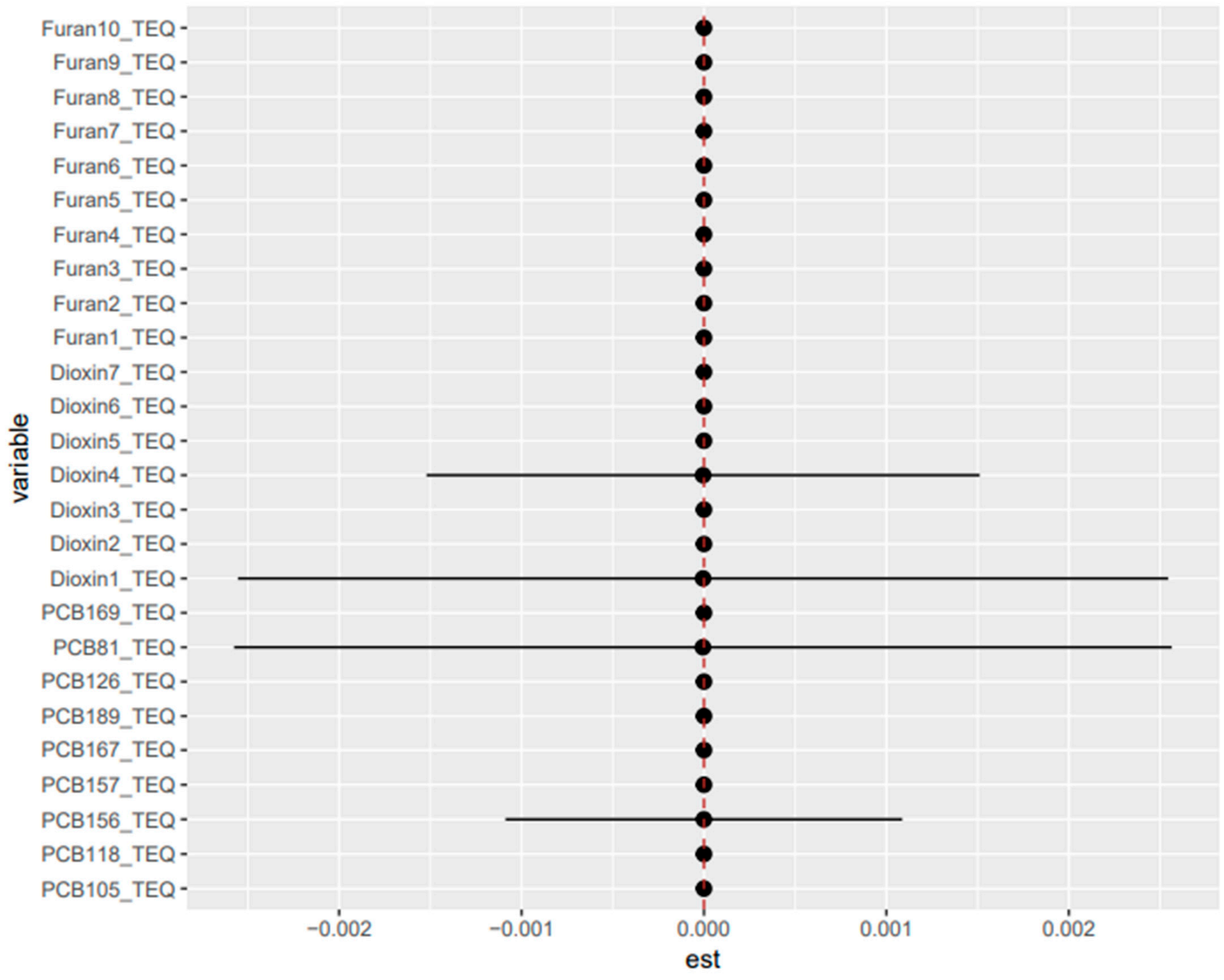

SUPPLEMENTARY DOCUMENTS

WEIGHTED QUANTILE SUM (WQS) REGRESSION RESULTS (MAIN EFFECT)

S66. ALBUMIN

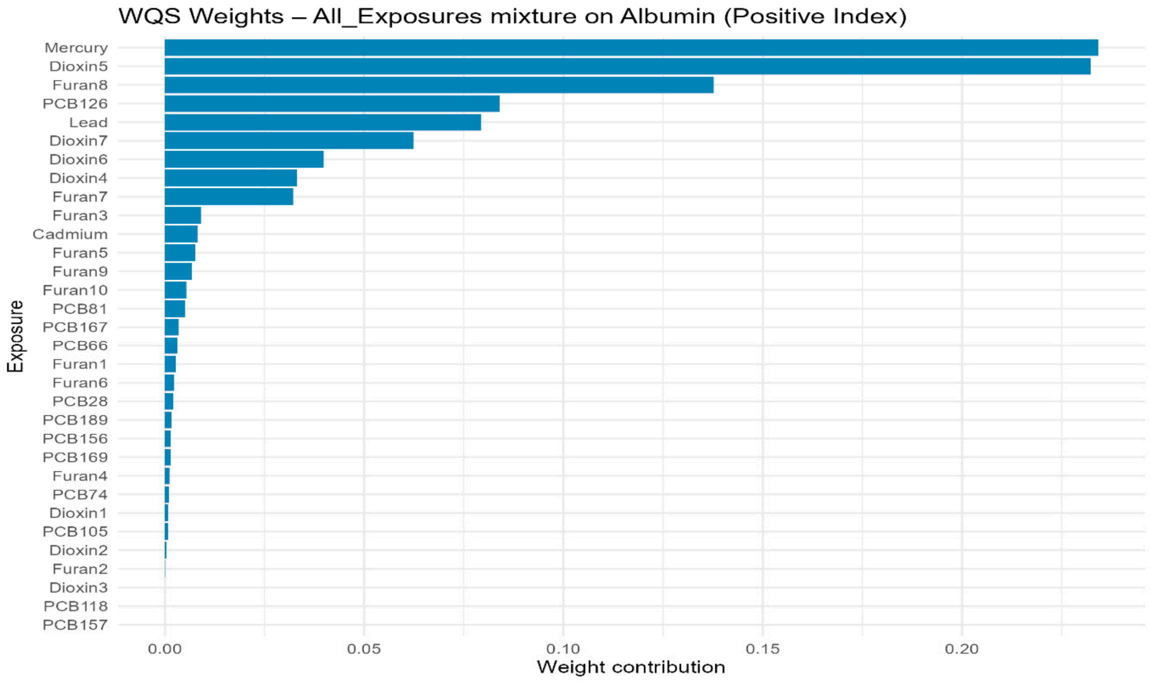

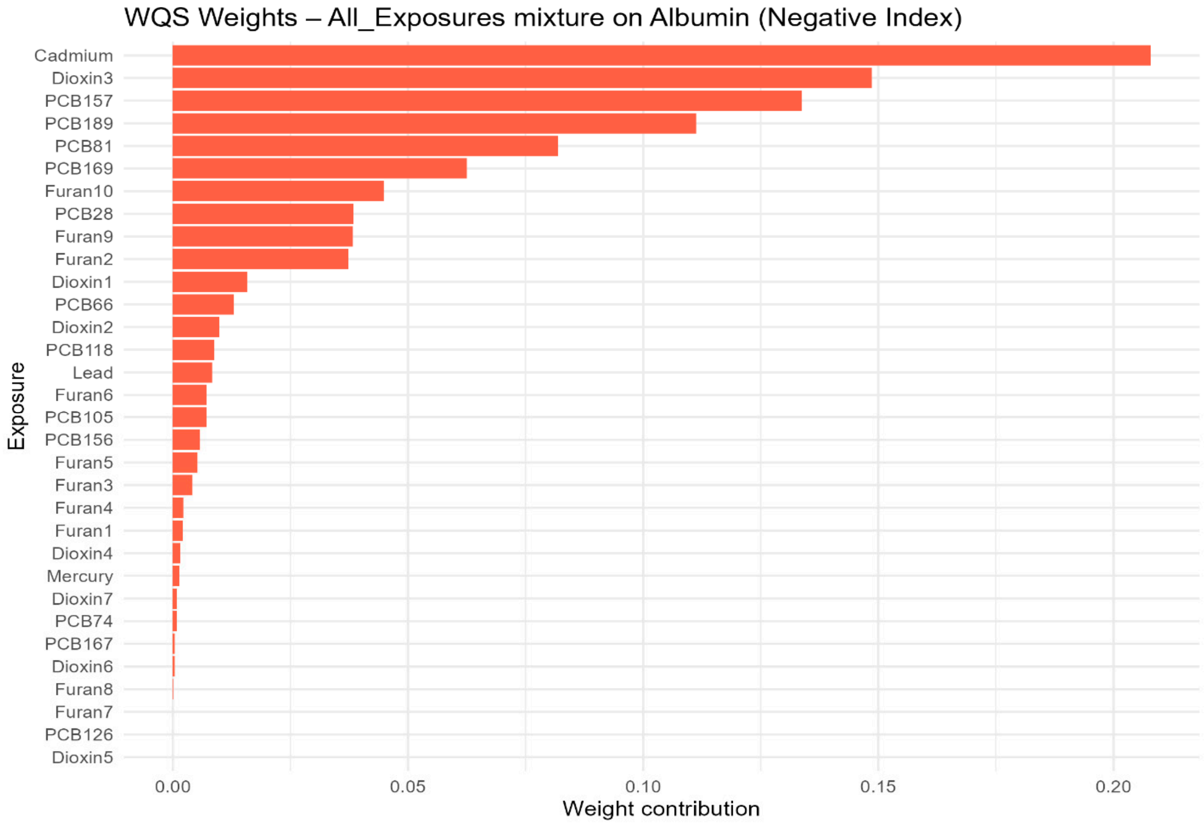

SUPPLEMENTARY DOCUMENTS

S67. ALP

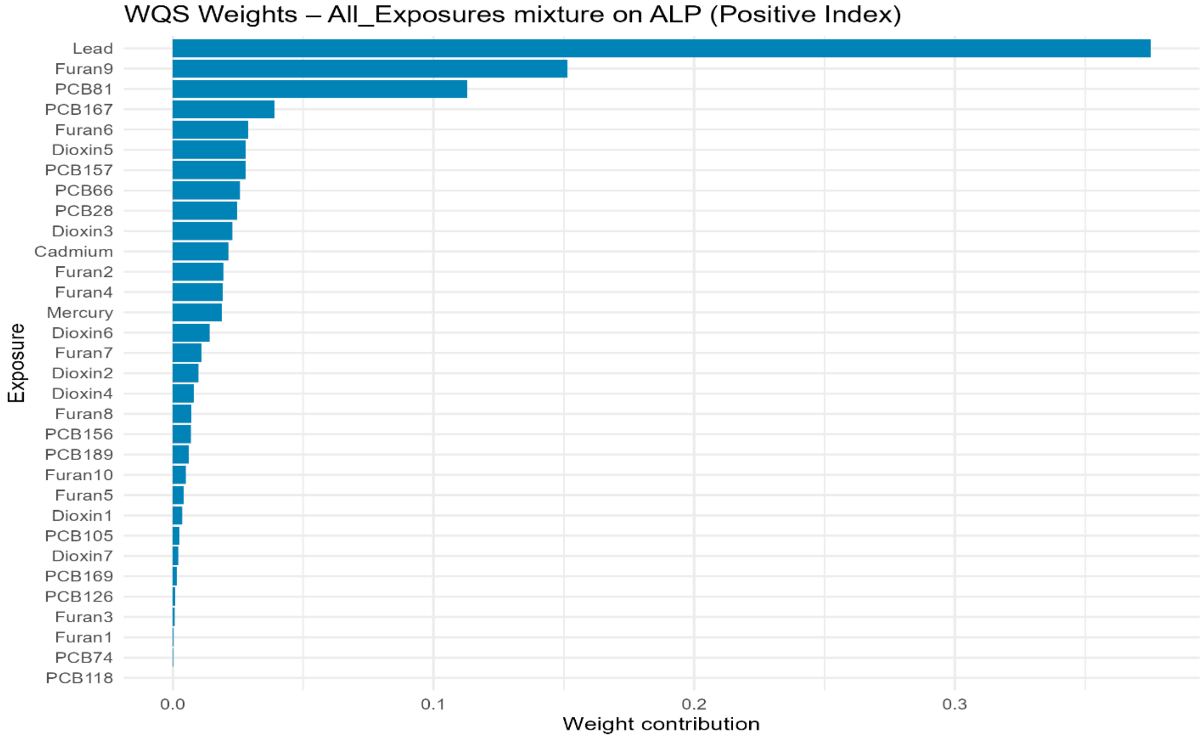

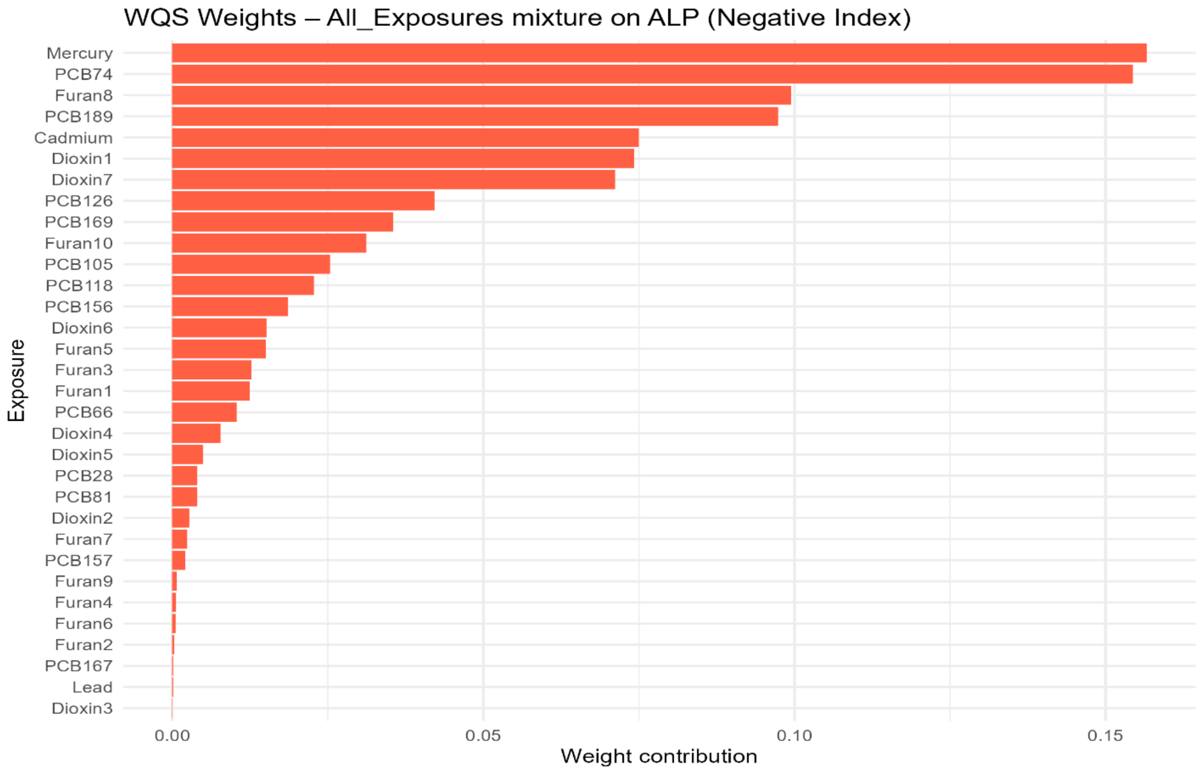

SUPPLEMENTARY DOCUMENTS

S68. ALT

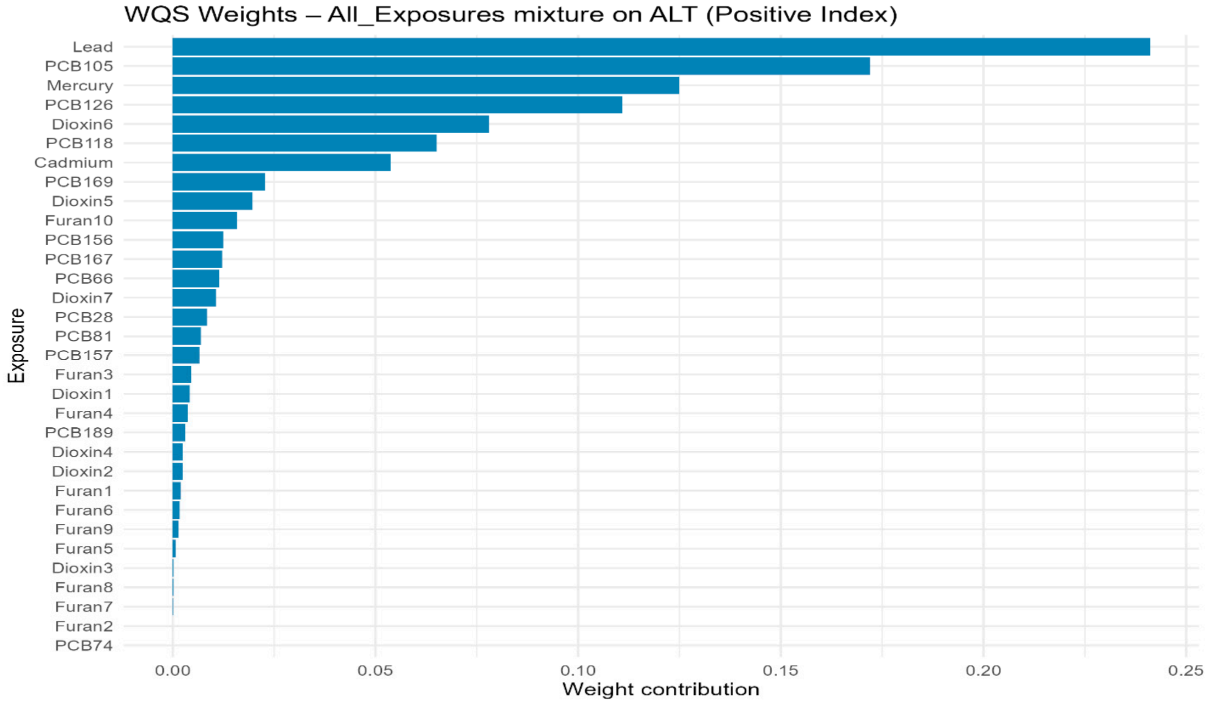

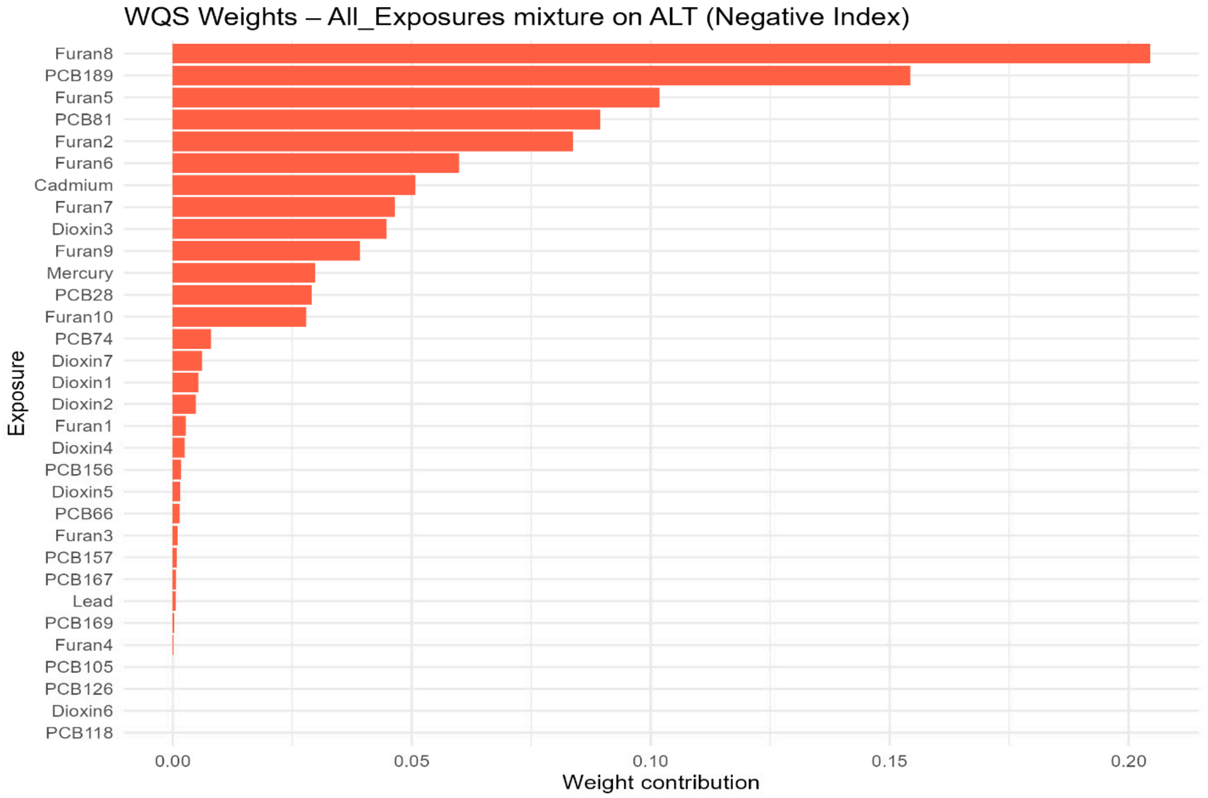

SUPPLEMENTARY DOCUMENTS

S69. AST

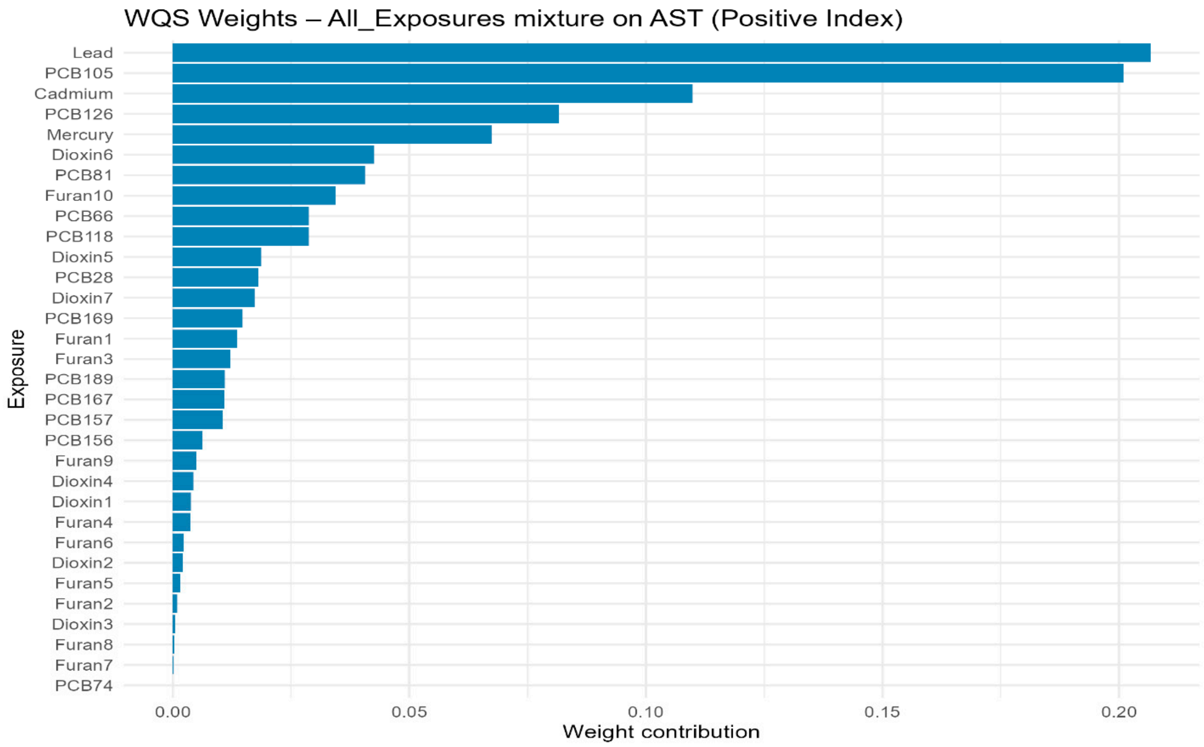

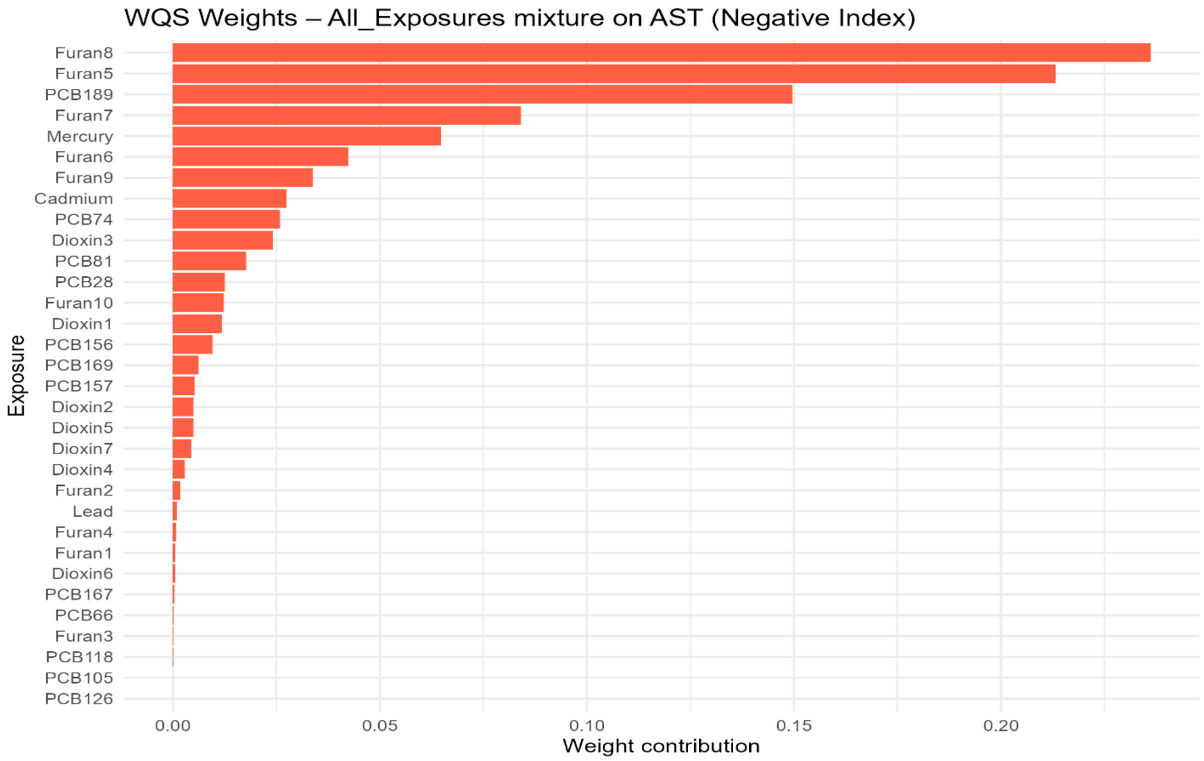

SUPPLEMENTARY DOCUMENTS

S70. GGT

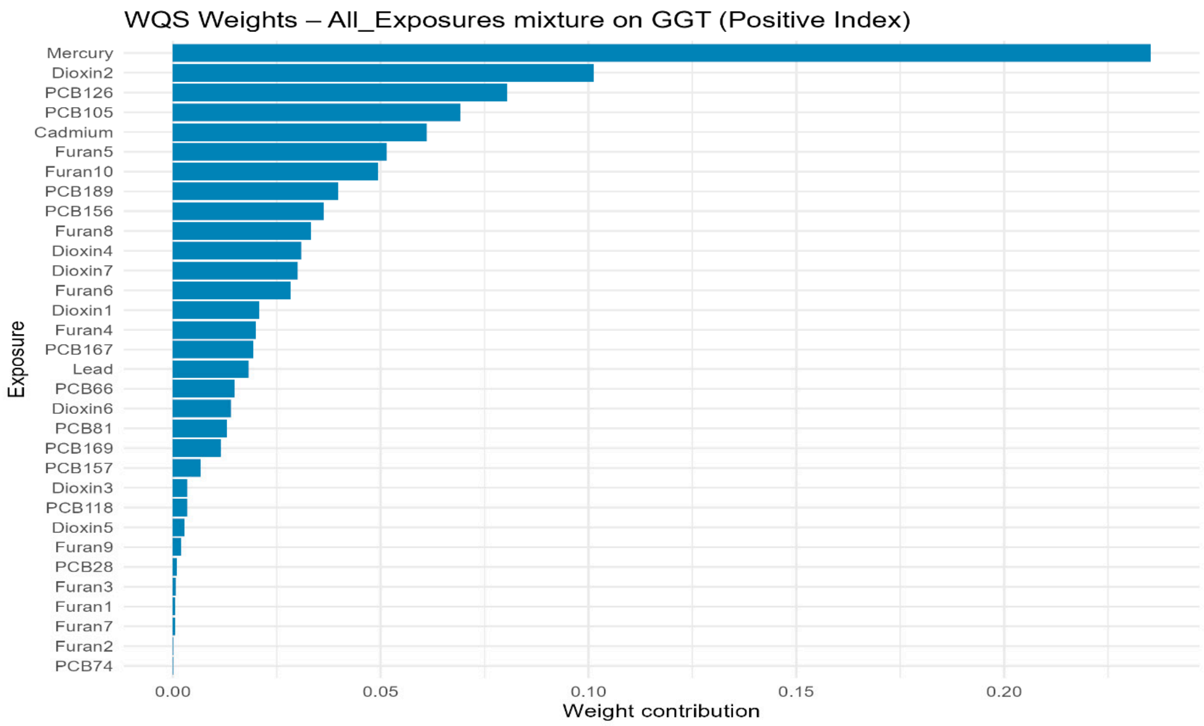

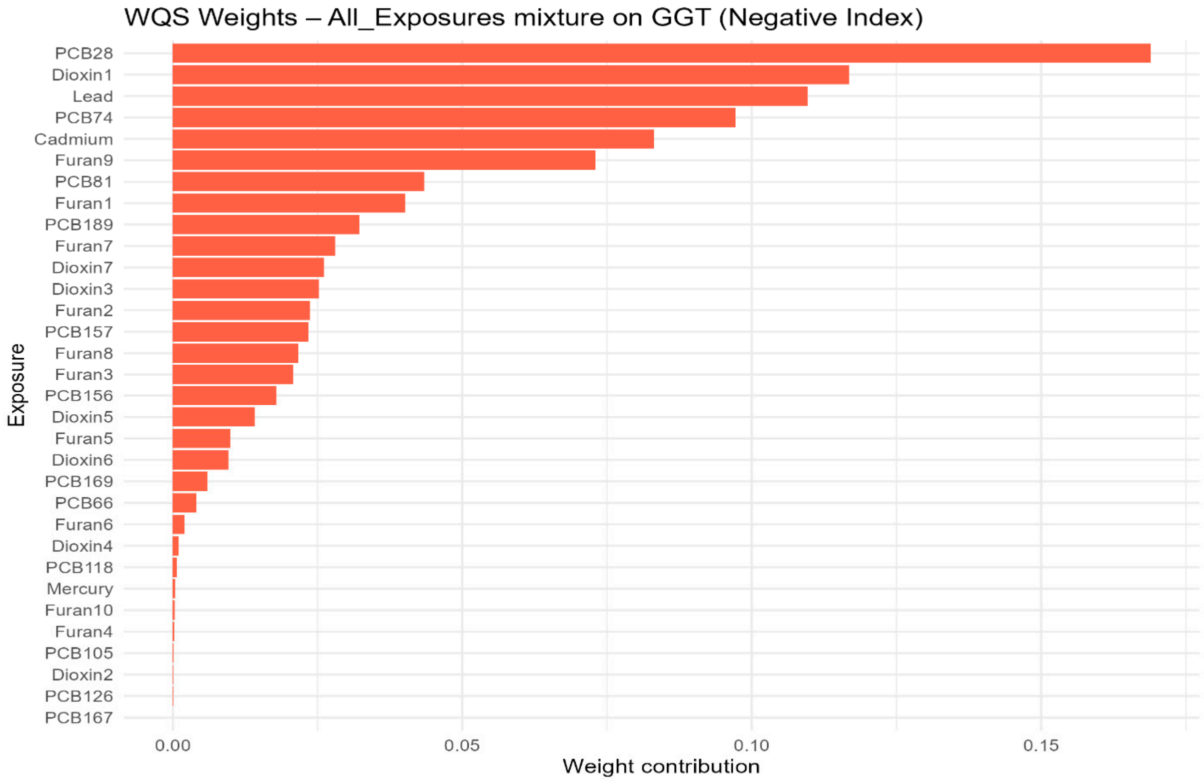

SUPPLEMENTARY DOCUMENTS

S71. LDH

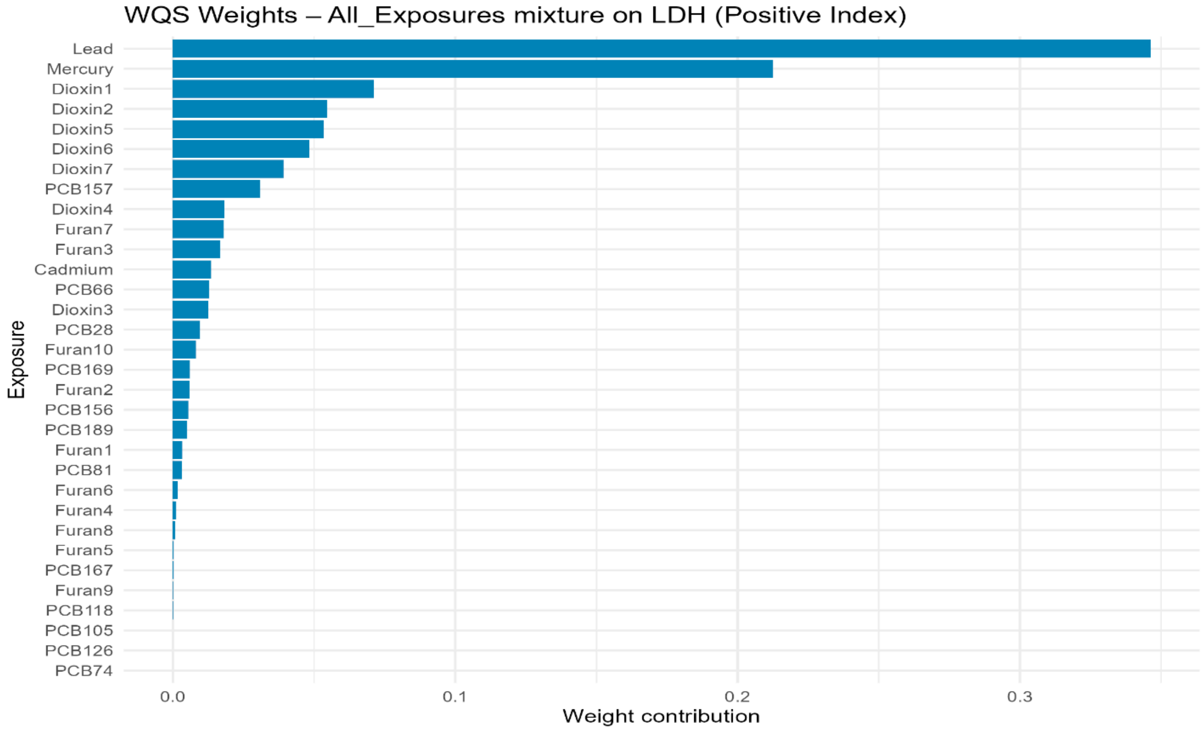

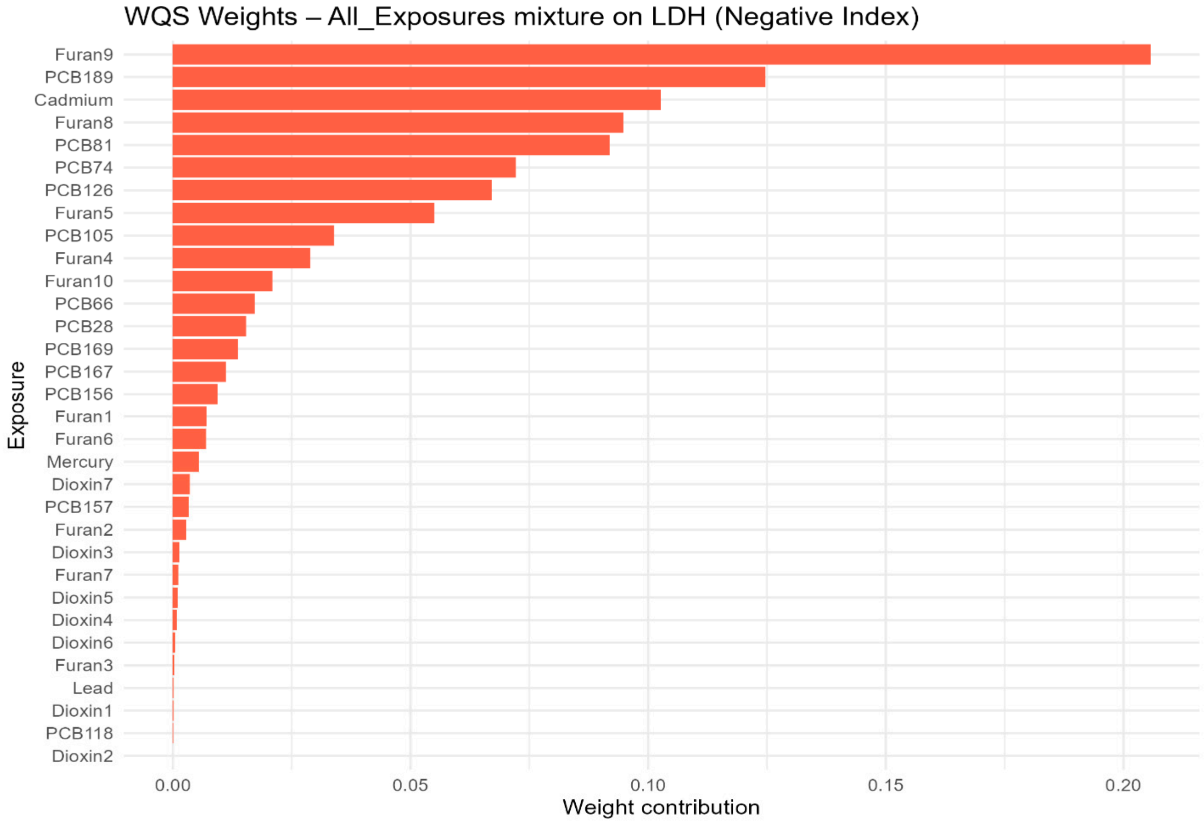

SUPPLEMENTARY DOCUMENTS

S72. TOTAL BILIRUBIN

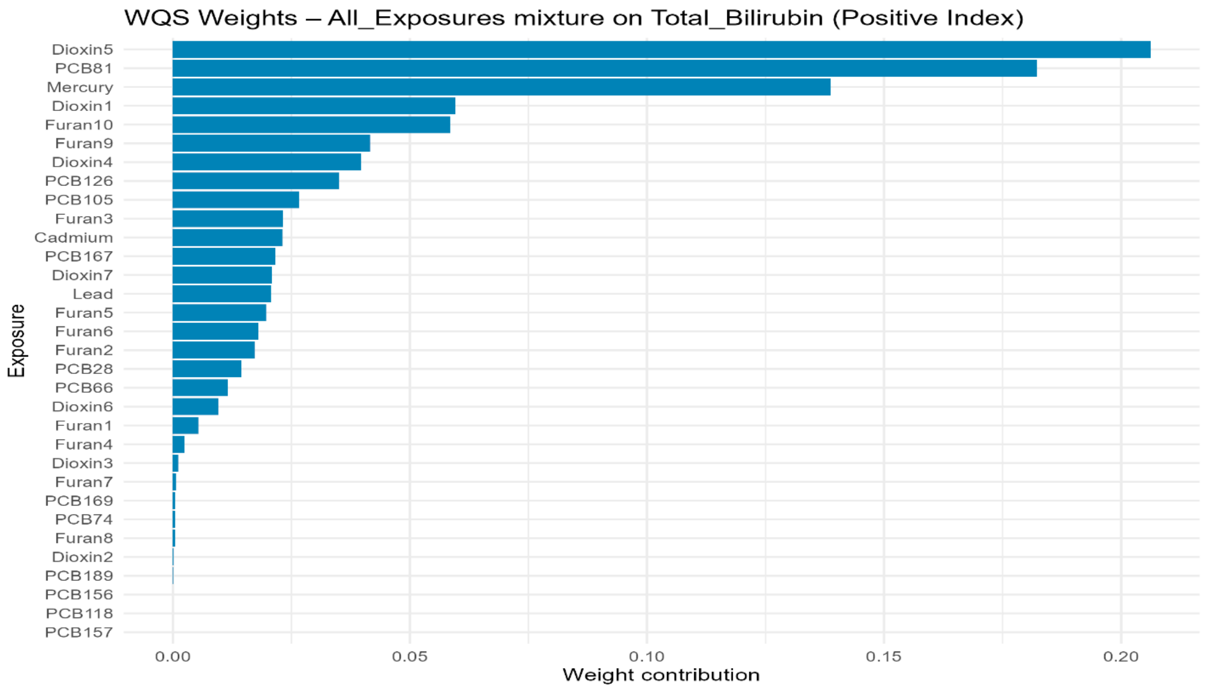

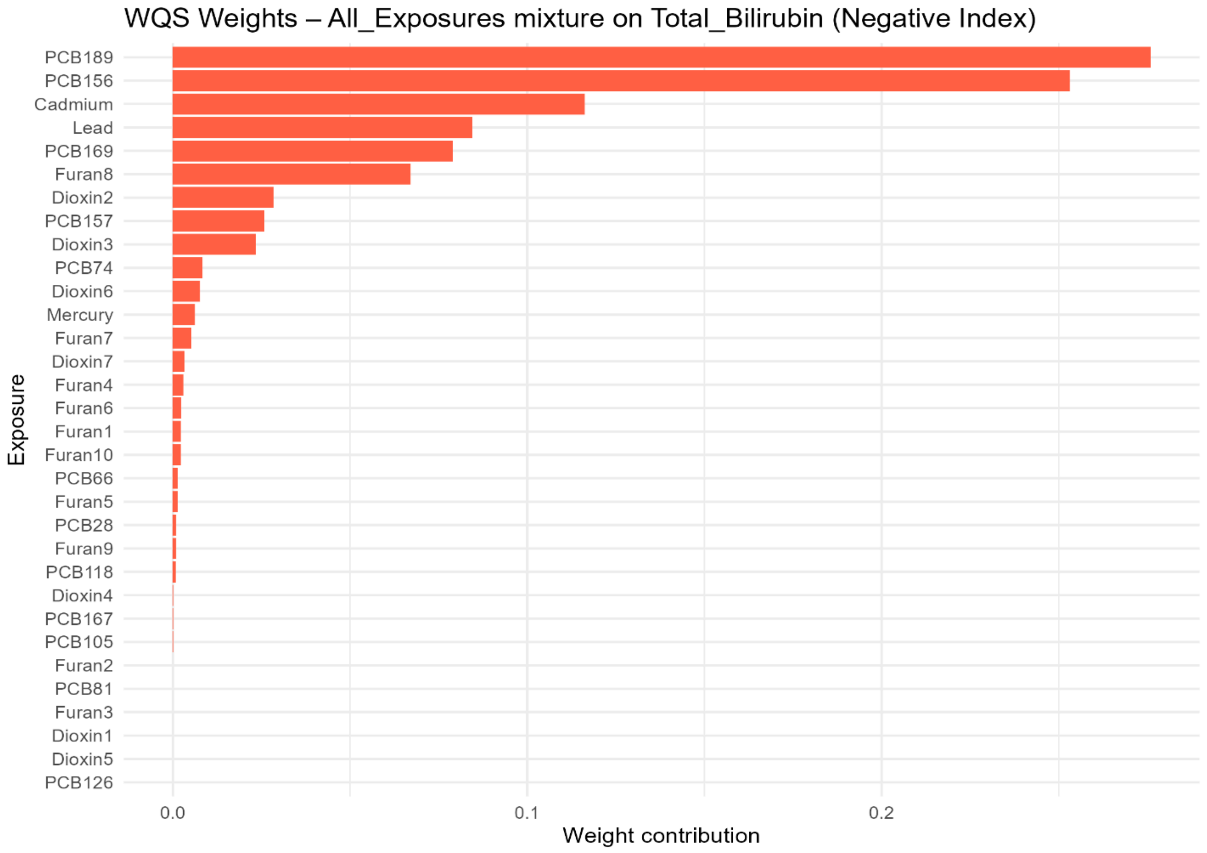

SUPPLEMENTARY DOCUMENTS

S73. TOTAL PROTEIN

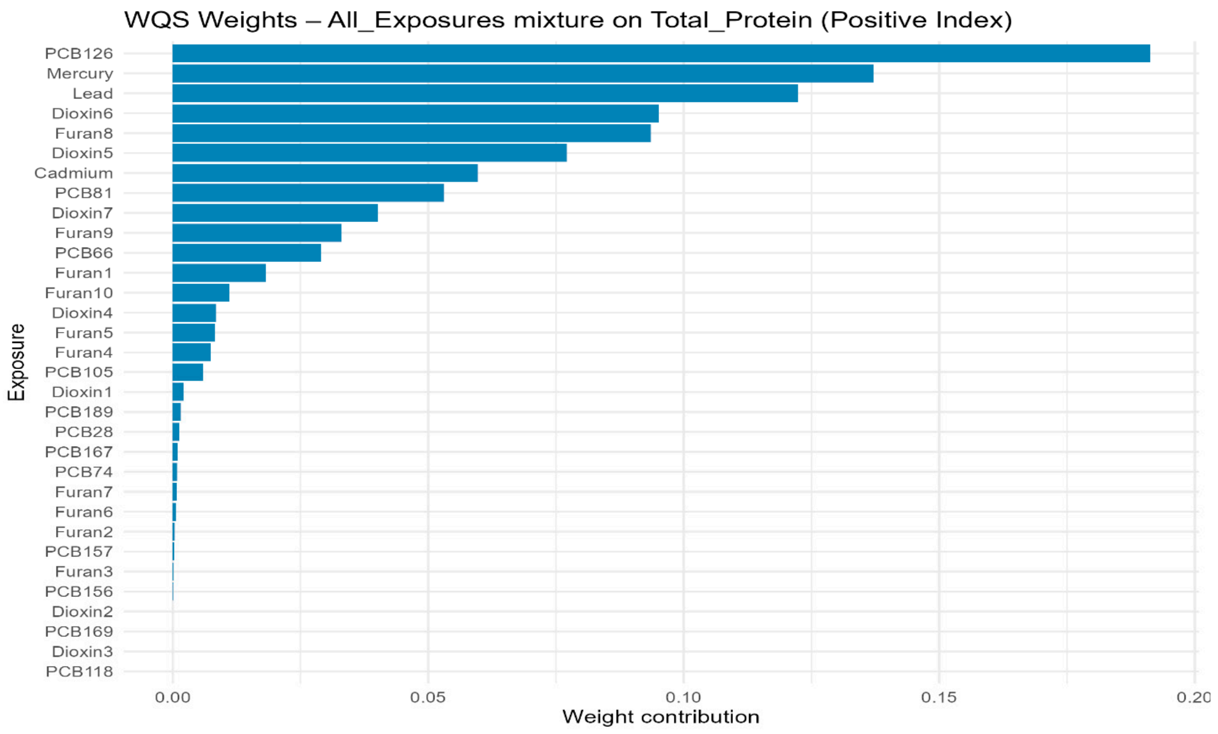

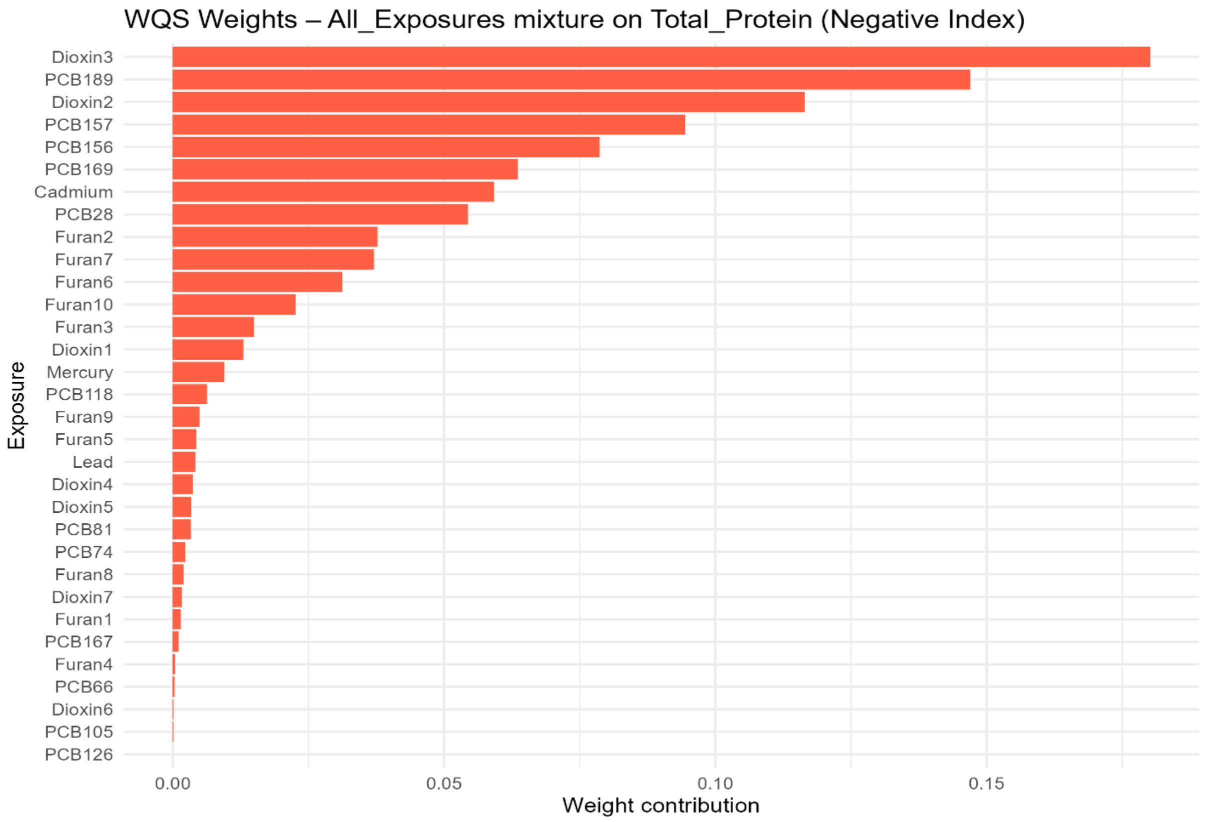

SUPPLEMENTARY DOCUMENTS

WEIGHTED QUANTILE SUM (WQS) REGRESSION RESULTS (METAL AND DIOXIN-LIKE TEQ)

S74. ALBUMIN

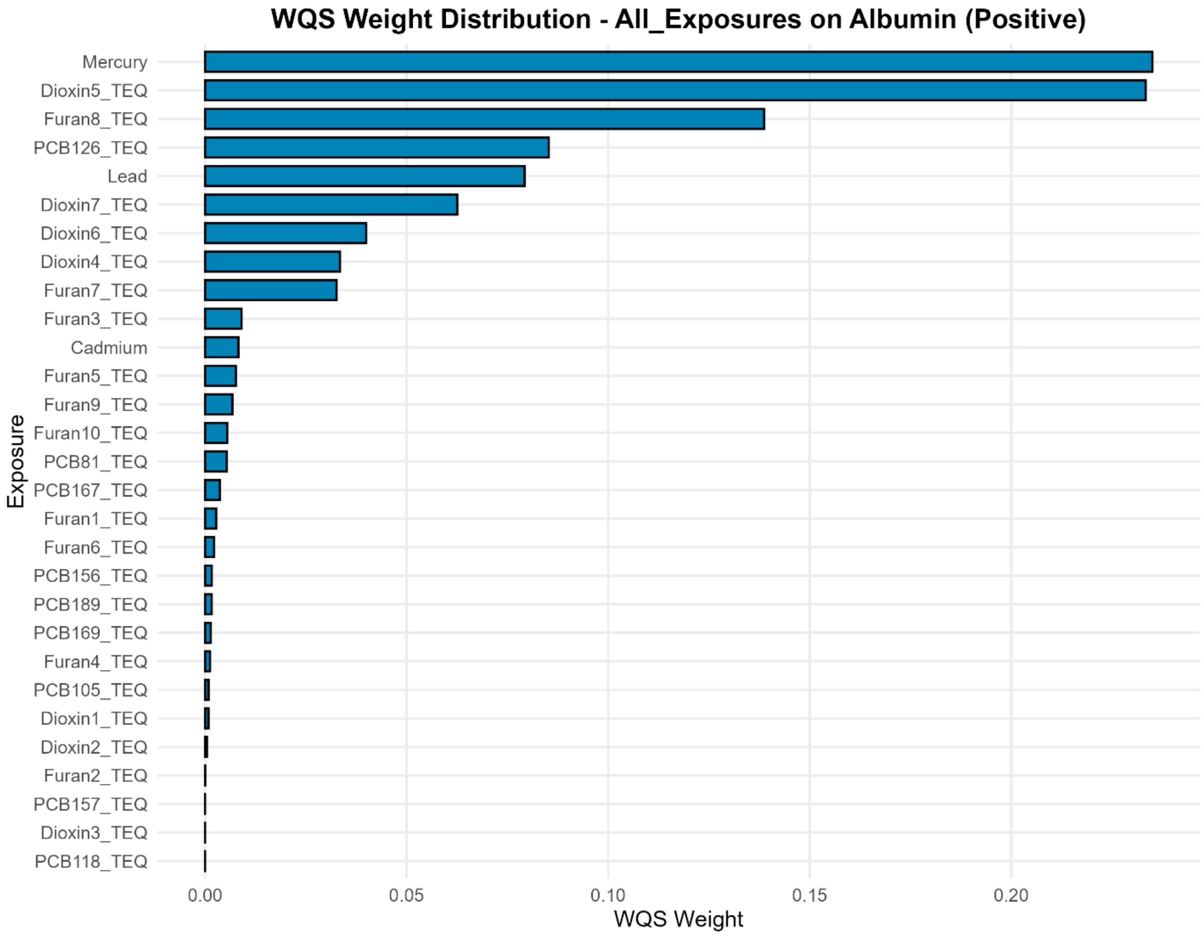

SUPPLEMENTARY DOCUMENTS

S75. ALP

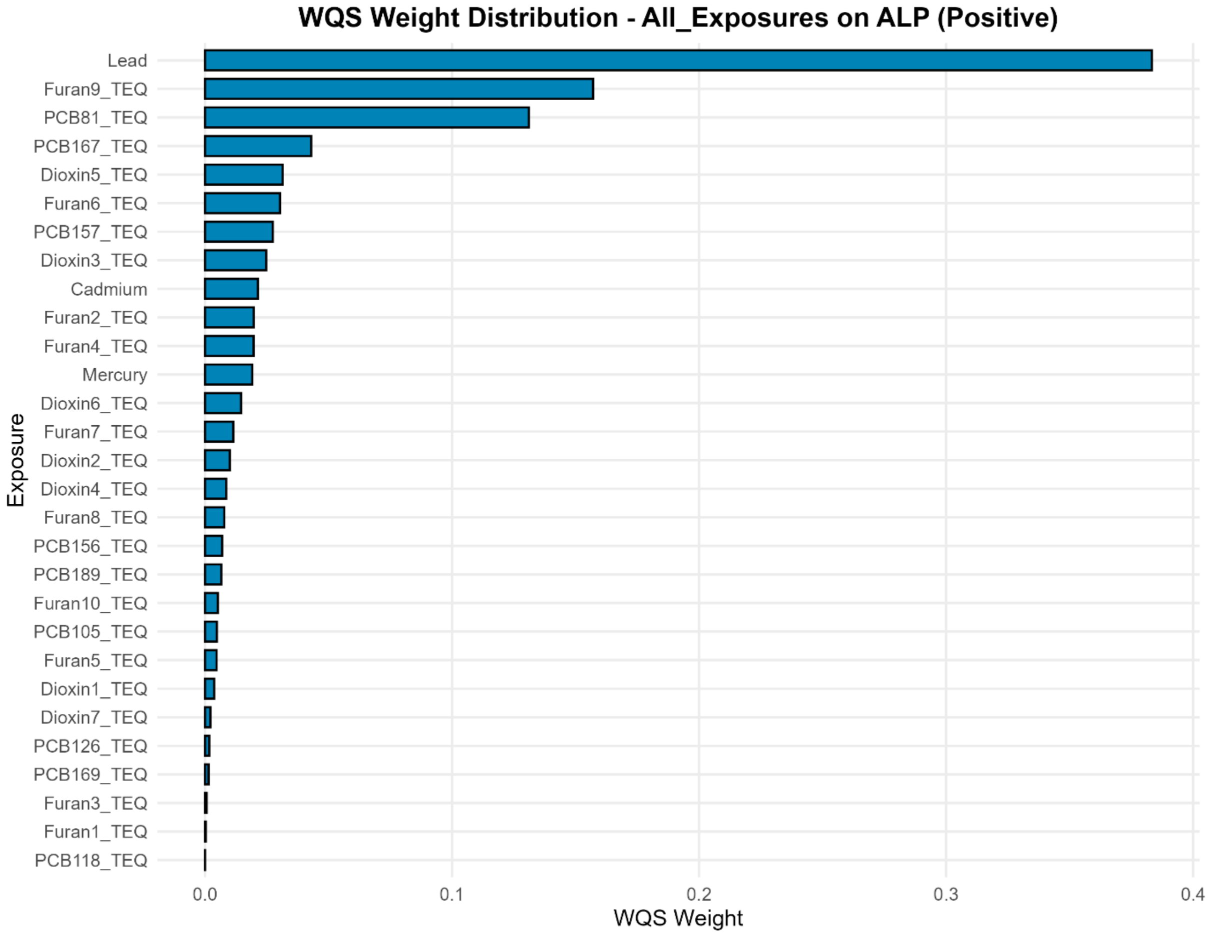

SUPPLEMENTARY DOCUMENTS

S76. ALT

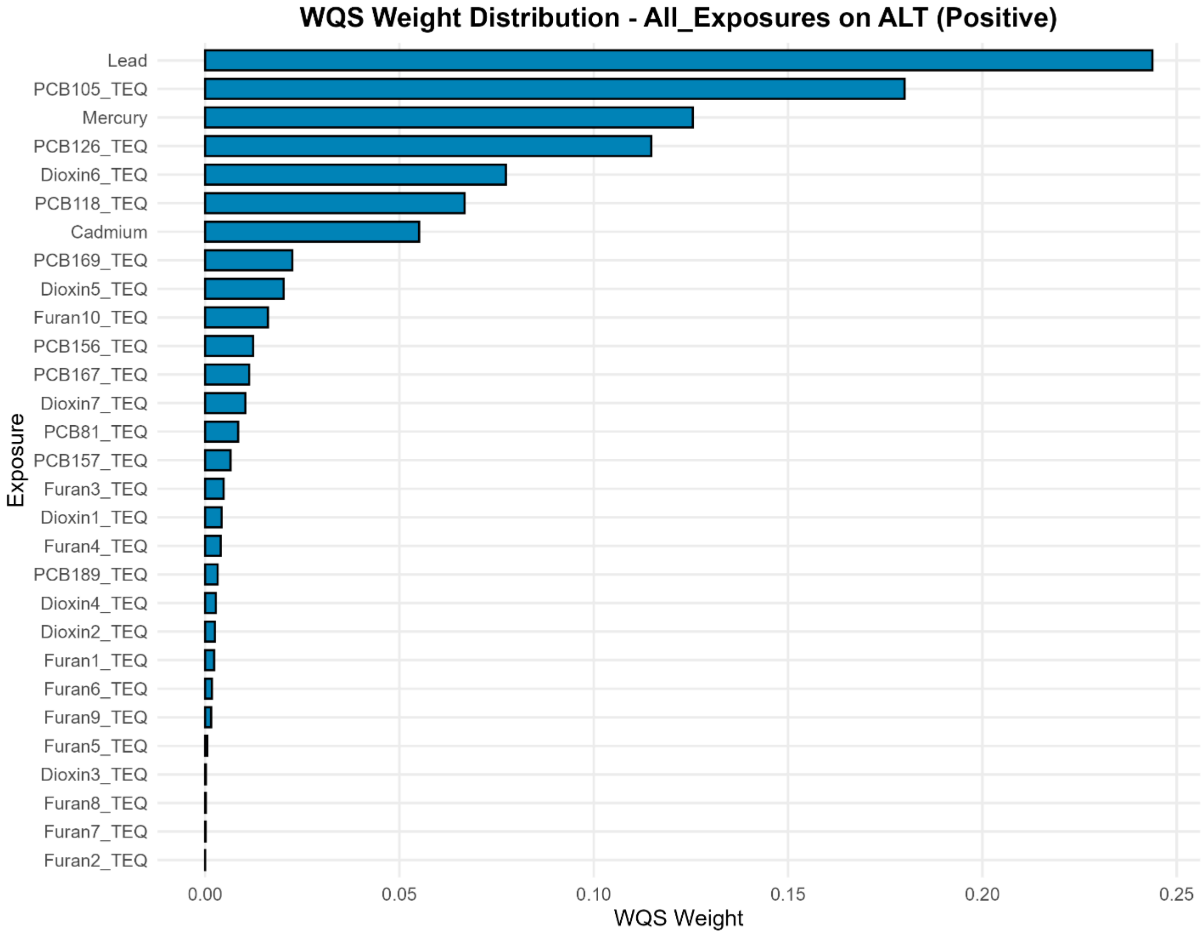

SUPPLEMENTARY DOCUMENTS

S77. AST

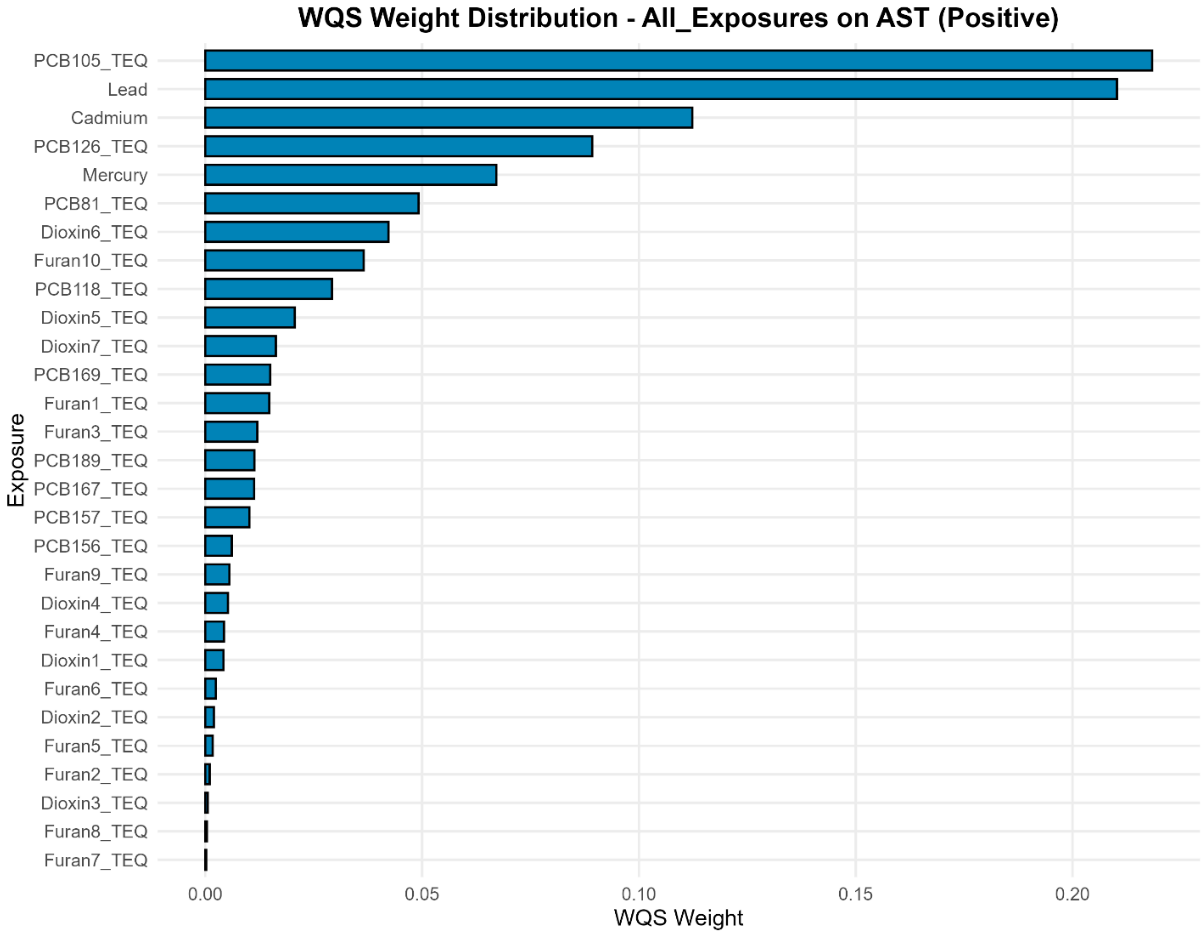

SUPPLEMENTARY DOCUMENTS

S78. GGT

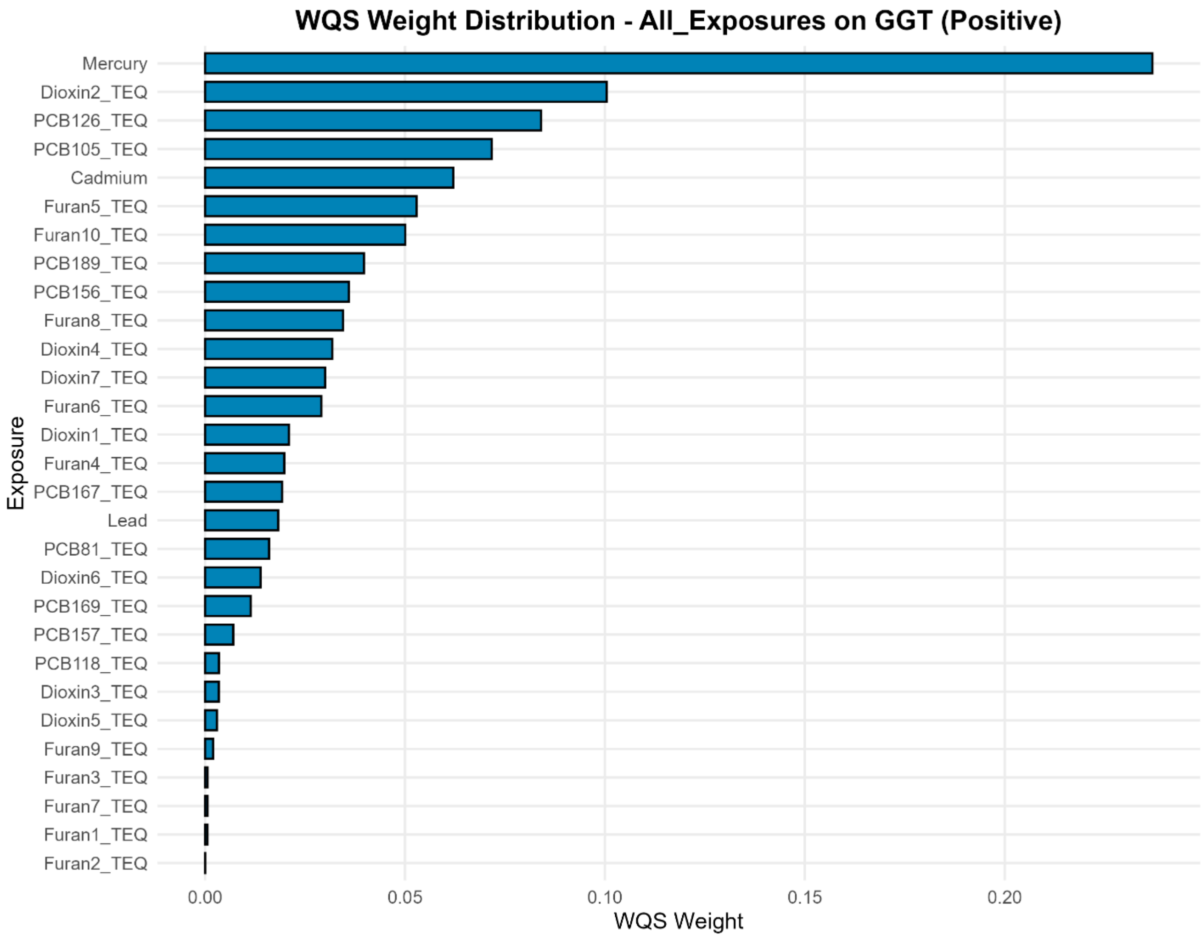

SUPPLEMENTARY DOCUMENTS

S79. LDH

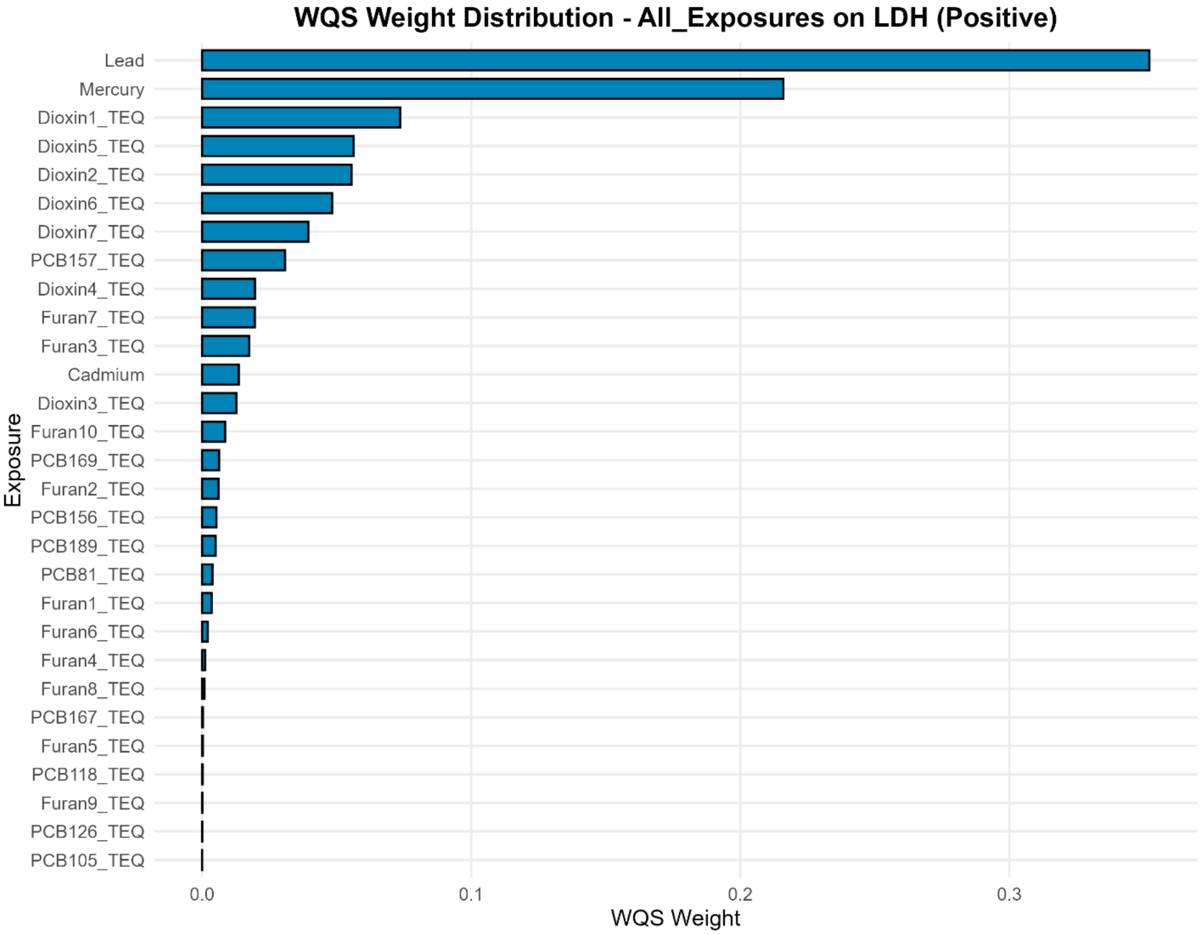

SUPPLEMENTARY DOCUMENTS

S80. TOTAL BILIRUBIN

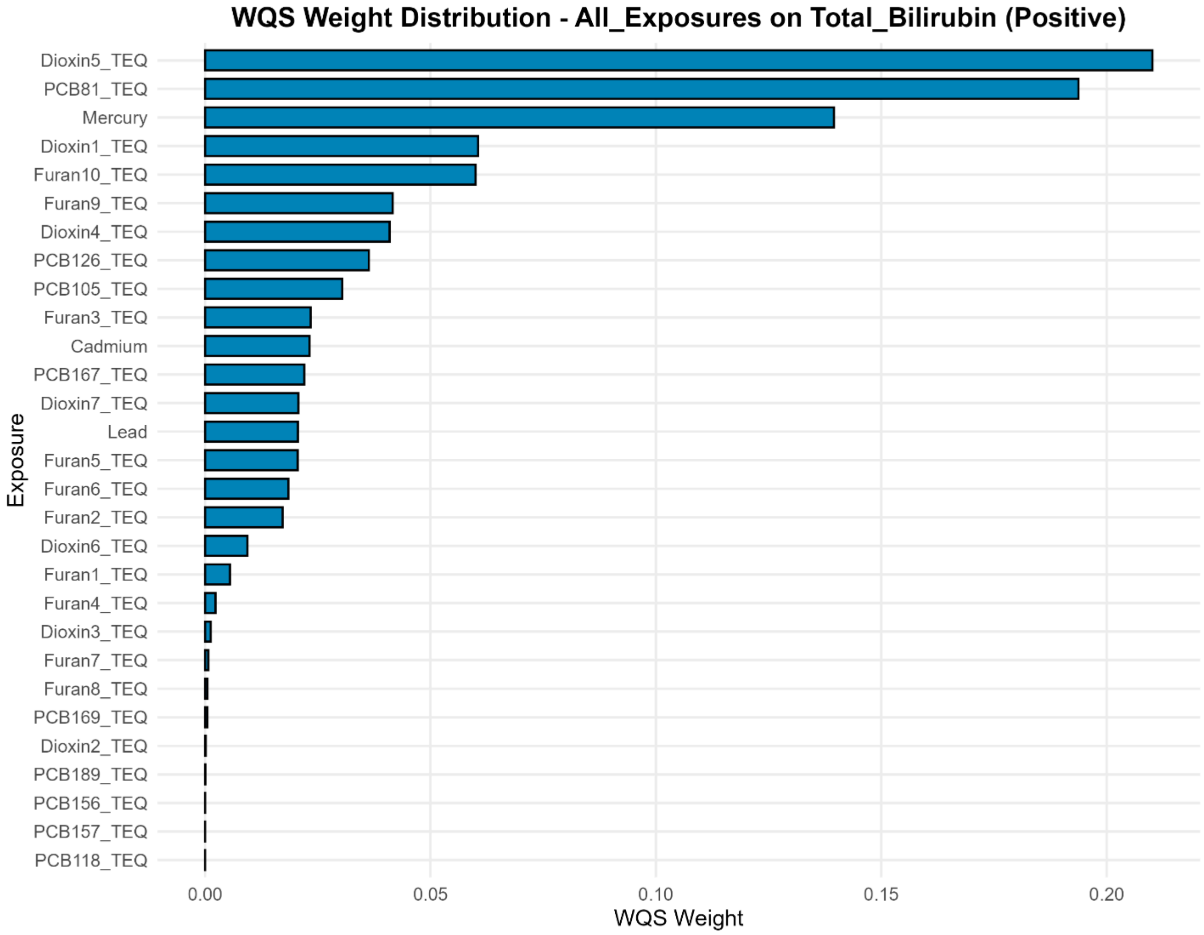

SUPPLEMENTARY DOCUMENTS

S81. TOTAL PROTEIN

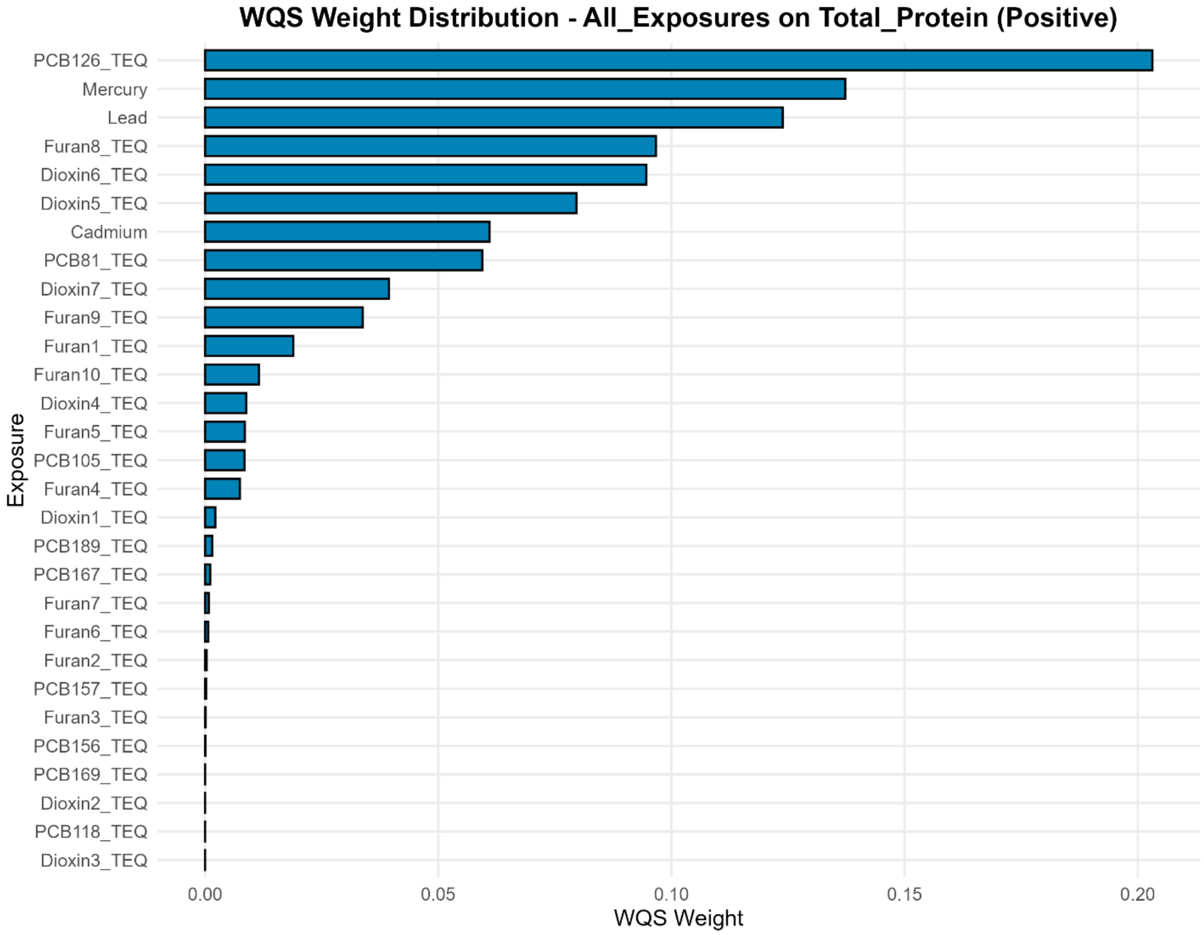

SUPPLEMENTARY DOCUMENTS

QUANTILE G-COMPUTATION RESULTS

S82. ALBUMIN

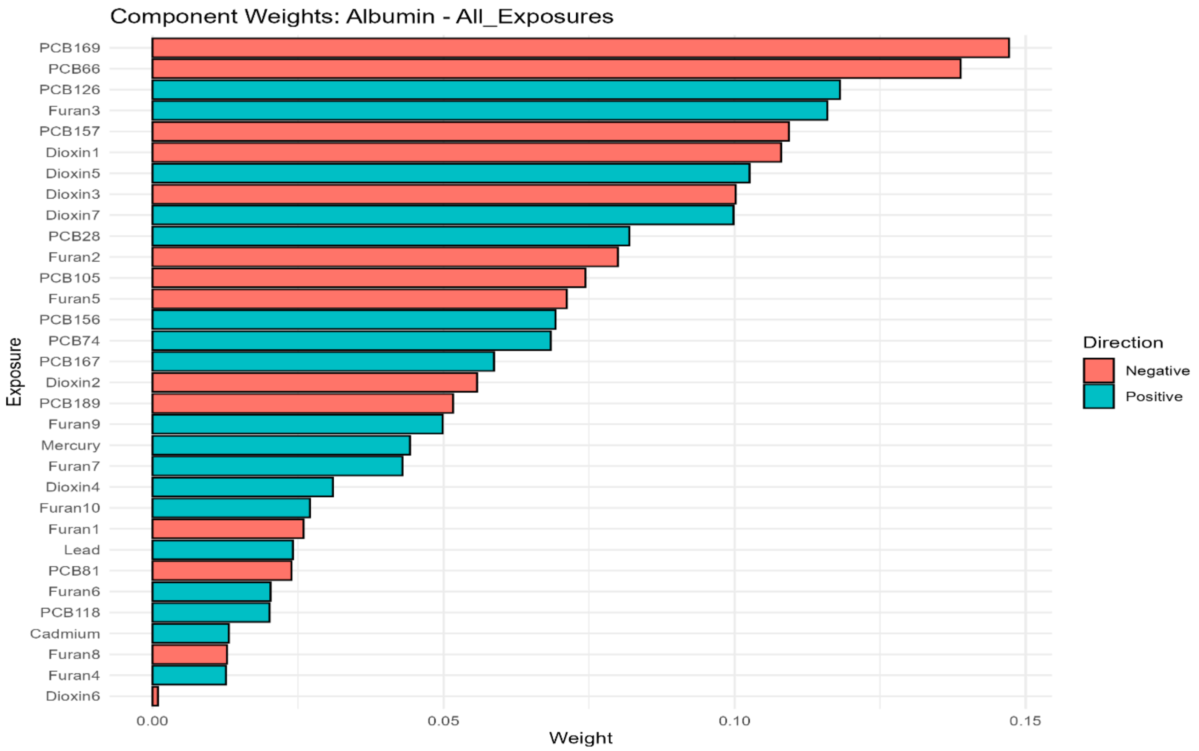

SUPPLEMENTARY DOCUMENTS

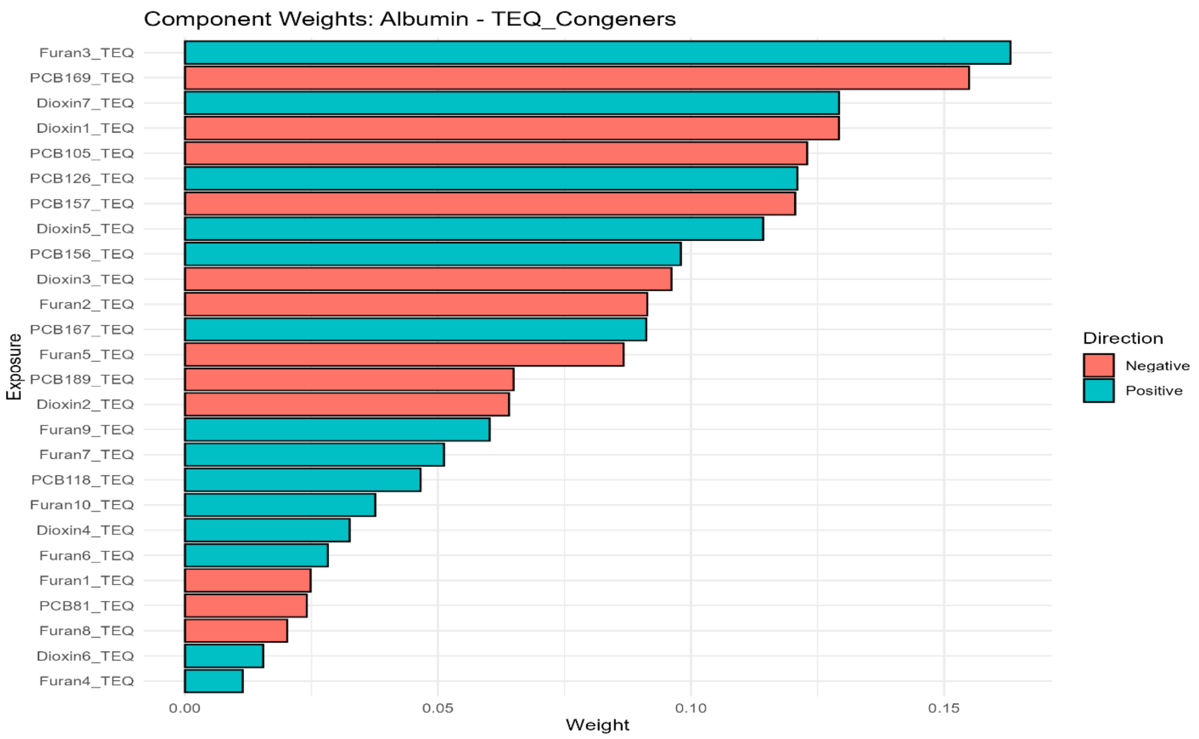

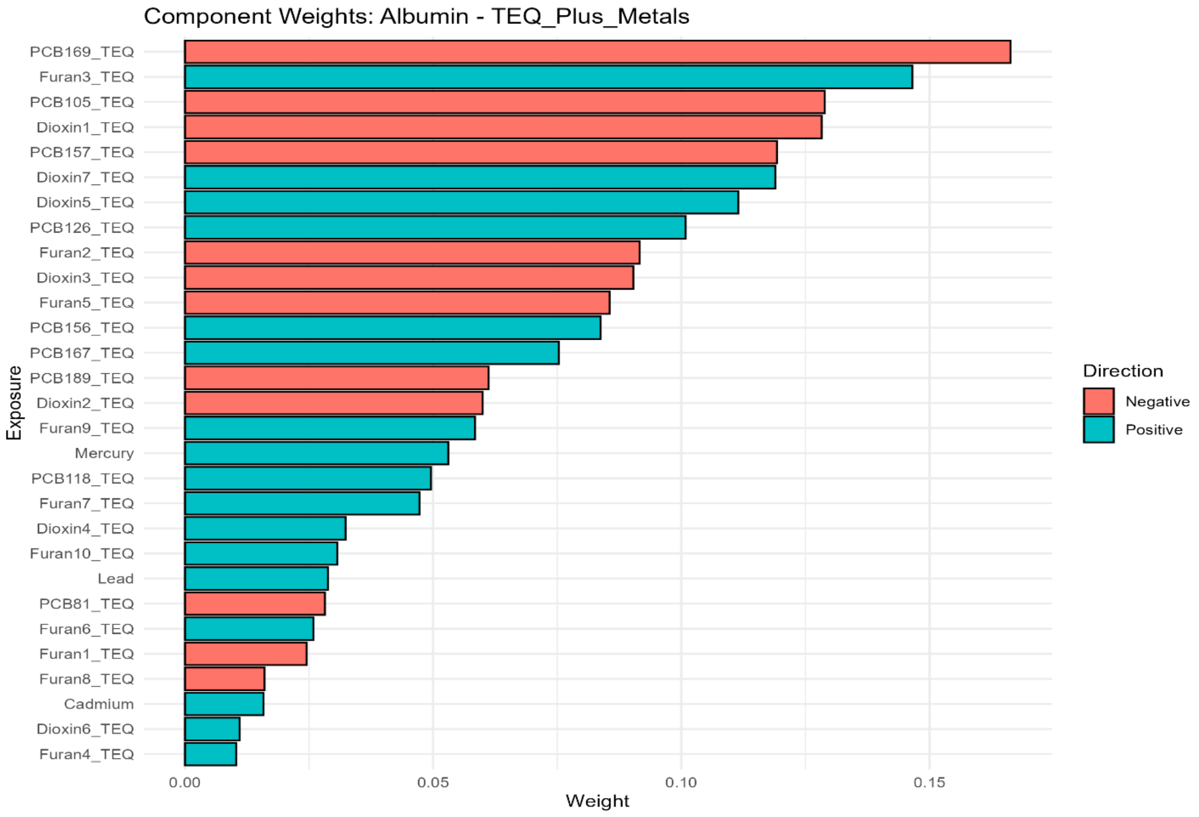

SUPPLEMENTARY DOCUMENTS

SUPPLEMENTARY DOCUMENTS

S83. ALP

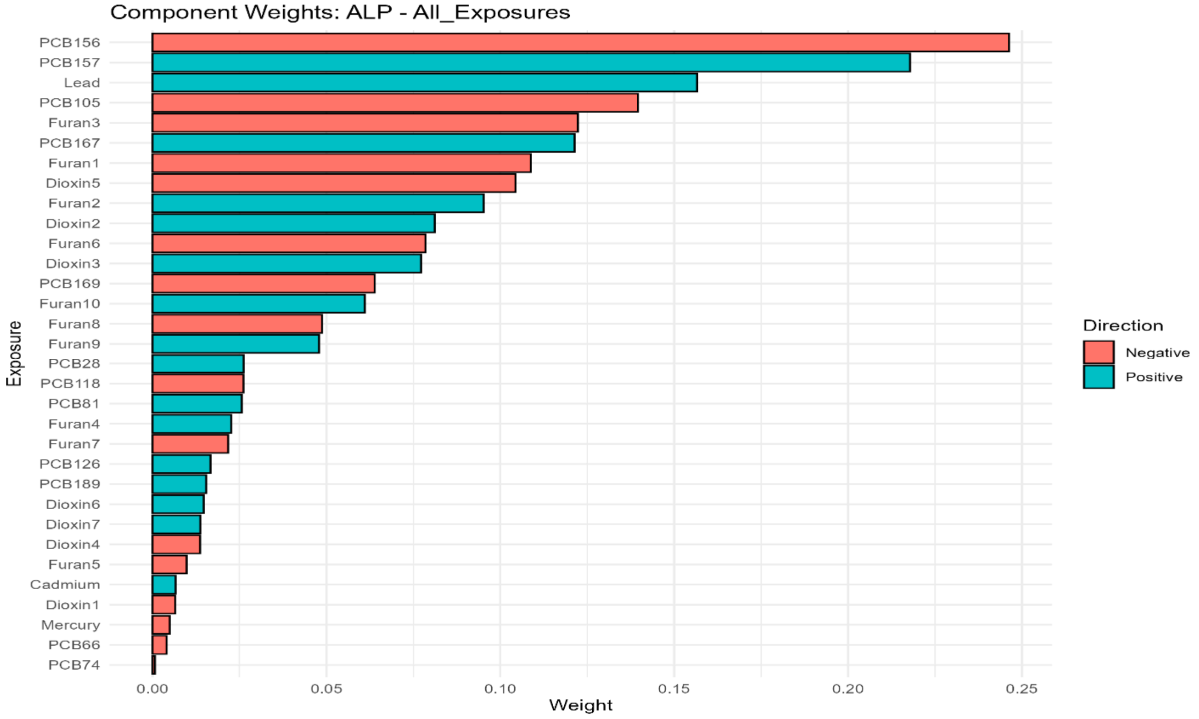

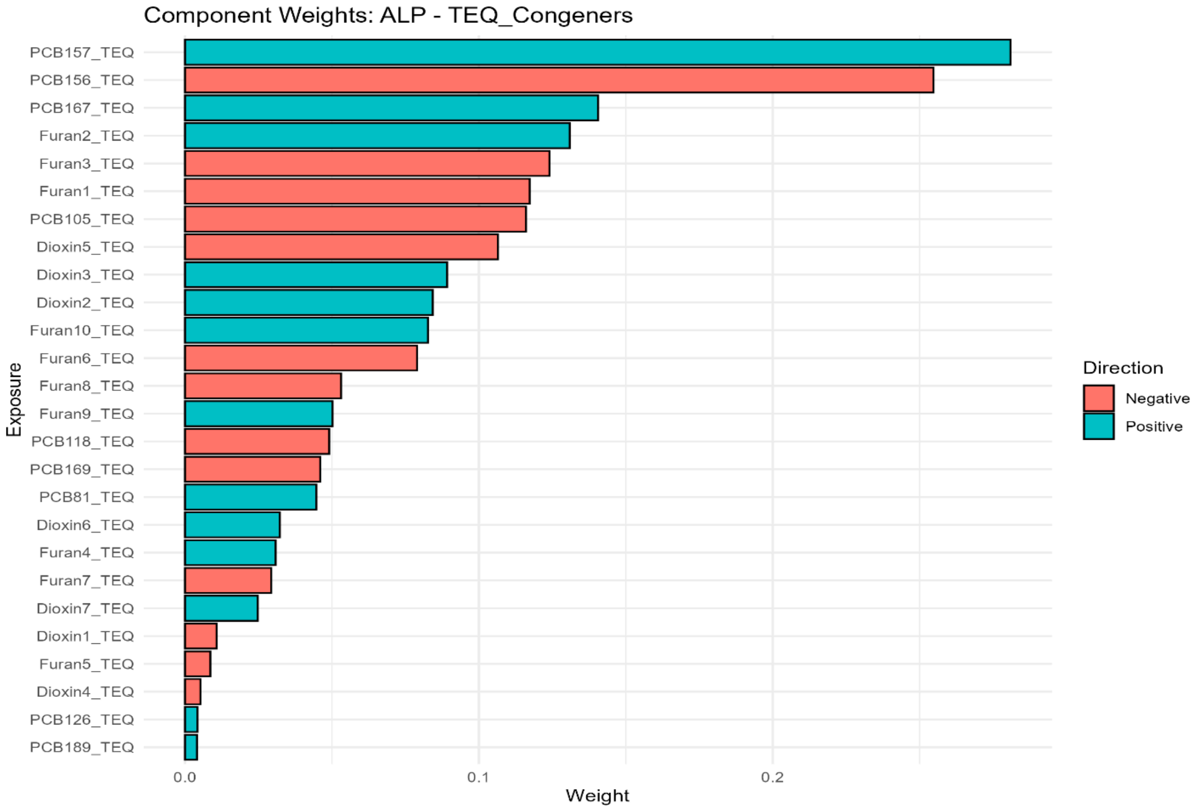

SUPPLEMENTARY DOCUMENTS

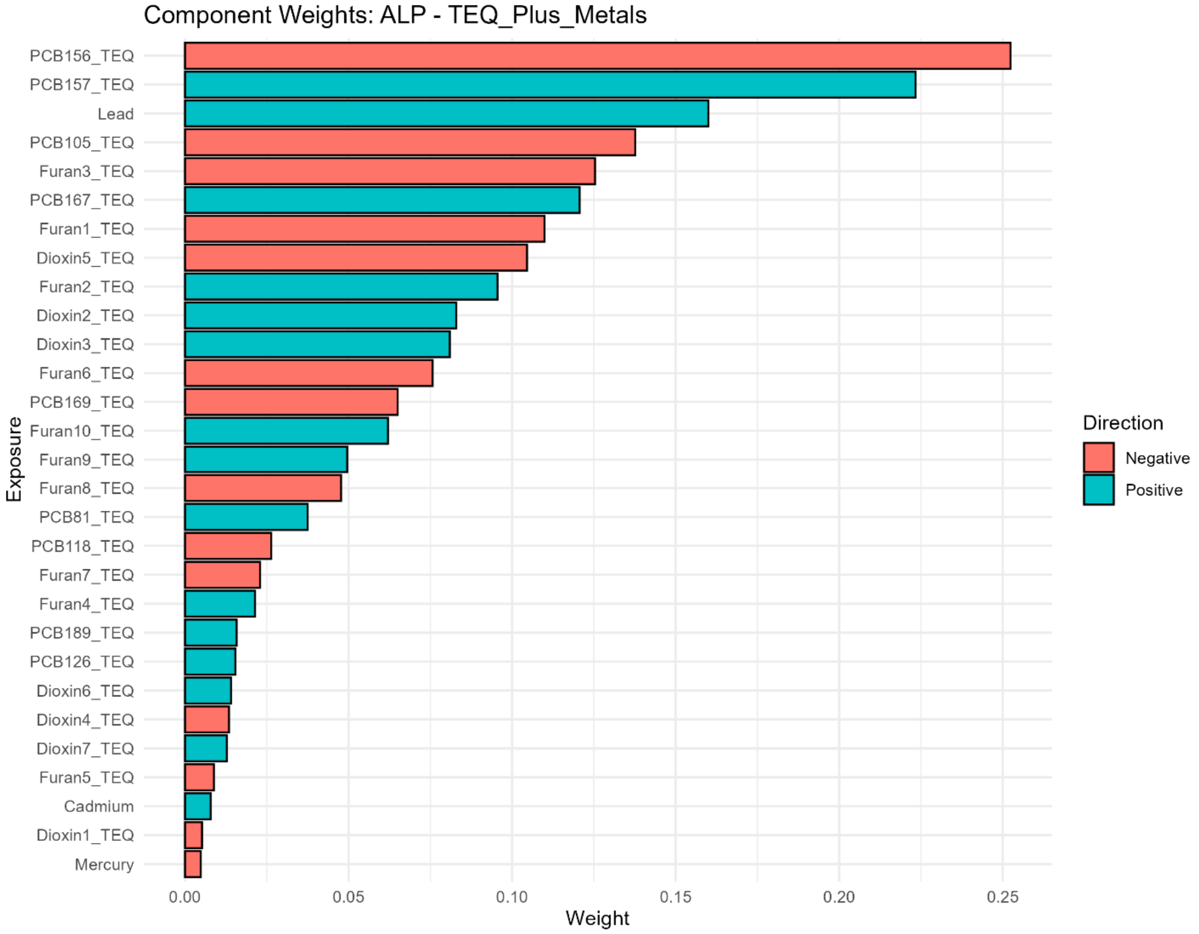

SUPPLEMENTARY DOCUMENTS

S84. ALT

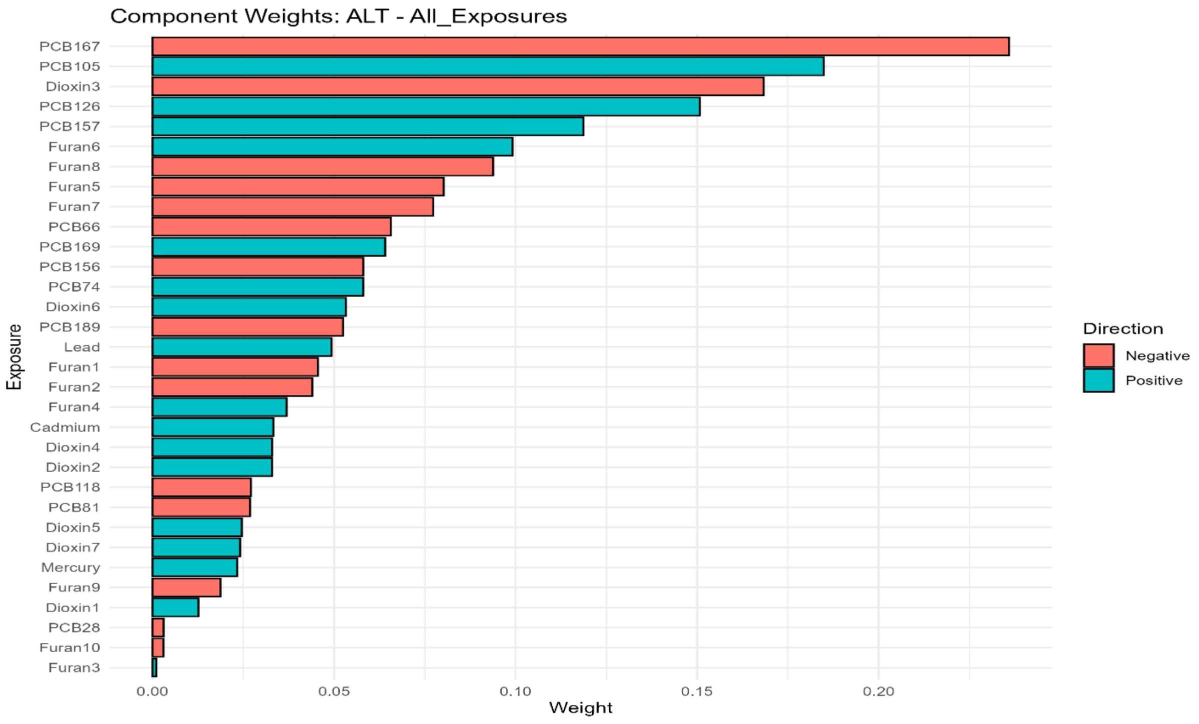

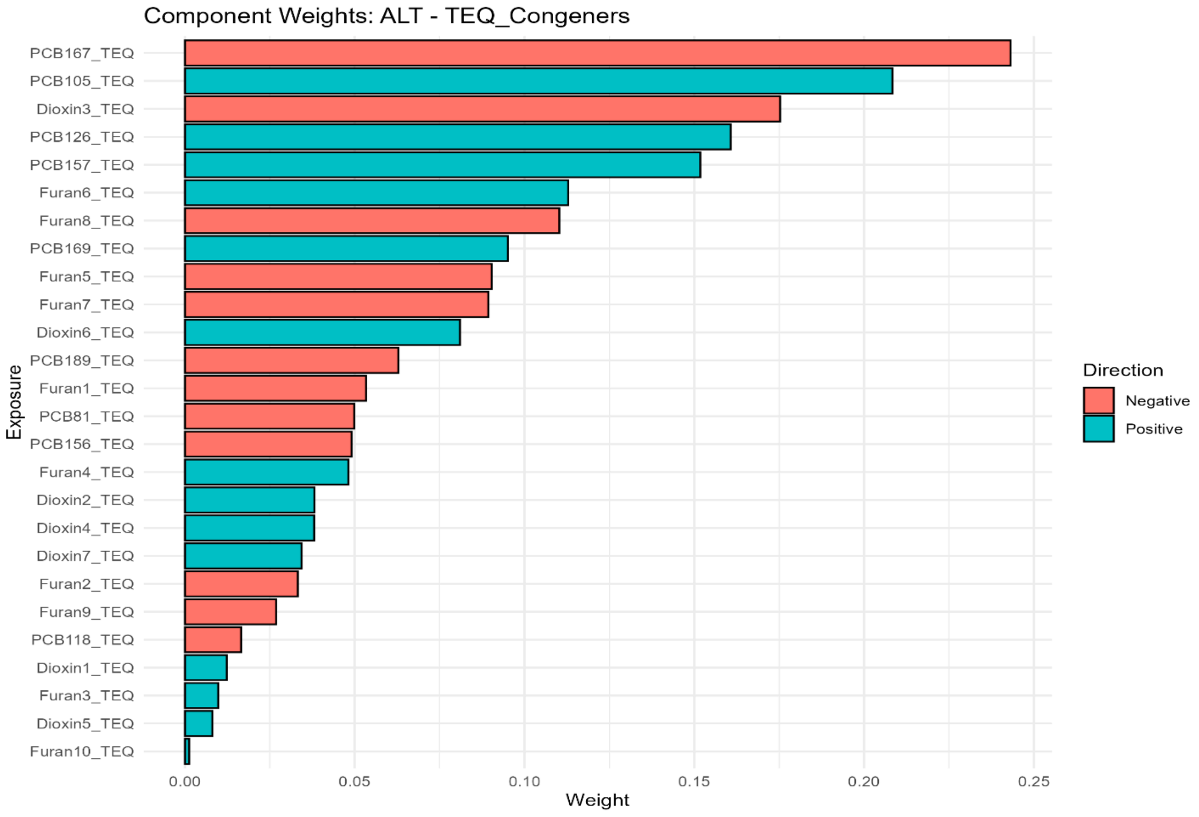

SUPPLEMENTARY DOCUMENTS

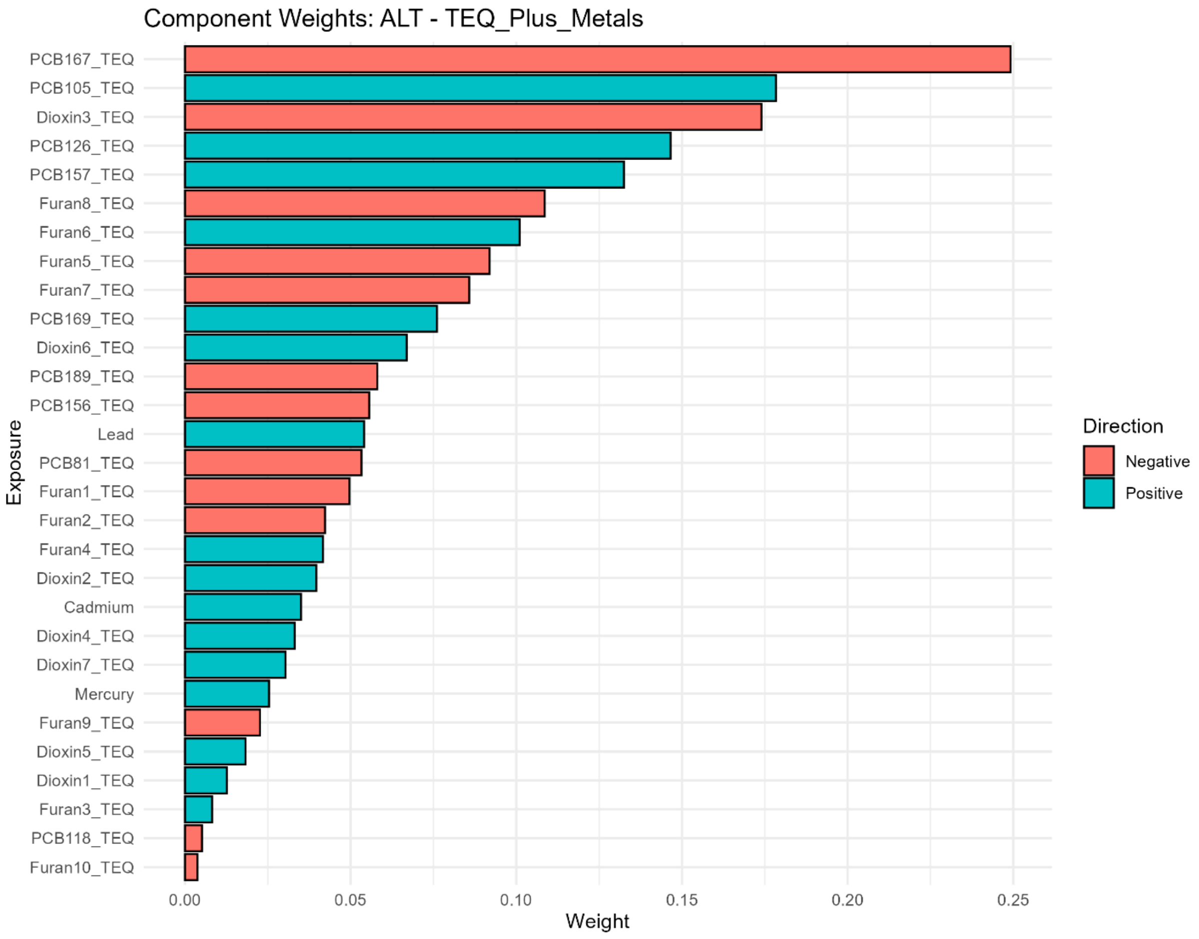

SUPPLEMENTARY DOCUMENTS

S85. AST

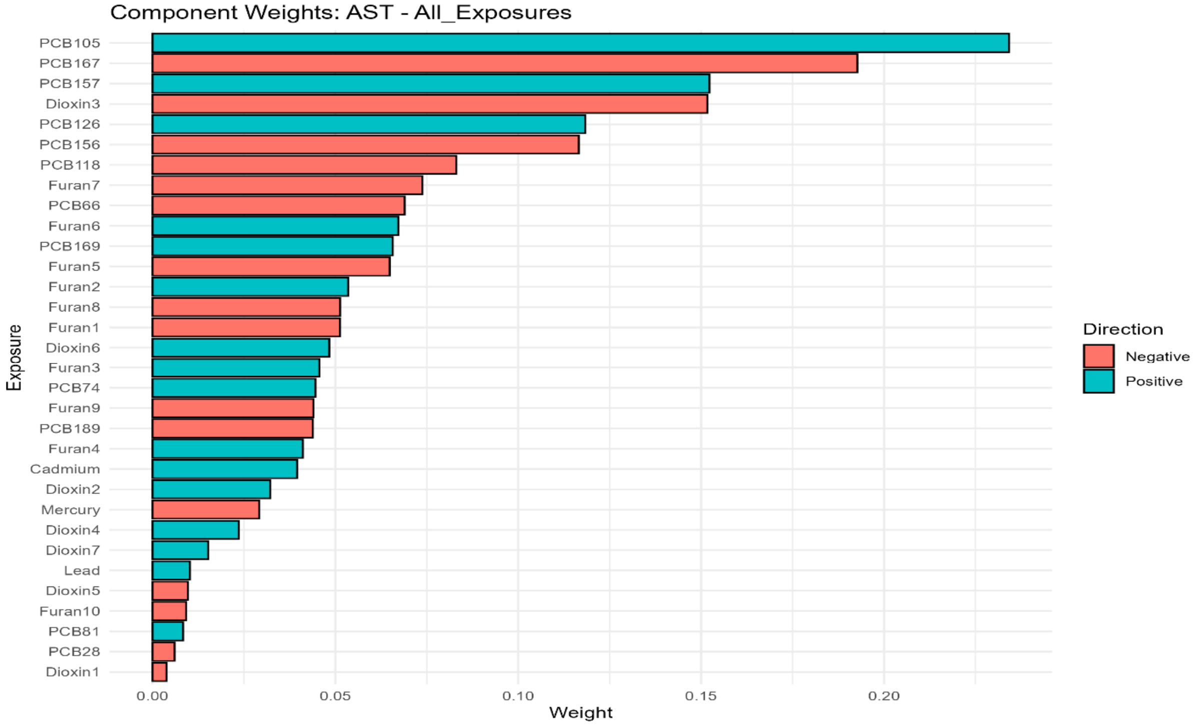

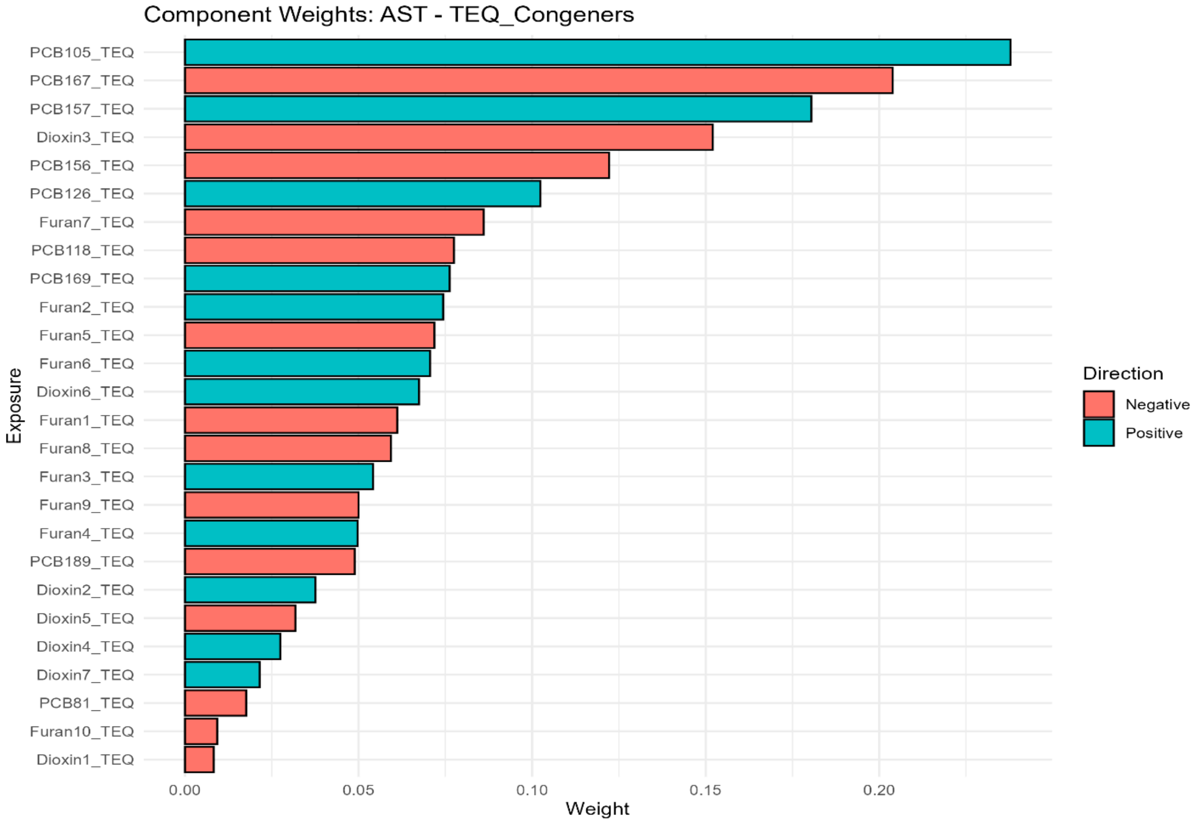

SUPPLEMENTARY DOCUMENTS

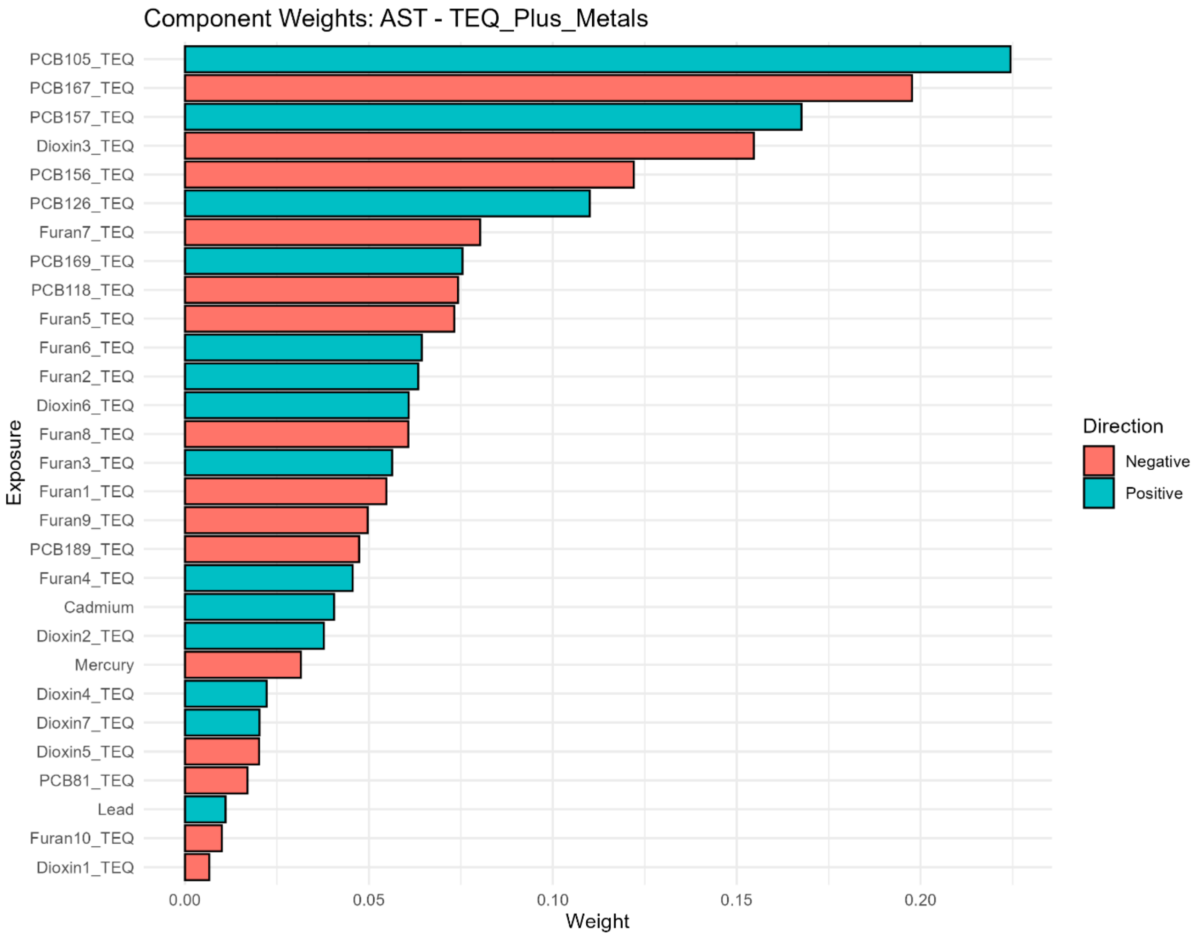

SUPPLEMENTARY DOCUMENTS

S86. GGT

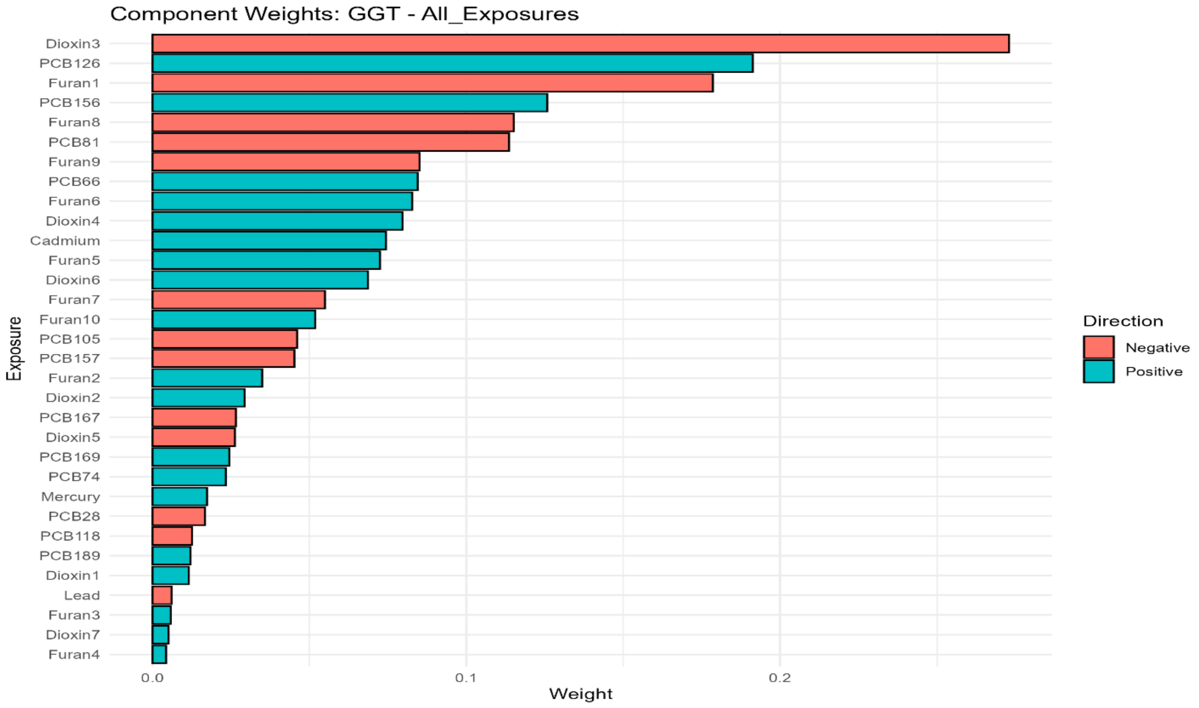

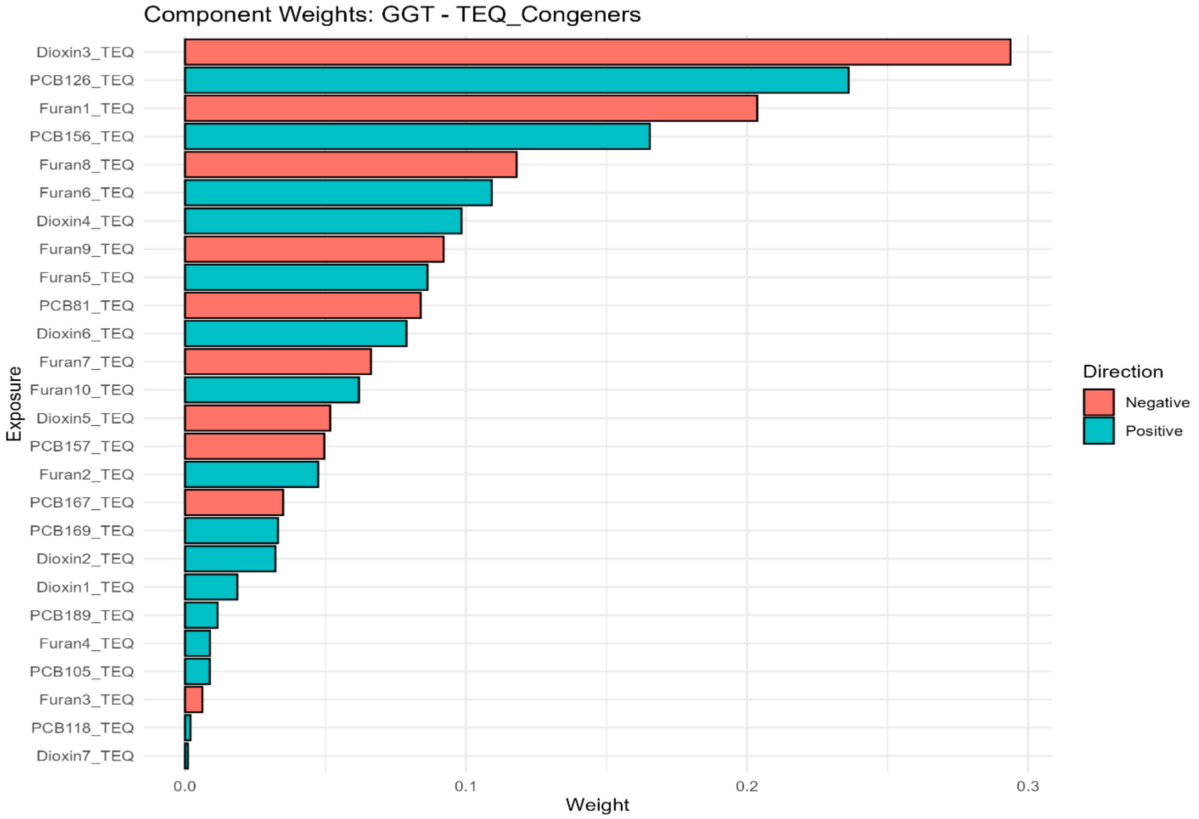

SUPPLEMENTARY DOCUMENTS

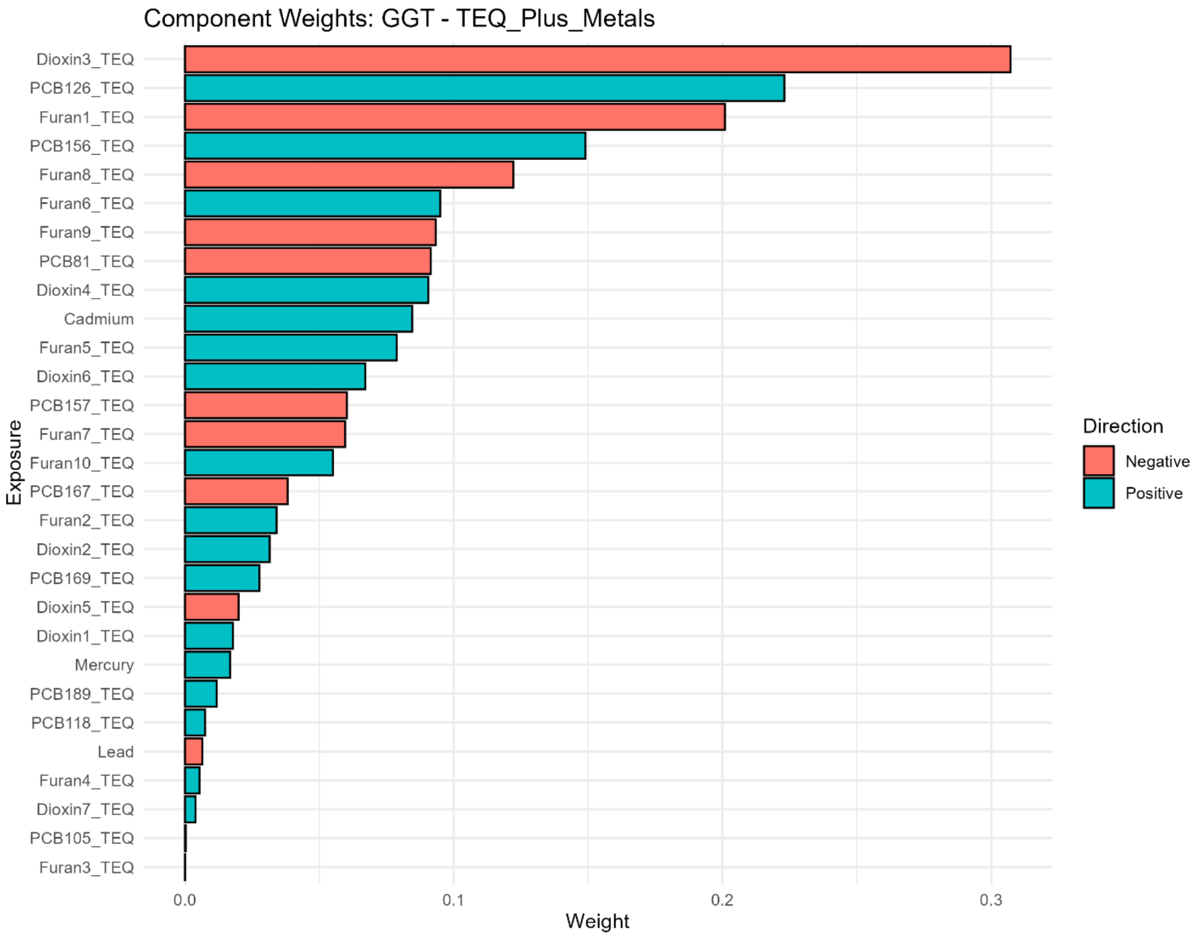

SUPPLEMENTARY DOCUMENTS

S87. LDH

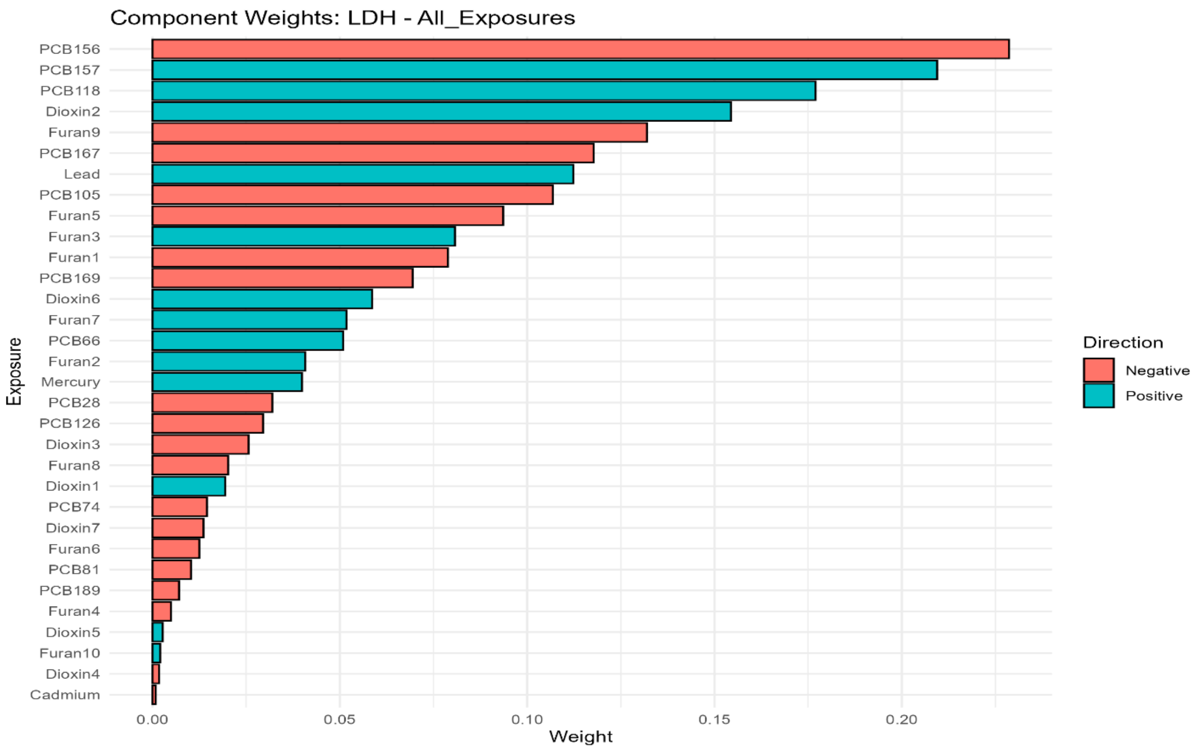

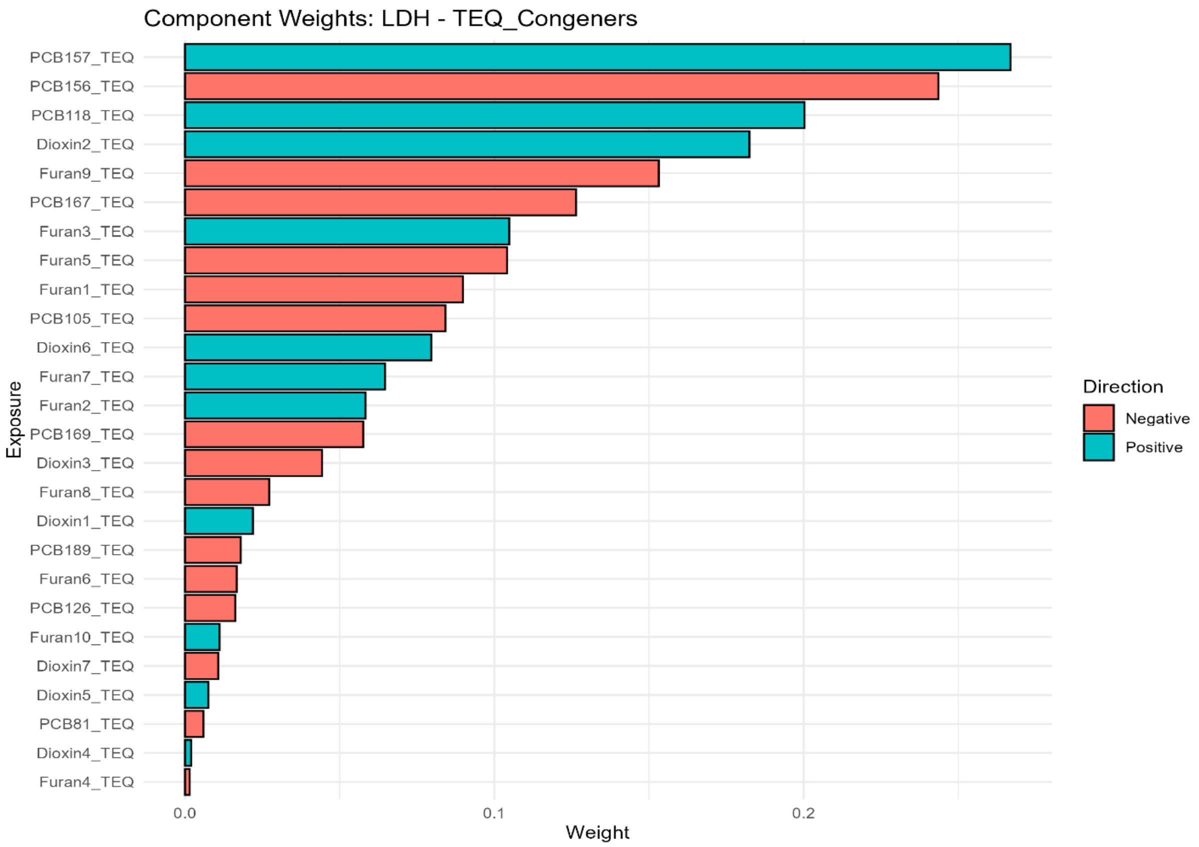

SUPPLEMENTARY DOCUMENTS

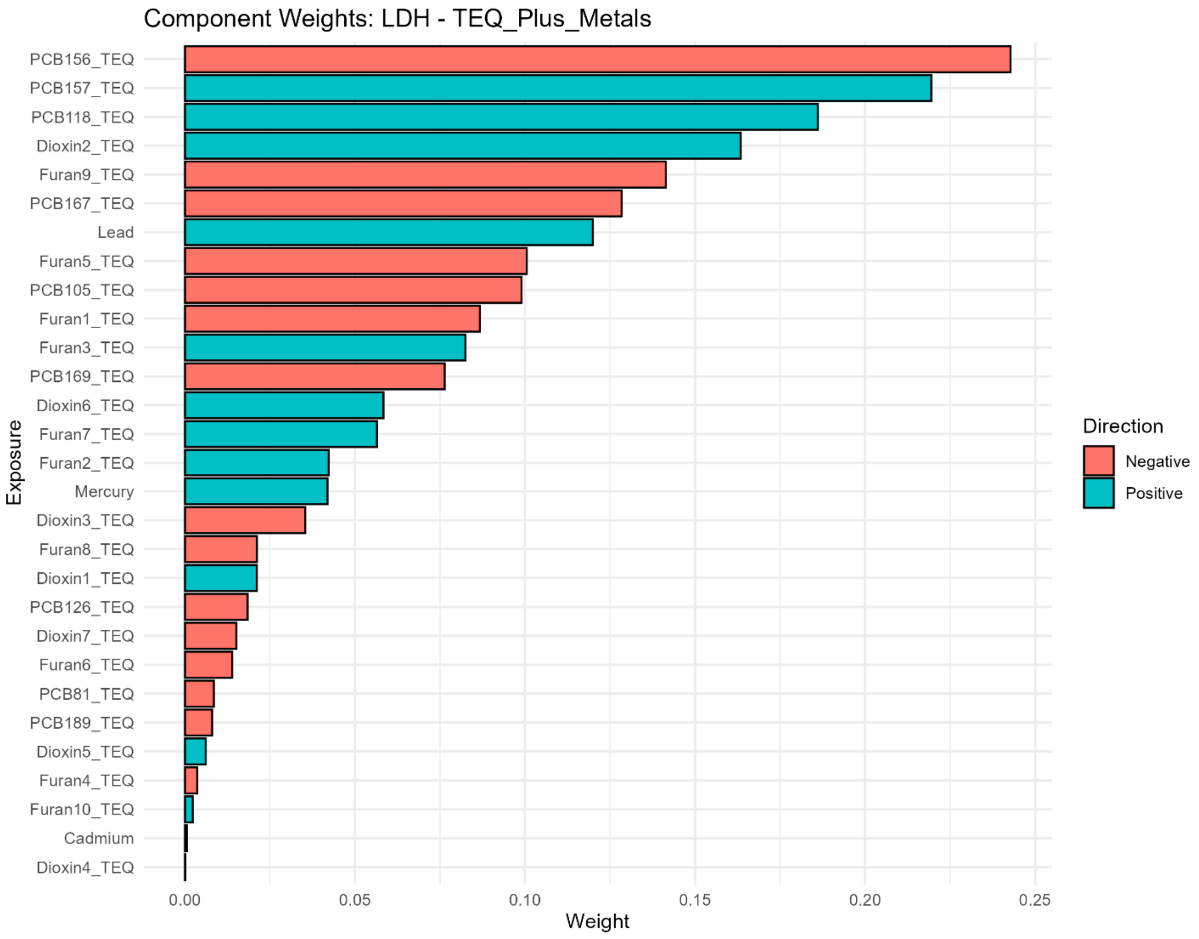

SUPPLEMENTARY DOCUMENTS

S88. TOTAL BILIRUBIN

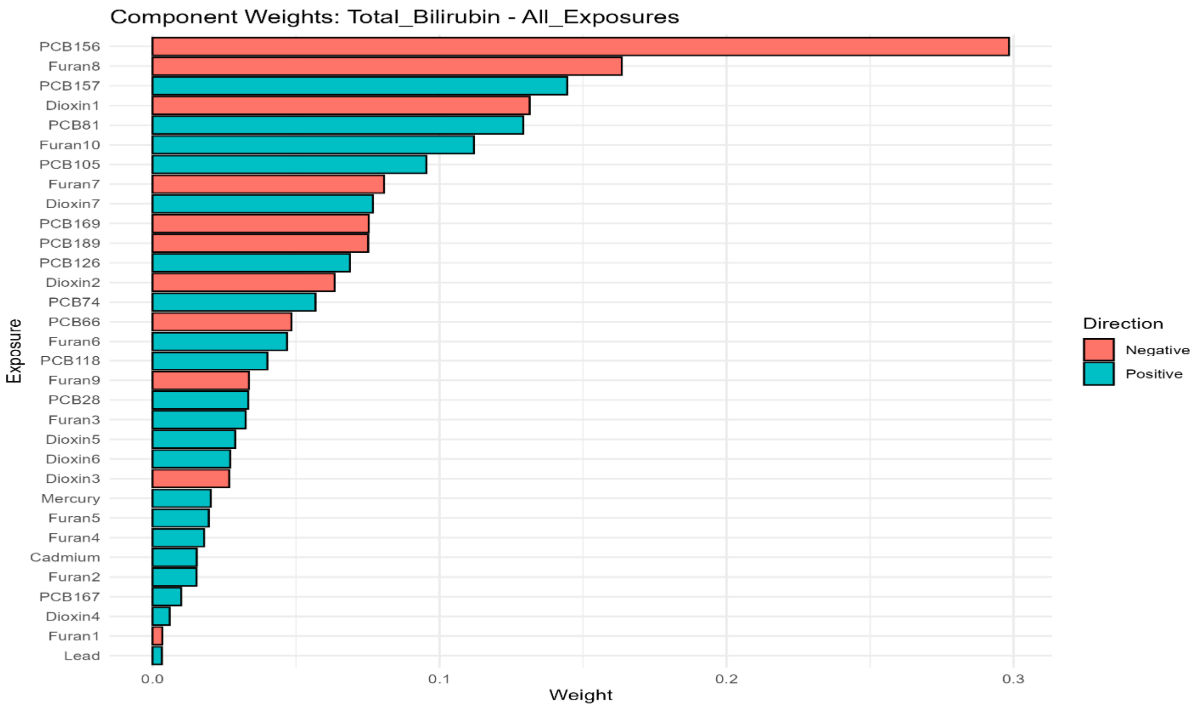

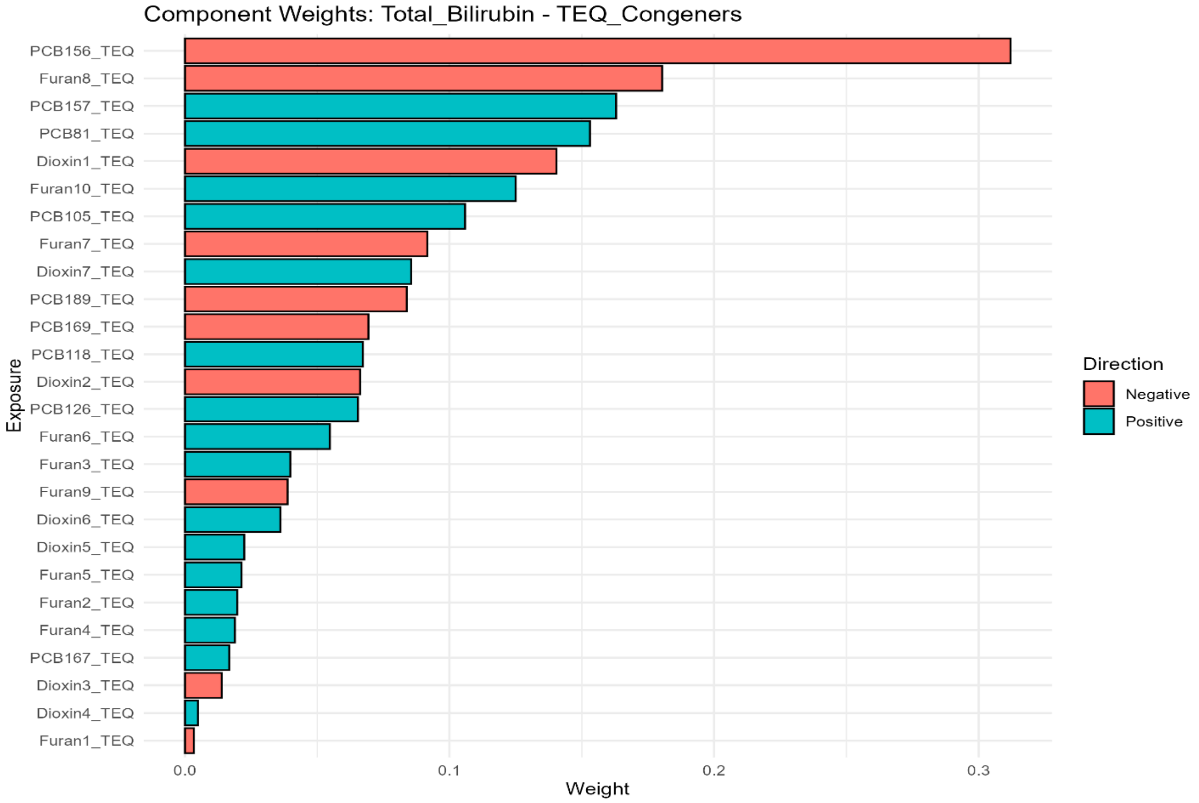

SUPPLEMENTARY DOCUMENTS

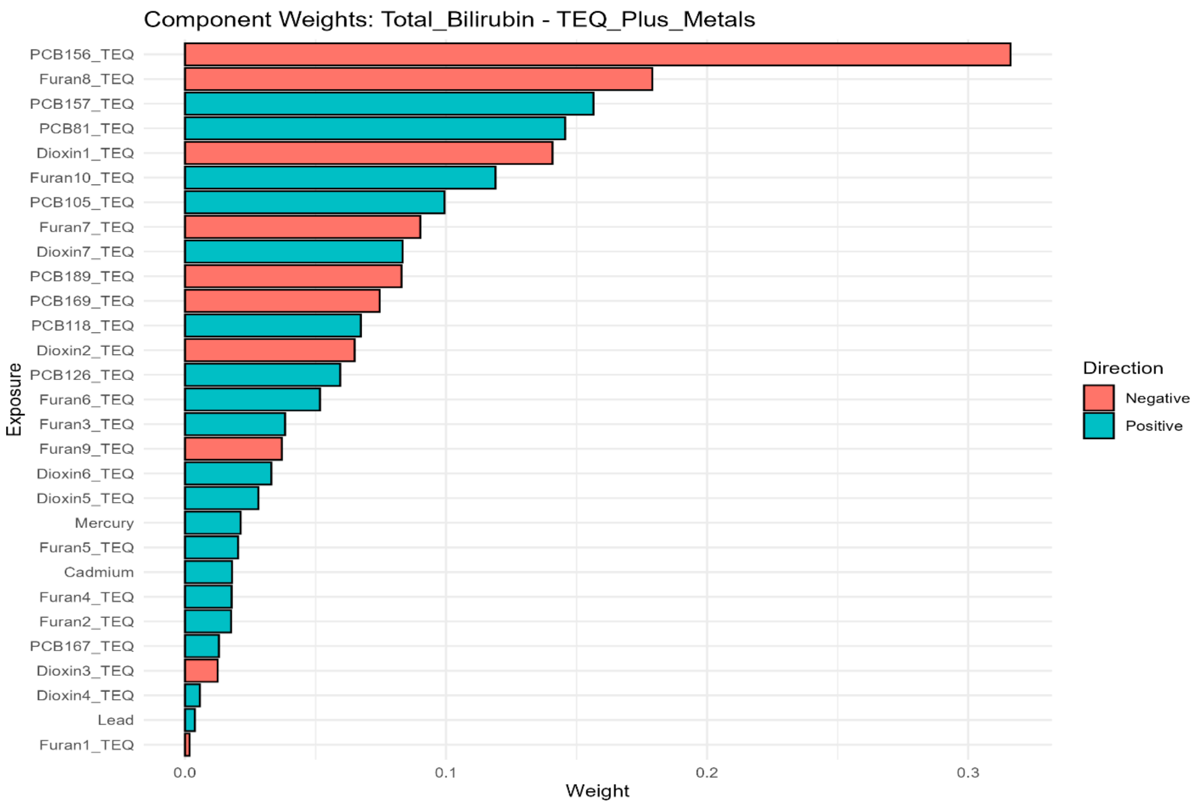

SUPPLEMENTARY DOCUMENTS

S89. TOTAL PROTEIN

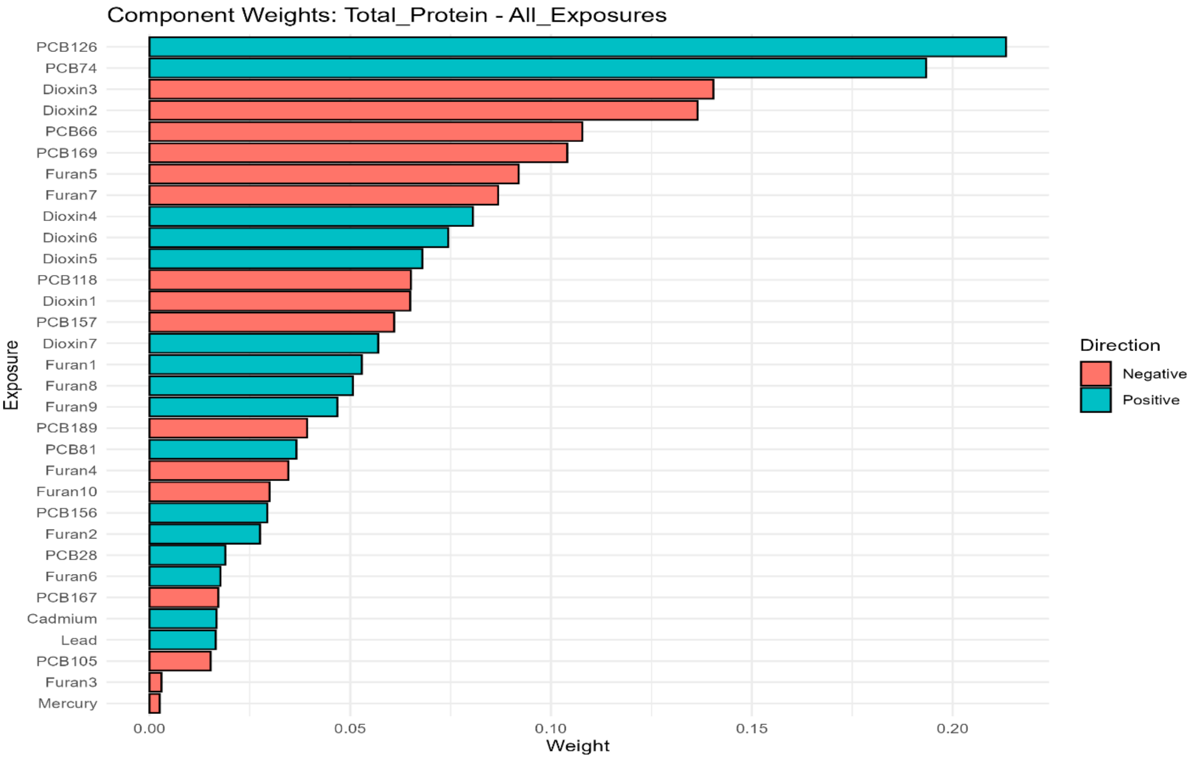

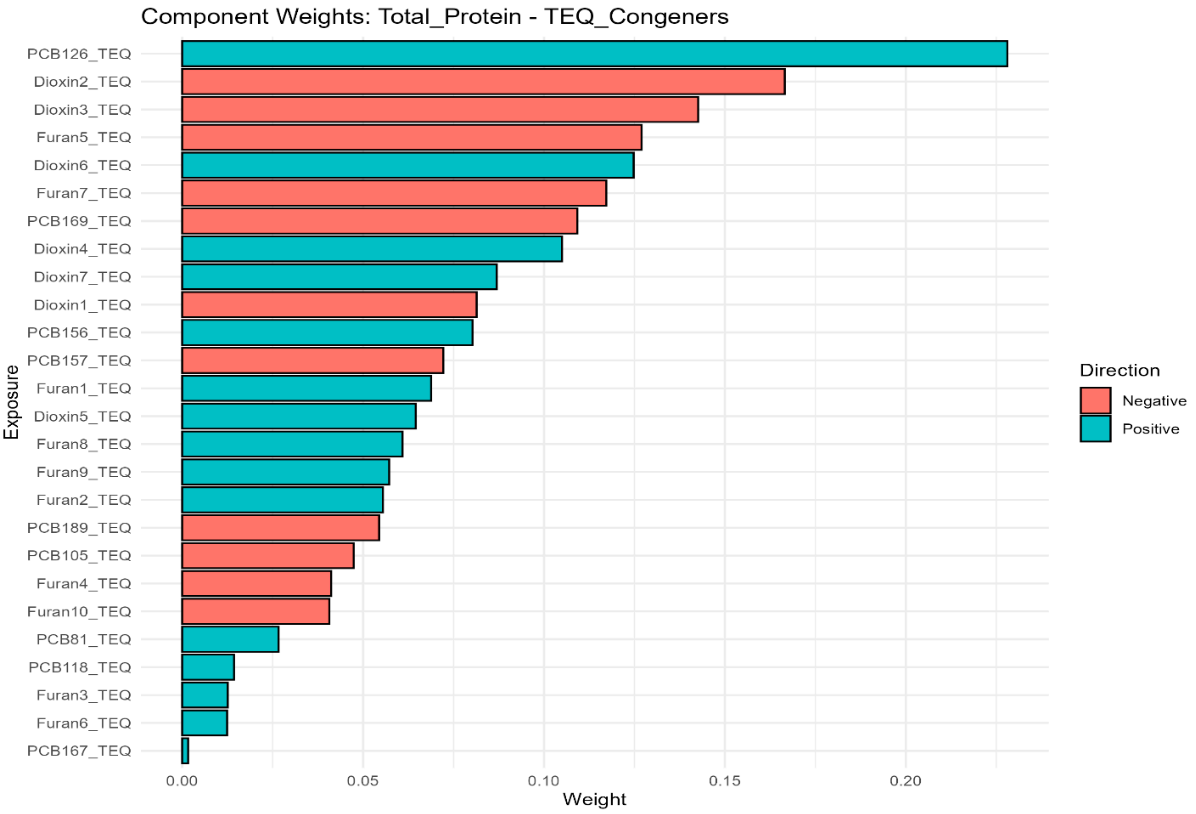

SUPPLEMENTARY DOCUMENTS

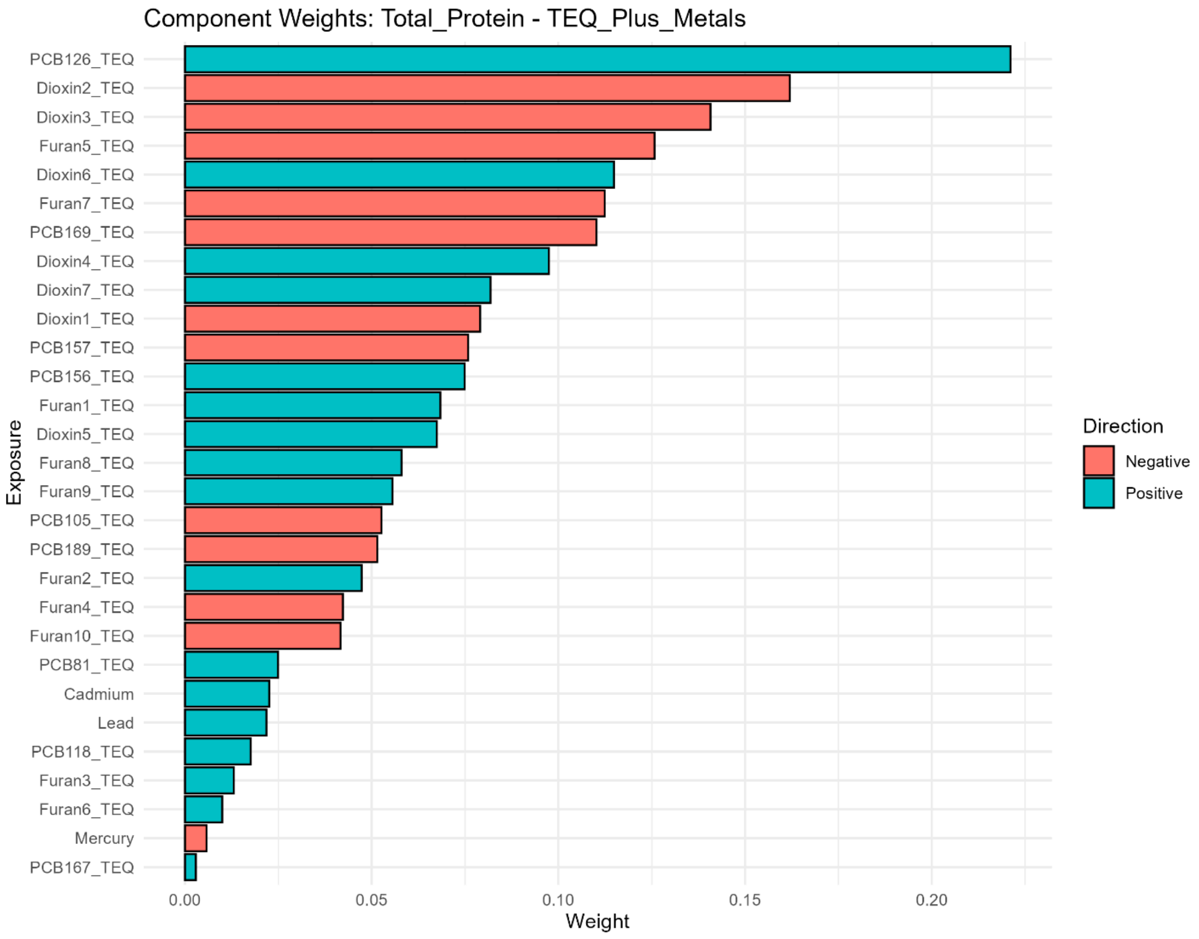

SUPPLEMENTARY DOCUMENTS

POSTERIOR INCLUSION PROBABILITY (PIP)

T S1. ALBUMIN

	VARIABLE	GROUP	GROUPPIP	CONDPIP
1	CADMIUM	1	0.00000	NAN
2	LEAD	1	0.00000	NAN
3	MERCURY	1	0.00000	NAN
4	PCB28	2	0.00056	0.00000000
5	PCB66	2	0.00056	0.00000000
6	PCB74	2	0.00056	0.00000000
7	PCB105	2	0.00056	0.00000000
8	PCB118	2	0.00056	0.00000000
9	PCB156	2	0.00056	0.00000000
10	PCB157	2	0.00056	0.00000000
11	PCB167	2	0.00056	0.00000000
12	PCB189	2	0.00056	0.00000000
13	PCB126	2	0.00056	0.00000000
14	PCB81	2	0.00056	0.00000000
15	PCB169	2	0.00056	1.00000000
16	DIOXIN1	3	0.01336	0.08383234
17	DIOXIN2	3	0.01336	0.00000000
18	DIOXIN3	3	0.01336	0.91616766
19	DIOXIN4	3	0.01336	0.00000000
20	DIOXIN5	3	0.01336	0.00000000
21	DIOXIN6	3	0.01336	0.00000000
22	DIOXIN7	3	0.01336	0.00000000
23	FURAN1	4	0.00000	NAN
24	FURAN2	4	0.00000	NAN
25	FURAN3	4	0.00000	NAN
26	FURAN4	4	0.00000	NAN
27	FURAN5	4	0.00000	NAN
28	FURAN6	4	0.00000	NAN
29	FURAN7	4	0.00000	NAN
30	FURAN8	4	0.00000	NAN
31	FURAN9	4	0.00000	NAN
32	FURAN10	4	0.00000	NAN

SUPPLEMENTARY DOCUMENTS

T S2. ALP

VARIABLE		GROUP	GROUPPIP	CONDPIP
**1**	CADMIUM	1	0.57096	0.0138013171
**2**	LEAD	1	0.57096	0.9234972678
**3**	MERCURY	1	0.57096	0.0627014152
**4**	PCB28	2	0.12952	0.0135886350
**5**	PCB66	2	0.12952	0.0172946263
**6**	PCB74	2	0.12952	0.0630018530
**7**	PCB105	2	0.12952	0.0043236566
**8**	PCB118	2	0.12952	0.0182211242
**9**	PCB156	2	0.12952	0.1970352069
**10**	PCB157	2	0.12952	0.0972822730
**11**	PCB167	2	0.12952	0.2041383570
**12**	PCB189	2	0.12952	0.0009264978
**13**	PCB126	2	0.12952	0.0067943175
**14**	PCB81	2	0.12952	0.0358245831
**15**	PCB169	2	0.12952	0.3415688697
**16**	DIOXIN1	3	0.06844	0.0356516657
**17**	DIOXIN2	3	0.06844	0.0888369375
**18**	DIOXIN3	3	0.06844	0.4617182934

**19**	DIOXIN4	3	0.06844	0.1876095850
**20**	DIOXIN5	3	0.06844	0.1098772648
**21**	DIOXIN6	3	0.06844	0.0905902981
**22**	DIOXIN7	3	0.06844	0.0257159556
**23**	FURAN1	4	0.14128	0.0928652322
**24**	FURAN2	4	0.14128	0.0266138165
**25**	FURAN3	4	0.14128	0.5101925255
**26**	FURAN4	4	0.14128	0.0254813137
**27**	FURAN5	4	0.14128	0.0948471121
**28**	FURAN6	4	0.14128	0.0291619479
**29**	FURAN7	4	0.14128	0.0158550396
**30**	FURAN8	4	0.14128	0.0000000000
**31**	FURAN9	4	0.14128	0.0855039638
**32**	FURAN10	4	0.14128	0.119479048

SUPPLEMENTARY DOCUMENTS

T S3.   ALT

**VARIABLE**		**GROUP**	**GROUPPIP**	**CONDPIP**
**1**	CADMIUM	1	0.61964	0.024982248
**2**	LEAD	1	0.61964	0.034988058
**3**	MERCURY	1	0.61964	0.940029695
**4**	PCB28	2	0.23860	0.012740989
**5**	PCB66	2	0.23860	0.012573345
**6**	PCB74	2	0.23860	0.026152557
**7**	PCB105	2	0.23860	0.297401509
**8**	PCB118	2	0.23860	0.040234702
**9**	PCB156	2	0.23860	0.047275775
**10**	PCB157	2	0.23860	0.033025985
**11**	PCB167	2	0.23860	0.026990780
**12**	PCB189	2	0.23860	0.015926236
**13**	PCB126	2	0.23860	0.456328583
**14**	PCB81	2	0.23860	0.007208718
**15**	PCB169	2	0.23860	0.024140821
**16**	DIOXIN1	3	0.05712	0.075630252
**17**	DIOXIN2	3	0.05712	0.080532213

**18**	DIOXIN3	3	0.05712	0.474789916
**19**	DIOXIN4	3	0.05712	0.061624650
**20**	DIOXIN5	3	0.05712	0.117647059
**21**	DIOXIN6	3	0.05712	0.067226891
**22**	DIOXIN7	3	0.05712	0.122549020
**23**	FURAN1	4	0.25348	0.080321919
**24**	FURAN2	4	0.25348	0.185418968
**25**	FURAN3	4	0.25348	0.017200568
**26**	FURAN4	4	0.25348	0.014833517
**27**	FURAN5	4	0.25348	0.111724791
**28**	FURAN6	4	0.25348	0.031560675
**29**	FURAN7	4	0.25348	0.071800537
**30**	FURAN8	4	0.25348	0.409973173
**31**	FURAN9	4	0.25348	0.051128294
**32**	FURAN10	4	0.25348	0.026037557

SUPPLEMENTARY DOCUMENTS

T S4. AST

VARIABLE		GROUP	GROUPPIP	CONDPIP
**1**	CADMIUM	1	0.01100	7.563636E-01
**2**	LEAD	1	0.01100	2.909091E-02
**3**	MERCURY	1	0.01100	2.145455E-01
**4**	PCB28	2	0.95100	9.859096E-01
**5**	PCB66	2	0.95100	8.412198E-05
**6**	PCB74	2	0.95100	3.364879E-04
**7**	PCB105	2	0.95100	6.351209E-03
**8**	PCB118	2	0.95100	1.093586E-03
**9**	PCB156	2	0.95100	1.682440E-03
**10**	PCB157	2	0.95100	1.219769E-03
**11**	PCB167	2	0.95100	1.261830E-04
**12**	PCB189	2	0.95100	0.000000E+00
**13**	PCB126	2	0.95100	2.986330E-03
**14**	PCB81	2	0.95100	2.103049E-04
**15**	PCB169	2	0.95100	0.000000E+00
**16**	DIOXIN1	3	0.00872	1.284404E-01
**17**	DIOXIN2	3	0.00872	0.000000E+00
**18**	DIOXIN3	3	0.00872	2.385321E-01

**19**	DIOXIN4	3	0.00872	5.137615E-01
**20**	DIOXIN5	3	0.00872	4.587156E-03
**21**	DIOXIN6	3	0.00872	2.752294E-02
**22**	DIOXIN7	3	0.00872	8.715596E-02
**23**	FURAN1	4	0.00896	4.464286E-03
**24**	FURAN2	4	0.00896	1.785714E-02
**25**	FURAN3	4	0.00896	3.035714E-01
**26**	FURAN4	4	0.00896	1.785714E-02
**27**	FURAN5	4	0.00896	2.633929E-01
**28**	FURAN6	4	0.00896	3.571429E-02
**29**	FURAN7	4	0.00896	1.562500E-01
**30**	FURAN8	4	0.00896	8.928571E-02
**31**	FURAN9	4	0.00896	8.035714E-02
**32**	FURAN10	4	0.00896	3.125000E-02

SUPPLEMENTARY DOCUMENTS

T S5. GGT

VARIABLE		GROUP	GROUPPIP	CONDPIP
**1**	CADMIUM	1	0.60528	0.33498546
**2**	LEAD	1	0.60528	0.30372720
**3**	MERCURY	1	0.60528	0.36128734
**4**	PCB28	2	0.63592	0.07132973
**5**	PCB66	2	0.63592	0.08076488
**6**	PCB74	2	0.63592	0.09013712
**7**	PCB105	2	0.63592	0.08365832
**8**	PCB118	2	0.63592	0.08497924
**9**	PCB156	2	0.63592	0.08894201
**10**	PCB157	2	0.63592	0.09026293
**11**	PCB167	2	0.63592	0.07831174
**12**	PCB189	2	0.63592	0.07787143
**13**	PCB126	2	0.63592	0.08768399
**14**	PCB81	2	0.63592	0.07969556
**15**	PCB169	2	0.63592	0.08636306
**16**	DIOXIN1	3	0.61904	0.13343241
**17**	DIOXIN2	3	0.61904	0.14654950
**18**	DIOXIN3	3	0.61904	0.15107263

**19**	DIOXIN4	3	0.61904	0.14900491
**20**	DIOXIN5	3	0.61904	0.12755234
**21**	DIOXIN6	3	0.61904	0.13640476
**22**	DIOXIN7	3	0.61904	0.15598346
**23**	FURAN1	4	0.60612	0.11014321
**24**	FURAN2	4	0.60612	0.09450274
**25**	FURAN3	4	0.60612	0.11661057
**26**	FURAN4	4	0.60612	0.10275193
**27**	FURAN5	4	0.60612	0.09720847
**28**	FURAN6	4	0.60612	0.10545767
**29**	FURAN7	4	0.60612	0.08051211
**30**	FURAN8	4	0.60612	0.08664951
**31**	FURAN9	4	0.60612	0.10189401
**32**	FURAN10	4	0.60612	0.10426978

T S6. LDH

VARIABLE		GROUP	GROUPPIP	CONDPIP
**1**	CADMIUM	1	0.08704	0.984834559
**2**	LEAD	1	0.08704	0.003676471
**3**	MERCURY	1	0.08704	0.011488971
**4**	PCB28	2	0.06740	0.058160237
**5**	PCB66	2	0.06740	0.009495549
**6**	PCB74	2	0.06740	0.000000000
**7**	PCB105	2	0.06740	0.000000000
**8**	PCB118	2	0.06740	0.001186944
**9**	PCB156	2	0.06740	0.027299703
**10**	PCB157	2	0.06740	0.021958457
**11**	PCB167	2	0.06740	0.010682493
**12**	PCB189	2	0.06740	0.736498516
**13**	PCB126	2	0.06740	0.000000000
**14**	PCB81	2	0.06740	0.082492582
**15**	PCB169	2	0.06740	0.052225519
**16**	DIOXIN1	3	0.01972	0.162271805
**17**	DIOXIN2	3	0.01972	0.415821501
**18**	DIOXIN3	3	0.01972	0.255578093

**19**	DIOXIN4	3	0.01972	0.022312373
**20**	DIOXIN5	3	0.01972	0.068965517
**21**	DIOXIN6	3	0.01972	0.073022312
**22**	DIOXIN7	3	0.01972	0.002028398
**23**	FURAN1	4	0.01200	0.070000000
**24**	FURAN2	4	0.01200	0.000000000
**25**	FURAN3	4	0.01200	0.096666667
**26**	FURAN4	4	0.01200	0.156666667
**27**	FURAN5	4	0.01200	0.053333333
**28**	FURAN6	4	0.01200	0.020000000
**29**	FURAN7	4	0.01200	0.446666667
**30**	FURAN8	4	0.01200	0.000000000
**31**	FURAN9	4	0.01200	0.056666667
**32**	FURAN10	4	0.01200	0.100000000

SUPPLEMENTARY DOCUMENTS

T S7. TOTAL BILIRUBIN

VARIABLE		GROUP	GROUPPIP	CONDPIP
**1**	CADMIUM	1	0.01968	7.723577E-01
**2**	LEAD	1	0.01968	1.239837E-01
**3**	MERCURY	1	0.01968	1.036585E-01
**4**	PCB28	2	0.90748	1.057875E-02
**5**	PCB66	2	0.90748	5.747785E-02
**6**	PCB74	2	0.90748	1.234187E-03
**7**	PCB105	2	0.90748	0.000000E+00
**8**	PCB118	2	0.90748	1.322343E-04
**9**	PCB156	2	0.90748	8.815621E-05
**10**	PCB157	2	0.90748	4.407811E-05
**11**	PCB167	2	0.90748	5.289373E-04
**12**	PCB189	2	0.90748	1.322343E-04
**13**	PCB126	2	0.90748	5.694891E-02
**14**	PCB81	2	0.90748	8.717768E-01
**15**	PCB169	2	0.90748	1.057875E-03
**16**	DIOXIN1	3	0.10992	4.294032E-02
**17**	DIOXIN2	3	0.10992	2.147016E-02
**18**	DIOXIN3	3	0.10992	6.826783E-01

**19**	DIOXIN4	3	0.10992	8.078603E-02
**20**	DIOXIN5	3	0.10992	1.026201E-01
**21**	DIOXIN6	3	0.10992	2.583697E-02
**22**	DIOXIN7	3	0.10992	4.366812E-02
**23**	FURAN1	4	0.01408	0.000000E+00
**24**	FURAN2	4	0.01408	0.000000E+00
**25**	FURAN3	4	0.01408	0.000000E+00
**26**	FURAN4	4	0.01408	4.261364E-02
**27**	FURAN5	4	0.01408	0.000000E+00
**28**	FURAN6	4	0.01408	3.125000E-02
**29**	FURAN7	4	0.01408	2.840909E-03
**30**	FURAN8	4	0.01408	1.846591E-01
**31**	FURAN9	4	0.01408	7.670455E-02
**32**	FURAN10	4	0.01408	6.619318E-01

SUPPLEMENTARY DOCUMENTS

T S8. TOTAL PROTEIN

**VARIABLE**		**GROUP**	**GROUPPIP**	**CONDPIP**
**1**	CADMIUM	1	0	NAN
**2**	LEAD	1	0	NAN
**3**	MERCURY	1	0	NAN
**4**	PCB28	2	0	NAN
**5**	PCB66	2	0	NAN
**6**	PCB74	2	0	NAN
**7**	PCB105	2	0	NAN
**8**	PCB118	2	0	NAN
**9**	PCB156	2	0	NAN
**10**	PCB157	2	0	NAN
**11**	PCB167	2	0	NAN
**12**	PCB189	2	0	NAN
**13**	PCB126	2	0	NAN
**14**	PCB81	2	0	NAN
**15**	PCB169	2	0	NAN
**16**	DIOXIN1	3	0	NAN
**17**	DIOXIN2	3	0	NAN

**18**	DIOXIN3	3	0	NAN
**19**	DIOXIN4	3	0	NAN
**20**	DIOXIN5	3	0	NAN
**21**	DIOXIN6	3	0	NAN
**22**	DIOXIN7	3	0	NAN
**23**	FURAN1	4	0	NAN
**24**	FURAN2	4	0	NAN
**25**	FURAN3	4	0	NAN
**26**	FURAN4	4	0	NAN
**27**	FURAN5	4	0	NAN
**28**	FURAN6	4	0	NAN
**29**	FURAN7	4	0	NAN
**30**	FURAN8	4	0	NAN
**31**	FURAN9	4	0	NAN
**32**	FURAN10	4	0	NAN
